# Tailoring a novel colorectal cancer stem cell-targeted therapy by inhibiting the SMYD3/c-MYC axis

**DOI:** 10.1038/s41392-025-02290-z

**Published:** 2025-06-30

**Authors:** Martina Lepore Signorile, Elisabetta Di Nicola, Giovanna Forte, Paola Sanese, Candida Fasano, Vittoria Disciglio, Katia De Marco, Marialaura Latrofa, Loris De Cecco, Marica Ficorilli, Marta Lucchetta, Erica Torchia, Chiara Dossena, Giusy Bianco, Vito Spilotro, Claudia Ferroni, Nicoletta Labarile, Raffaele Armentano, Francesco Albano, Anna Mestice, Gianluigi Gigante, Valerio Lantone, Giuliano Lantone, Leonardo Vincenti, Alberto Del Rio, Greta Varchi, Valentina Grossi, Cristiano Simone

**Affiliations:** 1 Medical Genetics, National Institute of Gastroenterology, IRCCS “Saverio de Bellis” Research Hospital, Castellana Grotte, BA Italy; 2 Integrated Biology of Rare Tumors, Department of Experimental Oncology, Fondazione IRCCS Istituto Nazionale dei Tumori, Milano, Italy; 3 Animal facility, National Institute of Gastroenterology, IRCCS “Saverio de Bellis” Research Hospital, Castellana Grotte, BA Italy; 4 Institute for Organic Synthesis and Photoreactivity, National Research Council, Bologna, Italy; 5 Histopathology Unit, National Institute of Gastroenterology, IRCCS “Saverio de Bellis” Research Hospital, Castellana Grotte, BA Italy; 6 Department of Precision and Regenerative Medicine and Jonic Area (DiMePRe-J), University of Bari Aldo Moro, Bari, Italy; 7 Department of General Surgery, National Institute of Gastroenterology, IRCCS “Saverio de Bellis” Research Hospital, Castellana Grotte, BA Italy; 8 Unit of Surgery, “Lorenzo Bonomo” Hospital, Andria, BAT Italy; 9 Innovamol Srl, Modena, Italy

**Keywords:** Gastrointestinal cancer, Cancer stem cells

## Abstract

Cancer stem cells (CSCs) are responsible for colorectal cancer (CRC) chemoresistance, recurrence, and metastasis. Therefore, identifying molecular stemness targets that are involved in tumor growth is crucial for effective treatment. Here, we performed an extensive in vitro and in vivo molecular and functional characterization, revealing the pivotal role of the lysine methyltransferase SET and MYND Domain Containing 3 (SMYD3) in colorectal cancer stem cell (CRC-SC) biology. Specifically, we showed that SMYD3 interacts with and methylates c-MYC at K158 and K163, thereby modulating its transcriptional activity, which is implicated in stemness and colorectal malignancy. Our in vitro data suggest that SMYD3 pharmacological inhibition or its stable genetic ablation affects the clonogenic and self-renewal potential of patient-derived CRC-SCs and organoids by altering their molecular signature. Moreover, we found that SMYD3 stable knock-out or pharmacological inhibition drastically reduces CRC tumorigenicity in vivo and CRC-SC metastatic potential. Overall, our findings identify SMYD3 as a promising therapeutic target acting directly on c-MYC, with potential implications for countering CRC-SC proliferation and metastatic dissemination.

## Introduction

Colorectal cancer (CRC) is the second leading cause of cancer death worldwide,^1^ with only 13% of patients with advanced and metastatic disease surviving up to five years from diagnosis.^2^ Currently, CRC management primarily relies on surgery and chemotherapy,^3^ an aggressive form of chemical drug therapy that destroys rapidly growing cells by damaging DNA.^4,5^ Unfortunately, chemotherapy efficacy is limited by the development of chemoresistance mechanisms. Recently, it has been shown that CRC chemoresistance, recurrence, and metastasis depend on a small population of cells with low proliferative potential, called CRC stem cells (CRC-SCs), which can renew indefinitely, escaping apoptotic cell death signals, and may cause cancer recurrence even years after treatment.^6^ Moreover, following primary tumor growth, CRC-SCs are implicated in recurrence and metastatic dissemination through dormancy, re-initiation, and escape from immune surveillance mechanisms.^7^ This process is mediated by Wnt signaling, which can re-initiate cell cycle progression in dormant cancer stem cells (CSCs) by up-regulating c-MYC expression.^8^ Consistently, stable c-MYC knock-down decreases colony formation in primary and secondary organs.^9^ Besides, it has been shown that c-MYC is necessary for the self-renewal, migratory, and invasive properties of CSCs since its ablation reduces both the formation and the migration and invasion of tumorspheres.^10^ Intriguingly, c-MYC has been found to mediate DNA repair in CRC-SCs exposed to chemotherapeutic agents, thereby sustaining drug resistance.^11^

We recently showed that the methyltransferase SMYD3 has a major role in DNA repair in cancer cells.^12^ In particular, SMYD3 is part of a multiprotein complex, which includes ATM, BRCA2, and CHK2, required for the final loading of RAD51 at DNA double-strand breaks and the completion of homologous recombination.^5,13^ As a result, SMYD3-inhibited cancer cells fail to repair damaged DNA, becoming vulnerable to therapies.^12^ Moreover, SMYD3 promotes oncogenesis through different mechanisms: it can exert its enzymatic activity both on histone and non-histone proteins and can affect transcriptional-related functions by modifying chromatin accessibility and promoting gene expression.^5,14^

Although SMYD3 functions in CSCs have not been thoroughly characterized yet, preliminary studies in gastric CSCs indicated that SMYD3 enhances the transcription of ASCL2, a key regulator of stemness associated with the Wnt signaling pathway.^15^ Remarkably, SMYD3 inhibition reduced gastric cancer tumorsphere growth and the number of sphere-initiating cells.^15^ Moreover, SMYD3 promotes the epithelial-mesenchymal transition (EMT) in breast cancer cell lines, which leads to the acquisition of migration and self-renewal abilities, fostering the formation of secondary tumors at distant sites.^16^ Indeed, SMYD3 has been found necessary for SMAD3 direct association with EMT gene regulatory regions, suggesting that it acts as a SMAD3 cofactor promoting TGFβ-dependent mesenchymal gene expression.^16^ Additionally, in previous reports, we showed that SMYD3 interacts with MET, a well-known factor involved in stemness, EMT, chemoresistance, and metastasis in gastric cancer cells,^17^ and that higher SMYD3 levels, which were detected in almost one-third of the analyzed CRC patient cohort, were correlated with reduced overall survival and worse prognosis.^12^

Although these studies provided preliminary clues pointing to a major role for SMYD3 in CSC biology, further investigation is needed to clarify its involvement in tumor formation and metastatic dissemination. Indeed, understanding the molecular basis of SMYD3 function in cancer settings may help develop effective therapeutic strategies. Since finding biological inhibitors of c-MYC has proven difficult because of its nuclear localization, and recent evidence suggests that epigenetic control of the Wnt pathway is needed for the regulation of CSC self-renewal,^18^ here we explored the role of SMYD3 in CRC-SC biology and its involvement in c-MYC activity. To gain insight into the role of SMYD3 in CRC, we carried out an in-depth cellular and molecular investigation across multiple CRC models, including established cell lines, tumorspheres, patient-derived CRC-SCs, xenografts, and metastatic mouse models. Our data indicate that SMYD3 interacts with and methylates c-MYC, a central player in the Wnt signaling cascade. In particular, we found that SMYD3 is located on chromatin, and its pharmacological modulation influences key cancer-related processes. Moreover, we showed that targeting SMYD3 may inhibit uncontrolled proliferation and prevent c-MYC-dependent metastatic dissemination in vivo, making it a promising therapeutic target.

## Results

### Analysis of SMYD3 involvement in cancer stemness and cancer features in CRC models

Understanding the key regulators involved in the complex network of signaling pathways that drive cancer progression and resistance is crucial for designing novel strategies to enhance cancer cell responsiveness to conventional treatments. In this light, based on a library of rare tripeptides (termed P1-P19) tested for their in vitro binding affinity to SMYD3, we recently performed an in silico screening of the human proteome using each tripeptide as an in silico probe, leading to the identification of a total of 8,650 proteins (termed P-proteins) encompassing at least one P-tripeptide.^12,19^

Here, to deepen our knowledge of the function of SMYD3 in CRC stemness-related processes, we identified and subsequently clustered 64 P-proteins involved in developmental and stem cell biology pathways, based on the corresponding UniProt entry and current literature (Supplementary Fig. 1a, Supplementary Table 1). Interestingly, this cluster includes 7 (c-MYC, SOX2, SOX15, STAT3, GDF3, FTHL17, and FBXO15) of the 24 genes initially identified by Takahashi and Yamanaka as candidate factors inducing pluripotency in somatic cells.^20^ Our functional clustering analysis of these 64 P-proteins was also based on the associated pathways described in the Reactome database (Supplementary Table 1). This allowed us to focus on major cancer effectors, and especially on c-MYC, which is a crucial player both in CSC features and in CRC tumorigenesis.^21^ In particular, evidence is mounting that impaired c-MYC function may affect CSC stemness maintenance, metastasis of CSC-associated tumors, and resistance to therapy.^22,23^

**Fig. 1:**
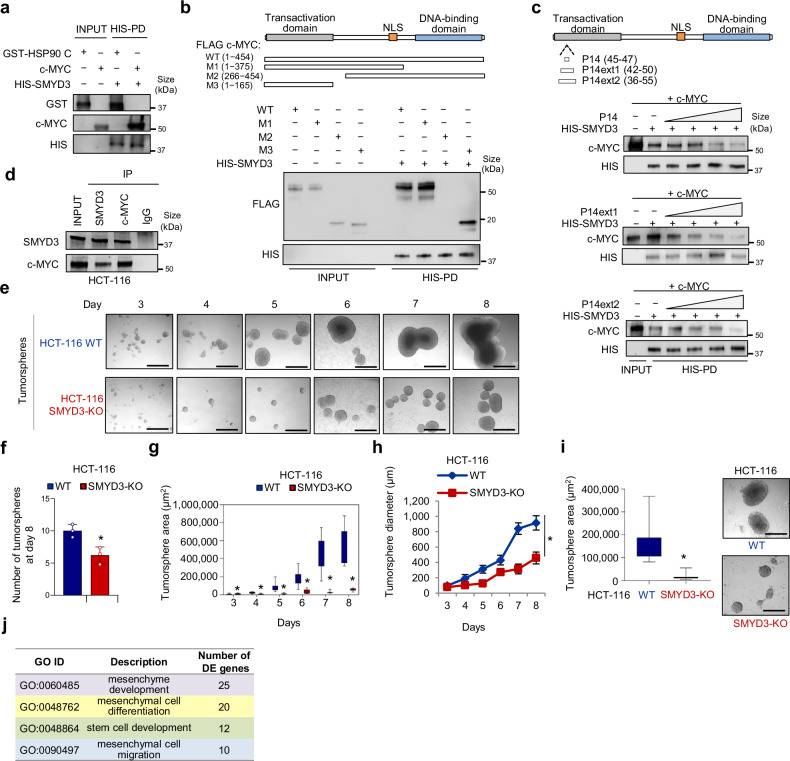
Analysis of SMYD3 involvement in cancer stemness and other cancer features in CRC models. **a** In vitro binding assay between HIS-SMYD3 and untagged recombinant c-MYC protein. GST-HSP90 C-terminal (616–736) was used as a positive control. **b** Upper panel: scheme showing the four FLAG c-MYC (WT + M1-M3) fusion proteins used in this study. Lower panel: in vitro binding assay between HIS-SMYD3 and recombinant FLAG c-MYC fragments. **c** Upper panel: Scheme showing the position of the purified P14, P14ext1, and P14ext2 peptides, which are located in the c-MYC transactivation domain (TAD). Lower panel: in vitro competition assay. HIS-SMYD3 bound to histidine beads was incubated with untagged c-MYC recombinant protein in the presence of escalating doses (0, 1, 5, 25, 125 µM) of the purified P14, P14ext1, and P14ext2 peptides. Bound proteins were visualized by immunoblot using anti-c-MYC and anti-HIS antibodies. **d** Co-immunoprecipitation assay of endogenous SMYD3 and c-MYC in HCT-116 CRC cells. **e**−**h** Tumorsphere formation assay of WT and SMYD3-KO HCT-116 cells: tumorsphere brightfield imaging (**e**, scale bar: 500 μm), number (**f**, day 8), area (**g**), and diameter (**h**) at day 3 to 8. **i** Area (left panel) and brightfield imaging (right panel, scale bar: 500 μm) of replated WT and SMYD3-KO HCT-116 tumorspheres. **j** Results of the Gene Ontology (GO) enrichment analysis of differentially expressed (DE) genes in SMYD3-KO vs WT HCT-116 cells. *p < 0.05 SMYD3-KO vs WT parental cells. HIS-PD= His-Pull down. Where applicable, data are expressed as means ± SD of 3 independent experiments

In order to validate our in silico screening, we performed an in vitro binding assay analyzing the interaction between a full-length HIS-tagged SMYD3 recombinant protein and a c-MYC recombinant protein. Interestingly, HIS pull-down assay results showed that c-MYC interacts with SMYD3 (Fig. 1a). GST-HSP90 C-terminal (616–736) was used as a positive control, based on a previous report by Brown and colleagues.^24^

Then, to resolve the interface of SMYD3/c-MYC interaction, we analyzed in vitro the binding between a full-length HIS-tagged SMYD3 recombinant protein and a series of four FLAG fusion proteins, designated WT, M1 to M3, which span the entire c-MYC coding region (Fig. 1b, upper panel). Our results showed that the M1 and M3 fragments interacted with SMYD3 (Fig. 1b, lower panel), indicating that c-MYC binds to SMYD3 through its N-terminal domain. Based on our in silico analysis, c-MYC contains one P-tripeptide (P14: NFY at aa position 45) located in the transactivation domain (TAD), which is crucial for c-MYC transcriptional activity. To validate the involvement of the P14 tripeptide in c-MYC interaction with SMYD3, we performed an in vitro competition assay between full-length HIS-tagged SMYD3 and full-length c-MYC with escalating doses of the purified P14 tripeptide (N_45_FY_47_) and two of its extensions, named P14 ext1 (E_42_EENFYQQQ_50_) and P14 ext2 (P_36_YFYCDEEENFYQQQQQSEL_55_) (Fig. 1c, upper panel). All of these peptides interfered with the binding between HIS-SMYD3 and full-length c-MYC in a dose-dependent manner (Fig. 1c, lower panel). These results validated the predictions from our proteomic screening by demonstrating a direct interaction between SMYD3 and c-MYC.

To determine whether this interaction also takes place in cellulo, we conducted co-immunoprecipitation assays of the endogenous proteins in HCT-116 CRC cells, which express high levels of SMYD3 and c-MYC and exhibit marked stemness properties, such as sphere- and soft agar colony-forming abilities, as well as increased expression of several CRC-SC markers.^25^ Our findings confirmed that SMYD3 physically interacts with c-MYC in this CRC cell model (Fig. 1d).

To investigate the role of SMYD3 in CSC biology, we took advantage of tumor-derived spheroids, or tumorspheres, a cellular model that is enriched for CSCs or cells with stem cell-related features.^26^ Specifically, HCT-116 CRC cells cultured as floating spheroids have been previously used as a surrogate system to evaluate the CRC-SC-related characteristics of solid tumors in vitro.^27^ We performed an immunoblot analysis to assess the expression of both SMYD3 and c-MYC in HCT-116 cells grown as a monolayer or in tumorsphere culture conditions and found that both proteins were enriched in the tumorsphere population. Tumorspheres also showed increased levels of CD44, a well-known CRC-SC marker^28^ (Supplementary Fig. 1b). Then, to assess whether SMYD3 was involved in tumorsphere-initiating mechanisms, we generated SMYD3-knock-out (KO) HCT-116 cells by employing CRISPR/Cas9-mediated genome editing (Supplementary Fig. 1c) and evaluated their ability to grow as tumorspheres. Our results showed that wild-type (WT) HCT-116 cells produced a higher number of tumorspheres in vitro, which also displayed a larger size (calculated by mean area and diameter) compared to their SMYD3-KO counterpart, suggesting that SMYD3 ablation impaired their tumorsphere-forming ability (Fig. 1e–h, Supplementary Fig. 1d). These results were further confirmed with replated second-passage tumorspheres (Fig. 1i).

Then, we evaluated the expression of the CRC stemness markers CD44, CD133, and EpCAM^29^ in WT and SMYD3-KO HCT-116 tumorspheres by flow cytometry. Our results revealed decreased levels of these CRC-SC-markers in SMYD3-KO HCT-116 tumorspheres compared to their WT counterpart (Supplementary Fig. 1e).

In addition, we performed BrdU-labeling assays to analyze symmetric/asymmetric division patterns in WT and SMYD3-KO HCT-116 tumorspheres. On day 1, both WT and SMYD3-KO tumorspheres within gate R3 maintained most of the BrdU incorporation, thus representing cells undergoing symmetric cell division (Supplementary Fig. 1f). After BrdU withdrawal, on day 3 we noticed an enrichment of tumorspheres within gate R4 that lost BrdU labeling, indicating that these cells started to undergo asymmetric (or lowly symmetric) cell division (Supplementary Fig. 1f). On day 5, WT HCT-116 tumorspheres showed an increase in the asymmetric population compared to their SMYD3-KO counterpart (Supplementary Fig. 1f), suggesting the involvement of SMYD3 in promoting asymmetric division, a feature that is correlated with cellular stemness level.^30^

Overall, these data suggest that SMYD3 expression is linked to the CSC phenotype in CRC cells. To corroborate this association, we performed an RNA-sequencing (RNA-seq) analysis comparing WT and SMYD3-KO HCT-116 cells. This differential expression comparison detected 767 significant differentially expressed (DE) genes, of which 558 were down-regulated and 209 were up-regulated in the absence of SMYD3. We used these deregulated genes to perform a Gene Ontology (GO) enrichment analysis, which indicated that they are primarily involved in biological processes related to stem cell biology, including mesenchyme development, mesenchymal cell differentiation, stem cell development, and mesenchymal cell migration (Fig. 1j, Supplementary Fig. 1g). These findings further support that SMYD3 plays a role in these processes.

**Fig. 2:**
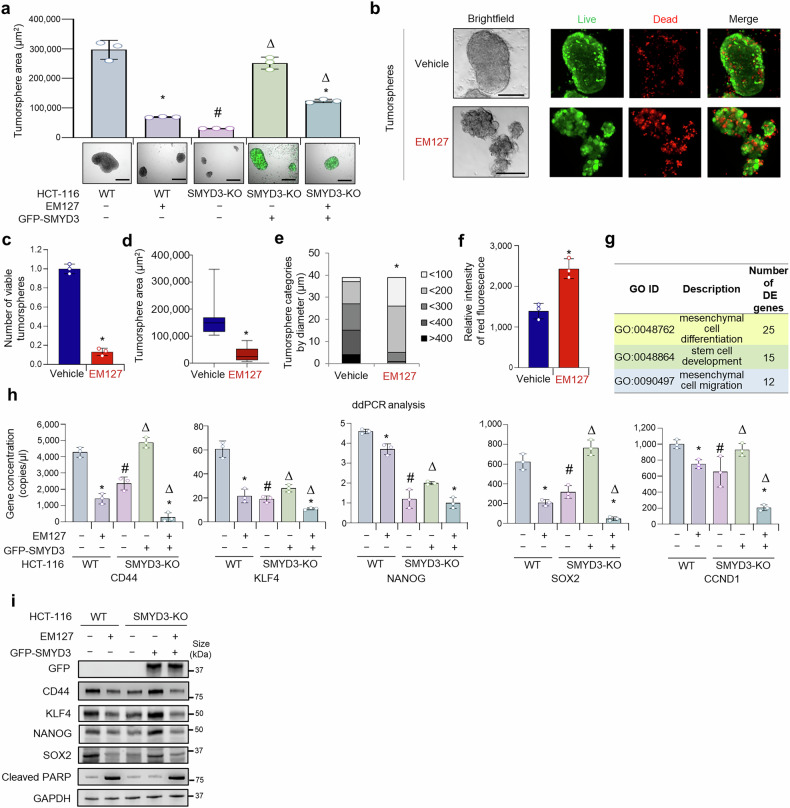
Effect of SMYD3 pharmacological inhibition on CRC stemness. **a** Tumorsphere formation assay of WT and SMYD3-KO HCT-116 tumorspheres treated or not with EM127 (5 μM) for 72 h. Where indicated, SMYD3 expression was reconstituted in SMYD3-KO tumorspheres with a GFP-SMYD3 construct. Scale bar: 500 μm. **b**−**f** Tumorsphere formation assay of WT HCT-116 tumorspheres treated or not with EM127 (5 μM) for 72 h. Tumorsphere brightfield imaging and live and dead staining (green: live cells; red: dead cells) (**b**, scale bar: 200 μm), number (**c**), area (**d**), diameter (**e**), and relative intensity of red fluorescence (**f**). **g** Results of the Gene Ontology (GO) enrichment analysis of differentially expressed (DE) genes in EM127-treated (5 μM for 24 h) vs untreated WT HCT-116 cells. **h** ddPCR analysis of stemness-related c-MYC target genes in WT and SMYD3-KO HCT-116 tumorspheres treated or not with EM127 (5 μM) for 24 h. Where indicated, SMYD3 expression was reconstituted in SMYD3-KO tumorspheres with a GFP-SMYD3 construct. **i** Immunoblot analysis of stemness-related c-MYC target gene products and Cleaved PARP in WT and SMYD3-KO HCT-116 tumorspheres treated or not with EM127 (5 μM) for 48 h. Where indicated, SMYD3 expression was reconstituted in SMYD3-KO tumorspheres with a GFP-SMYD3 construct. GAPDH was used as a loading control. *p < 0.05 EM127-treated vs untreated. #*p* < 0.05 SMYD3-KO vs WT parental tumorspheres. Δ*p* < 0.05 SMYD3-KO tumorspheres transfected with the GFP-SMYD3 construct vs untransfected SMYD3-KO tumorspheres. Where applicable, data are expressed as means ± SD of 3 independent experiments

To evaluate the potential therapeutic efficacy of SMYD3 genetic ablation, we assessed the cellular and molecular effects of its pharmacological inhibition using the SMYD3 inhibitor EM127. EM127 is a site-specific, covalent SMYD3 ligand leading to strong and sustained inhibition of its enzymatic methyltransferase activity.^31^ WT HCT-116 tumorspheres treated with EM127 showed reduced tumorsphere-forming ability, similar to SMYD3-KO tumorspheres. Of note, reconstitution of SMYD3 expression with a GFP-SMYD3 construct in SMYD3-KO tumorspheres was associated with an increase in their tumorsphere-forming ability (Fig. 2a, Supplementary Fig. 2a). However, this effect was reversed by treatment with EM127 (Fig. 2a). Similar results were observed in a different cancer model, i.e., MDA-MB-231 cells grown as tumorspheres (Supplementary Fig. 2b, c).

Additionally, live and dead staining revealed that SMYD3 pharmacological inhibition has a cytotoxic effect in WT HCT-116 tumorspheres (Fig. 2b). Indeed, tumorspheres treated with EM127 showed reduced number and size of viable tumorspheres (Fig. 2c–e) and increased intensity of the dead cell marker BOBO-3 iodide (red signal) (Fig. 2f). Moreover, our findings revealed that EM127 treatment affected the expression of CRC-SC markers, similar to what was observed when knocking out SMYD3 (Supplementary Fig. 2d)

To ascertain whether gene clusters related to stem cell biology were also modulated upon SMYD3 inhibition with EM127, as observed with SMYD3-KO, we performed an RNA-seq analysis comparing untreated and EM127-treated WT HCT-116 cells. In this analysis, we identified 786 significant DE genes, 668 of which were down-regulated while 118 were up-regulated. Again, a GO enrichment analysis encompassing all these deregulated genes revealed that they are primarily involved in processes such as mesenchymal cell differentiation, stem cell development, and mesenchymal cell migration (Fig. 2g; Supplementary Fig. 2e). A total of 42 genes showing GO enrichment in stem cell development, mesenchymal cell migration, mesenchymal cell differentiation, and mesenchyme development were identified to be deregulated in SMYD3-KO and/or EM127-treated WT HCT-116 cells (Supplementary Fig. 2f).

To corroborate the biological relevance of these 42 genes, we cross-matched them with six gene sets that were associated with CRC stemness in previous studies.^32–35^ These CRC stemness-related gene sets comprise a total of 2,141 unique genes (Supplementary Table 2), which include 20 (BCL2, EPHA4, FGF9, FOLR1, IL17RD, MEF2C, NKX2-1, NOG, NRP1, NRP2, PDCD4, PRICKLE1, RET, SEMA3A, SEMA3D, SEMA3F, SEMA4G, SEMA6A, TGFB1, TWIST1) of the 42 (47%) genes identified in our GO enrichment analysis, corroborating their potential role in CRC stemness (Supplementary Table 3). Among these 20 deregulated genes, five (FOLR1, NRP2, SEMA3A, SEMA3F, NKX2-1) were found to be deregulated in both SMYD3-KO and EM127-treated WT HCT-116 cells. Of note, we validated these results for some of these genes by performing a droplet digital PCR (ddPCR) analysis in SMYD3-KO HCT-116 cells or EM127-treated WT HCT-116 cells compared to untreated WT HCT-116 cells (Supplementary Fig. 2g).

These findings confirmed that SMYD3 is involved in the regulation of multiple CRC stemness features. Based on our data showing a direct interaction between SMYD3 and c-MYC, and considering that c-MYC exerts its oncogenic role by promoting cancer-related gene expression, we performed a ddPCR assay to investigate c-MYC stemness-related transcriptional activity upon loss of SMYD3 function, either genetically determined or pharmacologically induced. This analysis revealed that both SMYD3-KO and EM127 treatment resulted in the downregulation of c-MYC target genes, including CD44, KLF4, NANOG, SOX2, CCND1 (CSC proliferation markers) in HCT-116 tumorspheres (Fig. 2h). Importantly, reconstitution of SMYD3 expression with a SMYD3-GFP construct in SMYD3-KO tumorspheres resulted in higher transcription of these genes, while concomitant treatment with EM127 led to decreased expression levels (Fig. 2h). These findings were further validated at the protein level (Fig. 2i).

### Role of SMYD3 activity on c-MYC in patient-derived CRC-SCs

To improve the translational meaning of our work, we collected normal and pathological tissues from CRC patients who underwent surgical procedures at our hospital. In culture, we propagated cells obtained from these tissues both as patient-derived stem cells (normal [SCs] and tumoral [CRC-SCs]) grown as tumorspheres and as patient-derived organoids (PDOs) (normal [PDNOs] and tumoral [PDTOs]) (Supplementary Fig. 3a). Then, we performed a ddPCR analysis to identify patients with tumors overexpressing SMYD3 (Supplementary Fig. 3b).

Next, we characterized the effects of EM127 treatment in patient-derived CRC-SCs from patients with high-grade tumors overexpressing SMYD3. Our results revealed that SMYD3 pharmacological inhibition decreased the expression of the CSC markers CD44, EpCAM, and CD133 (Supplementary Fig. 4a). Since high aldehyde dehydrogenase (ALDH) enzymatic activity is considered an important CRC-SC hallmark,^36^ we assessed the impact of SMYD3 pharmacological inhibition on this feature. Of note, EM127 reduced the ALDH-positive population in patient-derived CRC-SCs (Supplementary Fig. 4b). Moreover, we carried out an in vitro limiting-dilution assay to ascertain whether SMYD3 pharmacological inhibition could impair the self-renewal activity of patient-derived CRC-SCs and found that treatment with EM127 reduced their sphere-initiating cell frequency (Supplementary Fig. 4c). EM127-treated patient-derived CRC-SCs also showed a decrease in tumorsphere size compared to their untreated counterpart (Supplementary Fig. 4d).

To further assess stemness features in our patient-derived model, we immunostained PDTOs overexpressing SMYD3. This immunofluorescence analysis showed that SMYD3 was co-expressed with CD44, a well-known CRC-SC marker^28^ (Supplementary Fig. 4e).

**Fig. 3:**
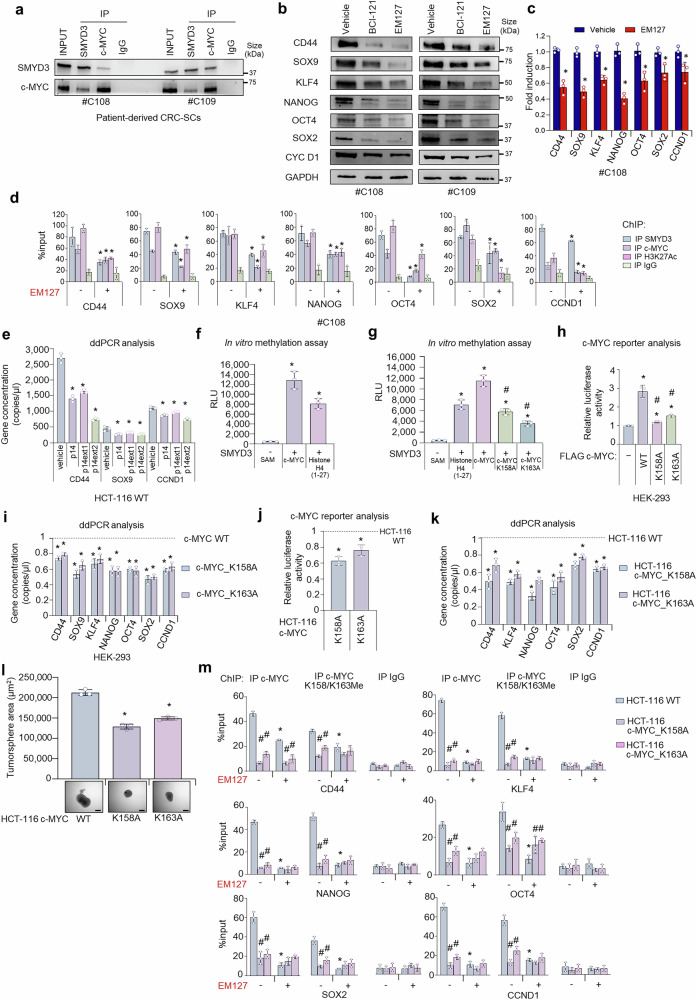
Molecular and functional characterization of SMYD3-dependent c-MYC methylation in CRC stemness. **a** Co-immunoprecipitation assay of endogenous SMYD3 and c-MYC in patient-derived CRC-SCs. **b** Immunoblot analysis of c-MYC stemness-related target gene products in patient-derived CRC-SCs treated or not with BCI-121 (100 μM) or EM127 (10 μM) for 48 h. **c** Real-time PCR analysis of c-MYC target genes in patient-derived CRC-SCs treated or not with EM127 (10 μM) for 24 h. **d** Chromatin immunoprecipitation (ChIP) assay of patient-derived CRC-SCs treated or not with EM127 (10 μM) for 24 h. Chromatin was pulled down with anti-SMYD3, anti-c-MYC, or anti-H3K27Ac antibodies, as indicated. Quantification was done using the % input method. *p < 0.05 EM127-treated vs untreated. **e** ddPCR analysis of c-MYC stemness-related target genes in WT HCT-116 cells cultured in the presence of P14 (125 μM), P14ext1 (125 μM), or P14ext2 (50 μM) for 24 h. **f** In vitro methylation assay showing c-MYC methylation by SMYD3. Histone H4 (1-27) was used as a SMYD3 control substrate. *p < 0.05 vs SAM. **g** In vitro methylation assay of mutagenized c-MYC. Histone H4 (1-27) was used as a SMYD3 control substrate. *p < 0.05 vs SAM; #*p* < 0.05 vs WT c-MYC. **h** Bar plot of the relative c-MYC luciferase reporter activity in HEK-293 cells transiently transfected with WT c-MYC, mutant c-MYC_K158A, or mutant c-MYC_K163A constructs. *p < 0.05 vs empty vector; #*p* < 0.05 vs WT c-MYC. **i** ddPCR analysis of c-MYC stemness-related target genes in HEK-293 cells transiently transfected with mutant c-MYC_K158A or c-MYC_K163A vs WT c-MYC constructs. *p < 0.05 vs WT c-MYC. **j** Bar plot of the relative c-MYC luciferase reporter activity in c-MYC_K158A or c-MYC_K163A knock-in vs WT HCT-116 cells. *p < 0.05 vs WT HCT-116 cells. **k** ddPCR analysis of c-MYC target genes in c-MYC_K158A or c-MYC_K163A knock-in vs WT HCT-116 cells. *p < 0.05 vs WT HCT-116 cells. **l** Tumorsphere formation assay of WT, c-MYC_K158A, and c-MYC_K163A HCT-116 cells. *p < 0.05 vs WT HCT-116 cells. Scale bar: 200 μm. **m** ChIP assay of WT, c-MYC_K158A, and c-MYC_K163A HCT-116 cells treated or not with EM127 (5 μM) for 24 h. Chromatin was pulled down with anti-c-MYC or anti-c-MYC_K158/K163Me antibodies. Quantification was done using the % input method. *p < 0.05 EM127-treated vs untreated, #*p* < 0.05 mutant c-MYC knock-in cells vs WT parental cells. **a**, **d**, **m** Anti-IgGs were used as negative controls. SAM = S-Adenosine-Methionine. Where applicable, data are expressed as means ± SD of 3 independent experiments

Subsequently, we performed immunoprecipitation assays of whole-cell lysates from patient-derived CRC-SCs and confirmed that SMYD3 co-immunoprecipitates with c-MYC in these models (Fig. 3a). In order to characterize the role of SMYD3 in the expression of c-MYC-related CRC-SC markers, we treated patient-derived CRC-SCs with two different SMYD3 pharmacological inhibitors, the previously characterized substrate-competitive inhibitor BCI-121^26^ and the covalent inhibitor EM127. Immunoblot analysis revealed a marked reduction in several c-MYC target gene products involved in CSC biology after treatment with SMYD3 pharmacological inhibitors (Fig. 3b).

Then, we investigated the changes promoted by EM127 in c-MYC-dependent transcription. Our results showed that treatment of patient-derived CRC-SCs with EM127 triggers the downregulation of c-MYC target genes, including CD44, SOX9, KLF4, NANOG, OCT4, SOX2, CCND1 (CSC markers) (Fig. 3c).

These data prompted us to investigate the functional role of SMYD3/c-MYC complexes in transcriptional regulation. To this end, we evaluated the recruitment of SMYD3 to the promoter regions of c-MYC target genes by chromatin immunoprecipitation (ChIP) in patient-derived CRC-SCs. ChIP assays revealed that SMYD3 was recruited with c-MYC to Wnt responsive elements (WREs) of several c-MYC targets, including CD44, SOX9, KLF4, NANOG, OCT4, SOX2, and CCND1, which are all involved in CRC stemness features and tumor progression^37^ (Fig. 3d). Further characterization of these regions showed acetylation of lysine 27 (H3K2Ac) on histone H3, an epigenetic modification typical of active enhancers (Fig. 3d). Moreover, we found that EM127 treatment reduced SMYD3 and c-MYC occupancy at the analyzed WREs (Fig. 3d). This evidence implies that SMYD3 acts as a co-regulator of c-MYC, promoting the transcription of its target genes in these cells.

The reduced chromatin-binding ability of SMYD3/c-MYC complexes upon EM127 treatment prompted us to evaluate whether SMYD3 activity was required for its physical interaction with c-MYC in patient-derived CRC-SCs. Co-immunoprecipitation assays showed that SMYD3 pharmacological inhibition with the covalent inhibitor EM127 did not prevent complex formation (Supplementary Fig. 4f).

### Molecular and functional characterization of SMYD3-dependent c-MYC methylation in colorectal cancer stemness

Next, we investigated whether impairment of SMYD3/c-MYC interaction affects c-MYC transcriptional activity. To this end, we treated HCT-116 cells with the purified P14 tripeptide (N_45_ FY_47_) and two P14 extensions (P14 ext1 and P14 ext2), and then we performed a ddPCR analysis to investigate c-MYC target gene expression. Our results showed that impairing the binding between SMYD3 and c-MYC reduced c-MYC transcriptional activity (Fig. 3e).

Given that c-MYC transcriptional activity is modulated by established post-translational modifications at both its N-terminal and C-terminal regions, we carried out an in vitro methylation assay using purified protein samples. This analysis showed that active SMYD3 can efficiently methylate c-MYC (Fig. 3f). Then, to identify c-MYC methylation sites, we performed a mass spectrometry analysis and found that SMYD3 methylates c-MYC on lysine 158 (K158) and lysine 163 (K163) (Supplementary Fig. 5a). Intriguingly, both these lysines are included in the N-terminal transactivation domain (NTD) of mammalian c-MYC proteins.^38^ Of note, the NTD region comprises the TAD, which is primarily responsible for c-MYC transactivation and transrepression.^39^ Next, we assessed the functional and evolutionary significance of these lysine residues by performing a multiple alignment analysis of c-MYC homologous proteins across a variety of species, ranging from Asterias rubens to humans. Consistent with the evidence that post-translational modifications preferentially occur in evolutionarily conserved regions, human c-MYC K158 and K163 are highly conserved amino acids across different species (Supplementary Fig. 5b). Moreover, to better characterize the exposure of these residues in c-MYC structure, we carried out an in silico analysis to estimate the relative surface accessibility (RSA) of K158 and K163. Interestingly, according to our in silico results, both lysines (RSA = 59% for K158; RSA = 54% for K163; threshold at 25%) are predicted to be exposed in c-MYC tridimensional structure (Supplementary Fig. 5c).

To consolidate these data, we performed in vitro methylation assays on site-specific mutants generated by replacing these lysines with alanine residues, which cannot be methylated (c-MYC_K158A and c-MYC_K163A). Our data demonstrated that, under these conditions, SMYD3 exhibits significantly lower catalytic activity in methylating c-MYC, further confirming its role in this process (Fig. 3g).

To investigate whether methylation of these two lysines modulates c-MYC transcriptional activity in cellulo, we performed c-MYC luciferase reporter assays using a reporter that contains the firefly luciferase gene under the control of multimerized MYC-responsive elements located upstream of a minimal promoter. We transfected HEK-293 cells with plasmids encoding for WT c-MYC or site-specific mutants. Importantly, luciferase activity was reduced in cells overexpressing the c-MYC K158A or K163A mutants compared to cells overexpressing WT c-MYC (Fig. 3h, Supplementary Fig. 5d), suggesting that SMYD3-mediated methylation of both lysines is essential for c-MYC transcriptional activity. To confirm these data, we performed a ddPCR analysis to evaluate CRC-SC-related markers whose expression depends on c-MYC transcriptional activity. Our results showed decreased expression of these markers in HEK-293 cells transfected with the c-MYC_K158A or K163A plasmids (Fig. 3i).

Subsequently, to get further insights into the role and biological significance of c-MYC K158 and K163 methylation, we generated c-MYC_K158A and c-MYC_K163A knock-in (KI) HCT-116 CRC cell lines by using the CRISPR/Cas9 system for genome editing (Supplementary Fig. 5e). To investigate c-MYC transcriptional activity in these cells, we performed a c-MYC luciferase reporter assay (Fig. 3j). Interestingly, luciferase activity was reduced in c-MYC_K158A and c-MYC_K163A KI HCT-116 cells compared to c-MYC_WT HCT-116 cells (Fig. 3j). These data were further corroborated by ddPCR analysis, showing that c-MYC target gene expression was reduced in c-MYC_K158A and c-MYC_K163A KI HCT-116 cells compared to HCT-116 cells expressing WT c-MYC (Fig. 3k). Then, to assess whether SMYD3-dependent c-MYC methylation was involved in tumorsphere-initiating mechanisms, we evaluated the ability of c-MYC_K158A and c-MYC_K163A KI HCT-116 cells to grow as tumorspheres. Our results showed that c-MYC_WT HCT-116 tumorspheres displayed a larger size compared to their c-MYC-KI counterpart, suggesting that SMYD3-dependent c-MYC methylation promoted their tumorsphere-forming ability (Fig. 3l). Furthermore, we performed various assays to investigate the role of c-MYC methylation by SMYD3 in CRC-SC properties. We first evaluated CRC-SC migratory and invasion ability and found that c-MYC_K158A and c-MYC_K163A KI HCT-116 cells showed decreased migration and invasiveness compared to their WT counterpart (Supplementary Fig. 5f, g). Subsequently, we carried out an in vitro limiting-dilution assay to ascertain whether the impairment of SMYD3-mediated c-MYC methylation could impair the self-renewal activity of these cells. Our results revealed that c-MYC_K158A and c-MYC_K163A KI HCT-116 tumorspheres showed reduced sphere-initiating cell frequencies (Supplementary Fig. 5h).

To further explore these mechanisms, we developed an antibody specific for the c-MYC protein methylated at K158 and K163 (c-MYC K158/K163Me). The affinity-purified c-MYC K158/K163Me antibody showed high specificity towards the immunizing dimethyl K158/163Me peptide compared to control peptide in immuno dot-blot analysis and immunizing peptide blocking experiments (Supplementary Fig. 6a, b).

Importantly, the presence of a functional SMYD3 protein was associated with high c-MYC methylation levels in cells overexpressing WT c-MYC but had a minimal effect in cells overexpressing the K158A or K163A mutant proteins, indicating a reduced methylation signal in the latter (Supplementary Fig. 6c).

To validate the decrease of c-MYC K158/K163Me levels in c-MYC_K158A and c-MYC_K163A KI HCT-116 cells, we performed an immunofluorescence analysis with our specific anti-c-MYC K158/K163Me antibody. Interestingly, we observed a reduction in c-MYC methylation levels both in WT HCT-116 cells treated with EM127 and in KI cells (c-MYC_K158A and c-MYC_K163A HCT-116 cells). No change in methylated c-MYC levels was noticed in EM127-treated KI cells compared to their untreated counterpart (Supplementary Fig. 6d). Of note, ChIP assays revealed that the recruitment of c-MYC on WREs was reduced in c-MYC_K158A and c-MYC_K163A KI HCT-116 cells compared to HCT-116 cells expressing WT c-MYC (Fig. 3m). Moreover, SMYD3 pharmacological inhibition was not associated with decreased c-MYC recruitment on WREs in c-MYC_K158A and c-MYC_K163A KI HCT-116 cells (Fig. 3m). Overall, these data support the important role of SMYD3-dependent c-MYC methylation in cancer stemness.

### The SMYD3/c-MYC axis is involved in cancer stemness and other cancer features in patient-derived CRC-SCs

**Fig. 4:**
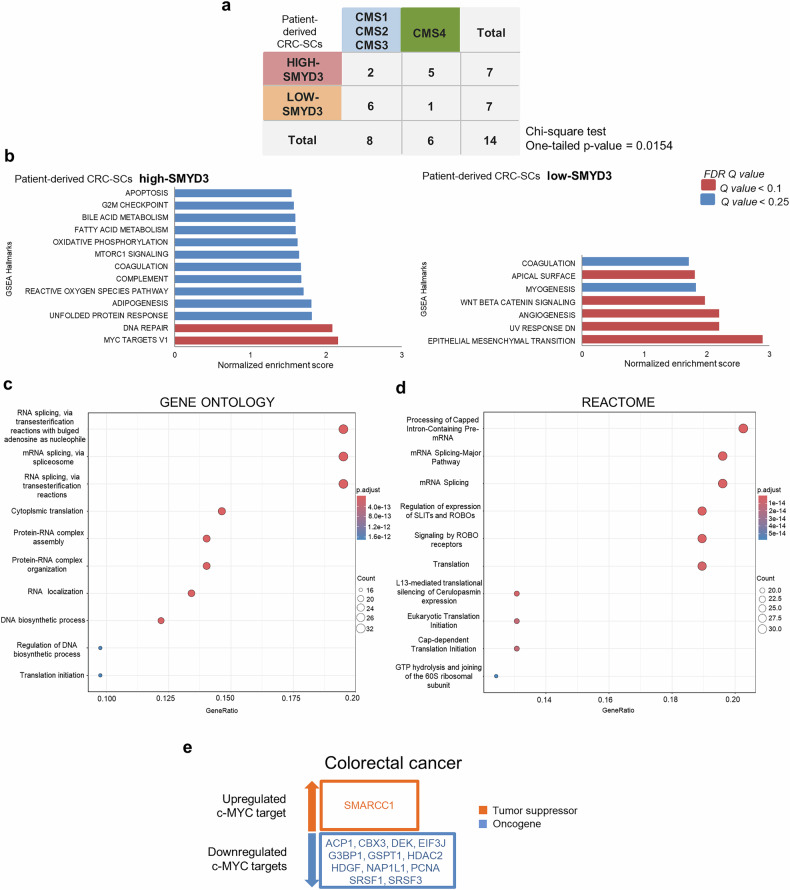
Consensus molecular subtype (CMS) classification and gene set enrichment analysis (GSEA) hallmark pathways in patient-derived CRC-SCs. **a** Distribution of CMS groups in patient-derived CRC-SCs overexpressing SMYD3 (high-SMYD3) (*n* = 7) and not overexpressing SMYD3 (low-SMYD3) (*n* = 7). **b** GSEA results of the hallmark pathway in EM127-treated (5 μM for 24 h) vs untreated patient-derived CRC-SCs. The graphs represent the main hallmarks (y-axis) identified as significantly enriched in the high-SMYD3 and low-SMYD3 groups. The false discovery rate (FDR) Q value is also reported. **c**, **d** Dot plots of the top 20 ranked terms obtained from the Gene Ontology (GO) enrichment analysis (**c**) and the REACTOME analysis (**d**) of the MYC TARGETS V1 hallmark per the GSEA described in **b**. “Count” indicates the number of genes enriched in a GO term. “Gene ratio” indicates the percentage of enriched genes in the given GO term. **e** CancerMine (http://bionlp.bcgsc.ca/cancermine) classification of the c-MYC-downregulated and -upregulated targets identified as oncogenes (blue rectangles) or tumor suppressors (orange rectangle) in CRC

To get further insight into the molecular signature of patient-derived CRC-SCs, we performed an RNA-seq analysis. Functional clinical data for all patients are listed in Supplementary Table 4. We analyzed the gene expression patterns of CRC-SCs derived from 7 CRC patients with SMYD3 overexpression (high-SMYD3) and 7 CRC patients without SMYD3 overexpression (low-SMYD3). Interestingly, we found that 5 out of 7 SMYD3-overexpressing CRC patients belong to the consensus molecular subtype 4 (CMS4), which is considered the worst CMS because it is characterized by a mesenchymal phenotype with gene signatures consistent with an activated stroma.^40^ Moreover, CMS4 CRCs are often diagnosed at advanced stages and have a poor prognosis, with the worst 5-year overall survival (62%) and relapse-free survival (60%) of any molecular subtype.^40^ These data suggest that patients with SMYD3-overexpressing CRC have a more aggressive phenotype (Fig. 4a). Then, we took advantage of the Preranked Gene Set Enrichment Analysis (GSEA) computational method^41,42^ to identify specific hallmarks that were enriched after SMYD3 pharmacological inhibition. Interestingly, we found that the most enriched hallmark in high-SMYD3 patients after EM127 treatment was “MYC TARGETS V1”. In contrast, no enrichment was detected for this hallmark in low-SMYD3 patients (Fig. 4b). The “MYC TARGETS V1” hallmark comprises 168 genes, 57 of which have a negative score (log2FC < 0), while 111 have a positive score (log2FC > 0). In broad terms, the ranking score used by GSEA is a measure of the association of a gene to a particular phenotype. In our case, a positive score indicates a higher expression of the gene in EM127-treated CRC-SCs from patients with SMYD3-overexpressing cancers compared to untreated cells, while a negative score denotes a lower gene expression. Supplementary Tables 5 and 6 list the functional information reported in the UniProt database at the time of analysis (January 2024), along with the corresponding GO annotation, for each c-MYC target gene that obtained a negative (Supplementary Table 5) or a positive score (Supplementary Table 6) in GSEA. Among the 168 c-MYC target genes identified, 10 genes (17.5%) involved in stemness-related processes showed decreased expression, and 24 genes (21.6%) involved in stemness-related processes showed increased expression after treatment with EM127, as reported in Supplementary Tables 5 and 6, respectively.^43^

Importantly, our GO and REACTOME enrichment analysis revealed the involvement of the 168 c-MYC target genes identified by GSEA in four large functional clusters: i) mRNA splicing and processing; ii) translation pathways; iii) signaling by ROBO receptors; and iv) DNA biosynthetic processes (Fig. 4c, d). To explore the cancer-related role of these SMYD3-modulated c-MYC target genes, we determined whether they act as oncogenes or tumor suppressors in CRC based on CancerMine database annotations (http://bionlp.bcgsc.ca/cancermine). We found that among the 57 c-MYC target genes showing decreased expression after EM127 treatment, 12 (21%) are known to be oncogenes, whereas among the 111 c-MYC target genes showing increased expression after EM127 treatment, only 1 (1.11%) is known to be a tumor suppressor in CRC (Fig. 4e).

Some of the GSEA-identified c-MYC target genes have been shown to play important functions in CRC. For example, the splicing factor SRSF1 is involved in maintaining stemness in human CRC organoids,^44^ the translation initiation factor EIF3J plays a role in translational reprogramming processes during tumor onset and progression,^45,46^ and the Slit protein family has been implicated in CRC cell migration through signaling activation by ROBO receptors.^47,48^ Notably, ROBO-Slit complexes can act both as oncogenes and as tumor suppressors in CRC and other cancer types.^48–52^ Another example is PCNA, an important biomarker of DNA biosynthetic processes, which are known to play a pivotal function in CRC stemness (Fig. 4c–e).^53,54^

Collectively, these findings indicate that inhibiting SMYD3 leads to the suppression of a c-MYC-driven oncogenic gene expression program.

### SMYD3 as a potential therapeutic target in CRC-SCs

**Fig. 5:**
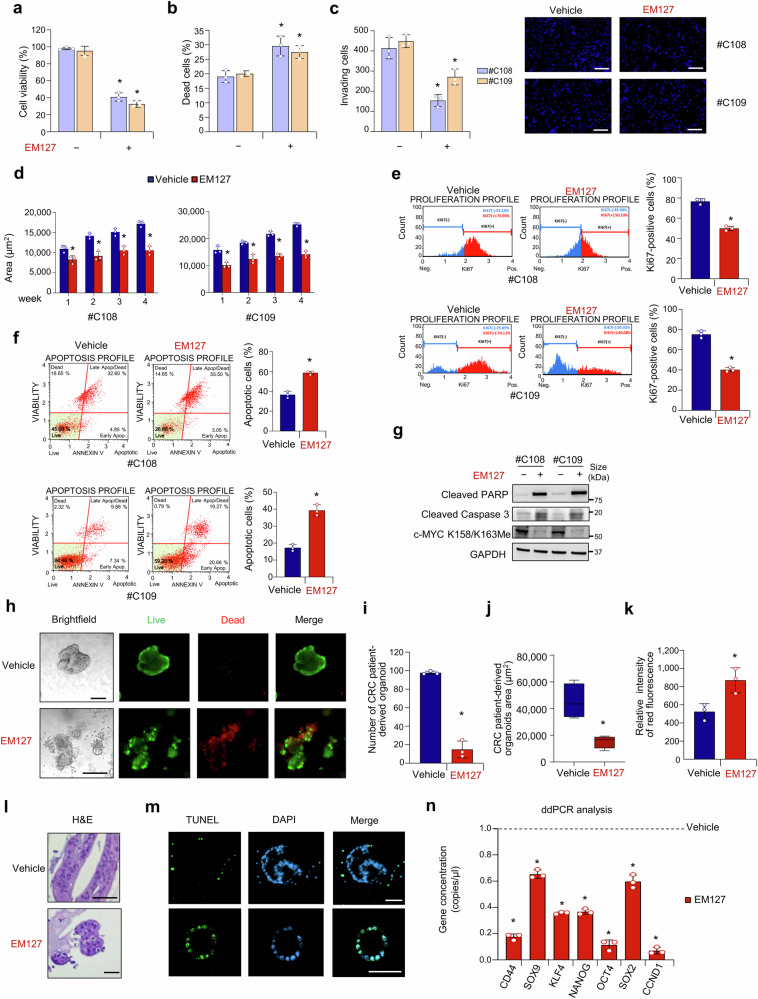
SMYD3 as a potential therapeutic target in CRC-SCs. **a** Quantification of cell viability by CellTiter-Glo in patient-derived CRC-SCs (#C108 and #C109) treated or not with EM127 (10 μM) for 72 h. **b** Quantification of cell death by Trypan blue staining in patient-derived CRC-SCs (#C108 and #C109) treated or not with EM127 (10 μM) for 72 h. **c** Invasive ability of growth factor-starved patient-derived CRC-SCs (#C108 and #C109) placed in the inner chamber of transwell plates and treated or not with EM127 (10 μM) for 16 h. Invading cells were fixed and counted under a fluorescence microscope. Scale bar: 200 μm. **d** Colony-forming ability of patient-derived CRC-SCs (#C108 and #C109) seeded onto double-layer soft agar and treated or not with EM127 (10 μM) for 72 h. Data represent the area of the evaluated colonies during four weeks. **e** Flow cytometry analysis (left panel) and corresponding bar plot representation (right panel) of Ki67 expression in patient-derived CRC-SCs (#C108 and #C109) treated or not with EM127 (10 μM) for 72 h. **f** Flow cytometry analysis (left panel) and corresponding bar plot representation (right panel) of annexin V staining in patient-derived CRC-SCs (#C108 and #C109) treated or not with EM127 (10 μM) for 72 h. The graph summarizes the percentage of apoptotic cells (early + late). **g** Immunoblot analysis of Cleaved PARP, Cleaved Caspase 3, and c-MYC K158/K163Me levels in patient-derived CRC-SCs (#C108 and #C109) treated or not with EM127 (10 μM) for 72 h. GAPDH was used as a loading control. **h**−**n** Characterization of PDTOs treated or not with EM127 (10 μM) for 72 h. PDTO brightfield imaging and live and dead staining (green: live cells; red: dead cells) (**h**, scale bar: 200 μm), number (**i**), area (**j**), relative intensity of red fluorescence (**k**), hematoxylin and eosin (H&E) staining (**l**, scale bar: 50 μm), TUNEL staining (green) with nuclei counterstained with DAPI (blue) (**m**, scale bar: 50 μm), and ddPCR analysis for stemness-related c-MYC target genes (**n**). *p < 0.05 EM127-treated vs untreated. Where applicable, data are expressed as means ± SD of 3 independent experiments

Next, we performed various assays to thoroughly evaluate the effects of SMYD3 pharmacological inhibition on important biological features of patient-derived CRC-SCs. Treatment with EM127 reduced CRC-SC viability and proliferation (Fig. 5a and Supplementary Fig. 7a) and increased cell death (Fig. 5b). Given that patient-derived CRC-SCs grow as tumorspheres, we also carried out a spheroid-based invasion assay to evaluate their invasive ability. Pharmacological inhibition of SMYD3 resulted in a significant reduction in CRC-SC invasiveness (Fig. 5c). Then, we conducted a long-term soft agar assay to assess the ability of these cells to form anchorage-independent tumor colonies and, more importantly, to sustain regrowth over time (Fig. 5d). To this end, we treated patient-derived CRC-SCs with EM127 for 72 h and then monitored their ability to grow for up to four weeks in the absence of the inhibitor. Of note, EM127-treated CRC-SCs only formed small clones with limited growth over time (Fig. 5d). These data suggest that SMYD3 pharmacological inhibition also affects CRC-SC regrowth ability. To further explore the biological effects of EM127 treatment on the fate of patient-derived CRC-SCs, we analyzed Ki67 expression and annexin V staining by flow cytometry (Fig. 5e, f). Ki67 is widely recognized as a cell proliferation marker; moreover, it plays a role in the maintenance of the stem cell niche and hence can also be used as a CSC marker.^55^ Based on our results, SMYD3 pharmacological inhibition reduced Ki67 staining in patient-derived CRC-SCs (Fig. 5e), and this effect was associated with the induction of apoptosis (Fig. 5f). Activation of the apoptotic pathway in CRC-SCs treated with EM127 was further validated by immunoblotting experiments for Cleaved Caspase 3 and PARP (Fig. 5g). Moreover, we validated also in this cellular model that SMYD3 pharmacological inhibition reduced c-MYC methylation at K158/K163 (Fig. 5g).

c-MYC is a master regulator of the Wnt signaling pathway, which is a key driver in the development of CRC.^56^ Thus, we assessed whether the effect of SMYD3 pharmacological inhibition could be reversed by Wnt3a-induced exogenous activation of the Wnt pathway. Our data showed a decrease in the expression of CSC markers in patient-derived CRC-SCs treated with EM127, both as pre-treatment and as co-treatment with Wnt3a (Supplementary Fig. 7b). Subsequently, we analyzed the morphological and molecular features of SMYD3-overexpressing PDTOs upon SMYD3 pharmacological inhibition. Live and dead staining revealed that treatment with EM127 had a cytotoxic effect on these organoids (Fig. 5h). These results were corroborated by the reduced number and size of viable PDTOs (Fig. 5i, j) and the increased intensity of the dead cell marker BOBO-3 iodide (red signal) after EM127 treatment (Fig. 5k). Moreover, hematoxylin and eosin staining revealed a massive presence of pyknotic nuclei in EM127-treated PDTOs (Fig. 5l). These data were supported by TUNEL staining (Fig. 5m). Finally, ddPCR assays showed that the expression of c-MYC target genes was decreased in PDTOs exposed to EM127 (Fig. 5n), confirming that SMYD3 plays an important role in cancer stemness features also in this complex cellular model.

### In vivo studies to tailor a novel therapeutic approach based on SMYD3 inhibition

**Fig. 6:**
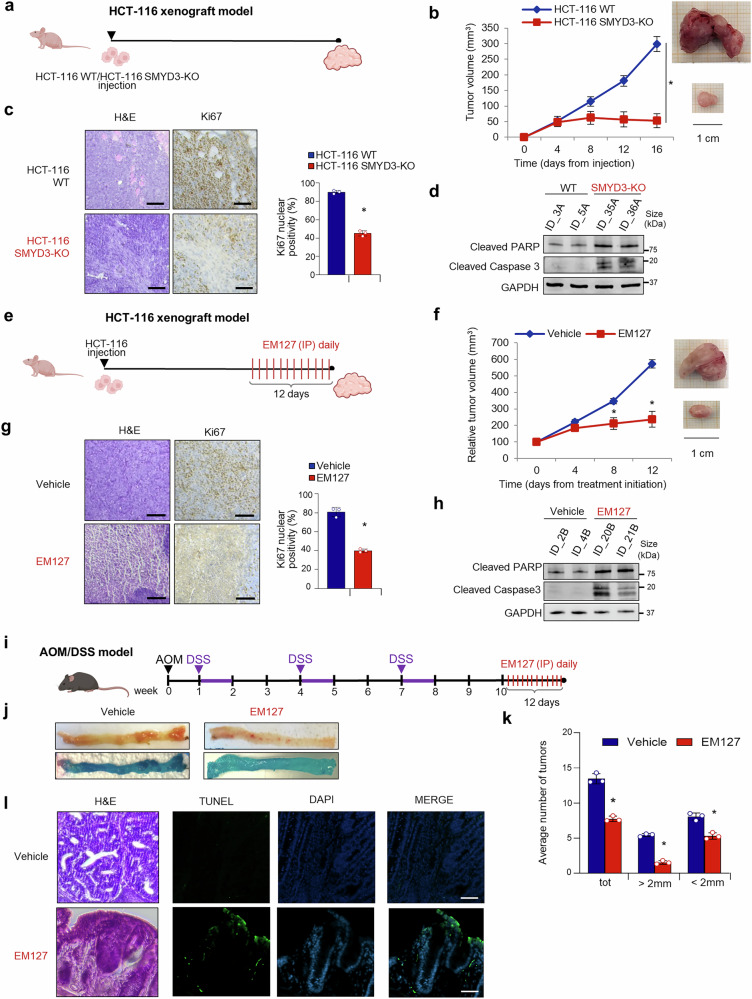
In vivo studies to tailor a novel therapeutic approach based on SMYD3 inhibition. **a** Schematic illustration of the establishment of WT and SMYD3-KO HCT-116 cell xenograft mice. Analysis of tumors explanted from mice treated as depicted in **a**. **b**−**d** Tumor volume over time (**b**), hematoxylin and eosin (H&E) and Ki67 immunohistochemical staining (left panel) and bar plot summarizing the mean percentage of Ki67-positive cells (right panel) (**c**, scale bar: 100 μm), and immunoblot analysis of Cleaved PARP and Cleaved Caspase 3 levels (GAPDH was used as a loading control) (**d**). **e** Schematic illustration of the establishment of WT HCT-116 cell xenograft mice and EM127 treatment. As soon as the tumors reached a measurable size, mice were treated with daily intraperitoneal injections of EM127 (10 mg/kg) or the vehicle alone for 12 days and then sacrificed. **f**−**h** Analysis of tumors explanted from mice treated as depicted in **e**. Tumor volume over time (**f**), hematoxylin and eosin (H&E) and Ki67 immunohistochemical staining (left panel, scale bar: 100 μm) and bar plot summarizing the mean percentage of Ki67-positive cells (right panel) (**g**), and immunoblot analysis of Cleaved PARP and Cleaved Caspase 3 levels (GAPDH was used as a loading control) (**h**). **i** Schematic illustration of the establishment of AOM/DSS mice and EM127 treatment. After three cycles of DSS, mice were treated with daily intraperitoneal injections of EM127 (10 mg/kg) or the vehicle alone for 12 days and then sacrificed. **j**−**l** Analysis of tumors explanted from mice treated as depicted in **i**. Examination of explanted tissues showing tumor formation (**j**), average tumor number (**k**), and hematoxylin and eosin (H&E) and TUNEL staining (green) with nuclei counterstained with DAPI (blue) of colon sections (**l**, scale bar: 50 μm). AOM azoxymethane, DSS dextran sodium sulfate, IP intraperitoneal. **b**, **c** *p < 0.05 SMYD3-KO vs WT parental cells; **f**, **g**, **k** *p < 0.05 treated vs untreated. Where applicable, data are expressed as means ± SD of 3 independent experiments

To investigate the impact of SMYD3 knock-out on cancer cell behavior in vivo, we monitored tumor growth in a mouse model. Xenograft tumors were established by injecting WT or SMYD3-deficient HCT-116 cells into athymic nude mice (Fig. 6a). After 16 days, tumor volume was significantly lower in mice xenografted with SMYD3-KO HCT-116 cells compared to WT HCT-116 cells (Fig. 6b), consistent with previous reports indicating that the absence of SMYD3 impairs tumor development.^57,58^ At the conclusion of the study, xenograft tumors were explanted and subjected to histological evaluation (Fig. 6c). The results of this experiment revealed marked tumor regression, which was primarily characterized by the presence of fibrotic tissue tending to restrain neoplastic invasion in SMYD3-KO tumors compared to their WT SMYD3 counterpart. Interestingly, SMYD3-KO xenograft tumors showed an average Ki67 positivity score of 45% compared to 90% in tumors with WT SMYD3 (Fig. 6c), along with increased apoptosis induction, as shown by the upregulation of Cleaved PARP and Cleaved Caspase 3 levels (Fig. 6d). Importantly, our molecular analysis revealed that SMYD3-KO xenograft tumors expressed reduced levels of various c-MYC-related CRC-SCs markers (Supplementary Fig. 8a, b).

After analyzing the effects of SMYD3 genetic ablation, we moved on to evaluate its pharmacological inhibition in the same in vivo model. Xenograft tumors were established by injecting WT HCT-116 cells into athymic nude mice. When tumors reached a measurable size, animals were separated into two groups and treated with the vehicle or with EM127. In all the in vivo studies involving EM127 carried out in this work, we took advantage of nanoformulation techniques to improve EM127 solubility and bioavailability. These methodologies have demonstrated considerable efficacy in enhancing the stability, as well as the pharmacokinetic and pharmacodynamic characteristics of a variety of bioactive compounds.^59^ In particular, human serum albumin (HSA), the predominant serum protein, has emerged as a well-established and biocompatible carrier. Indeed, its ability to prolong the systemic half-life of therapeutic agents and selectively accumulate in tumor tissues contributes to reduced off-target toxicity.^60^ We thus used EM127 encapsulated within HSA-based nanoparticles for the subsequent in vivo studies. Drug treatments were administered daily by intraperitoneal injection for 12 days (Fig. 6e), and tumor size and body weight were routinely measured every 2 or 3 days. At the conclusion of the treatment period, tumor volume was significantly lower in EM127-treated compared to vehicle-treated HCT-116-xenografted mice (Fig. 6f). Xenograft tumors were then explanted and subjected to histological examination (Fig. 6g). This analysis showed marked tumor regression in EM127-treated HCT-116-xenografted mice, which was associated with a marked reduction in the average Ki67 positivity score (40% vs 80% in vehicle-treated animals) (Fig. 6g) and increased levels of Cleaved PARP and Cleaved Caspase 3 (Fig. 6h). Of note, SMYD3 pharmacological inhibition reduced the expression levels of c-MYC-related CRC-SCs markers in these in vivo xenograft models (Supplementary Fig. 8c, d).

To further validate our findings, we carried out in vivo experiments in the orthotopic azoxymethane/dextran sulfate sodium (AOM/DSS) colitis-associated murine carcinoma model, which is widely recognized for its reproducibility and relevance to human sporadic CRC.^61^ C57BL/6 mice were first treated with the carcinogen AOM, followed by three cycles of DSS to induce colitis. Two weeks after the final DSS cycle, animals were randomly divided into two groups, which underwent daily intraperitoneal EM127 or vehicle injections. Following 12 days of treatment (Fig. 6i), mice were euthanized and tissues were collected for further analysis (Fig. 6j). Interestingly, EM127 significantly reduced the number of tumors compared to the vehicle, and most tumors were smaller in size (<2 mm) (Fig. 6k). Histopathological examination of hematoxylin and eosin-stained colon sections from EM127-treated mice predominantly revealed low-grade dysplastic lesions, accompanied by inflammatory infiltrates and features consistent with apoptosis, indicative of substantial tumor regression (Fig. 6l). Furthermore, apoptotic cell evaluation by TUNEL staining revealed a significant increase in apoptotic cells in tumor sections from mice treated with EM127 compared to animals injected with the vehicle (Fig. 6l). Collectively, these findings support the therapeutic potential of EM127 in preclinical models of CRC.

### In vivo studies to address the potential of targeting SMYD3 in CRC preclinical metastatic mice models

**Fig. 7:**
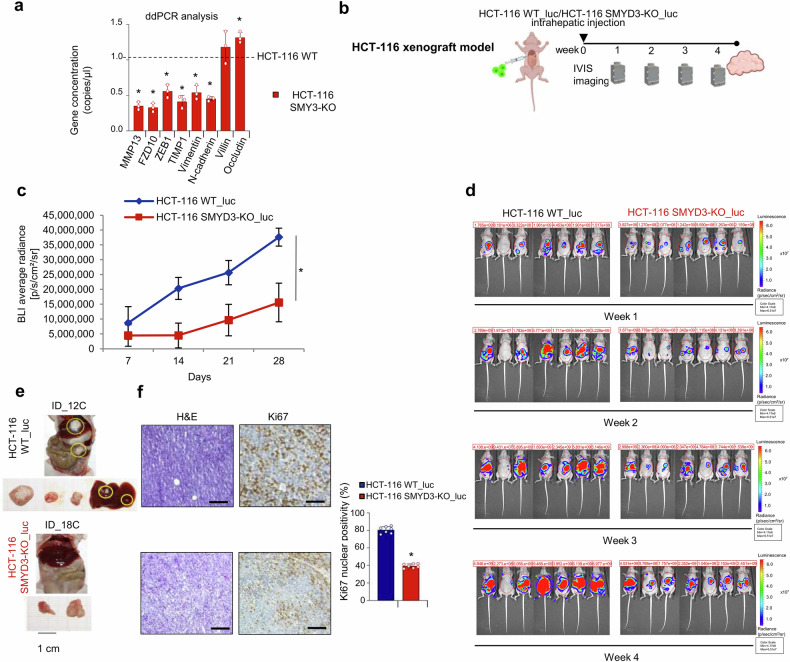
In vivo studies to address the potential of targeting SMYD3 in CRC preclinical metastatic mice models. **a** ddPCR analysis of EMT genes in SMYD3-KO vs WT HCT-116 tumorspheres. Data are expressed as means ± SD of 3 independent experiments. **b** Schematic illustration of the establishment of WT_luc and SMYD3-KO_luc HCT-116 cell xenograft mice. **c**, **d** Analysis of bioluminescence signal emission in whole animals treated as depicted in **b**. Bioluminescence imaging (BLI) average radiance measured over time (**c**) and images of bioluminescence emission by IVIS taken once a week for four weeks, with the intensity of photon emission being represented as a pseudo-color image (**a** representative scale bar is shown on the right) (**d**). **e** Representative necroscopy of mice treated as depicted in **b** showing numerous tumor masses (yellow circles). **f** Hematoxylin and eosin (H&E) and Ki67 immunohistochemical staining (left panel, scale bar: 100 μm) and bar plot summarizing the mean ± SD percentage of Ki67-positive cells (each dot represents one mouse, right panel) of tumors explanted from mice treated as depicted in **b**. *p < 0.05 SMYD3-KO vs WT parental cells

Since different reports have suggested that HCT-116 cells can form distant metastatic colonies in the lung and liver,^62^ we used these cells as a model system for examining the function of SMYD3 in the metastatic cascade. Considering that the EMT has been implicated in the metastatic process by enhancing mobility, invasion, and resistance to apoptotic stimuli,^63^ we investigated the expression of EMT markers by ddPCR analysis in our CRC models. Interestingly, SMYD3-KO HCT-116 cells showed reduced expression levels of mesenchymal genes, such as MMP13, FZD10, ZEB1, TIMP1, Vimentin, and N-cadherin, compared to their WT counterpart, while a slight increase was observed in the expression of epithelial genes (Villin, Occludin) (Fig. 7a). These results prompted us to perform in vivo studies. First, we infected WT and SMYD3-KO HCT-116 cells with the pHAGE PGK-GFP-IRES-LUC-W vector, which contains the luciferase coding sequence, to obtain luciferase-expressing cells detectable in metastatic processes. Then, WT_luc and SMYD3-KO_luc HCT-116 cells were intrahepatically injected into athymic nude mice. Intrahepatic tumor growth was evaluated weekly by in vivo imaging system (IVIS) measurement (Fig. 7b). Bioluminescence imaging (BLI) is a readily accessible, repeatable tool for the evaluation of interventional oncologic therapies in preclinical models.^64^ After 14 days from the injection, the average relative bioluminescence was significantly greater in WT_luc HCT-116 cells compared to SMYD3-KO_luc HCT-116 cells, and this difference increased over time (Fig. 7c, d). At the end of the experiment, xenograft tumors were harvested and processed for histological analysis (Fig. 7e, f). Examination of tissue sections revealed significant tumor regression and reduction in Ki67 staining in samples derived from SMYD3-KO HCT-116-xenografted mice (Fig. 7f).

**Fig. 8:**
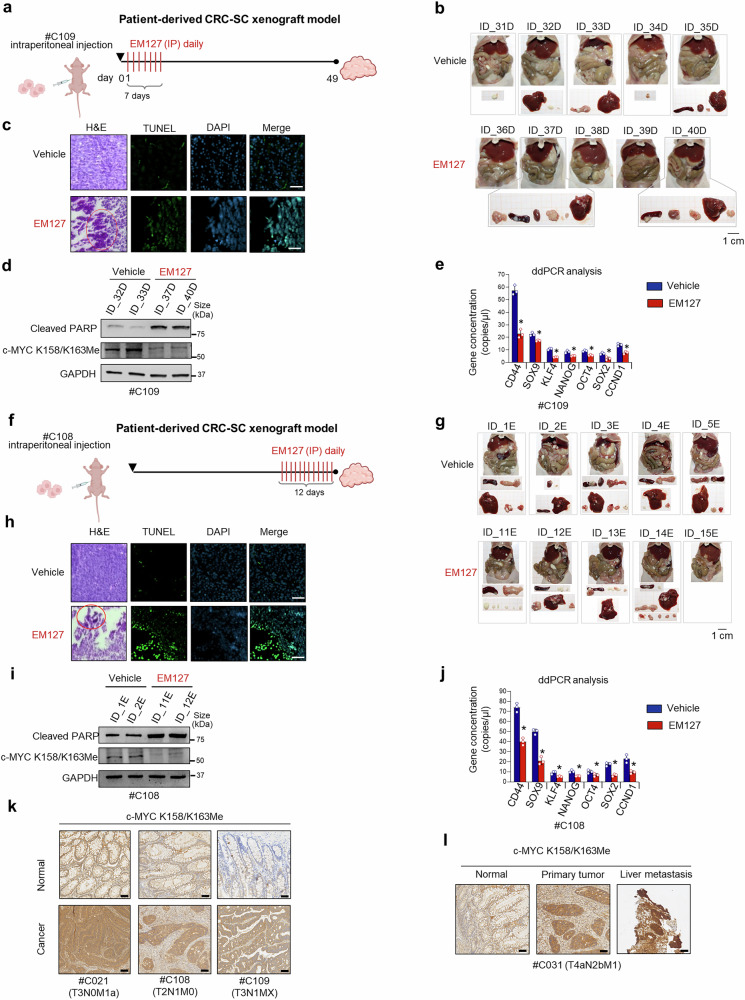
In vivo studies to address the potential of targeting SMYD3 in patient-derived CRC-SC metastatic mice models. **a** Schematic illustration of the establishment of patient-derived CRC-SC xenograft mice and EM127 treatment. Starting from day 1 after cell inoculation, mice were treated with daily intraperitoneal injections of EM127 (10 mg/kg) or the vehicle alone for seven days and sacrificed after six further weeks. **b**−**e** Analysis of tumors explanted from mice treated as depicted in **a**. Representative necroscopy showing numerous tumor masses (**b**), hematoxylin and eosin (H&E) staining with calcium salts visible in the red circle and TUNEL staining (**c**, scale bar: 50 μm), immunoblot analysis of Cleaved PARP and c-MYC K158/K163Me levels (GAPDH was used as a loading control) (**d**), and ddPCR analysis of stemness-related c-MYC target genes (**e**). **f** Schematic illustration of the establishment of patient-derived CRC-SC xenograft mice and EM127 treatment. Thirty days after cell inoculation, mice were treated with daily intraperitoneal injections of EM127 (10 mg/kg) or the vehicle alone for 12 days and then sacrificed. **g**–**j** Analysis of tumors explanted from mice treated as depicted in f. Representative necroscopy showing numerous tumor masses (**g**), hematoxylin and eosin (H&E) staining with calcium salts visible in the red circle and TUNEL staining (**h**, scale bar: 50 μm), immunoblot analysis of Cleaved PARP and c-MYC K158/K163Me levels (GAPDH was used as a loading control) (**i**), and ddPCR analysis of stemness-related c-MYC target genes (**j**). **k**, **l** Representative immunohistochemical analysis of c-MYC K158/K163Me levels in normal and cancer tissues from CRC patients (**k**, scale bar: 100 μm) and in normal tissue, primary tumor, and liver metastasis from metastatic CRC patients (**l**, scale bar: 100 μm). The TNM staging is indicated in parenthesis for each sample. *p < 0.05 EM127-treated vs untreated. Where applicable, data are expressed as means ± SD of 3 independent experiments

We next assessed the impact of SMYD3 pharmacological inhibition in preventing the spreading of patient-derived CRC-SCs in a xenograft metastasis model. Given that the liver and peritoneum are the primary sites of colorectal cancer dissemination, patient-derived CRC-SCs were intraperitoneally injected into CD1 nude female mice. The animals were distributed into two experimental groups, which were treated for 7 days with the vehicle or with EM127 to assess the potential of SMYD3 inhibition to prevent tumor metastasis. After 6 weeks, mice were sacrificed (Fig. 8a). Necroscopic evaluation revealed the complete absence of tumor masses in 60% of EM127-treated mice vs 0% of mice treated with the vehicle (Fig. 8b), suggesting a role for SMYD3 inhibition in preventing CRC metastatic spreading. At the end of the experiment, the explanted xenograft tumors were subjected to several analyses. Flow cytometry and immunofluorescence assays revealed reduced expression of CRC-SC markers, such as CD44, EpCAM, and CD133, in tumor mass samples explanted from EM127-treated mice (Supplementary Fig. 9a, b). Moreover, histological examination revealed the accumulation of calcium salts, the main histological marker of damaged tissues,^65^ in tumor regression areas of EM127-treated mice (Fig. 8c). We also detected the activation of the apoptotic pathway upon SMYD3 inhibition, as shown by TUNEL assay (Fig. 8c) and immunoblotting for Cleaved PARP (Fig. 8d). In addition, samples from EM127-treated mice showed reduced levels of c-MYC K158/K163Me (Fig. 8d) and a significant decrease in the expression of CRC-SC markers compared to vehicle-treated animals (Fig. 8e).

Then, we evaluated the effect of SMYD3 pharmacological treatment in an established patient-derived CRC-SC xenograft metastasis model. Patient-derived CRC-SCs were intraperitoneally injected into CD1-nude female mice, and 30 days after cell inoculation, mice were treated for 12 days with EM127 or the vehicle alone (Fig. 8f). Necroscopic evaluation showed the complete absence of tumor masses in 20% of EM127-treated mice vs 0% of mice treated with the vehicle (Fig. 8g). At the end of the experiment, xenograft tumors were harvested and processed for histological analysis, revealing extensive areas of tumor regression (Fig. 8h) due to apoptotic processes, as indicated by TUNEL staining (Fig. 8h) and cleaved PARP analysis (Fig. 8i). The cytotoxic effect of EM127 was also associated with reduced levels of c-MYC K158/K163Me (Fig. 8i), resulting in the downregulation of c-MYC target genes identified as CRC-SC stemness markers (Fig. 8j). Finally, to evaluate the clinical relevance of c-MYC methylation by SMYD3, we assessed c-MYC K158/K163Me levels by immunohistochemistry in normal and cancer tissues from CRC patients with advanced-stage cancer. Our results showed strong and diffuse staining across cancer tissues, with markedly higher levels of c-MYC methylation compared to their normal counterpart (Fig. 8k). In addition, immunohistochemical analysis of c-MYC K158/K163Me levels in CRC tissues and paired liver metastatic lesions revealed increased c-MYC methylation in both primary tumors and liver metastases, while weak staining was observed in normal tissues (Fig. 8l). These findings further support the clinical relevance of c-MYC methylation by SMYD3.

Overall, these findings indicate that SMYD3 significantly contributes to the development and progression of primary CRC, as well as to its metastatic dissemination in vivo.

### Targeting SMYD3 in patient-derived CRC-SCs to circumvent c-MYC mediated 5-FU chemoresistance

**Fig. 9:**
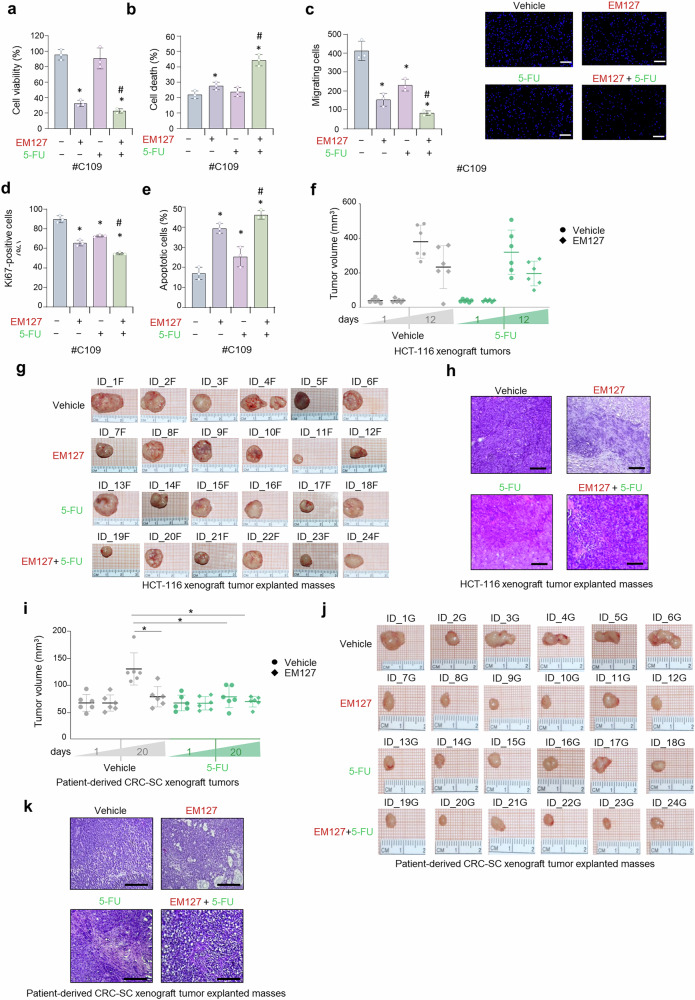
Targeting SMYD3 in patient-derived CRC-SCs to circumvent c-MYC mediated 5-FU chemoresistance. **a** Quantification of cell viability by CellTiter-Glo in patient-derived CRC-SCs pre-treated or not with EM127 (10 μM) for 48 h and then treated or not with 5-FU (10 μM) for 24 h. **b** Quantification of cell death by Trypan blue staining in patient-derived CRC-SCs treated as described in **a**. **c** Migratory ability of growth factor-starved patient-derived CRC-SCs placed in the inner chamber of transwell plates and treated or not with EM127 (10 μM) and/or 5-FU (10 μM) for 16 h. Migrating cells were fixed and counted under a fluorescence microscope. Scale bar: 100 μm. **d** Bar plot representation of the flow cytometry analysis of Ki67 expression in patient-derived CRC-SCs treated as described in **a**. **e** Bar plot representation of the flow cytometry analysis of annexin V staining in patient-derived CRC-SCs treated as described in **a**. The graph summarizes the percentage of apoptotic cells (early + late). **f**−**h** Analysis of tumors explanted from WT HCT-116 cell xenograft mice treated with EM127 (10 mg/kg daily) and/or 5-FU (25 mg/kg every 3 days) or the vehicle alone for 12 days. Tumor volume (data are expressed as means ± SD, each dot represents one mouse) (**f**), representative images (**g**), and hematoxylin and eosin (H&E) staining (**h**, scale bar: 100 μm). **i**–**k** Analysis of tumors explanted from patient-derived CRC-SC xenograft mice treated with EM127 (10 mg/kg daily) and/or 5-FU (25 mg/kg every 3 days) or the vehicle alone for 12 days. Tumor volume (data are expressed as means ± SD, each dot represents one mouse) (**i**), representative images (**j**), and hematoxylin and eosin (H&E) staining (**k**, scale bar: 200 μm). *p < 0.05 treated vs untreated; #p < 0.05 combined treatment vs corresponding single treatments. Where applicable, data are expressed as means ± SD of 3 independent experiments

Previous reports showed that c-MYC regulates the transcription of cancer stemness genes and promotes chemoresistance, especially against 5-fluorouracil (5-FU).^66^ We previously performed an extensive characterization of SMYD3 function in mediating cancer cell sensitivity to chemotherapeutic drugs.^5^ Prompted by these data, we investigated the potential of EM127 as a sensitizing agent in chemoresistant patient-derived CRC-SCs within a combinatorial strategy involving 5-FU. To this end, patient-derived CRC-SCs pre-treated with EM127 for 48 h were subsequently treated with 5-FU for 24 h. Our results showed that this combined therapeutic approach has nonlinear cumulative effects and is more effective than 5-FU alone. Specifically, pre-treatment with EM127 led to a significant reduction in CRC-SC proliferative index (Fig. 9a) and a concomitant increase in cell death (Fig. 9b). These findings support the potential of inhibiting SMYD3 to enhance 5-FU sensitivity in CRC. Moreover, we found that co-treatment with EM127 and 5-FU leads to a remarkable decrease in CRC-SC migratory ability (Fig. 9c). We further investigated the biological impact of co-treatment with EM127 and 5-FU on patient-derived CRC-SC fate by flow cytometry analysis. Ki67 assays showed that EM127 enhanced the growth-inhibitory activity of 5-FU (Fig. 9d), and this effect was accompanied by increased apoptotic cell death (Fig. 9e).

To assess the impact of SMYD3 pharmacological inhibition on chemosensitivity in vivo, we monitored tumor growth in a xenograft mouse model. Xenograft tumors were established by injecting WT HCT-116 cells into athymic nude mice. Once tumors reached a measurable size, the animals were randomly assigned to four groups, which were treated with the vehicle, 5-FU, EM127, or both 5-FU and EM127. 5-FU treatments were administered every 3 days, while EM127 was administered by daily intraperitoneal injection for 12 days, and tumor size and body weight were recorded every 2–3 days. After 12 days, tumor volume in mice treated with EM127 or both 5-FU and EM127 was significantly lower compared to the vehicle alone (Fig. 9f, g). In particular, the combined treatment with 5-FU and EM127 significantly arrested the growth of WT HCT-116 xenograft tumors (Fig. 9f, g). At the end of the treatment, xenograft tumors were harvested and processed for histological analysis (Fig. 9h). This examination showed marked tumor regression, which was mainly characterized by the presence of fibrotic tissue tending to restrain neoplasm invasion along with necro-inflammatory areas in tumors subjected to the combined treatment with EM127 and 5-FU compared to those treated with the vehicle alone. Interestingly, peripheral necrosis with signs of inflammation and fibrotic areas tending to restrain neoplasm invasion was observed in EM127-treated tumors, while expansive growth was detected in WT HCT-116 xenograft tumors exposed to the vehicle alone. 5-FU-treated tumors also revealed expansive growth, along with poor presence of fibrotic tissue and low evidence of necrosis (Fig. 9h).

Subsequently, we performed the same treatment schedule in a patient-derived CRC-SC xenograft tumor mice model. Our results showed that on day 12, the groups treated with EM127 or 5-FU exhibited smaller tumor masses compared to the vehicle group (Fig. 9i, j). A significant reduction in tumor growth was also observed in animals subjected to the combined treatment with EM127 and 5-FU. Histological analysis revealed marked tumor regression, characterized by the presence of fibrotic tissue tending to restrain neoplasm invasion, in mice treated with both EM127 and 5-FU compared to the vehicle alone. Notably, in the group treated with both drugs, no evidence of viable neoplasms was detected. Moreover, apoptosis and peripheral necrosis with signs of inflammation were observed in EM127-treated tumors, while expansive growth was detected in tumors from the vehicle group. Tumors treated with 5-FU revealed viable neoplastic cells, few signs of regression, and low presence of fibrotic tissue (Fig. 9k). Importantly, the significant reduction in tumor masses was not associated with loss of body weight, rectal bleeding, or rectal prolapse in any of the three active treatment groups (EM127 alone, 5-FU alone, and combined EM127 and 5-FU), suggesting that these treatments did not affect the overall well-being or nutritional status of the animals. These results indicate that EM127, alone or in combination with 5-FU, may be considered a promising treatment for tumor mass reduction in CRC, as it has a lasting therapeutic effect with a favorable safety profile, warranting further studies to fully explore its clinical potential. Collectively, all the above data suggest that SMYD3 pharmacological inhibition may prove effective both as monotherapy and in combination with 5-FU for CRC patient treatment.

## Discussion

The prevalence of CRC has been growing at an alarming rate,^67^ with the efficacy of current therapeutic strategies being limited by the development of therapy resistance, ultimately resulting in tumor relapse, metastatic progression, and overall poor prognosis.^68^ In particular, CRC-SCs are potential seeds for tumor relapse due to their resilience to adverse microenvironmental conditions^6^; hence, their eradication is necessary for effective cancer therapies. As a result, the identification of molecular targets that are positively selected and are essential for stemness maintenance and tumor growth may lead to the design of successful therapies for CRC.

One of the main players involved in CRC-SC biology is c-MYC, which acts as a link between stemness and malignancy.^69^ c-MYC has been found overexpressed in up to 80% of sporadic CRCs^70^ and consistently upregulated in CRC-SCs, while its downregulation has been shown to prevent CRC-SC self-renewal and xenograft growth.^69^ Moreover, deletion of the c-MYC gene has been shown to suppress intestinal tumorigenesis in CRC mouse models, indicating that its function is essential in this process.^71^

CRC cell fate is governed by an intricate network of signaling pathways that intersect with epigenetic regulators at the chromatin level.^72^ In this context, we and other groups have provided evidence of the relevance of epigenetic factors in cancer stemness.^17,73^ In particular, we hypothesized that SMYD3 can contribute to the maintenance of the CSC state, acting not only by regulating the expression of stemness genes but also through functional interactions and direct methylation of stemness proteins.

Here, we characterized the involvement and the mechanism of action of SMYD3 in the intricate network of cancer stemness signaling cascades that regulate the onset, progression, and metastatization of CRC. Our data indicate that SMYD3 plays a major role in CRC-SC self-renewal, migration, and metastatization. Mechanistically, we showed that SMYD3 contributes to CRC carcinogenesis by interacting with c-MYC and modulating its methylation status. Specifically, SMYD3 methylates c-MYC at K158 and K163, two evolutionarily conserved residues. This methylation may influence response to WREs by enabling c-MYC to interact with chromatin and induce stemness gene expression. The recruitment of methyltransferases to promoter regulatory elements can have important functional consequences; indeed, these enzymes can both methylate histones, thereby modulating the accessibility of chromatin to transcription factors,^74^ and directly methylate transcription factors, thereby regulating their transcriptional activity.^75,76^ For example, previous reports demonstrated that lysine methylation of p53 and nuclear factor-κB enhances their promoter-binding capacity and activate downstream target gene expression.^77,78^ In addition, further evidence revealed a complex model in which the binding of transcription factors to the DNA facilitates the recruitment of chromatin modification complexes, which can subsequently affect the transcription factors themselves as well as histones.^79^ Similarly, our data suggest that regulation of c-MYC transcriptional activity in CRC depends heavily on SMYD3-dependent c-MYC methylation at K158 and K163, and warrant further studies to corroborate the potential of targeting c-MYC methylation as a therapeutic approach in CRC therapy. In particular, as a next step in our research program, we plan to screen a large cohort of paired normal and CRC samples for c-MYC K158/K163Me levels and correlate c-MYC methylation with key clinical outcomes, such as survival rates and treatment response, in order to validate it as a clinical biomarker.

Importantly, in this study we used EM127, a site-specific SMYD3 covalent inhibitor, in several experimental models that recapitulate tumor complexity and, as such, are more relevant to clinical practice. Specifically, besides taking advantage of CRC cell lines capable of generating tumorspheres that are enriched in CSCs, we used patient-derived CRC-SCs cultured as spheroids and organoids, along with CRC xenograft and metastatic murine models.

Overall, our findings support a major role for SMYD3 in CSC biology. Indeed, we found that its pharmacological inhibition affects the clonogenic and self-renewal potential of patient-derived CRC-SCs by altering their molecular signature. Moreover, we showed that SMYD3 stable knock-out or pharmacological inhibition drastically decreases CRC tumorigenicity in vivo and reduces the metastatic potential of CRC-SCs, indicating that SMYD3 is involved in primary tumor initiation and metastatic tumor dissemination. This sound evidence about the role of SMYD3 in cancer stemness makes it a promising target for the development of personalized therapies for CRC patients with SMYD3-overexpressing tumors. Moreover, the identification of SMYD3 as a novel key co-factor for c-MYC cancer-promoting functions opens new avenues for taking advantage of the indirect inhibition of an “undruggable” factor such as c-MYC^80^ and suggests that this pharmacological strategy may efficiently target the SMYD3/c-MYC axis in CRC. For this reason, findings from this work may have important translational relevance and potential clinical applicability, helping to expand the armamentarium of personalized targeted therapies in CRC.

In this work, we focused on the impact of SMYD3 inhibition on c-MYC functions, but we did not analyze the further interactors identified in our in silico screening analysis. These interactors may also play a role in the SMYD3/c-MYC axis and will be addressed in future studies. Moreover, while we showed that SMYD3 can efficiently methylate c-MYC at K158 and K163, and that this modification is associated with the expression of c-MYC target genes, the underlying molecular mechanisms mediating c-MYC transcriptional activity still need to be elucidated. Furthermore, although we used the CRISPR/Cas9 genome editing technique to knock out SMYD3 in order to validate the results obtained in vivo with the specific SMYD3 inhibitor EM127, we cannot completely rule out the possibility of EM127 off-target effects. Finally, we are conscious that part of our molecular and cellular results is based on a small cohort of CRC patients.

## Materials and Methods

### ALDH assay

Cells were stained using the ALDEFLUOR Kit (01700, Stem Cell Technologies) according to the manufacturer’s instructions. Briefly, approximately 1 × 10^6^ cells were harvested and incubated in Aldefluor reagent for 40 min at 37 °C. Negative control samples were treated with the Aldefluor reagent DEAB as an inhibitor of ALDH enzymatic activity. The fluorescence intensity of stained cells was analyzed with a FACS Flow Cytometer (BD FACS CANTO II, Biosciences). DEAB-treated cells were used to define the baseline. Based on the fluorescence intensity below or beyond the threshold defined by the reaction with DEAB, each of the sorted cells was designated to be ALDH1-negative or ALDH1-positive cells.

### Annexin V staining

For this assay, 1 × 10^6^ cells were cultured in ultra-low attachment 6-well plates (3471, Costar Corning) at 37 °C, 5% CO_2_. After 24 h, cells were treated or not with EM127 or 5-fluorouracil. Next, 5 × 10^4^ cells/plate were collected and resuspended in 1X PBS-1% FBS, and the Muse Annexin V and Dead Cell reagent (MCH100105, Luminex) was added to each tube. Cells were then incubated at room temperature for 20 min in the dark. Flow cytometry was performed using the Guava Muse Cell Analyzer (Merck Millipore). Cells were considered to be apoptotic if they were Annexin V + /PI- (early apoptotic) and Annexin V + /PI+ (late apoptotic). Each analysis was performed by evaluating at least 2000 events, using the assay-specific software module included in the Guava Muse Cell Analyzer instrument (Merck Millipore).

### Asymmetric cell division assay

Wild-type (WT) and SMYD3-knock-out (KO) HCT-116 cell preparation for 5-bromo-2’-deoxyuridine (BrdU) (ab126556 Abcam) incorporation was assessed following the principle of pulse-chase experiments as previously described,^81^ in order to test the ability of cells to asymmetrically divide their BrdU-labeled template DNA to one daughter cell only. Briefly, 1 µM BrdU was added to the culture medium every 48 h, for a total of two weeks, to ensure labeling of all cells. Both cell populations were synchronized with nocodazole (100 ng/mL) (M1404, Sigma-Aldrich) for 16 h. Then, BrdU was withdrawn for 48 and 96 h, and cells were harvested and immunostained for BrdU detection. To reveal BrdU-labeled DNA, harvested cells were fixed and permeabilized with a mixture of ethanol and NaOH (Fixing solution, ab126556 Abcam) for 30 min at the indicated time points. Next, cells were washed with PBS, incubated with an anti-BrdU antibody (Prediluted anti-BrdU Detector Antibody, ab126556 Abcam) for 1 h and then with Alexa Fluor 488 (A-11094, Thermo Fisher Scientific) for 30 min at room temperature, and subjected to flow cytometry analysis. Gating strategies for data analysis were carried out as previously reported.^30^

### Bioinformatic analysis

After sequencing, we imported FASTQ files into a Lexogen platform for quality controls and alignment. Subsequently, we acquired the raw gene counts. For cell line RNA-seq analysis, we used the edgeR Bioconductor/R package (version 3.42.4).^82^ Low-expression genes were filtered out by the ‘filterByExpr’ function, and TMM normalization was applied. Afterwards, we performed a differential expression analysis (DEA) using the Generalized Linear Model (GLM). Two comparisons were performed: (1) SMYD3-KO vs WT HCT-116 cells, and (2) EM127-treated vs untreated WT HCT-116 cells. For each comparison, we set a log fold change (logFC) >= 1 or <= −1 and False Discovery Rate (FDR) < 0.01 to identify significantly differentially expressed (DE) genes. In order to ascertain in silico the biological function of the DE genes, we performed a Gene Ontology enrichment analysis using the clusterProfiler Bioconductor/R package (version 4.8.3).^83^ Only terms/pathways with an adjusted *p*-value < 0.05 were considered. Additionally, a Preranked Gene Set Enrichment Analysis (GSEA)^41^ was carried out to detect enriched hallmarks in the patient’s cohort. The absolute values of a normalized enrichment score > 1 and a false discovery rate (FDR) *q*-value < 0.25 were considered as GSEA sets. Moreover, c-MYC target genes were further analyzed using the clusterProfiler Bioconductor/R package (version 4.8.3) and a Reactome enrichment analysis using the ReactomePA Bioconductor/R package (version 1.46.0).^84^ Only terms/pathways with an adjusted *p*-value < 0.05 were considered. Based on data reported in the ChEA database, an integrated genome-wide ChIP-X experiment dataset, we identified in silico the c-MYC target genes involved in stemness-related processes (https://maayanlab.cloud/Harmonizome/dataset/CHEA+Transcription+Factor+Targets).^43^ In addition, based on CancerMine database annotations (http://bionlp.bcgsc.ca/cancermine), we classified c-MYC target genes as oncogenes or tumor suppressors in the context of CRC.^43^

### Cell death analysis

Cell death was assessed by counting. Briefly, a total of 2 × 10^3^ dissociated cells were plated in flat bottom, ultra-low attachment 96-well plates (3474, Costar Corning). After 24 h, cells were treated or not with EM127 for 72 h. Then, supernatants (containing dead/floating cells) were collected. Cell pellets were resuspended in 1X PBS, and 10 μL were mixed with an equal volume of 0.01% Trypan blue solution (T8154, Sigma-Aldrich). Viable cells (unstained, trypan blue-negative cells) and dead cells (stained, trypan blue-positive cells) were counted with a phase-contrast microscope, and the percentages of dead cells were calculated.

### Cell lines

Colorectal cancer (CRC) HCT-116, breast cancer MDA-MB-231, human embryonic kidney HEK-293 and HEK-293T cell lines were purchased from ATCC. SMYD3-KO HCT-116, c-MYC_K158A knock-in (KI) HCT-116, c-MYC_K163A KI HCT-116, and SMYD3-KO MDA-MB-231 cells were generated using the CRISPR/Cas9 technology. WT_luc HCT-116 and SMYD3-KO_luc HCT-116 cells were obtained by lentivirus infection. WT, SMYD3-KO, c-MYC_K158A KI, and c-MYC_K163A KI HCT-116 cells, and WT and SMYD3-KO MDA-MB-231 cells were cultured in DMEM high glucose (HG), without pyruvate (11360-070, Gibco) with 10% FBS (A5256701, Gibco) and 100 IU/mL penicillin-streptomycin (15140122, Gibco). HEK-293 and HEK-293T cells were cultured in DMEM high glucose (HG), without pyruvate (11360-070, Gibco) with 10% FBS (A5256701, Gibco), 100 IU/mL penicillin-streptomycin (15140122, Gibco), 1% sodium pyruvate (11360-039, Gibco), and 1% MEM non-essential amino acids (NEAA) (11140-035, Gibco). WT_luc and SMYD3-KO_luc HCT-116 cells were cultured in DMEM high glucose (HG), without pyruvate (11360-070, Gibco) with 10% FBS (A5256701, Gibco), 100 IU/mL penicillin-streptomycin (15140122, Gibco), and 0,5 mg/ml puromycin (A1113803, Gibco). Patient-derived CRC-SCs were isolated from CRC patients, who signed an informed consent form in accordance with the ethical standards of the responsible Institutional Committee (Ethics Committee name: Comitato Etico Istituto tumori “Giovanni Paolo II”, Istituto di ricovero e cura a carattere scientifico, Viale Orazio Flacco, 65-70124, Bari; approval code: Prot. n. 379/C.E.; approval date: 16 September 2020). Cells were isolated and propagated from CRC patients as previously described.^85,86^ Cells were dissociated using a tumor dissociation kit (130095929, Miltenyi) and propagated in Advanced DMEM/F-12 (12634010, Gibco) supplemented with 1% L-glutamine (25030081, Gibco), 100 IU/mL penicillin-streptomycin (15140122, Gibco), 0.6% glucose solution (G8769, Sigma-Aldrich), B-27 (12587010, Gibco), N-2 (17502048, Gibco), 10 ng/mL HbFGF (F0291, Sigma-Aldrich), 20 ng/mL EGF (E9644, Sigma-Aldrich). Cells were routinely propagated under standard conditions. All cell lines were tested to be mycoplasma-free (117048, Minerva Biolabs) multiple times throughout the study. All cell cultures were performed in a 37 °C and 5% CO_2_ incubator.

### Cell transfection

SMYD3-KO HCT-116 and SMYD3-KO MDA-MB-231 tumorspheres were transiently transfected with the mammalian expression plasmid pEGFP-N3-SMYD3 using the Neon NxT Electroporation System (Thermo Fisher Scientific) according to the manufacturer’s instructions.

Recombinant lentiviruses were generated by transfection of 80% confluent HEK-293T cells with the modified pHAGE PGK-GFP-IRES-LUC-W vector (46793, Addgene) and the packing plasmids psPAX2 (12260, Addgene) and pMD2.G (12259, Addgene) using Lipofectamine 3000 (L3000015, Thermo Fisher Scientific) according to the manufacturer’s instructions.

HEK-293 cells were transiently transfected with the mammalian expression plasmids p3xFLAG CMV14 empty, p3xFLAG CMV14 c-MYC, p3xFLAG CMV14 c-MYC-K158A, and p3xFLAG CMV14 c-MYC-K163A using Lipofectamine 3000 (L3000015, Thermo Fisher Scientific) according to the manufacturer’s instructions.

To obtain recombinant proteins, HEK-293 cells were transiently transfected with the mammalian expression plasmids p3xFLAG CMV14 c-MYC-M1 (aa 1-375), p3xFLAG CMV14 c-MYC-M2 (aa 266-454), and p3xFLAG CMV14 c-MYC-M3 (aa 1-165) using Lipofectamine 3000 (L3000015, Thermo Fisher Scientific) according to the manufacturer’s instructions.

### Cell viability and cell proliferation assay

Cell viability and cell proliferation were assessed using the CellTiter-Glo Luminescent Cell Viability Assay Kit (G7570, Promega) and CellTiter 96 AQueous One Solution Cell Proliferation Assay (MTS) Kit (G3582, Promega), respectively, according to the manufacturer’s instructions. Briefly, a total of 2 × 10^3^ dissociated cells were plated in flat-bottom, ultra-low attachment 96-well plates (3474, Costar Corning). After 24 h, cells were treated or not with EM127 for 72 h. Then, cells were processed according to the manufacturer’s instructions. The luminescent signal was read using the SPECTROstar Omega microplate reader (BMG Labtech).

### Chemicals

Azoxymethane (AOM) (A5486), BCI-121 (SML1817), 5-bromo-2’-deoxyuridine (BrdU) (ab126556, Abcam), 5-fluorouracil (5-FU) (S-F6627), Trypan blue (T8154), and Wnt3a (H17001) were all purchased from Sigma-Aldrich. Dextran sodium sulfate (90-11-18-1) was purchased from MP Biomedicals. EM127 was obtained through the straightforward procedure described in Parenti et al.^31^ For nanoparticle formulation, human serum albumin (HSA) (A1653) and α-tocopherol (T3251) were purchased from Sigma-Aldrich. The P14 (NFY), P14ext1 (EEENFYQQQ), and P14ext2 (PYFYCDEEENFYQQQQQSEL) peptides were custom purchased from Thermo Fisher Scientific. For each chemical, doses and treatment duration are indicated in the figure legends.

### Chromatin immunoprecipitation

Chromatin isolated from WT, c-MYC_K158A KI, and c-MYC_K163A KI HCT-116 cells and patient-derived CRC-SCs was subjected to chromatin immunoprecipitation (ChIP) using the MAGnify Chromatin Immunoprecipitation System (492024, Thermo Fisher Scientific) according to the manufacturer’s instructions. Briefly, cells were cross-linked in 1% formaldehyde (252549, Sigma-Aldrich) for 10 min. Cross-linking was blocked with 0.125 M glycine (23390.04, Serva) for 5 min, then cells were washed with 1X PBS and lysed in lysis buffer. Chromatin was sonicated to a fragment length of about 200-500 bp and immunoprecipitated with 1 µg of rabbit IgG anti-SMYD3 (12859, Cell Signaling Technology), anti-c-MYC (9402, Cell Signaling Technology), anti-H3K27Ac (ab4729, Abcam), and anti-c-MYC K158/K163Me (custom made, project code 2ZG1940M, Thermo Fisher Scientific) antibodies. Quantitative real-time PCR was performed using PowerUp SYBR Green Master Mix (A25741, Thermo Fisher Scientific). ChIP primer sequences are available upon request. Results are representative of at least three independent experiments.

### Cloning and mutagenesis

The plasmids described in this manuscript were generated with specific primers, as previously described.^12^

The pEGFP-N3-SMYD3 construct was generated starting from the human SMYD3 cDNA ORF in the pMD18-T Simple cloning vector (HG11217-M, Sino Biological), used as a template to amplify SMYD3. The EcoRI_SMYD3_FW 5’-ATTACGAATTCTATGGAGCCGCTGAAG and EcoRI_SMYD3_RV 5’-ATTACGAATTCTGGGATGCTCTGATGT primers were used for the PCR. The SMYD3 fragment was cloned into the pEGFP-N3 empty vector (Takara) linearized with EcoRI (R3101S, New England Biolabs).

The p3xFLAG CMV14 c-MYC-WT construct was generated starting from the human pcDNA3-c-MYC plasmid (16011, Addgene), used as a template to amplify c-MYC. The NotI_c_MYC_FW 5’-CCAGCGGATCCATGGATTTTTTTCGGGTAGTGGAAAACCAGCAGCCTCCCGCGACGATGCCCCTCAACGTTAGCTTCACC and BamHI_c_MYC RV 5’-TTCAGCCCGGGATCCCGCACAAGAGTTCCGTAGCTG primers were used for the PCR. The c-MYC fragment was cloned into the p3xFLAG-CMV14 empty plasmid (E7908, Sigma-Aldrich) linearized with NotI and BamHI (R0189, and R0136, New England Biolabs, respectively).

The p3xFLAG CMV14 c-MYC-K158A and p3xFLAG CMV14 c-MYC-K163A constructs were generated using the Q5® Site-Directed Mutagenesis Kit (#E05545, New England Biolabs) according to the manufacturer’s instructions.

The p3xFLAG CMV14 c-MYC-M1 (aa 1-375), p3xFLAG CMV14 c-MYC-M2 (aa 266-454), and p3xFLAG CMV14 c-MYC-M3 (aa 1-165) constructs were generated starting from the p3xFLAG CMV14 c-MYC-WT construct, used as a template to amplify c-MYC fragments. Primers used for these amplifications are listed below:

NotI_ c_MYC FW 5’-CTTAAGCTTGCGGCCGCATGGATTTTTTTCGGGTAGTGGA

BamHI_ c_MYC 375 RV 5’-ATCAGCCCGGGATCCGTTGTGTGTTCGCCTCTTGACA

BamHI c_MYC 165 RV 5’-TCAGCCCGGGATCCGGCCAGCTTCTCTGAGACGAG

NotI_c_MYC 266 FW 5’-GAATTAAGCTTGCGGCCGCATGGACTCTGAGGAGGAACAAGAAG

BamHI_c_MYC RV 5’-TTCAGCCCGGGATCCCGCACAAGAGTTCCGTAGCTG

c-MYC fragments were cloned into the p3xFLAG-CMV14 empty plasmid (E7908, Sigma-Aldrich) linearized with NotI and BamHI (R0189 and R0136, New England Biolabs, respectively).

The pGEX-4T-3 c-MYC construct was generated by cloning the c-MYC fragment into the pGEX-4T-3 expression vector (28-9545-52, GE Healthcare) using the BamHI and NotI enzymes (R0136 and R0189, New England Biolabs, respectively). The c-MYC-K158A and c-MYC-K163A constructs were generated by site-directed mutagenesis, performed using the Q5® Site-Directed Mutagenesis Kit (E05545, New England Biolabs) according to the manufacturer’s instructions. Site-directed mutagenesis was performed using the following primers:

- SDM_c-MYC_K158A_FW 5’-GGCCGCCGCCGCGCTCGTCTCA
- SDM_c-MYC_K158A_RV 5’-GAGAAGCCGCTCCACATAC
- SDM_c-MYC_K163A_FW 5’-CGTCTCAGAGGCGCTGGCCTCCTACCAGGCTG
- SDM_c-MYC_K163A_RV 5’-AGCTTGGCGGCGGCCGAG

pET28-MHL-SMYD3 (#32048), GST-Hsp90 C(626-732) (#22483), pHAGE PGK-GFP-IRES-LUC-W vector (#46793), psPAX2 (#12260) and pMD2.G (#12259) were purchased from Addgene. Efficiency DH5α competent cells (C2987H) and BL21 (DE3) competent E. coli cells (C2527I) purchased from New England Biolabs were used for all cloning experiments performed in this study. Cells were grown in Luria broth medium (10855021, Gibco).

### c-MYC reporter luciferase assay

HEK-293 cells were transiently co-transfected with the mammalian expression plasmid p3xFLAG CMV14 c-MYC-WT or the relevant mutants (K158A and K163A) and the c-MYC luciferase reporter vector + the constitutively expressing Renilla luciferase vector (component A) (60519, BPS Biosciences) using Lipofectamine 3000 (L3000015, Thermo Fisher Scientific) according to the manufacturer’s instructions. After 48 h, c-MYC activity was assessed using a Dual-Luciferase Reporter System (E1910, Promega) according to the manufacturer’s instructions. c-MYC activity was determined by the ratio of firefly to Renilla luciferase activity.

WT, c-MYC_K158A KI, and c-MYC_K163A KI HCT-116 cells were transiently transfected with the c-MYC luciferase reporter vector + the constitutively expressing Renilla luciferase vector (component A) (60519, BPS Biosciences) using Lipofectamine 3000 (L3000015, Thermo Fisher Scientific) according to the manufacturer’s instructions. c-MYC transcriptional activity was assessed as described above.

### Colony formation assay

For the clonogenic assay, dissociated cells were plated in triplicate at 500 cells/well suspended in 0.3% agarose over a layer of 0.5% agarose and treated or not with EM127 for 72 h. Then, cells were allowed to grow for 4 weeks. Plates were stained with crystal violet solution (80299, Liofilchem), washed with water several times to remove excess crystal violet, and then dried at room temperature. The area of the colonies was evaluated using ImageJ software.

### Co-immunoprecipitation

Cells were collected and homogenized in lysis buffer (50 mM Tris-HCl pH 7,4, 5 mM EDTA, 250 mM NaCl, and 1% Triton X-100) supplemented with protease and phosphatase inhibitors (78443, Thermo Fisher Scientific). Coupling between Dynabeads Protein A (10002D, Thermo Fisher Scientific) and antibodies, i.e., anti-SMYD3 (12859, Cell Signaling Technology) or anti-c-MYC (9402, Cell Signaling Technology), was performed in 100 μL of 0.01% Tween20-1X PBS for 45 min at room temperature on a rocking platform. Cell lysates were immunoprecipitated with antibody-bead complexes. Immunocomplexes were washed extensively, boiled in 2x Laemmli sample buffer (1610737, Bio-Rad Laboratories), and subjected to SDS-PAGE and immunoblot analysis. Input corresponds to 10% of the lysate. IgG was used as a negative control. Primary antibodies used: SMYD3 (12859, Cell Signaling Technology) and c-MYC (9402, Cell Signaling Technology). Rabbit IgG HRP (NA934V, GE Healthcare) was used as a secondary antibody and revealed using the Clarity Max Western ECL Substrate (1705062, Bio-Rad Laboratories) according to the manufacturer’s instructions.

### Construction of stable luciferase-expressing human CRC cells

Recombinant lentiviruses were generated by transfection of 80% confluent HEK-293T cells with the modified pHAGE PGK-GFP-IRES-LUC-W vector (46793, Addgene) and the packing plasmids psPAX2 (12260, Addgene) and pMD2.G (12259, Addgene) using Lipofectamine 3000 (L3000015, Thermo Fisher Scientific) according to the manufacturer’s instructions. Lentiviral particles were harvested at 24 h and 48 h after transfection. WT_luc and SMYD3-KO_luc HCT-116 cells were generated by lentivirus infection. For lentivirus infection, WT and SMYD3-KO HCT-116 cells were seeded into T25 flasks (430639, Costar Corning) at 50% confluency and infected with lentiviral particles. After 48 h, isolation of clonal populations was performed with agarose-based cloning rings (C1059, Sigma-Aldrich). Clones were screened by evaluating GFP-expressing cells under fluorescence microscopy.

### CRISPR/Cas9 system

To generate SMYD3-KO HCT-116 cells, WT HCT-116 cells were transfected with the TrueCut Cas9 Protein V2 and SMYD3 TrueGuide gRNAs (CRISPR1032607 and CRISPR1032618, Thermo Fisher Scientific) using Lipofectamine CRISPRMAX transfection reagent (CMAX00001, Thermo Fisher Scientific) according to the manufacturer’s instructions. After 48 h, isolation of clonal populations was performed with agarose-based cloning rings (C1059, Sigma-Aldrich). Cell clones were tested for site-specific loss-of-function alterations by PCR, using the following sequencing primers: SMYD3 gRNA FW 5’-AGCCCGTGAGACGCCCGCTGCTGG and SMYD3 gRNA RV 5’-GAAAAGTTCGCAACCGCCAA.To generate c-MYC_K158A KI HCT-116 cells, WT HCT-116 cells were transfected with the TrueCut Cas9 Protein (A36496, Thermo Fisher Scientific), MYC_C2 TrueGuide gRNAs 5’-CTCTGAGACGAGCTTGGCGG (A35534, Thermo Fisher Scientific), and MYC_C2_Donor DNA 5’-CAGGACTGTATGTGGAGCGGCTTCTCGGCCGCCGCCGAGCTCGTCTCAGAGAAGCTGGCCTCCTACCAGGCT. Similarly, c-MYC K163A KI HCT-116 cells were obtained by transfecting WT HCT-116 cells with the TrueCut Cas9 Protein (A36496, Thermo Fisher Scientific), MYC_C1 TrueGuide gRNAs 5’- CAAGCTCGTCTCAGAGAAGC (A35534, Thermo Fisher Scientific), and MYC_C1_Donor DNA 5’-GCGGCTTCTCGGCCGCCGCCAAGCTCGTCTCAGAGGCGCTGGCCTCCTACCAGGCTGCGCGCAAAGACAGCG.

Transfection was carried out using the Neon NxT Electroporation System (Thermo Fisher Scientific) according to the manufacturer’s instructions. After 48 h, isolation of clonal populations was performed. Cell clones were tested for site-specific substitution by PCR, using the following sequencing primers: MYC_SEQ_FWD_P2 FW 5’-AAGAAATTCGAGCTGCTGCC and MYC_SEQ_REV_P2 5’-TGCTGTCGTTGAGAGGGTAG. Sequencing products were purified using the BigDye XTerminator Purification Kit (4376486, Thermo Fisher Scientific) and sequenced on a SeqStudio Genetic Analyzer (Thermo Fisher Scientific).

### Droplet digital PCR (ddPCR) assay

ddPCR was conducted on treated or untreated cells and tumor samples. Chemicals, doses, and treatment duration are detailed in the figure legends.

ddPCR was performed using 10 ng of RNA extracted from patient-derived CRC-SCs and explanted patient-derived CRC-SC xenograft tumors and PDTOs or 1 ng of RNA obtained from WT, SMYD3-KO, c-MYC_K158A KI, c-MYC_K163A KI HCT-116 cells, HEK-293 cells and explanted HCT-116 xenograft tumors. RNA was extracted using the Purelink RNA Micro Kit (12183016, Thermo Fisher Scientific) and retro-transcribed to cDNA using the Super-Script VILO cDNA Synthesis Kit (11755050, Thermo Fisher Scientific) according to the manufacturer’s instructions. Reactions were prepared using the ddPCR Supermix for Probes Kit (1863024, Bio-Rad Laboratories) according to the manufacturer’s instructions. 20 μL of each reaction mix was converted into droplets with the QX200 Droplet Generator (Bio-Rad Laboratories). Droplets were transferred to a 96-well plate, sealed, and cycled in a C100 Thermocycler (Bio-Rad Laboratories) under the following cycling protocol: 25 °C for 3 min, 95 °C for 10 min followed by 40 cycles at 94 °C for 30 s and 60 °C for 1 min, a post-cycling step at 98 °C for 10 min, and infinite hold at 4 °C. The plate was then transferred into a QX200 Reader (Bio-Rad Laboratories). Data were analyzed using Bio-Rad QX Manager 1.2 Standard Edition. Probes were as follows:

- NANOG: ddPCR Gene Expression Assay, NANOG, Human, Homo sapiens (dHsaCPE5191856, Bio-Rad Laboratories).
- CCND1: ddPCR Gene Expression Assay, CCND1, Human, Homo sapiens (dHsaCPE5051730, Bio-Rad Laboratories).
- KLF4: ddPCR Gene Expression Assay, KLF4, Human, Homo sapiens (dHsaCPE5037001, Bio-Rad Laboratories).
- SOX9: ddPCR Gene Expression Assay, SOX9, Human, Homo sapiens (dHsaCPE5035865, Bio-Rad Laboratories).
- OCT4: ddPCR Gene Expression Assay, OCT4, Human, Homo sapiens (dHsaCPE5191336, Bio-Rad Laboratories).
- SOX2: ddPCR Gene Expression Assay, SOX2, Human, Homo sapiens (dHsaCPE5030245, Bio-Rad Laboratories).
- CD44: ddPCR Gene Expression Assay, CD44, Human, Homo sapiens (dHsaCPE5051601, Bio-Rad Laboratories).
- SMYD3: ddPCR Gene Expression Assay, SMYD3 Human, Homo sapiens (dHsaCPE5058764, Bio-Rad Laboratories).

For ddPCR analysis of epithelial-mesenchymal transition (EMT) genes, reactions were prepared using the ddPCR EvaGreen Supermix (1864033, Bio-Rad Laboratories) and specific primers (200 nM) according to the manufacturer’s instructions. Droplets were processed as described above and amplified under the following cycling protocol: a denaturation step at 95 °C for 5 min, followed by 40 cycles at 95 °C for 30 s and 60 °C for 1 min, a post-cycling step for signal stabilization at 4 °C for 5 min and 95 °C for 5 min, and infinite hold at 4 °C. The plate was then transferred into a QX200 Reader (Bio-Rad Laboratories). Data were analyzed using Bio-Rad QX Manager 1.2 Standard Edition. ddPCR primer sequences are available upon request.

### Preparation of EM127@HSA nanoparticles

HSA was dissolved in Milli-Q water to achieve a final concentration of 2.5 mg/mL. The solution was vigorously stirred for 5 min and then filtered using a Chromafil® Xtra RC-45/13 0.45 μm syringe filter. EM127 (3.6 mg/mL) and α-tocopherol (15 mg/mL) ethanolic stock solutions were then mixed and slowly added via syringe to the HSA solution to achieve a final EM127/HSA ratio of 15% w/w and α-tocopherol/HSA ratio of 3% w/w. Sudden opalescence indicated nanoparticle formation. Then, the mixture was further vigorously stirred for 10 min at room temperature, and 200 µL of the solution were withdrawn and diluted in a cuvette with 1.6 mL of Milli-Q water to perform the dynamic light scattering (DLS) analysis. EM127@HSA nanoparticles showed an average hydrodynamic diameter between 80 and 100 nm, with a polydispersity index (PDI) below 0.2 and a ζ -potential value of about -25 mV. The solution was then freeze-dried, producing a white powder of EM127@HSA, which was stored at 4 °C.

### Ethics approval and consent to participate

The study was conducted in accordance with the ethical standards of the Institutional Committee on Human Experimentation after informed consent (Ethics Committee name: Comitato Etico Istituto tumori “Giovanni Paolo II”, Istituto di ricovero e cura a carattere scientifico, Viale Orazio Flacco, 65-70124, Bari; approval code: Prot. n. 379/C.E.; approval date: 16 September 2020). The procedures involving animals were conducted in conformity with the institutional guidelines that comply with national and international laws and policies (Authorization number: 870/2021-PR). The experiment carried out by Biogem Animal House was assessed under Ministerial Authorization (nr: 08/2023-UT).

### Extreme limiting dilution assay (ELDA)

Clonogenicity activity of patient-derived CRC-SCs was determined by plating 1, 25, 50, or 100 cells/well in culture medium, and analyzed with the Extreme Limiting Dilution Analysis (ELDA) online software to calculate cancer cell initiating frequency and significance (http://bioinf.wehi.edu.au/software/elda/).

### Flow cytometry analysis

Explanted tumors from mice xenografted with patient-derived CRC-SCs were disaggregated using gentleMACS™ C Tubes (130-093-237, Miltenyi Biotec) with gentleMACS™ Dissociator (130-093-235, Miltenyi Biotec) according to the manufacturer’s instructions. Patient-derived CRC-SCs and cells obtained from explanted tumors were dissociated with trypsin, resuspended at 1 × 10^6^ cells/mL in 200 µL of PBS, and subsequently incubated with FITC-conjugated anti-CD44 (75122, Cell Signaling), FITC-conjugated anti-EpCAM (MA1-10197, Thermo Fisher Scientific), or PE-conjugated anti-CD133 (12-1338-42, Thermo Fisher Scientific) antibodies for 20 min at room temperature. Cells were then washed with ice-cold PBS and subjected to flow cytometry analysis. The fluorescence intensity of stained cells was analyzed with a FACS Flow Cytometer (BD FACS CANTO II, Biosciences). Isotype-matched immunoglobulins were used as controls. Dead cell exclusion was performed by using 7-AAD (IM3630C, Beckman Coulter).

### Gene expression profiling

Sequencing libraries were constructed from total RNA using the QuantSeq 3’ mRNA-Seq Library Prep Kit FWD (Lexogen) following the manufacturer’s instructions. After a quality check on the 4150 TapeStation System (Agilent), libraries were equimolarly pooled and sequenced on a NextSeq 500 System (Illumina) to an average depth of 7 × 10^6^ single-end reads. FastQ files were processed using the QuantSeq 3′ mRNA-seq pipeline implemented on the Lexogen genomic platform.

### Immunoblotting

Whole-cell extracts were obtained from cells and tissues. Cells were collected and homogenized in lysis buffer (50 mM Tris-HCl pH 7.4, 5 mM EDTA, 250 mM NaCl, and 1% Triton X-100) supplemented with protease and phosphatase inhibitors (78443, Thermo Fisher Scientific).

Tissues were collected and homogenized in T-PER™ Tissue Protein Extraction Reagent (78510, Thermo Fisher Scientific) supplemented with protease and phosphatase inhibitors (78443, Thermo Fisher Scientific). Tissue dissociation was carried out using the gentleMACS Tissue Dissociator (130-093-235, Miltenyi Biotec) and gentleMACS™ M Tubes (130-093-236, Miltenyi Biotec). 30 μg of protein extracts from each sample were denatured in 4x Laemmli sample buffer (1610747, Bio-Rad Laboratories) and loaded into an SDS-polyacrylamide gel for immunoblot analysis. Primary antibodies used: active-β-catenin (8814, Cell Signaling Technology), CD44 (3570, Cell Signaling Technology), cleaved Caspase-3 (9661, Cell Signaling Technology), cleaved PARP (5625, Cell Signaling Technology), c-MYC (9402, Cell Signaling Technology), c-MYC K158/K163Me (custom made, project code 2ZG1940M, Thermo Fisher Scientific), Cyclin D1 (2978, Cell Signaling Technology), FLAG-M2 (F1804, Sigma-Aldrich), GAPDH (5174, Cell Signaling Technology), GFP (2956, Cell Signaling Technology), GST-Tag (2625, Cell Signaling Technology), HSP90 (Sc13119, Santa Cruz Biotechnology), KLF4 (12173, Cell Signaling Technology), NANOG (4903, Cell Signaling Technology), OCT4 (2750, Cell Signaling Technology), polyHistidine (H1029, Sigma-Aldrich), SMYD3 (12859, Cell Signaling Technology), SOX2 (2748, Cell Signaling Technology), and SOX9 (82630, Cell Signaling Technology). Rabbit IgG HRP and Mouse IgG HRP (1706515 and 1706516, Bio-Rad Laboratories, respectively) were used as secondary antibodies and revealed using the Clarity Max Western ECL Substrate (1705062, Bio-Rad Laboratories).

For immunizing peptide-blocking experiments, the customized c-MYC K158/K163Me antibody was incubated overnight at 4 °C with or without the immunizing dimethyl K158/163Me peptide (23 µg/ml). The ratio between the c-MYC K158/K163Me antibody and the dimethyl K158/163me peptide was 1:5.

### Immuno dot-blot

Immuno-dot blot analysis was performed to validate the specificity of the affinity-purified c-MYC K158/K163Me antibody (custom-made, project code 2ZG1940M, Thermo Fisher Scientific). Decreasing doses (25-5-1-0,1-0,01 ng/μL) of the immunizing control (unmethylated) and dimethyl K158/163me peptides were dotted in triplicate onto a 0,2 µm nitrocellulose membrane (1620112, Bio-Rad Laboratories). Next, the membrane was incubated with the affinity-purified c-MYC K158/K163Me antibody (2ZG1940M, Thermo Fisher Scientific, 1:500 dilution). Rabbit IgG HRP (NA934V, GE Healthcare) was used as a secondary antibody and revealed using the Clarity Max Western ECL Substrate (1705062, Bio-Rad Laboratories) according to the manufacturer’s instructions.

### Immunofluorescence

Tissue sections and PDTOs were deparaffinized, fixed in 4% paraformaldehyde, permeabilized with Proteinase K (P6556, Sigma-Aldrich), and blocked with 3% BSA. Cells were seeded on glass coverslips, fixed in 4% paraformaldehyde, and permeabilized using 0.01–0.1% Triton X-100. Coverslips were incubated with the indicated primary antibodies and then with Alexa Fluor 488 (A-11094, Thermo Fisher Scientific) and Alexa Fluor 647 (A-32728, Thermo Fisher Scientific) secondary antibodies; nuclei were counterstained using DAPI (D9542, Sigma-Aldrich). Slides were sealed using ProLong^TM^ Diamond Antifade Mountant (P36961, Thermo Fisher Scientific). Images were acquired using a Zeiss Axio Observer fluorescence microscope (Carl Zeiss). Primary antibodies used: SMYD3 (12859, Cell Signaling Technology, 1:100 dilution), CD44 (3570, Cell Signaling Technology, 1:200 dilution), and c-MYC K158/K163Me (custom made, project code 2ZG1940M, Thermo Fisher Scientific, 1:100 dilution), FITC-conjugated CD44 (75122, Cell Signaling Technology, 1:100 dilution), FITC-conjugated EpCAM (MA1-10197, Thermo Fisher Scientific, 1:50 dilution), PE-conjugated CD133 (12-1338-42, Thermo Fisher Scientific, 1:100 dilution).

### Immunohistochemistry

Tissue specimens were fixed in 4% buffered formalin and embedded in paraffin. Sequential sections (4 μm) were cut, stained with hematoxylin and eosin (H&E), and used for morphological studies and immunohistochemical analysis. Sections were dewaxed and rehydrated in dH_2_O. Endogenous peroxidase activity was blocked by incubation in 3% hydrogen peroxide for 10 min. Then, sections were mounted on Apex Bond IHC Slides (3800040, Leica Biosystems) and used for immunohistochemical analysis. Immunohistochemical staining procedures were carried out on a BOND III automated immunostainer (Leica Biosystems), from deparaffinization to counterstaining with hematoxylin, using the Bond Polymer Refine Detection Kit (DS9800, Leica Biosystems). Then, sections were incubated overnight with FLEX Monoclonal Ki67 Antigen, Clone MIB-1 (GA626, Agilent Dako) or c-MYC K158/K163Me (custom-made, project code 2ZG1940M, Thermo Fisher Scientific) as a primary antibody. Antigen retrieval was performed using the BOND Epitope Retrieval Solution 2, a ready-to-use EDTA-based pH 9 reagent (AR9640, Leica Biosystems). Negative controls were used in each experiment. Images were acquired using a Zeiss Axio Observer Z1 optical microscope (Carl Zeiss).

### In silico clustering of the P-proteins involved in stemness-related processes

In silico clustering of the P-proteins involved in the stemness hallmark was performed by evaluating the relevant Reactome clusters (‘Developmental Biology’ pathways; Reactome Id: R-HSA-1266738.14) (https://reactome.org). At the time of analysis (February 2023), 64 P-proteins were identified as effectors of different pathways (Transcriptional regulation of pluripotent stem cells, Id: R-HSA-452723.4; Signaling by NODAL, Id: R-HSA-1181150.3; Activation of HOX genes during differentiation Id: R-HSA-5619507.5; Keratinization, Id: R-HSA-6805567.5) comprised in the ‘Developmental Biology’ Reactome cluster. We analyzed in silico all these 64 P-proteins, reporting P-tripeptide matches and positions, gene names, protein names, lengths, functions, and Reactome IDs supplied in the UniProt database. We further validated in cellulo the ability of selected candidates to interact with SMYD3 based on their oncogenic relevance.

### In silico P-tripeptide screening

The screening of the P1-P19 tripeptides was performed as previously described.^12,19^ Briefly, we took advantage of a library of rare tripeptides (P1-P19) to test their in vitro binding affinity to SMYD3. Subsequently, we used these tripeptides as in silico probes to detect all human proteins (termed P-proteins) containing them. To this end, we screened the human proteome using the UniProt Peptide Search tool (https://www.uniprot.org/peptidesearch). A total of 169,671 human proteins reported in the UniProt database at the time of the analysis (December 2018) were searched and mapped for each P-tripeptide, and 8,650 proteins (reviewed; UniProt annotation score = 5) were found to contain at least one P-tripeptide and thus considered potential SMYD3 interactors.

### In silico prediction analysis of c-MYC relative surface accessibility

In the absence of a comprehensive knowledge of c-MYC three-dimensional structure, we assessed the relative surface accessibility of lysines 158 and 163 by performing an in silico prediction analysis with the NetSurfP 3.0 server (https://services.healthtech.dtu.dk/services/NetSurfP-3.0/). The NetSurfP3 server provides predictions of surface accessibility, secondary structure, disorder, and phi/psi dihedral angles of amino acids in a given amino acid sequence.

### In vitro methylation assay

A luminometric methylation assay, MTase-Glo^TM^ Methyltransferase Assay (V7601, Promega), was performed to assess the methylation activity of SMYD3. Briefly, a SMYD3-GST active protein (500 ng, S348-380CG SignalChem) was assayed in a methylation reaction buffer (20 mM Tris (pH 8), 100 mM NaCl, 1 mM EDTA, 3 mM MgCl2, 100 μg/mL BSA, 1 mM DTT) with 20 μM SAM, and 500 ng of WT or mutant c-MYC human recombinant protein (untagged human full-length c-Myc protein ab169901 Abcam, GST-c-MYC, GST-c-MYC-K158A, and GST-c-MYC-K163A) in a final volume of 20 μL. Histone H4 Peptide (1-21) (650 ng, H13-58 SignalChem) was used as a positive control. The reaction was incubated for 2 h at 37 °C. Then, 5 μL of 5x MTase-Glo™ reagent was added to convert SAH to ADP. Next, MTase-Glo™ Detection Solution was added to convert ADP to ATP, which was determined by a luciferase/luciferin reaction. The generated luminescence was measured using a SPECTROstar Omega luminometer (BMG LABTECH). Each data point was collected in triplicate.

### In vitro pull-down assay

Untagged human full-length c-MYC protein expressed in E. coli (ab169901, Abcam) was incubated with HIS-SMYD3 fusion protein. The HSP90C-GST fusion protein was used as a positive control. Proteins were incubated for 1 h at 4 °C on a rocking platform for in vitro binding. Recombinant proteins were precipitated with Dynabeads HIS-Tag Isolation and Pulldown (10104D, Thermo Fisher Scientific) according to the manufacturer’s instructions, then washed extensively in buffer A (20 mM Tris-HCl pH 8, 150 mM KCl, 5 mM MgCl2, 0.2 mM EDTA, 10% glycerol, 0.1% NP-40) containing protease inhibitors (A32965, Thermo Fisher Scientific) and 1 mM DTT. Recombinant FLAG c-MYC fragments, expressed in mammalian cell lines (generated as reported in the “Recombinant protein expression/purification” section), were incubated with HIS-SMYD3 fusion protein. In vitro binding was performed as described above. Then, precipitates were resolved on 10% SDS-PAGE and analyzed by immunoblotting. Primary antibodies used: polyHistidine (H1029, Sigma-Aldrich), c-MYC (9402, Cell Signaling Technology), GST-Tag (2625, Cell Signaling Technology), and FLAG-M2 (F1804, Sigma-Aldrich). Rabbit IgG HRP and Mouse IgG HRP (1706515 and 1706516, Bio-Rad Laboratories, respectively) were used as secondary antibodies and revealed using the Clarity Max Western ECL Substrate (1705062, Bio-Rad Laboratories).

For the competition assay, 500 ng of HIS-SMYD3 recombinant protein and 200 ng of untagged human full-length c-MYC protein (ab169901, Abcam) were incubated for 1 h at 4 °C on a rocking platform in the presence of escalating doses (0, 1, 5, 25, 125 µM) of the purified P14, P14ext1, and P14ext2 peptides, respectively. Bound proteins were precipitated and resolved as described above.

### In vivo experiments

During the study, each mouse was given drinking water ad libitum and a complete pellet diet. Health monitoring was performed daily for all animals. Mice were monitored daily for clinical signs and mortality, and their body weight was assessed every 2–3 days. All animal-involving procedures were conducted in conformity with institutional guidelines, which comply with national and international laws and policies.

The HCT-116-xenografted mouse model was established at the Biogem Animal House in Ariano Irpino (Avellino, Italy) under the National Academy of Sciences guidelines.

Figure 6a–d. 10 × 10^6^ WT (*n* = 12) or SMYD3-KO (*n* = 12) HCT-116 cells were injected subcutaneously into the flanks (0.2 mL per flank, serum-free DMEM culture medium) of 5 to 6-week-old CD1 immunodeficient female nude mice (Charles River Laboratories International, Inc.). Throughout the study (16 days), tumor volume was measured every 2–3 days using the following formula: volume (mm^3^) = (width)^2^ x length x 0.5 with Mitutoyo forceps.

Figure 6e–h. 10 × 10^6^ WT HCT-116 cells were injected subcutaneously into the flanks (0.2 mL per flank, serum-free DMEM culture medium) of 5 to 6-week-old CD1 immunodeficient female nude mice (Charles River Laboratories International, Inc.) (*n* = 24). When tumor volume reached 100 mm^3^, mice were randomized to two treatment groups. Mice were treated for 12 days with 10 mg/kg of EM127@HSA dissolved in physiological solution administered daily by intraperitoneal injection (*n* = 12) or the vehicle (physiological solution) alone administered daily by intraperitoneal injection (n = 12). The tumor volume was measured every 2–3 days using the following formula: volume (mm^3^) = (width)^2^ x length x 0.5 with Mitutoyo forceps.

Figure 6i–l. The chemically-induced colitis-associated carcinogenesis mouse (AOM/DSS) model was established at the IRCCS “S. De Bellis” Animal House in Castellana Grotte (Bari, Italy). C57BL/6 mice (n = 24) were injected intraperitoneally with 12 mg/kg of AOM. Then, 2% DSS was given in their drinking water over a week, followed by two weeks of regular water. This cycle was repeated three times. Two weeks after the last round of DSS, mice were treated for 12 days with 10 mg/kg of EM127@HSA dissolved in physiological solution administered daily by intraperitoneal injection (n = 12) or the vehicle (physiological solution) alone administered daily by intraperitoneal injection (n = 12). For AOM/DSS–treated animals, welfare was recorded every 2^nd^ day during the DSS treatment regimen using a customized score sheet for body weight, appearance and behavior, rectal bleeding, and rectal prolapse. Assessment of appearance and behavior included evaluation of overall conditions, activity, movement, and facial expression of pain. Blood in feces was recorded as rectal bleeding.

To establish orthotopic colorectal cancer liver metastasis models in CD-1 female nude mice, three independent set-up experiments were performed. Cells were intrahepatically or intraperitoneally injected into CD1-nude female mice, and the most common sites of CRC metastasis, such as the liver and peritoneum, were observed for tumor formation.

Figure 7b–f. 3 × 10^6^ WT-luc (*n* = 7) or SMYD3-KO-luc (n = 7) HCT-116 cells were injected intrahepatically (0.05 mL, serum-free DMEM culture medium) into 5 to 6-week-old CD1 immunodeficient female nude mice (Charles River Laboratories International, Inc.). Throughout the study (28 days), intrahepatic tumor growth was evaluated weekly by IVIS measurement.

Figure 8a–e. 3 × 10^6^ patient-derived CRC-SCs (#C109 cells were injected intraperitoneally (0,05 mL, serum-free DMEM F12 advanced culture medium) into 5 to 6-week-old CD1 immunodeficient female nude mice (Charles River Laboratories International, Inc.) (*n* = 10). 24 h after cell inoculation, mice were treated for 7 days with 10 mg/kg of EM127@HSA dissolved in physiological solution administered daily by intraperitoneal injection (*n* = 5) or the vehicle (physiological solution) alone administered daily by intraperitoneal injection (n = 5). Animals were clinically observed and sacrificed after 49 days from cell inoculation.

Figure 8f–j. 3 × 10^6^ patient-derived CRC-SCs (#C108 cells were injected intraperitoneally (0.05 mL, serum-free DMEM F12 advanced culture medium) into 5 to 6-week-old CD1 immunodeficient female nude mice (Charles River Laboratories International, Inc.) (*n* = 10). After 28 days from cell inoculation, mice were treated for 12 days with 10 mg/kg of EM127@HSA dissolved in physiological solution administered daily by intraperitoneal injection (*n* = 5) or the vehicle (physiological solution) alone administered daily by intraperitoneal injection (n = 5). Animals were clinically observed and sacrificed after 40 days from cell inoculation.

Figure 9f, g. 10 × 10^6^ WT HCT-116 cells were injected subcutaneously into the flanks (0.2 mL per flank, serum-free DMEM culture medium) of 5 to 6-week-old CD1 immunodeficient female nude mice (Charles River Laboratories International, Inc.) (*n* = 24). When tumor volume reached a measurable size, mice were randomized to four treatment groups. Mice were treated for 12 days with 10 mg/kg of EM127@HSA dissolved in physiological solution administered daily by intraperitoneal injection (*n* = 6), 5-fluorouracil (25 mg/kg) dissolved in physiological solution administered every 3 days by intraperitoneal injection (*n* = 6), both (*n* = 6), or the vehicle (physiological solution) alone (n = 6). Tumor volume was measured every 2–3 days using the following formula: volume (mm^3^) = (width)^2^ x length x 0.5 with Mitutoyo forceps.

Figure 9h, i. 3 × 10^6^ patient-derived CRC-SC cells were injected subcutaneously into the flanks (0.2 mL per flank, serum-free DMEM culture medium) of 5 to 6-week-old CD1 immunodeficient female nude mice (Charles River Laboratories International, Inc.) (*n* = 24). When tumor volume reached a measurable size, mice were randomized to four treatment groups. Mice were treated for 12 days with 10 mg/kg of EM127@HSA dissolved in physiological solution administered daily by intraperitoneal injection (*n* = 6), 5-fluorouracil (25 mg/kg) dissolved in physiological solution administered every 3 days by intraperitoneal injection (*n* = 6), both (*n* = 6), or the vehicle (physiological solution) alone (n = 6). Tumor volume was measured every 2–3 days using the following formula: volume (mm^3^) = (width)^2^ x length x 0.5 with Mitutoyo forceps.

At the end of each study, mice were sacrificed by cervical dislocation, and tumor masses were photographed and collected. All tissues were fixed overnight in 10% formalin and embedded in paraffin.

### Ki67 assay

Patient-derived CRC-SCs were cultured in ultra-low attachment 6-well plates (3471, Costar Corning) at 37 °C, 5% CO_2_, with complete medium. Cells were treated or not with EM127 or 5-fluorouracil. 1 × 10^5^ cells/plate were collected, resuspended in 1X PBS, and analyzed to determine the percentage of proliferating cells based on Ki67 expression using the Muse Ki67 Proliferation Kit (MCH100114, Merck Millipore) according to the manufacturer’s instructions. Flow cytometry was performed using the Guava Muse Cell Analyzer (Merck Millipore). Each analysis was performed by evaluating at least 2000 events, using the assay-specific software module included in the Guava Muse Cell Analyzer instrument (Merck Millipore).

### Live and dead assay

HCT-116 tumorspheres and patient-derived tumor organoids (PDTOs) were stained using the LIVE/DEAD® Cell Imaging Kit (R37601, Thermo Fisher Scientific) according to the manufacturer’s instructions. Specifically, live cells were stained green (Calcein AM), and dead cells were stained red (BOBO-3 Iodide). Digital image acquisition was carried out with a Zeiss Axio Observer fluorescence microscope (Carl Zeiss) using a 10x magnification objective. Quantification of cell death induction in tumorspheres was performed by analyzing the intensity of the red signal using ZEN blue software 3.3 version (Carl Zeiss).

### Mass spectrometry analysis

Mass spectrometry analysis was conducted by the Cogentech srl service. Gel bands underwent reduction using dithiothreitol (DTT) (A39255, Thermo Fisher Scientific), alkylation using iodoacetamide (IAA) (A39271, Thermo Fisher Scientific), and double enzymatic digestion using the endoproteinases AspN (V1621, Promega), which selectively cleaves protein and peptide bonds N-terminal to aspartic acid residues, and Glu-C (V1651, Promega), which specifically cleaves at the C-terminus of either aspartic or glutamic acid residues. Then, the flow-through was treated with C18 Spin Tips & Columns (84850, Thermo Fisher Scientific) for desalting. The samples and the desalted flow-through were further purified with SP3 and then analyzed by nLC-ESI-MS/MS on a Q Exactive HF mass spectrometer (Thermo Fisher Scientific) with a 45-minute gradient. Samples were run in technical duplicate, in a positive mode with electrospray ionization. Data acquisition and processing were performed with Analyst TF (version 1.7.1, AB SCIEX). c-MYC was identified with a sequence coverage of 72% after the two analyses. Data were analyzed using the Proteome Discoverer, Mascot, and Scaffold setting software. The parameter settings of data processing were as follows: DataBase = Uniprot_CP_Human_2020 (Database Uniprot_cp_Human c-MYC; Human sequence, Accession Numbers: P01106-2); Enzymes = AspN (cuts at N-term of D) and Glu-C (cuts at C-term of D and also on E); Modifications = Acetyl (Protein N-term), Carbamidomethyl (C), Oxidation (M), Phosphorylation (STY); Peptide Thresholds: 95.0% minimum; Protein Thresholds: 99.0% minimum; 2 peptides/protein minimum.

### Methylene blue staining

Methylene blue was used to stain and score the number of tumors. The colon was excised from the anus to the cecum, rinsed, and flushed with ice-cold PBS to remove any intestinal contents and then slit open longitudinally. Next, the colon was fixed flat between two PBS-soaked filter papers held together with staples. The flat-fixed colon was then stored in 10% neutral buffered formalin for at least 24 h before staining and removed from the filter paper. Any remaining fat on the muscularis side of the colon was carefully removed with forceps, and then the colon was transferred to a new beaker filled with distilled water. After 1 min, the colon was transferred to another beaker containing a 70% ethanol solution for 45 min. Next, the colon was moved to a beaker containing 0.2% methylene blue (M9140, Sigma-Aldrich), stained for 5–10 s, and transferred to a new beaker filled with distilled water to wash off excess methylene blue. Colon specimens were mounted on a microscope slide and observed under an inverted microscope (Primovert, Carl Zeiss) equipped with a digital color camera. Once scored, specimens were paraffin-embedded.

### Motility and invasion assay

Cell migration and invasion were assessed as described by Justus et al.^87^ Briefly, 1 × 10^4^ patients-derived CRC-SCs were treated or not for 16 h with EM127. Next, cells were suspended in 200 μL of non-supplemented stem cell medium and plated into the upper wells of Matrigel-coated (for invasion) or non-coated (for migration) Boyden chambers containing 8 μm diameter polycarbonate membranes (CLS3422-48EA, Costar Corning). Lower wells contained 600 μL of stem cell medium supplemented with 20 ng/mL EGF (E9644, Sigma-Aldrich), 10 ng/mL basic HbFGF (F0291, Sigma-Aldrich), and/or EM127 (10 μM). Cells were cultured in these conditions for 16 h. Migrated and invading cells were fixed, stained with DAPI (D9542, Sigma-Aldrich) in 1X PBS, 1% NP40 for 5 min, and counted under a fluorescence microscope.

### Multiple sequence alignment

The protein sequences of human c-MYC and homologous proteins from other species were aligned with the latest version of Clustal Omega (http://www.clustal.org/, accessed on June 2023), an online program based on phylogenetic guide trees and probabilistic models of the evolutionary changes that have occurred in a set of related sequences to generate alignments between three or more sequences with high accuracy.

### Patient-derived organoids

Patient-derived organoids were generated from patients’ biopsies according to the Gentle Cell Dissociation Reagent product information sheet (100-0485, StemCell Technology). Briefly, 4 × 10^3^ dissociated crypts were mixed into a DMEM/F-12 (31330038, Gibco) and Matrigel Matrix (356234, Costar Corning) solution at a 1:2 volume ratio in 24-well TC-treated plates (3526, Costar Corning) at 37 °C until Matrigel solidification. Then, 750 μL of IntestiCult™-SF Organoid Growth Medium Human (100-0340, StemCell Technologies) was added on top of the Matrigel dome. Patient-derived organoids were passaged every 7–10 days.

### Quantification and statistical analysis

The statistical significance of the results was analyzed using Student’s t-test, and *P < 0.05 was considered statistically significant.

### Quantitative real-time PCR

Total RNA was isolated using the PureLink RNA Mini Kit (12183018 A, Thermo Fisher Scientific) according to the manufacturer’s instructions. RNA purity was confirmed by spectrophotometry, and RNA integrity by agarose gel electrophoresis. cDNA was synthesized by retro-transcribing 1 μg of total RNA using the iScript cDNA Synthesis Kit (1708890, Bio-Rad Laboratories) according to the manufacturer’s instructions. Real-time PCR primers were designed using Primer Express software. PCR assays were performed in 96-well optical reaction plates using a QuantStudio 3 machine (Thermo Fisher Scientific). Each assay was carried out in triplicate wells. Baseline values of amplification plots were set automatically and threshold values were kept constant to obtain normalized cycle times and linear regression data. The following reaction mixture per well was used: 5 μL of PowerUp SYBR Green Master Mix (A25741, Thermo Fisher Scientific), 1.1 μL of primers at a final concentration of 100 nM, 2.4 μL of RNAse-free water, 1.5 μL of cDNA. For all experiments, the following PCR conditions were used: denaturation at 95 °C for 30 seconds, followed by 40 cycles at 95 °C for 15 seconds and 60 °C for 30 seconds. Primer sequences are available upon request. Quantitative normalization of cDNA in each sample was performed using *GAPDH* as an internal control. Relative quantification was done using the ddCT method.

### Recombinant protein expression/purification

BL21 (DE3) competent E. coli cells (C2527I), transformed with different constructs, were grown in Luria broth medium (10855021, Gibco) with ampicillin (A9518, Sigma-Aldrich) or kanamycin (K1377, Sigma-Aldrich) and induced with 1 mM IPTG when they reached the optical density of 0.6 (A600) at 37 °C for 3 h. Cells were then collected by centrifugation, and pellets were lysed with B-PER lysis buffer (78248, Thermo Fisher Scientific). The lysate was centrifuged at 20,000*×g* for 20 min at 4 °C. GST-fusion proteins were purified with a GST bulk kit (27457001, Cytiva) according to the manufacturer’s instructions. HIS-fusion proteins were purified with Dynabeads HIS-Tag Isolation and Pulldown (10104D, Thermo Fisher Scientific) according to the manufacturer’s instructions. GST-fusion proteins and HIS-fusion proteins were evaluated and quantified by SDS-PAGE.

The p3xFLAG CMV14 c-MYC-WT, p3xFLAG CMV14 c-MYC-M1 (aa 1–375), p3xFLAG CMV14 c-MYC-M2 (aa 266–454), and p3xFLAG CMV14 c-MYC-M3 (aa 1–165) constructs were transfected in HEK-293 cells for 48 h using Lipofectamine 3000 (L3000015, Thermo Fisher Scientific) according to the manufacturer’s instructions. Cells were collected and lysed with IP buffer (87787, Thermo Fisher Scientific), and the supernatant (cleared lysate) was applied over Pierce Anti-DYKDDDDK Affinity Resin (A36801, Thermo Fisher Scientific) according to the manufacturer’s instructions. Proteins were evaluated and quantified by SDS-PAGE.

### Tumorsphere formation efficiency assay

Cells (500/well) were seeded into 24-well ultra-low attachment cluster plates (3473, Costar Corning) and cultured in Advanced DMEM/F-12 (12634010, Gibco) supplemented with 1% L-glutamine (25030081, Gibco), 100 IU/mL penicillin-streptomycin (15140122, Gibco), 0.6% glucose solution (G8769, Sigma-Aldrich), B-27 (12587010, Gibco), N-2 (17502048, Gibco), 10 ng/mL HbFGF (F0291, Sigma-Aldrich), 20 ng/mL EGF (E9644, Sigma-Aldrich). After two days in culture, spheres were photographed, counted, and analyzed daily.

### TUNEL assay

The TUNEL assay was performed using the Click-iT Plus TUNEL Assay kit (C10618, Thermo Fisher Scientific) according to the manufacturer’s instructions. Briefly, tissue sections were deparaffinized, fixed in 4% paraformaldehyde, and permeabilized with Proteinase K (P6556, Sigma-Aldrich). Sections were then soaked in TdT reaction buffer at 37 °C for 10 min. Next, the TdT reaction buffer was discarded, and sections were incubated at 37 °C for 60 min with the TdT reaction mixture. Subsequently, sections were incubated at 37 °C for 30 min in the dark with the Click-iT Plus TUNEL reaction cocktail. Sections were then stained with DAPI solution (D9542, Sigma-Aldrich) and examined under a Zeiss Axio Observer fluorescence microscope (Carl Zeiss).

## Supplementary information

Supplementary Materials

## Data Availability

The data that support the results of this study are available from the corresponding author. All raw and processed RNA-seq data generated from HCT-116 cell lines have been deposited in the Gene Expression Omnibus (GEO) repository under accession number GSE264157. In addition, raw and processed RNA-seq data derived from patient-derived CRC-SCs are available in GEO under accession number GSE264227.
