# Supplementary material for: Tailoring a novel colorectal cancer stem cell-targeted therapy by inhibiting the SMYD3/c-MYC axis
Source: Signal Transduct Target Ther. 2025 Jun 30;10:206. doi: 10.1038/s41392-025-02290-z (PMC12209437; doi:10.1038/s41392-025-02290-z)
Supplement: Supplementary file 1 — Supplementary Materials [file 41392_2025_2290_MOESM1_ESM.docx]

Supplementary Materials for

Tailoring a novel colorectal cancer stem cell-targeted therapy by inhibiting the SMYD3/c-MYC axis

Martina Lepore Signorile, Elisabetta Di Nicola, Giovanna Forte, Paola Sanese, Candida Fasano, Vittoria Disciglio, Katia De Marco, Marialaura Latrofa, Loris De Cecco, Marica Ficorilli, Marta Lucchetta, Erica Torchia, Chiara Dossena, Giusy Bianco, Vito Spilotro, Claudia Ferroni, Nicoletta Labarile, Raffaele Armentano, Francesco Albano, Anna Mestice, Gianluigi Gigante, Valerio Lantone, Giuliano Lantone, Leonardo Vincenti, Alberto Del Rio, Greta Varchi, Valentina Grossi*, and Cristiano Simone*

*Correspondence to: cristianosimone73@gmail.com; valentina.grossi@irccsdebellis.it

**This PDF file includes:**

Figures. S1 to S9 (with figure legends below the pictures)

Tables S1 to S6 (with table legends above the tables)

**Other Supplementary Materials for this manuscript include the following:**

Uncropped western blots

Figure. S1.


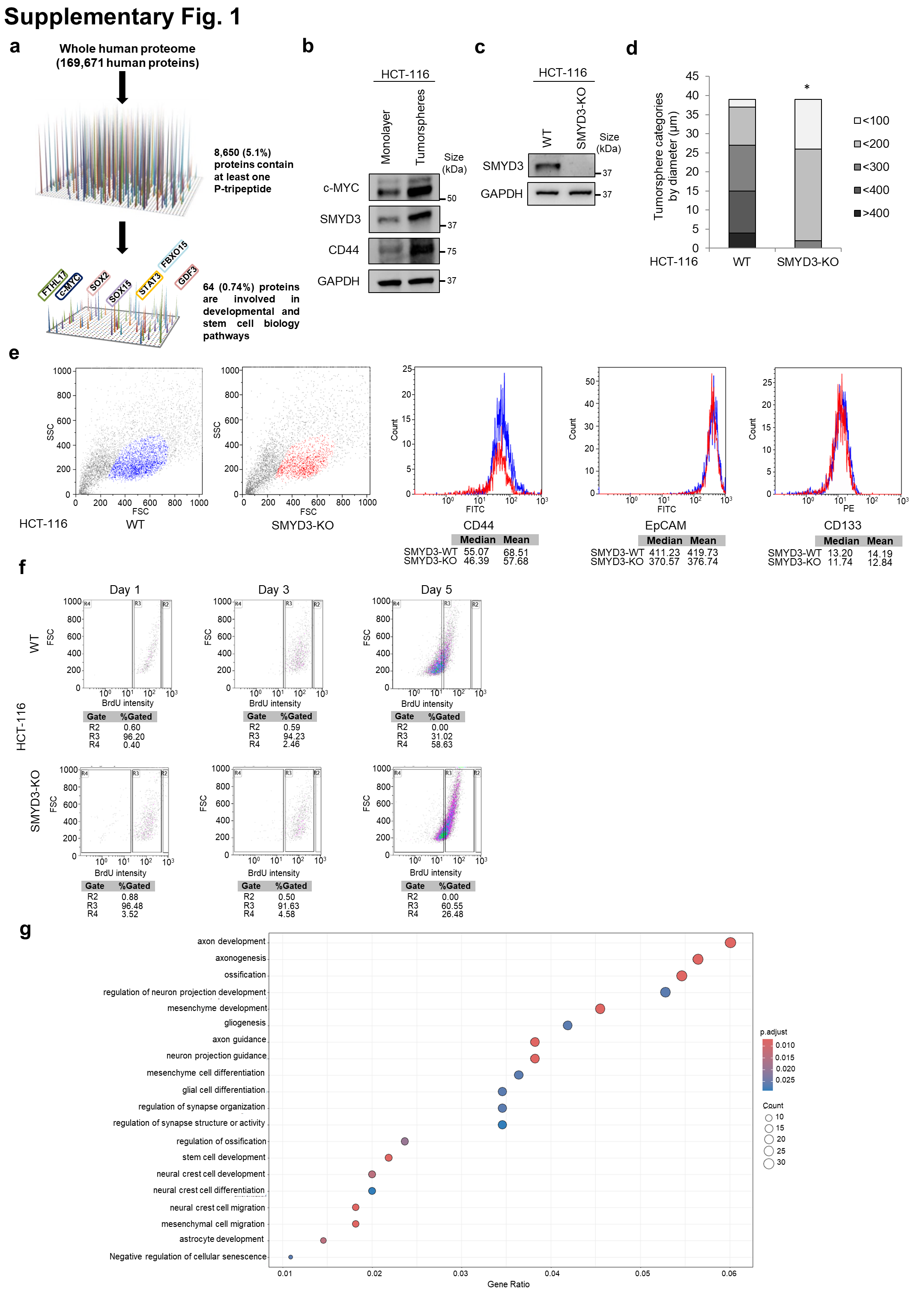


**Supplementary Fig. 1. Characterization of the stemness phenotype in WT and SMYD3-KO HCT-116 tumorspheres.** (**a**) Procedural scheme of the *in silico* P-tripeptide screening. Proteins were clustered based on their biological function as annotated in the corresponding UniProt entry, and the clustering was confirmed in the REACTOME (*https://reactome.org*) database. (**b**) Immunoblot analysis of SMYD3, c-MYC, and CD44 levels in HCT-116 cells grown as monolayer or in tumorsphere culture conditions. GAPDH was used as a loading control. (**c**) Immunoblot analysis of SMYD3 levels in WT and SMYD3-KO HCT-116 cells. GAPDH was used as a loading control. (**d**) Diameter of tumorspheres formed by WT and SMYD3-KO HCT-116 cells, as determined by tumorsphere formation assay. (**e**) Flow cytometry analysis of the CRC-SC markers CD44, EpCAM, and CD133 in WT (blue) and SMYD3-KO (red) HCT-116 tumorspheres. (**f**) Differential patterns of symmetric and asymmetric DNA distribution in WT and SMYD3-KO HCT-116 tumorspheres. Day 1 was the last day of BrdU labeling. **(g**) Dot plot of the top 20 ranked terms obtained from the Gene Ontology (GO) enrichment analysis. “Count” indicates the number of genes enriched in a GO term. “Gene ratio” indicates the percentage of enriched genes in the given GO term. *p<0.05 SMYD3-KO vs WT parental cells.

Figure. S2


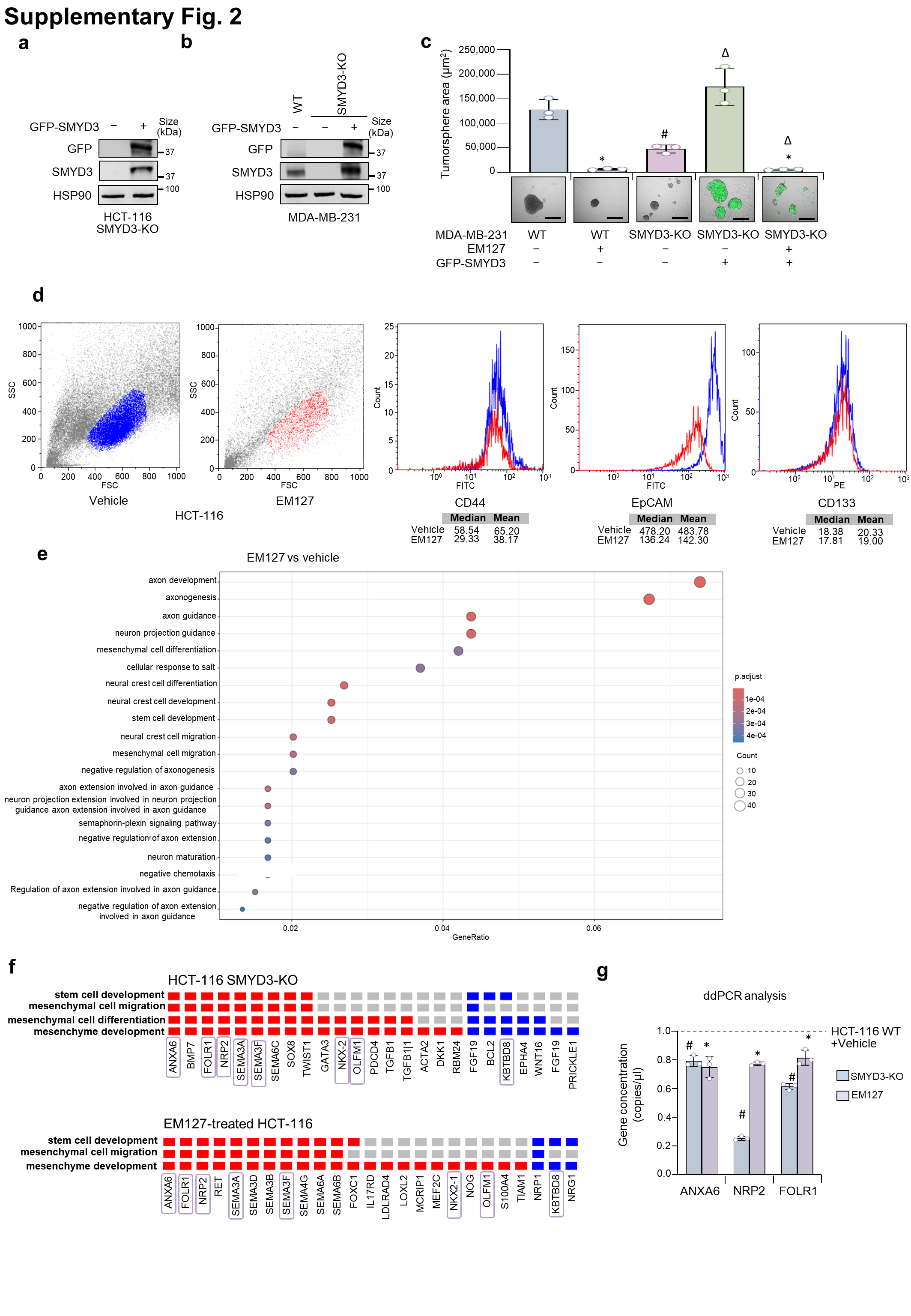
.

**Supplementary Fig. 2.** **Effect of SMYD3 pharmacological inhibition on HCT-116 cancer stemness properties.** (**a**) Immunoblot analysis of SMYD3 levels in HCT-116 SMYD3-KO tumorspheres transfected with a GFP-SMYD3 construct. HSP90 was used as a loading control. (**b**) Immunoblot analysis of SMYD3 and GFP levels in WT MDA-MB-231 and in SMYD3-KO MDA-MB-231 tumorspheres transfected with a GFP-SMYD3 construct. HSP90 was used as a loading control. (**c**) Tumorsphere formation assay of WT and SMYD3-KO MDA-MB-231 tumorspheres treated or not with EM127 (5 μM) for 72 hours. Where indicated, SMYD3 expression was reconstituted in SMYD3-KO tumorspheres with a GFP-SMYD3 construct. *p<0.05 EM127-treated vs untreated. #p<0.05 SMYD3-KO vs WT parental cells. Δ p<0.05 SMYD3-KO cells transfected with the GFP-SMYD3 construct vs untransfected SMYD3-KO cells. Scale bar: 200 μm. (**d**) Flow cytometry analysis of the CRC-SC markers CD44, EpCAM, and CD133 in WT HCT-116 tumorspheres treated (red) or not (blue) with EM127 (5 μM) for 24 hours. (**e**) Dot plot of the top 20 ranked terms obtained from the Gene Ontology (GO) enrichment analysis described in Figure 2g. “Count” indicates the number of genes enriched in a GO term. “Gene ratio” indicates the percentage of enriched genes in the given GO term. (**f**) Plots of relevant GO enrichment results. The plots depict deregulated genes (red, down-regulated; blue, up-regulated) involved in relevant GO terms (stem cell development, mesenchymal cell migration, mesenchymal cell differentiation, mesenchyme development). Genes boxed in light violet represent deregulated genes identified in both SMYD3-KO HCT-116 cells and EM127-treated WT HCT-116 cells. (**g**) ddPCR analysis of differentially expressed genes in both WT HCT-116 tumorspheres treated with EM127 (5 μM) for 24 hours and SMYD3-KO HCT-116 tumorspheres vs untreated WT HCT-116 tumorspheres. *p<0.05 treated vs untreated; #p<0.05 SMYD3-KO vs WT parental cells. Where applicable, data are expressed as means ± SD of 3 independent experiments.

Figure. S3.


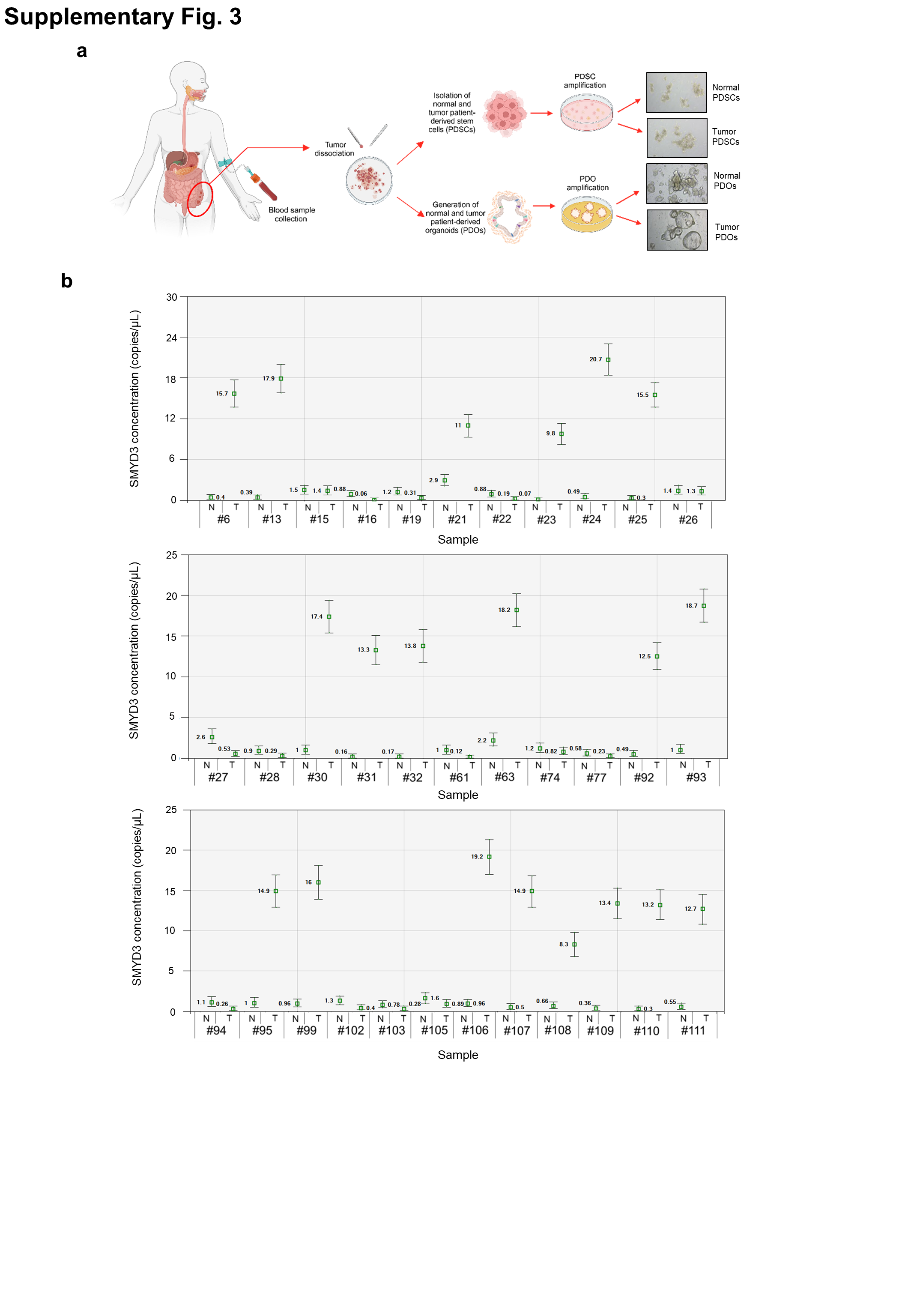


**Supplementary Figure 3. Analysis of SMYD3 overexpression in CRC patient-derived samples.** (**a**) Schematic diagram of the generation of CRC patient-derived normal and tumor stem cells and organoids. Created in Biorender. <https://BioRender.com/3zilqr8>. (**b**) ddPCR of SMYD3 levels in CRC patient-derived normal and tumor stem cells.

Figure. S4.


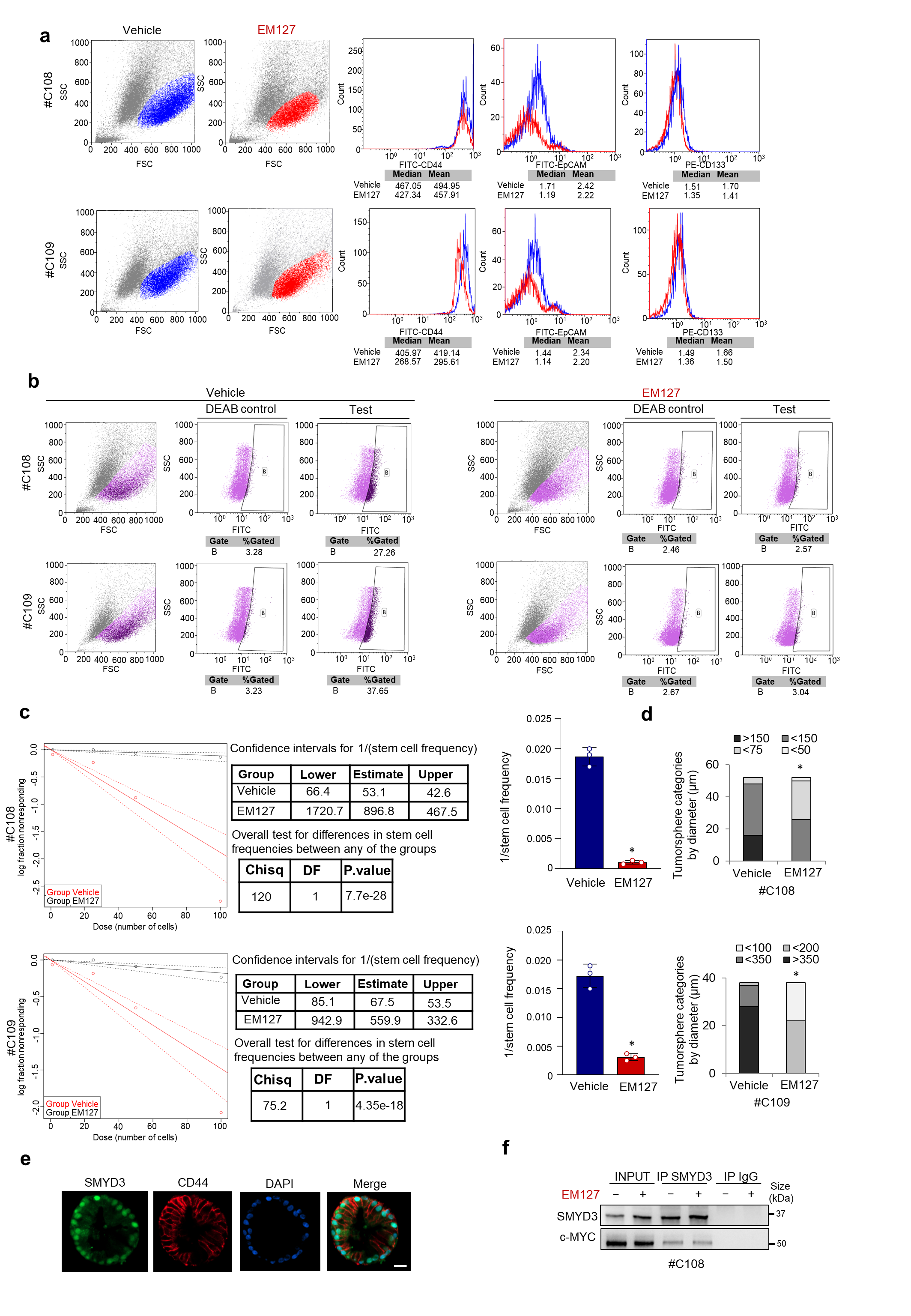


**Supplementary Figure 4. Characterization of the stemness phenotype in patient-derived CRC-SCs treated with EM127.** (**a**) Flow cytometry analysis of the CRC-SCs markers CD44, EpCAM, and CD133 in patient-derived CRC-SCs (#C108 and #C109) treated (red) or not (blue) with EM127 (10 μM) for 24 hours. (**b**) Flow-cytometry analysis of ALDH activity as determined using the Aldefluor assay system in patient-derived CRC-SCs (#C108 and #C109) treated or not with EM127 (10 μM) for 24 hours. Patient-derived CRC-SCs treated with the ALDH inhibitor DEAB were used as a negative control. (**c**) ELDA software analysis of clonogenic activity in patient-derived CRC-SCs (#C108 and #C109) treated (black) or not (red) with EM127 (10 μM). (**d**) Diameter of tumorspheres formed by patient-derived CRC-SCs (#C108 and #C109), as determined by tumorsphere formation assay. (**e**) Representative images of immunofluorescence staining of SMYD3 and CD44 in SMYD3-overexpressing PDTOs. Scale bar: 20 μm. (**f**) Immunoprecipitation assay of endogenous SMYD3 in patient-derived CRC-SCs treated or not with EM127 (10 μM) for 48 hours. Anti-IgGs were used as negative controls. *p<0.05 EM127-treated vs untreated. Where applicable, data are expressed as means ± SD of 3 independent experiments.

Figure. S5.


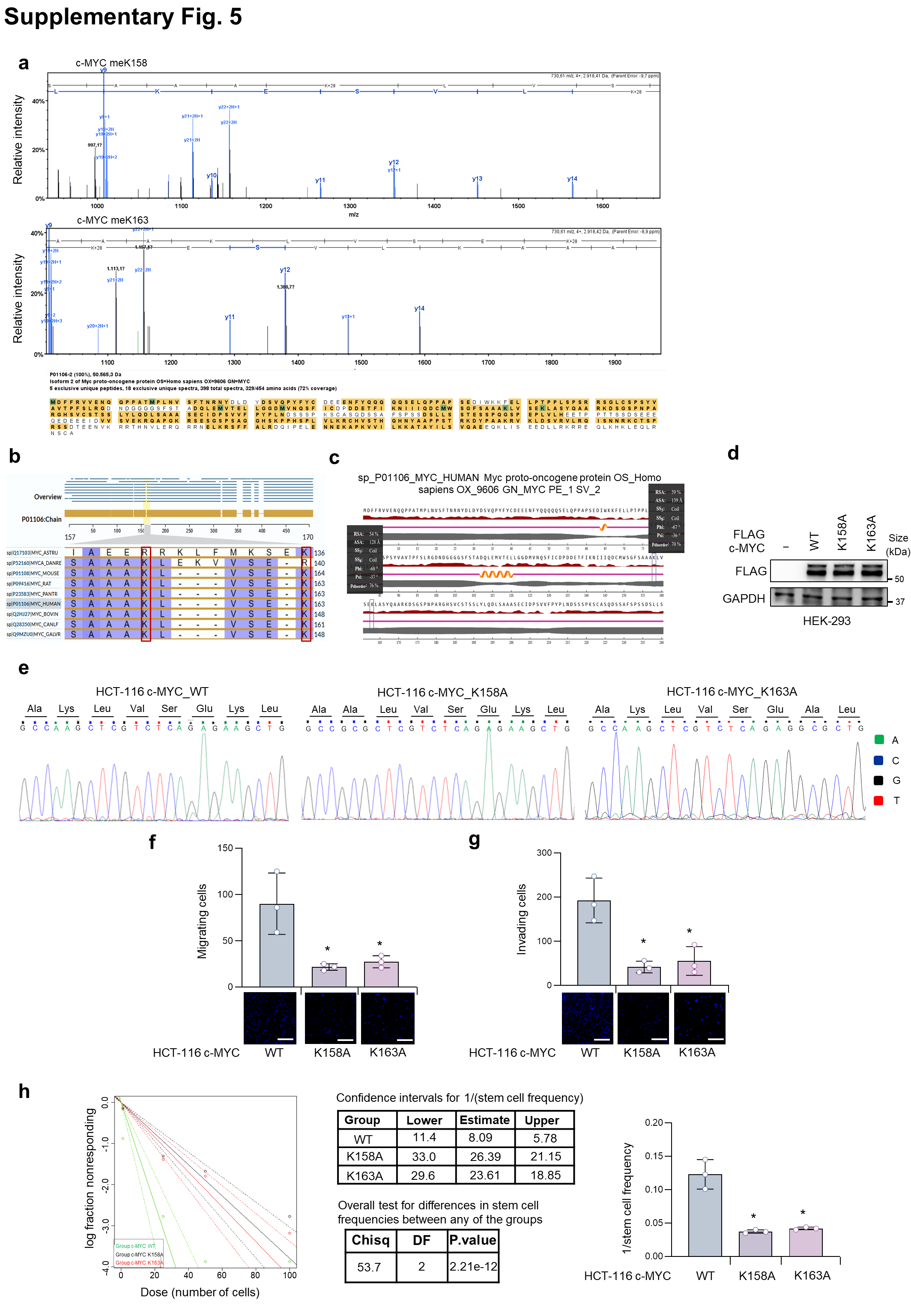


**Supplementary Figure 5.** **SMYD3 methylates c-MYC at K158 and K163.** (**a**) MS/MS spectra for peptides 147-172 including the methylated lysines K158 and K163, obtained by proteolytic digestion of SMYD3-methylated c-MYC with AspN and Glu-C. Sequence coverage map of c-MYC sequence (UniProt entry: P01106-2). (**b**) Multiple sequence alignment of human c-MYC and homologous proteins from other species. UniProt IDs are indicated on the left. K158 and K163 (red boxes) are located in highly conserved regions. ASTRU: Asterias rubens, DANRE: Danio rerio, PANTR: Pan troglodytes, CANLF: Canis lupus familiaris, GALVR: Galeopterus variegatus. (**c**) *In silico* analysis of the relative surface accessibility (RSA) of the c-MYC N-terminal region (240 residues) carried out using NetSurfP-3.0 server (https://services.healthtech.dtu.dk/services/NetSurfP-3.0/). Secondary structure motifs are differentially represented, with helix motifs in orange and coil motifs in pink. Disordered regions are indicated in grey, and the thickness of the line is proportional to the probability of a disordered residue. (**d**) Immunoblot analysis of FLAG c-MYC levels in HEK-293 cells transiently transfected with WT c-MYC, mutant c-MYC_K158A, or mutant c-MYC_K163A constructs. GAPDH was used as a loading control. (**e**) Sequencing electropherograms of genomic DNA from WT, c-MYC_K158A knock-in, and c-MYC_K163A knock-in HCT-116 cells confirming the substitutions. (**f,g**) Migratory (**f**) and invasive (**g**) ability of growth factor-starved WT, c-MYC_K158A knock-in, and c-MYC_K163A knock-in HCT-116 tumorspheres placed in the inner chamber of transwell plates for 16 hours. Migrating and invading cells were fixed and counted under a fluorescence microscope. Scale bar: 200 μm. (**h**) ELDA software analysis of clonogenic activity in WT (green), c-MYC_K158A knock-in (black), and c-MYC_K163A knock-in (red) HCT-116 tumorspheres. Where applicable, data are expressed as means ± SD of 3 independent experiments.

Figure. S6.


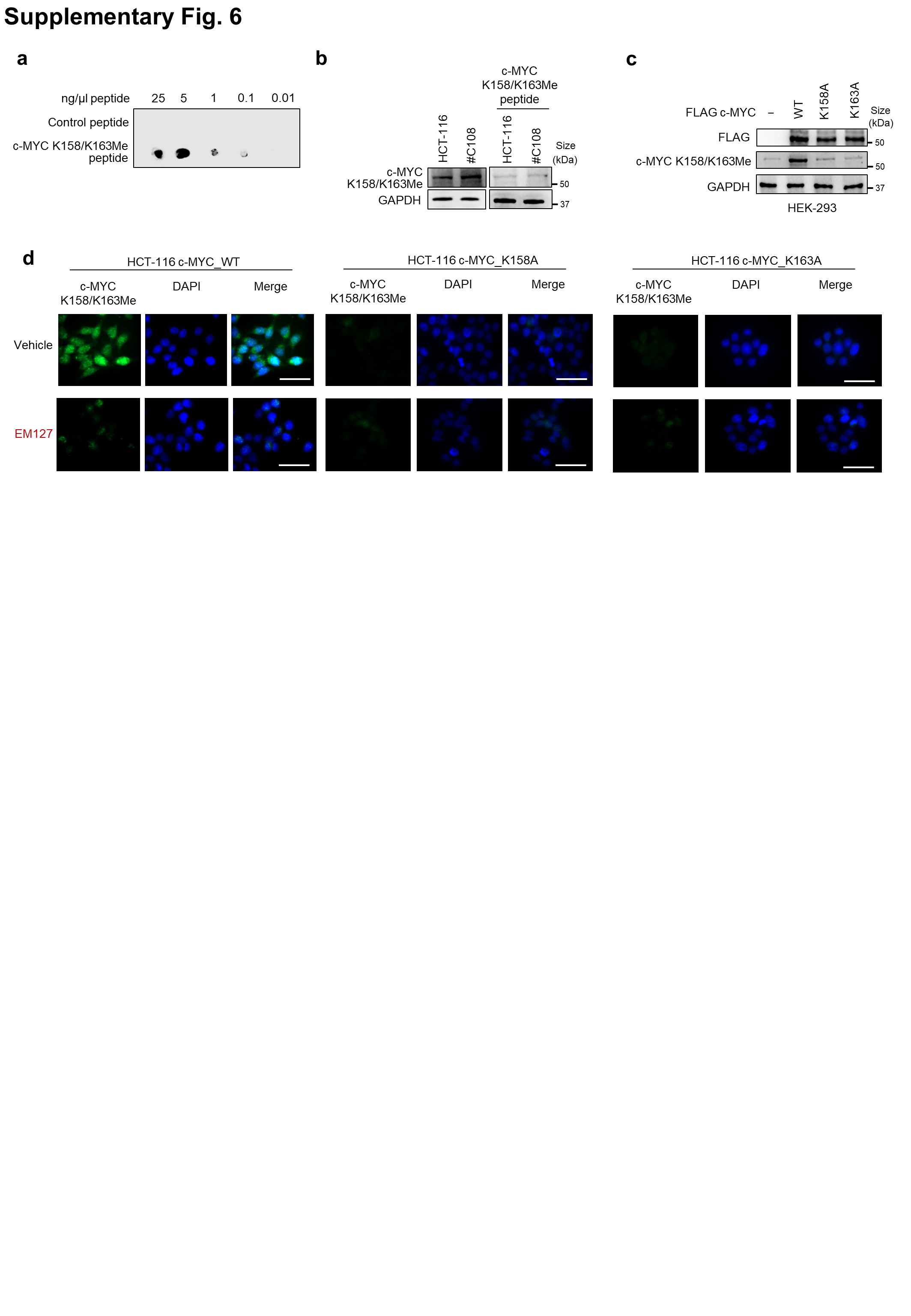


**Supplementary Figure 6. Characterization of the dimethyl c-MYC K158/K163** **custom antibody used in this study.** (**a**) Immuno dot-blot analysis with the affinity-purified c-MYC K158/K163Me antibody on serial dilutions of unmodified control (unmethylated) and dimethyl K158/163Me peptides. (**b**) Immunoblot analysis of c-MYC K158/K163Me levels in HCT-116 and patient-derived CRC-SC (#C108) in the absence and presence of a blocking dimethyl K158/163Me peptide (23 µg/ml). GAPDH was used as a loading control. (**c**) Immunoblot analysis of FLAG and c-MYC K158/K163Me levels in HEK-293 cells transiently transfected with WT c-MYC, mutant c-MYC_K158A, or mutant c-MYC_K163A constructs. GAPDH was used as a loading control. (**d**) Representative images of immunofluorescence staining of c-MYC K158/K163Me in WT, c-MYC_K158A knock-in, and c-MYC_K163A knock-in HCT-116 cells treated or not with EM127 (5 μM) for 48 hours. Scale bar: 50 μm.

Figure. S7.


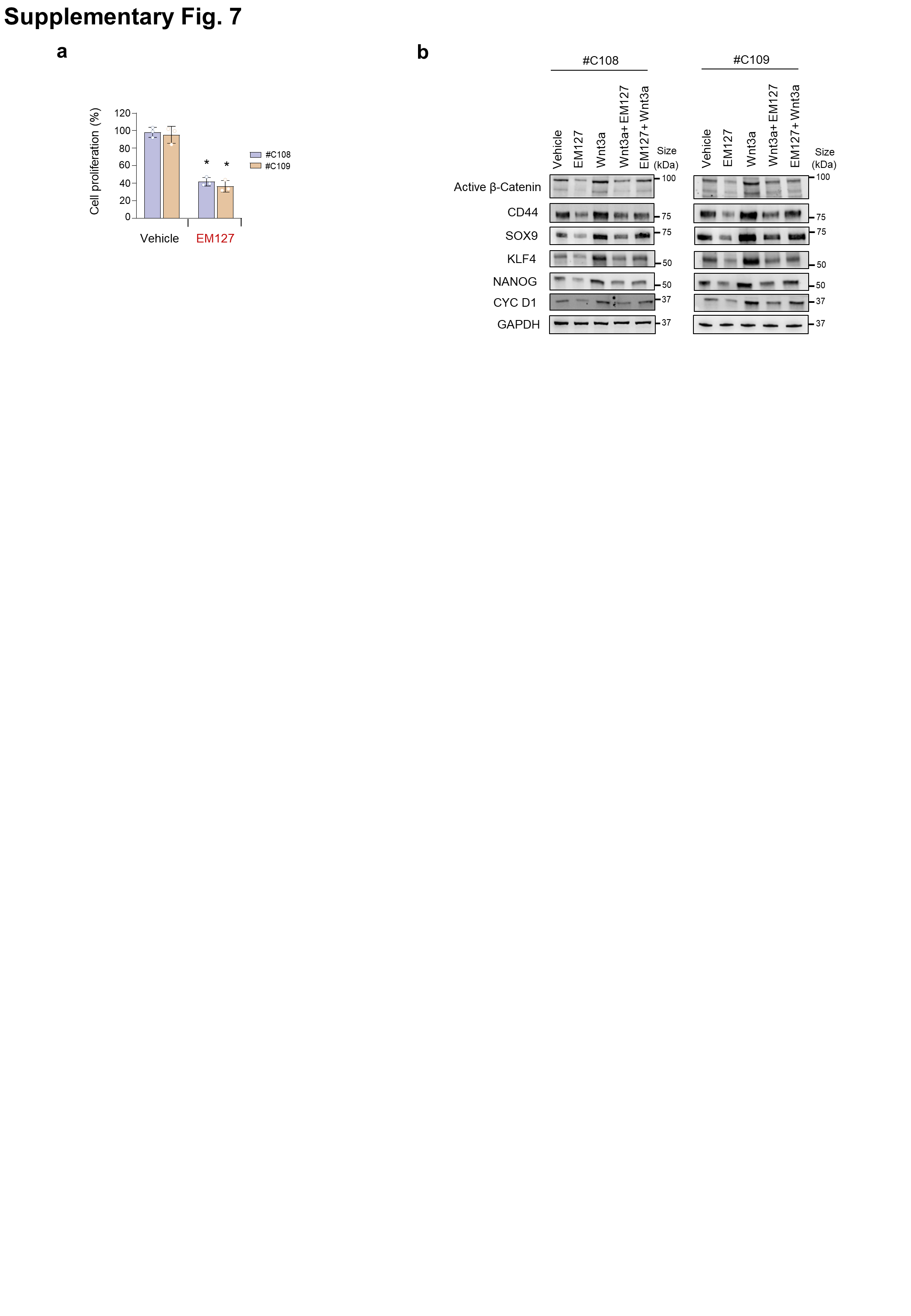


**Supplementary Figure 7. Effects of SMYD3 pharmacological inhibition on patient-derived CRC-SCs.** (**a**) Quantification of cell proliferation by CellTiter AQueous in patient-derived CRC-SCs treated or not with EM127 (10 μM) for 72 hours. *p<0.05 EM127-treated vs untreated. (**b**) Immunoblot analysis of c-MYC target gene products in patient-derived CRC-SCs treated with Wnt3a (50 ng) concomitantly or after treatment with EM127 (10 μM) for 24 hours. GAPDH was used as a loading control. Where applicable, data are expressed as means ± SD of 3 independent experiments.

Figure. S8.


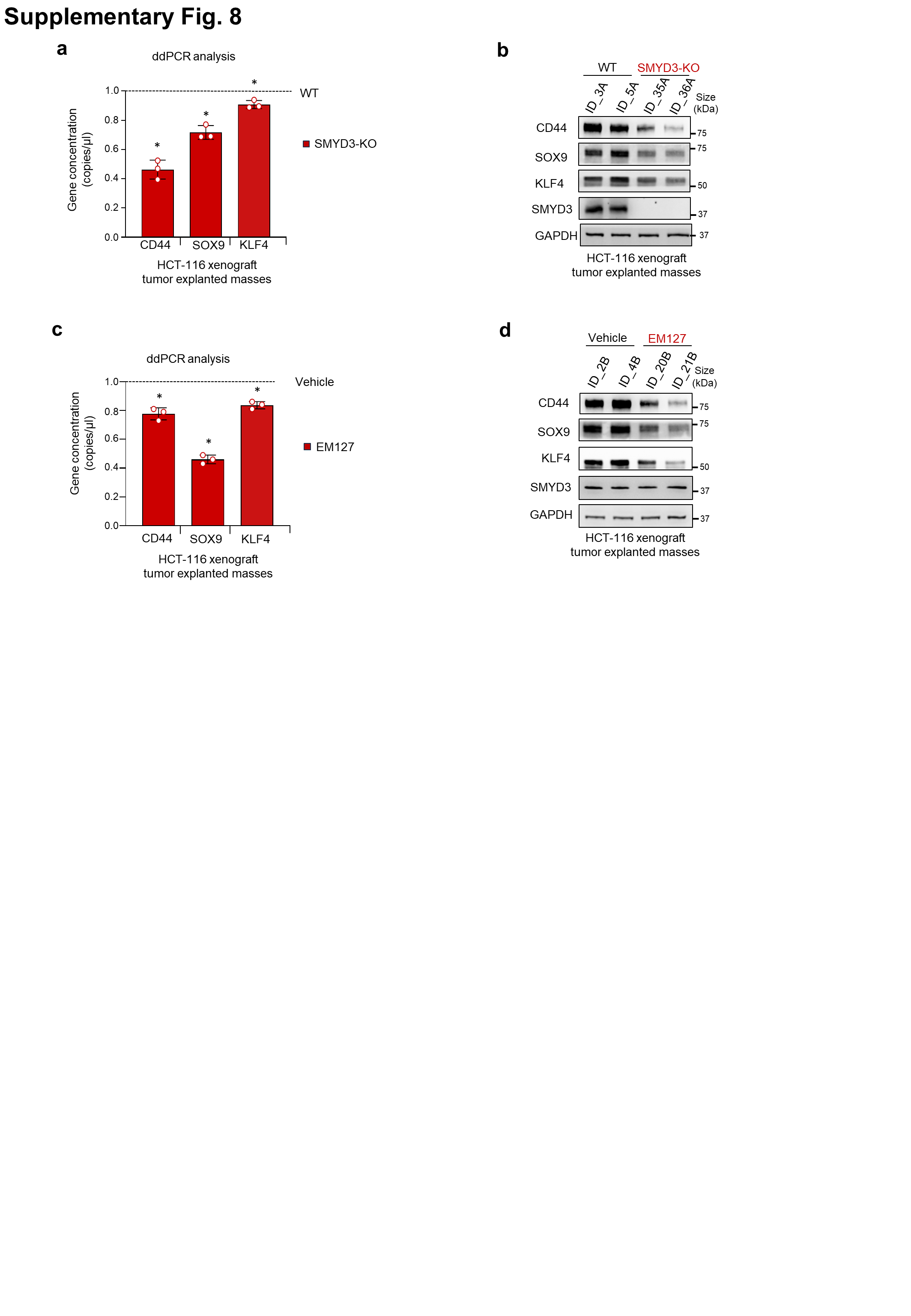


**Supplementary Figure 8.** **Molecular analysis of tumors explanted from HCT-116 cell xenograft mice.** (**a**) ddPCR analysis and (**b**) immunoblot analysis of stemness-related c-MYC target genes in tumors explanted from WT or SMYD3-KO HCT-116 cell xenograft mice (see treatment scheme depicted in Figure 6a). GAPDH was used as a loading control. *p<0.05 SMYD3-KO vs WT parental cells. (**c**) ddPCR analysis and (**d**) immunoblot analysis of stemness-related c-MYC target genes in tumors explanted from WT HCT-116 cell xenograft mice treated or not with EM127 (10 mg/kg) (see treatment scheme depicted in Figure 6e). GAPDH was used as a loading control. *p < 0.05 EM127-treated vs untreated. Where applicable, data are expressed as means ± SD of 3 independent experiments.

**Figure. S9.**


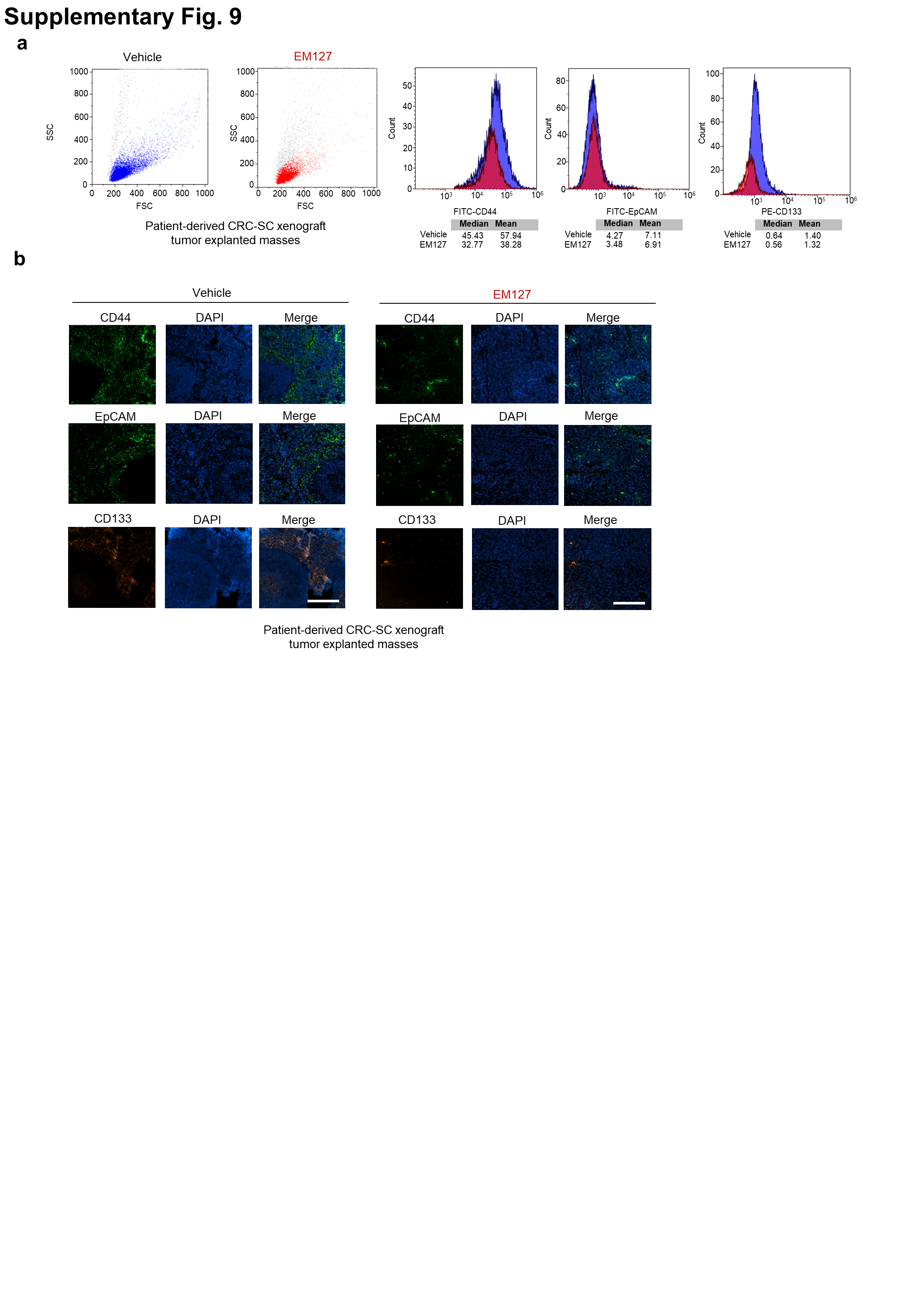


**Supplementary Figure 9. Characterization of CRC-SC markers expression in tumors explanted from patient-derived CRC-SC xenograft mice.** (**a**) Flow cytometry analysis of the CRC-SC markers CD44, EpCAM, and CD133 in tumors explanted from patient-derived CRC-SC xenograft mice treated (red) or not (blue) with EM127 (10 mg/kg) (see treatment scheme depicted in Figure 8f). (**b**) Immunostaining of the CRC-SC markers CD44 (green), EpCAM (green), and CD133 (orange) with nuclei counterstained with DAPI (blue), of colon sections from patient-derived CRC-SC xenograft mice treated or not with EM127 (10 mg/kg) (see treatment scheme depicted in Figure 8f). Scale bar: 200 μm.

**Table S1.**

| **Supplementary Table 1:** List of the 64 P-proteins identified in the Developmental Biology Reactome cluster (Reactome Id: R-HSA-1266738.14) that are implicated in stemness-related activities. The following Reactome pathways were found to include multiple P-proteins: Transcriptional regulation of pluripotent stem cells, Id: R-HSA-452723.4; Signaling by NODAL, Id: R-HSA-1181150.3; Activation of HOX genes during differentiation Id: R-HSA-5619507.5; Keratinization, Id: R-HSA-6805567.5. Proteins are listed in alphabetical order by UniProt entry name. | | | | | | | |
| --- | --- | --- | --- | --- | --- | --- | --- |
| **UniProt accession number** | **UniProt entry name** | **Gene** | **Protein names** | **Length** | **Function** | **Reactome ID** | **P-tripeptide matches** |
| O00292 | LFTY2_HUMAN | Lefty2 Ebaf Lefta Leftya Tgfb4 Psec0024 | Left-right determination factor 2 (Endometrial bleeding-associated factor) (Left-right determination factor A) (Protein lefty-2) (Protein lefty-A) (Transforming growth factor beta-4) (TGF-beta-4) | 366 | Required for left-right (L-R) asymmetry determination of organ systems in mammals. May play a role in endometrial bleeding. | R-HSA-114608; R-HSA-1181150; R-HSA-1433617; | NFW:185, |
| O14686 | KMT2D_HUMAN | Kmt2d Alr Mll2 Mll4 | Histone-lysine N-methyltransferase 2D (Lysine N-methyltransferase 2D) (EC 2.1.1.354) (ALL1-related protein) (Myeloid/lymphoid or mixed-lineage leukemia protein 2) | 5537 | Histone methyltransferase. Methylates 'Lys-4' of histone H3 (H3K4me). H3K4me represents a specific tag for epigenetic transcriptional activation. Acts as a coactivator for estrogen receptor by being recruited by ESR1, thereby activating transcription. {ECO:0000269|PubMed:16603732, ECO:0000269|PubMed:17500065, ECO:0000269|PubMed:17851529}. | R-HSA-201722; R-HSA-3214841; R-HSA-3769402; R-HSA-5617472; R-HSA-8936459; | LFF:241, NFH:2586, |
| O15379 | HDAC3_HUMAN | Hdac3 | Histone deacetylase 3 (HD3) (EC 3.5.1.98) (RPD3-2) (SMAP45) | 428 | Responsible for the deacetylation of lysine residues on the N-terminal part of the core histones (H2A, H2B, H3 and H4), and some other non-histone substrates. Histone deacetylation gives a tag for epigenetic repression and plays an important role in transcriptional regulation, cell cycle progression and developmental events. Histone deacetylases act via the formation of large multiprotein complexes. Participates in the BCL6 transcriptional repressor activity by deacetylating the H3 'Lys-27' (H3K27) on enhancer elements, antagonizing EP300 acetyltransferase activity and repressing proximal gene expression. Probably participates in the regulation of transcription through its binding to the zinc-finger transcription factor YY1; increases YY1 repression activity. Required to repress transcription of the POU1F1 transcription factor. Acts as a molecular chaperone for shuttling phosphorylated NR2C1 to PML bodies for sumoylation (PubMed:21444723, PubMed:23911289). Contributes, together with XBP1 isoform 1, to the activation of NFE2L2-mediated HMOX1 transcription factor gene expression in a PI(3)K/mTORC2/Akt-dependent signaling pathway leading to endothelial cell (EC) survival under disturbed flow/oxidative stress (PubMed:25190803). Regulates both the transcriptional activation and repression phases of the circadian clock in a deacetylase activity-independent manner (By similarity). During the activation phase, promotes the accumulation of ubiquitinated ARNTL/BMAL1 at the E-boxes and during the repression phase, blocks FBXL3-mediated CRY1/2 ubiquitination and promotes the interaction of CRY1 and ARNTL/BMAL1 (By similarity). The NCOR1-HDAC3 complex regulates the circadian expression of the core clock gene ARTNL/BMAL1 and the genes involved in lipid metabolism in the liver (By similarity). Serves as a corepressor of RARA, causing its deacetylation and inhibition of RARE DNA element binding (PubMed:28167758). In association with RARA, plays a role in the repression of microRNA-10a and thereby in the inflammatory response (PubMed:28167758). Interacts with SETD5 (By similarity). {ECO:0000250|UniProtKB:O88895, ECO:0000269|PubMed:21444723, ECO:0000269|PubMed:23911289, ECO:0000269|PubMed:25190803, ECO:0000269|PubMed:28167758}. | R-HSA-1368071; R-HSA-193670; R-HSA-1989781; R-HSA-2122947; R-HSA-2151201; R-HSA-2644606; R-HSA-2894862; R-HSA-3214815; R-HSA-350054; R-HSA-381340; R-HSA-390471; R-HSA-400206; R-HSA-400253; R-HSA-5617472; R-HSA-8940973; R-HSA-8943724; R-HSA-9022537; R-HSA-9022692; R-HSA-9029569; R-HSA-9609690; | NYF:197, NAF:86, NFH:15, |
| O15550 | KDM6A_HUMAN | Kdm6a Utx | Lysine-specific demethylase 6A (EC 1.14.11.68) (Histone demethylase UTX) (Ubiquitously-transcribed TPR protein on the X chromosome) (Ubiquitously-transcribed X chromosome tetratricopeptide repeat protein) ([histone H3]-trimethyl-L-lysine (27) demethylase 6A) | 1401 | Histone demethylase that specifically demethylates 'Lys-27' of histone H3, thereby playing a central role in histone code (PubMed:17851529, PubMed:17713478, PubMed:17761849). Demethylates trimethylated and dimethylated but not monomethylated H3 'Lys-27' (PubMed:17851529, PubMed:17713478, PubMed:17761849). Plays a central role in regulation of posterior development, by regulating HOX gene expression (PubMed:17851529). Demethylation of 'Lys-27' of histone H3 is concomitant with methylation of 'Lys-4' of histone H3, and regulates the recruitment of the PRC1 complex and monoubiquitination of histone H2A (PubMed:17761849). Plays a demethylase-independent role in chromatin remodeling to regulate T-box family member-dependent gene expression (By similarity). {ECO:0000250|UniProtKB:O70546, ECO:0000269|PubMed:17713478, ECO:0000269|PubMed:17761849, ECO:0000269|PubMed:17851529, ECO:0000269|PubMed:18003914}. | R-HSA-3214842; R-HSA-5617472; | DFF:94, NAF:144, NDF:1178, NNF:1150, |
| O43364 | HXA2_HUMAN | Hoxa2 Hox1k | Homeobox protein Hox-A2 (Homeobox protein Hox-1K) | 376 | Sequence-specific transcription factor which is part of a developmental regulatory system that provides cells with specific positional identities on the anterior-posterior axis. | R-HSA-5617472; R-HSA-9010553; | DFF:360, |
| O43548 | TGM5_HUMAN | Tgm5 Tgmx | Protein-glutamine gamma-glutamyltransferase 5 (EC 2.3.2.13) (Transglutaminase X) (TG(X)) (TGX) (TGase X) (Transglutaminase-5) (TGase-5) | 720 | Catalyzes the cross-linking of proteins and the conjugation of polyamines to proteins. Contributes to the formation of the cornified cell envelope of keratinocytes. | R-HSA-6809371; | NFI:425, NFH:335, |
| O60437 | PEPL_HUMAN | Ppl Kiaa0568 | Periplakin (190 kDa paraneoplastic pemphigus antigen) (195 kDa cornified envelope precursor protein) | 1756 | Component of the cornified envelope of keratinocytes. May link the cornified envelope to desmosomes and intermediate filaments. May act as a localization signal in PKB/AKT-mediated signaling. {ECO:0000269|PubMed:9412476}. | R-HSA-6809371; R-HSA-8851680; | NIF:169, NFI:257, |
| O75376 | NCOR1_HUMAN | Ncor1 Kiaa1047 | Nuclear receptor corepressor 1 (N-CoR) (N-CoR1) | 2440 | Mediates transcriptional repression by certain nuclear receptors (PubMed:20812024). Part of a complex which promotes histone deacetylation and the formation of repressive chromatin structures which may impede the access of basal transcription factors. Participates in the transcriptional repressor activity produced by BCL6. Recruited by ZBTB7A to the androgen response elements/ARE on target genes, negatively regulates androgen receptor signaling and androgen-induced cell proliferation (PubMed:20812024). Mediates the NR1D1-dependent repression and circadian regulation of TSHB expression (By similarity). The NCOR1-HDAC3 complex regulates the circadian expression of the core clock gene ARTNL/BMAL1 and the genes involved in lipid metabolism in the liver (By similarity). {ECO:0000250|UniProtKB:Q60974, ECO:0000269|PubMed:14527417, ECO:0000269|PubMed:20812024}. | R-HSA-1251985; R-HSA-1368071; R-HSA-1989781; R-HSA-2122947; R-HSA-2151201; R-HSA-2173795; R-HSA-2644606; R-HSA-2894862; R-HSA-3214815; R-HSA-350054; R-HSA-381340; R-HSA-383280; R-HSA-400206; R-HSA-400253; R-HSA-5617472; R-HSA-9022537; R-HSA-9022692; R-HSA-9029569; R-HSA-9609690; R-HSA-9623433; | LFF:291, NFI:1931, NFY:664, |
| O75530 | EED_HUMAN | Eed | Polycomb protein EED (hEED) (Embryonic ectoderm development protein) (WD protein associating with integrin cytoplasmic tails 1) (WAIT-1) | 441 | Polycomb group (PcG) protein. Component of the PRC2/EED-EZH2 complex, which methylates 'Lys-9' and 'Lys-27' of histone H3, leading to transcriptional repression of the affected target gene. Also recognizes 'Lys-26' trimethylated histone H1 with the effect of inhibiting PRC2 complex methyltransferase activity on nucleosomal histone H3 'Lys-27', whereas H3 'Lys-27' recognition has the opposite effect, enabling the propagation of this repressive mark. The PRC2/EED-EZH2 complex may also serve as a recruiting platform for DNA methyltransferases, thereby linking two epigenetic repression systems. Genes repressed by the PRC2/EED-EZH2 complex include HOXC8, HOXA9, MYT1 and CDKN2A. {ECO:0000269|PubMed:10581039, ECO:0000269|PubMed:14532106, ECO:0000269|PubMed:15225548, ECO:0000269|PubMed:15231737, ECO:0000269|PubMed:15385962, ECO:0000269|PubMed:16357870, ECO:0000269|PubMed:18285464, ECO:0000269|PubMed:20974918, ECO:0000269|PubMed:28229514, ECO:0000269|PubMed:9584199}. | R-HSA-212300; R-HSA-2559580; R-HSA-3214841; R-HSA-5617472; R-HSA-8943724; R-HSA-8953750; R-HSA-9609690; | NFY:146, |
| O75593 | FOXH1_HUMAN | Foxh1 Fast1 Fast2 | Forkhead box protein H1 (Forkhead activin signal transducer 1) (Fast-1) (hFAST-1) (Forkhead activin signal transducer 2) (Fast-2) | 365 | Transcriptional activator. Recognizes and binds to the DNA sequence 5'-TGT[GT][GT]ATT-3'. Required for induction of the goosecoid (GSC) promoter by TGF-beta or activin signaling. Forms a transcriptionally active complex containing FOXH1/SMAD2/SMAD4 on a site on the GSC promoter called TARE (TGF-beta/activin response element). {ECO:0000269|PubMed:9702198}. | R-HSA-1181150; R-HSA-1502540; | NFW:106, |
| O75610 | LFTY1_HUMAN | Lefty1 Leftb Leftyb Unq278/Pro317 | Left-right determination factor 1 (Left-right determination factor B) (Protein lefty-1) (Protein lefty-B) | 366 | Required for left-right axis determination as a regulator of LEFTY2 and NODAL. | R-HSA-1181150; R-HSA-1433617; | NFW:185, |
| O75928 | PIAS2_HUMAN | Pias2 Piasx | E3 SUMO-protein ligase PIAS2 (EC 2.3.2.-) (Androgen receptor-interacting protein 3) (ARIP3) (DAB2-interacting protein) (DIP) (E3 SUMO-protein transferase PIAS2) (Msx-interacting zinc finger protein) (Miz1) (PIAS-NY protein) (Protein inhibitor of activated STAT x) (Protein inhibitor of activated STAT2) | 621 | Functions as an E3-type small ubiquitin-like modifier (SUMO) ligase, stabilizing the interaction between UBE2I and the substrate, and as a SUMO-tethering factor. Plays a crucial role as a transcriptional coregulator in various cellular pathways, including the STAT pathway, the p53 pathway and the steroid hormone signaling pathway. The effects of this transcriptional coregulation, transactivation or silencing may vary depending upon the biological context and the PIAS2 isoform studied. However, it seems to be mostly involved in gene silencing. Binds to sumoylated ELK1 and enhances its transcriptional activity by preventing recruitment of HDAC2 by ELK1, thus reversing SUMO-mediated repression of ELK1 transactivation activity. Isoform PIAS2-beta, but not isoform PIAS2-alpha, promotes MDM2 sumoylation. Isoform PIAS2-alpha promotes PARK7 sumoylation. Isoform PIAS2-beta promotes NCOA2 sumoylation more efficiently than isoform PIAS2-alpha. Isoform PIAS2-alpha sumoylates PML at'Lys-65' and 'Lys-160'. {ECO:0000269|PubMed:15920481, ECO:0000269|PubMed:15976810, ECO:0000269|PubMed:22406621}. | R-HSA-3108214 [O75928-1]; R-HSA-3232118 [O75928-1]; R-HSA-3232142 [O75928-1]; R-HSA-3899300 [O75928-1]; R-HSA-4090294; R-HSA-4551638 [O75928-2]; R-HSA-5617472; | KFF:177, |
| O95678 | K2C75_HUMAN | Krt75 K6hf Kb18 | Keratin, type II cytoskeletal 75 (Cytokeratin-75) (CK-75) (Keratin-6 hair follicle) (hK6hf) (Keratin-75) (K75) (Type II keratin-K6hf) (Type-II keratin Kb18) | 551 | Plays a central role in hair and nail formation. Essential component of keratin intermediate filaments in the companion layer of the hair follicle. | R-HSA-6805567; R-HSA-6809371; | NFI:282, |
| P04632 | CPNS1_HUMAN | Capns1 Capn4 Capns | Calpain small subunit 1 (CSS1) (Calcium-activated neutral proteinase small subunit) (CANP small subunit) (Calcium-dependent protease small subunit) (CDPS) (Calcium-dependent protease small subunit 1) (Calpain regulatory subunit) | 268 | Regulatory subunit of the calcium-regulated non-lysosomal thiol-protease which catalyzes limited proteolysis of substrates involved in cytoskeletal remodeling and signal transduction. | R-HSA-1474228; R-HSA-6809371; R-HSA-8862803; | NFI:228, |
| P05787 | K2C8_HUMAN | Krt8 Cyk8 | Keratin, type II cytoskeletal 8 (Cytokeratin-8) (CK-8) (Keratin-8) (K8) (Type-II keratin Kb8) | 483 | Together with KRT19, helps to link the contractile apparatus to dystrophin at the costameres of striated muscle. {ECO:0000269|PubMed:16000376}. | R-HSA-6805567; R-HSA-6809371; | NFR:45, |
| P07384 | CAN1_HUMAN | Capn1 Canpl1 Pig30 | Calpain-1 catalytic subunit (EC 3.4.22.52) (Calcium-activated neutral proteinase 1) (CANP 1) (Calpain mu-type) (Calpain-1 large subunit) (Cell proliferation-inducing gene 30 protein) (Micromolar-calpain) (muCANP) | 714 | Calcium-regulated non-lysosomal thiol-protease which catalyzes limited proteolysis of substrates involved in cytoskeletal remodeling and signal transduction (PubMed:21531719, PubMed:2400579). Proteolytically cleaves CTBP1 at 'Asn-375', 'Gly-387' and 'His-409' (PubMed:23707407). {ECO:0000269|PubMed:21531719, ECO:0000269|PubMed:23707407, ECO:0000269|PubMed:2400579}. | R-HSA-1474228; R-HSA-6798695; R-HSA-6809371; R-HSA-8862803; | DFF:463, NFK:546 |
| P09038 | FGF2_HUMAN | Fgf2 Fgfb | Fibroblast growth factor 2 (FGF-2) (Basic fibroblast growth factor) (bFGF) (Heparin-binding growth factor 2) (HBGF-2) | 288 | Acts as a ligand for FGFR1, FGFR2, FGFR3 and FGFR4 (PubMed:8663044). Also acts as an integrin ligand which is required for FGF2 signaling (PubMed:28302677). Binds to integrin ITGAV:ITGB3 (PubMed:28302677). Plays an important role in the regulation of cell survival, cell division, cell differentiation and cell migration (PubMed:8663044, PubMed:28302677). Functions as a potent mitogen in vitro (PubMed:1721615, PubMed:3964259, PubMed:3732516). Can induce angiogenesis (PubMed:23469107, PubMed:28302677). Mediates phosphorylation of ERK1/2 and thereby promotes retinal lens fiber differentiation (PubMed:29501879). {ECO:0000269|PubMed:1721615, ECO:0000269|PubMed:29501879, ECO:0000269|PubMed:3732516, ECO:0000269|PubMed:3964259}. | R-HSA-109704; R-HSA-1257604; R-HSA-1839122; R-HSA-1839130; R-HSA-190322; R-HSA-190370; R-HSA-190372; R-HSA-190373; R-HSA-190375; R-HSA-190377; R-HSA-2033514; R-HSA-2033519; R-HSA-2219530; R-HSA-2892247; R-HSA-3000170; R-HSA-3000171; R-HSA-5654219; R-HSA-5654221; R-HSA-5654227; R-HSA-5654228; R-HSA-5654687; R-HSA-5654688; R-HSA-5654689; R-HSA-5654693; R-HSA-5654695; R-HSA-5654699; R-HSA-5654700; R-HSA-5654704; R-HSA-5654706; R-HSA-5654710; R-HSA-5654712; R-HSA-5654719; R-HSA-5654720; R-HSA-5654726; R-HSA-5654727; R-HSA-5654732; R-HSA-5654733; R-HSA-5655253; R-HSA-5655302; R-HSA-5658623; R-HSA-5673001; R-HSA-6785807; R-HSA-6811558; R-HSA-8851708; R-HSA-8853338; | FFF:235, |
| P10276 | RARA_HUMAN | Rara Nr1b1 | Retinoic acid receptor alpha (RAR-alpha) (Nuclear receptor subfamily 1 group B member 1) | 462 | Receptor for retinoic acid (PubMed:19850744, PubMed:16417524, PubMed:20215566). Retinoic acid receptors bind as heterodimers to their target response elements in response to their ligands, all-trans or 9-cis retinoic acid, and regulate gene expression in various biological processes (PubMed:28167758). The RXR/RAR heterodimers bind to the retinoic acid response elements (RARE) composed of tandem 5'-AGGTCA-3' sites known as DR1-DR5 (PubMed:28167758). In the absence of ligand, the RXR-RAR heterodimers associate with a multiprotein complex containing transcription corepressors that induce histone deacetylation, chromatin condensation and transcriptional suppression (PubMed:16417524). On ligand binding, the corepressors dissociate from the receptors and associate with the coactivators leading to transcriptional activation (PubMed:9267036, PubMed:19850744, PubMed:20215566). Formation of a complex with histone deacetylases might lead to inhibition of RARE DNA element binding and to transcriptional repression (PubMed:28167758). Transcriptional activation and RARE DNA element binding might be supported by the transcription factor KLF2 (PubMed:28167758). RARA plays an essential role in the regulation of retinoic acid-induced germ cell development during spermatogenesis (By similarity). Has a role in the survival of early spermatocytes at the beginning prophase of meiosis (By similarity). In Sertoli cells, may promote the survival and development of early meiotic prophase spermatocytes (By similarity). In concert with RARG, required for skeletal growth, matrix homeostasis and growth plate function (By similarity). Together with RXRA, positively regulates microRNA-10a expression, thereby inhibiting the GATA6/VCAM1 signaling response to pulsatile shear stress in vascular endothelial cells (PubMed:28167758). In association with HDAC3, HDAC5 and HDAC7 corepressors, plays a role in the repression of microRNA-10a and thereby promotes the inflammatory response (PubMed:28167758). {ECO:0000250|UniProtKB:P11416, ECO:0000269|PubMed:16417524, ECO:0000269|PubMed:19850744, ECO:0000269|PubMed:20215566, ECO:0000269|PubMed:28167758, ECO:0000269|PubMed:9267036}. | R-HSA-383280; R-HSA-4090294; R-HSA-5362517; R-HSA-5617472; R-HSA-9616222; | FFF:26, |
| P14652 | HXB2_HUMAN | Hoxb2 Hox2h | Homeobox protein Hox-B2 (Homeobox protein Hox-2.8) (Homeobox protein Hox-2H) (K8) | 356 | Sequence-specific transcription factor which is part of a developmental regulatory system that provides cells with specific positional identities on the anterior-posterior axis. {ECO:0000269|PubMed:10595394}. | R-HSA-5617472; | NFF:308, DFF:342, |
| P15924 | DESP_HUMAN | Dsp | Desmoplakin (DP) (250/210 kDa paraneoplastic pemphigus antigen) | 2871 | Major high molecular weight protein of desmosomes. Involved in the organization of the desmosomal cadherin-plakoglobin complexes into discrete plasma membrane domains and in the anchoring of intermediate filaments to the desmosomes. | R-HSA-351906; R-HSA-6798695; R-HSA-6805567; R-HSA-6809371; | QFF:380, KFF:2542, NFR:732, |
| P19012 | K1C15_HUMAN | Krt15 Krtb | Keratin, type I cytoskeletal 15 (Cytokeratin-15) (CK-15) (Keratin-15) (K15) | 456 |  | R-HSA-6805567; R-HSA-6809371; | NFH:435, |
| P19013 | K2C4_HUMAN | Krt4 Cyk4 | Keratin, type II cytoskeletal 4 (Cytokeratin-4) (CK-4) (Keratin-4) (K4) (Type-II keratin Kb4) | 520 |  | R-HSA-6805567; R-HSA-6809371; | NDF:239, |
| P19793 | RXRA_HUMAN | Rxra Nr2b1 | Retinoic acid receptor RXR-alpha (Nuclear receptor subfamily 2 group B member 1) (Retinoid X receptor alpha) | 462 | Receptor for retinoic acid that acts as a transcription factor (PubMed:11162439, PubMed:11915042). Forms homo- or heterodimers with retinoic acid receptors (RARs) and binds to target response elements in response to their ligands, all-trans or 9-cis retinoic acid, to regulate gene expression in various biological processes (PubMed:10195690, PubMed:11162439, PubMed:11915042, PubMed:28167758, PubMed:17761950, PubMed:16107141, PubMed:18800767, PubMed:19167885). The RAR/RXR heterodimers bind to the retinoic acid response elements (RARE) composed of tandem 5'-AGGTCA-3' sites known as DR1-DR5 to regulate transcription (PubMed:10195690, PubMed:11162439, PubMed:11915042, PubMed:17761950, PubMed:28167758). The high affinity ligand for retinoid X receptors (RXRs) is 9-cis retinoic acid (PubMed:1310260). In the absence of ligand, the RXR-RAR heterodimers associate with a multiprotein complex containing transcription corepressors that induce histone deacetylation, chromatin condensation and transcriptional suppression (PubMed:20215566). On ligand binding, the corepressors dissociate from the receptors and coactivators are recruited leading to transcriptional activation (PubMed:20215566, PubMed:9267036). Serves as a common heterodimeric partner for a number of nuclear receptors, such as RARA, RARB and PPARA (PubMed:10195690, PubMed:11915042, PubMed:28167758, PubMed:29021580). The RXRA/RARB heterodimer can act as a transcriptional repressor or transcriptional activator, depending on the RARE DNA element context (PubMed:29021580). The RXRA/PPARA heterodimer is required for PPARA transcriptional activity on fatty acid oxidation genes such as ACOX1 and the P450 system genes (PubMed:10195690). Together with RARA, positively regulates microRNA-10a expression, thereby inhibiting the GATA6/VCAM1 signaling response to pulsatile shear stress in vascular endothelial cells (PubMed:28167758). Acts as an enhancer of RARA binding to RARE DNA element (PubMed:28167758). May facilitate the nuclear import of heterodimerization partners such as VDR and NR4A1 (PubMed:12145331, PubMed:15509776). Promotes myelin debris phagocytosis and remyelination by macrophages (PubMed:26463675). Plays a role in the attenuation of the innate immune system in response to viral infections, possibly by negatively regulating the transcription of antiviral genes such as type I IFN genes (PubMed:25417649). Involved in the regulation of calcium signaling by repressing ITPR2 gene expression, thereby controlling cellular senescence (PubMed:30216632). {ECO:0000269|PubMed:10195690, ECO:0000269|PubMed:11162439, ECO:0000269|PubMed:11915042, ECO:0000269|PubMed:12145331, ECO:0000269|PubMed:1310260, ECO:0000269|PubMed:15509776, ECO:0000269|PubMed:16107141, ECO:0000269|PubMed:17761950, ECO:0000269|PubMed:18800767, ECO:0000269|PubMed:19167885, ECO:0000269|PubMed:20215566, ECO:0000269|PubMed:25417649, ECO:0000269|PubMed:26463675, ECO:0000269|PubMed:28167758, ECO:0000269|PubMed:29021580, ECO:0000269|PubMed:30216632, ECO:0000269|PubMed:9267036}. | R-HSA-1368082; R-HSA-1368108; R-HSA-159418; R-HSA-192105; R-HSA-193368; R-HSA-193807; R-HSA-1989781; R-HSA-200425; R-HSA-204174; R-HSA-211976; R-HSA-2151201; R-HSA-2426168; R-HSA-381340; R-HSA-383280; R-HSA-400206; R-HSA-400253; R-HSA-4090294; R-HSA-5362517; R-HSA-5617472; R-HSA-9029558; R-HSA-9029569; R-HSA-9031525; R-HSA-9031528; R-HSA-9616222; R-HSA-9623433; R-HSA-9632974; | LFF:436, FFF:437, |
| P20930 | FILA_HUMAN | Flg | Filaggrin | 4061 | Aggregates keratin intermediate filaments and promotes disulfide-bond formation among the intermediate filaments during terminal differentiation of mammalian epidermis. | R-HSA-6809371; | NIF:7, |
| P22735 | TGM1_HUMAN | Tgm1 Ktg | Protein-glutamine gamma-glutamyltransferase K (EC 2.3.2.13) (Epidermal TGase) (Transglutaminase K) (TG(K)) (TGK) (TGase K) (Transglutaminase-1) (TGase-1) | 817 | Catalyzes the cross-linking of proteins and the conjugation of polyamines to proteins. Responsible for cross-linking epidermal proteins during formation of the stratum corneum. Involved in cell proliferation (PubMed:26220141). {ECO:0000269|PubMed:26220141}. | R-HSA-6809371; | NFH:434, |
| P24928 | RPB1_HUMAN | Polr2a Polr2 | DNA-directed RNA polymerase II subunit RPB1 (RNA polymerase II subunit B1) (EC 2.7.7.6) (DNA-directed RNA polymerase II subunit A) (DNA-directed RNA polymerase III largest subunit) (RNA-directed RNA polymerase II subunit RPB1) (EC 2.7.7.48) | 1970 | DNA-dependent RNA polymerase catalyzes the transcription of DNA into RNA using the four ribonucleoside triphosphates as substrates. Largest and catalytic component of RNA polymerase II which synthesizes mRNA precursors and many functional non-coding RNAs. Forms the polymerase active center together with the second largest subunit. Pol II is the central component of the basal RNA polymerase II transcription machinery. It is composed of mobile elements that move relative to each other. RPB1 is part of the core element with the central large cleft, the clamp element that moves to open and close the cleft and the jaws that are thought to grab the incoming DNA template. At the start of transcription, a single-stranded DNA template strand of the promoter is positioned within the central active site cleft of Pol II. A bridging helix emanates from RPB1 and crosses the cleft near the catalytic site and is thought to promote translocation of Pol II by acting as a ratchet that moves the RNA-DNA hybrid through the active site by switching from straight to bent conformations at each step of nucleotide addition. During transcription elongation, Pol II moves on the template as the transcript elongates. Elongation is influenced by the phosphorylation status of the C-terminal domain (CTD) of Pol II largest subunit (RPB1), which serves as a platform for assembly of factors that regulate transcription initiation, elongation, termination and mRNA processing. Regulation of gene expression levels depends on the balance between methylation and acetylation levels of tha CTD-lysines (By similarity). Initiation or early elongation steps of transcription of growth-factors-induced immediate early genes are regulated by the acetylation status of the CTD (PubMed:24207025). Methylation and dimethylation have a repressive effect on target genes expression (By similarity). {ECO:0000250|UniProtKB:P08775, ECO:0000269|PubMed:20231364, ECO:0000269|PubMed:23748380, ECO:0000269|PubMed:24207025, ECO:0000269|PubMed:26124092, ECO:0000269|PubMed:9852112}.; (Microbial infection) Acts as an RNA-dependent RNA polymerase when associated with small delta antigen of Hepatitis delta virus, acting both as a replicate and transcriptase for the viral RNA circular genome. {ECO:0000269|PubMed:18032511}. | R-HSA-112382; R-HSA-113418; R-HSA-167152; R-HSA-167158; R-HSA-167160; R-HSA-167161; R-HSA-167162; R-HSA-167172; R-HSA-167200; R-HSA-167238; R-HSA-167242; R-HSA-167243; R-HSA-167246; R-HSA-167287; R-HSA-167290; R-HSA-168325; R-HSA-203927; R-HSA-5578749; R-HSA-5601884; R-HSA-5617472; R-HSA-674695; R-HSA-6781823; R-HSA-6781827; R-HSA-6782135; R-HSA-6782210; R-HSA-6796648; R-HSA-6803529; R-HSA-6807505; R-HSA-72086; R-HSA-72163; R-HSA-72165; R-HSA-72203; R-HSA-73776; R-HSA-73779; R-HSA-75953; R-HSA-75955; R-HSA-76042; R-HSA-77075; R-HSA-8851708; R-HSA-9018519; R-HSA-9670095; | FFF:836, NNF:764, NFK:765 |
| P27037 | AVR2A_HUMAN | Acvr2a Acvr2 | Activin receptor type-2A (EC 2.7.11.30) (Activin receptor type IIA) (ACTR-IIA) (ACTRIIA) | 513 | On ligand binding, forms a receptor complex consisting of two type II and two type I transmembrane serine/threonine kinases. Type II receptors phosphorylate and activate type I receptors which autophosphorylate, then bind and activate SMAD transcriptional regulators. Receptor for activin A, activin B and inhibin A. Mediates induction of adipogenesis by GDF6 (By similarity). {ECO:0000250|UniProtKB:P27038, ECO:0000269|PubMed:1314589}. | R-HSA-1181150; R-HSA-1433617; R-HSA-1502540; R-HSA-201451; | LFF:31, |
| P27539 | GDF1_HUMAN | Gdf1 | Embryonic growth/differentiation factor 1 (GDF-1) | 372 | May mediate cell differentiation events during embryonic development. | R-HSA-1181150; | LFF:348, |
| P29122 | PCSK6_HUMAN | Pcsk6 Pace4 | Proprotein convertase subtilisin/kexin type 6 (EC 3.4.21.-) (Paired basic amino acid cleaving enzyme 4) (Subtilisin-like proprotein convertase 4) (SPC4) (Subtilisin/kexin-like protease PACE4) | 969 | Serine endoprotease that processes various proproteins by cleavage at paired basic amino acids, recognizing the RXXX[KR]R consensus motif. Likely functions in the constitutive secretory pathway, with unique restricted distribution in both neuroendocrine and non-neuroendocrine tissues. | R-HSA-1181150; R-HSA-167060; R-HSA-6809371; R-HSA-8963889; | NFH:869, |
| P30876 | RPB2_HUMAN | Polr2b | DNA-directed RNA polymerase II subunit RPB2 (EC 2.7.7.6) (DNA-directed RNA polymerase II 140 kDa polypeptide) (DNA-directed RNA polymerase II subunit B) (RNA polymerase II subunit 2) (RNA polymerase II subunit B2) | 1174 | DNA-dependent RNA polymerase catalyzes the transcription of DNA into RNA using the four ribonucleoside triphosphates as substrates. Second largest component of RNA polymerase II which synthesizes mRNA precursors and many functional non-coding RNAs. Proposed to contribute to the polymerase catalytic activity and forms the polymerase active center together with the largest subunit. Pol II is the central component of the basal RNA polymerase II transcription machinery. It is composed of mobile elements that move relative to each other. RPB2 is part of the core element with the central large cleft, the clamp element that moves to open and close the cleft and the jaws that are thought to grab the incoming DNA template (By similarity). {ECO:0000250, ECO:0000269|PubMed:9852112}. | R-HSA-112382; R-HSA-113418; R-HSA-167152; R-HSA-167158; R-HSA-167160; R-HSA-167161; R-HSA-167162; R-HSA-167172; R-HSA-167200; R-HSA-167238; R-HSA-167242; R-HSA-167243; R-HSA-167246; R-HSA-167287; R-HSA-167290; R-HSA-168325; R-HSA-203927; R-HSA-5578749; R-HSA-5601884; R-HSA-5617472; R-HSA-674695; R-HSA-6781823; R-HSA-6781827; R-HSA-6782135; R-HSA-6782210; R-HSA-6796648; R-HSA-6803529; R-HSA-6807505; R-HSA-72086; R-HSA-72163; R-HSA-72165; R-HSA-72203; R-HSA-73776; R-HSA-73779; R-HSA-75953; R-HSA-75955; R-HSA-76042; R-HSA-77075; R-HSA-8851708; R-HSA-9018519; R-HSA-9670095; | NFI:319, NFH:739, |
| P35908 | K22E_HUMAN | Krt2 Krt2a Krt2e | Keratin, type II cytoskeletal 2 epidermal (Cytokeratin-2e) (CK-2e) (Epithelial keratin-2e) (Keratin-2 epidermis) (Keratin-2e) (K2e) (Type-II keratin Kb2) | 639 | Probably contributes to terminal cornification (PubMed:1380918). Associated with keratinocyte activation, proliferation and keratinization (PubMed:12598329). Plays a role in the establishment of the epidermal barrier on plantar skin (By similarity). {ECO:0000250|UniProtKB:Q3TTY5, ECO:0000269|PubMed:12598329, ECO:0000269|PubMed:1380918}. | R-HSA-6805567; R-HSA-6809371; | NDF:280, |
| P40763 | STAT3_HUMAN | Stat3 Aprf | Signal transducer and activator of transcription 3 (Acute-phase response factor) | 770 | Signal transducer and transcription activator that mediates cellular responses to interleukins, KITLG/SCF, LEP and other growth factors (PubMed:10688651, PubMed:12359225, PubMed:12873986, PubMed:15194700, PubMed:17344214, PubMed:18242580, PubMed:23084476). Once activated, recruits coactivators, such as NCOA1 or MED1, to the promoter region of the target gene (PubMed:17344214). May mediate cellular responses to activated FGFR1, FGFR2, FGFR3 and FGFR4 (PubMed:12873986). Upon activation of IL6ST/gp130 signaling by interleukin-6 (IL6), binds to the IL6-responsive elements identified in the promoters of various acute-phase protein genes (PubMed:12359225). Activated by IL31 through IL31RA (PubMed:15194700). Acts as a regulator of inflammatory response by regulating differentiation of naive CD4(+) T-cells into T-helper Th17 or regulatory T-cells (Treg): deacetylation and oxidation of lysine residues by LOXL3, leads to disrupt STAT3 dimerization and inhibit its transcription activity (PubMed:28065600). Involved in cell cycle regulation by inducing the expression of key genes for the progression from G1 to S phase, such as CCND1 (PubMed:17344214). Mediates the effects of LEP on melanocortin production, body energy homeostasis and lactation (By similarity). May play an apoptotic role by transctivating BIRC5 expression under LEP activation (PubMed:18242580). Cytoplasmic STAT3 represses macroautophagy by inhibiting EIF2AK2/PKR activity (PubMed:23084476). Plays a crucial role in basal beta cell functions, such as regulation of insulin secretion (By similarity). {ECO:0000250|UniProtKB:P42227, ECO:0000269|PubMed:10688651, ECO:0000269|PubMed:12359225, ECO:0000269|PubMed:12873986, ECO:0000269|PubMed:15194700, ECO:0000269|PubMed:17344214, ECO:0000269|PubMed:18242580, ECO:0000269|PubMed:23084476, ECO:0000269|PubMed:28065600}. | R-HSA-1059683; R-HSA-111453; R-HSA-1266695; R-HSA-1433557; R-HSA-1839117; R-HSA-186763; R-HSA-198745; R-HSA-2559582; R-HSA-2586552; R-HSA-2892247; R-HSA-390471; R-HSA-452723; R-HSA-6783783; R-HSA-6785807; R-HSA-8849474; R-HSA-8854691; R-HSA-8875791; R-HSA-8983432; R-HSA-8984722; R-HSA-8985947; R-HSA-9008059; R-HSA-9020933; R-HSA-9020956; R-HSA-9020958; R-HSA-9616222; R-HSA-9670439; R-HSA-9673767; R-HSA-9673770; R-HSA-982772; | NFF:491, |
| P48431 | SOX2_HUMAN | Sox2 | Transcription factor SOX-2 | 317 | Transcription factor that forms a trimeric complex with OCT4 on DNA and controls the expression of a number of genes involved in embryonic development such as YES1, FGF4, UTF1 and ZFP206 (By similarity). Binds to the proximal enhancer region of NANOG (By similarity). Critical for early embryogenesis and for embryonic stem cell pluripotency (PubMed:18035408). Downstream SRRT target that mediates the promotion of neural stem cell self-renewal (By similarity). Keeps neural cells undifferentiated by counteracting the activity of proneural proteins and suppresses neuronal differentiation (By similarity). May function as a switch in neuronal development (By similarity). {ECO:0000250|UniProtKB:P48430, ECO:0000250|UniProtKB:P48432, ECO:0000269|PubMed:18035408}. | R-HSA-2892245; R-HSA-2892247; R-HSA-3769402; R-HSA-452723; R-HSA-6785807; R-HSA-8986944; | NAF:46, |
| Q02413 | DSG1_HUMAN | Dsg1 Cdhf4 | Desmoglein-1 (Cadherin family member 4) (Desmosomal glycoprotein 1) (DG1) (DGI) (Pemphigus foliaceus antigen) | 1049 | Component of intercellular desmosome junctions. Involved in the interaction of plaque proteins and intermediate filaments mediating cell-cell adhesion. | R-HSA-351906; R-HSA-6798695; R-HSA-6805567; R-HSA-6809371; | NNF:222, |
| Q02487 | DSC2_HUMAN | Dsc2 Cdhf2 Dsc3 | Desmocollin-2 (Cadherin family member 2) (Desmocollin-3) (Desmosomal glycoprotein II) (Desmosomal glycoprotein III) | 901 | Component of intercellular desmosome junctions. Involved in the interaction of plaque proteins and intermediate filaments mediating cell-cell adhesion. May contribute to epidermal cell positioning (stratification) by mediating differential adhesiveness between cells that express different isoforms. | R-HSA-6805567; R-HSA-6809371; | NFK:403 |
| Q08554 | DSC1_HUMAN | Dsc1 Cdhf1 | Desmocollin-1 (Cadherin family member 1) (Desmosomal glycoprotein 2/3) (DG2/DG3) | 894 | Component of intercellular desmosome junctions. Involved in the interaction of plaque proteins and intermediate filaments mediating cell-cell adhesion. May contribute to epidermal cell positioning (stratification) by mediating differential adhesiveness between cells that express different isoforms. Linked to the keratinization of epithelial tissues. | R-HSA-6798695; R-HSA-6805567; R-HSA-6809371; | QFF:608, NFI:402, |
| Q09472 | EP300_HUMAN | Ep300 P300 | Histone acetyltransferase p300 (p300 HAT) (EC 2.3.1.48) (E1A-associated protein p300) (Histone butyryltransferase p300) (EC 2.3.1.-) (Histone crotonyltransferase p300) (EC 2.3.1.-) (Protein 2-hydroxyisobutyryltransferase p300) (EC 2.3.1.-) (Protein propionyltransferase p300) (EC 2.3.1.-) | 2414 | Functions as histone acetyltransferase and regulates transcription via chromatin remodeling (PubMed:23415232, PubMed:23934153, PubMed:8945521). Acetylates all four core histones in nucleosomes. Histone acetylation gives an epigenetic tag for transcriptional activation (PubMed:23415232, PubMed:23934153, PubMed:8945521). Mediates cAMP-gene regulation by binding specifically to phosphorylated CREB protein. Mediates acetylation of histone H3 at 'Lys-122' (H3K122ac), a modification that localizes at the surface of the histone octamer and stimulates transcription, possibly by promoting nucleosome instability. Mediates acetylation of histone H3 at 'Lys-27' (H3K27ac) (PubMed:23911289). Also functions as acetyltransferase for non-histone targets, such as ALX1, HDAC1, PRMT1 or SIRT2 (PubMed:12929931, PubMed:16762839, PubMed:18722353). Acetylates 'Lys-131' of ALX1 and acts as its coactivator (PubMed:12929931). Acetylates SIRT2 and is proposed to indirectly increase the transcriptional activity of TP53 through acetylation and subsequent attenuation of SIRT2 deacetylase function (PubMed:18722353). Acetylates HDAC1 leading to its inactivation and modulation of transcription (PubMed:16762839). Acetylates 'Lys-247' of EGR2 (By similarity). Acts as a TFAP2A-mediated transcriptional coactivator in presence of CITED2 (PubMed:12586840). Plays a role as a coactivator of NEUROD1-dependent transcription of the secretin and p21 genes and controls terminal differentiation of cells in the intestinal epithelium. Promotes cardiac myocyte enlargement. Can also mediate transcriptional repression. Acetylates FOXO1 and enhances its transcriptional activity (PubMed:15890677). Acetylates BCL6 wich disrupts its ability to recruit histone deacetylases and hinders its transcriptional repressor activity (PubMed:12402037). Participates in CLOCK or NPAS2-regulated rhythmic gene transcription; exhibits a circadian association with CLOCK or NPAS2, correlating with increase in PER1/2 mRNA and histone H3 acetylation on the PER1/2 promoter (PubMed:14645221). Acetylates MTA1 at 'Lys-626' which is essential for its transcriptional coactivator activity (PubMed:16617102). Acetylates XBP1 isoform 2; acetylation increases protein stability of XBP1 isoform 2 and enhances its transcriptional activity (PubMed:20955178). Acetylates PCNA; acetylation promotes removal of chromatin-bound PCNA and its degradation during nucleotide excision repair (NER) (PubMed:24939902). Acetylates MEF2D (PubMed:21030595). Acetylates and stabilizes ZBTB7B protein by antagonizing ubiquitin conjugation and degragation, this mechanism may be involved in CD4/CD8 lineage differentiation (PubMed:20810990). Acetylates GABPB1, impairing GABPB1 heterotetramerization and activity (By similarity). In addition to protein acetyltransferase, can use different acyl-CoA substrates, such as (2E)-butenoyl-CoA (crotonyl-CoA), butanoyl-CoA (butyryl-CoA), 2-hydroxyisobutanoyl-CoA (2-hydroxyisobutyryl-CoA) or propanoyl-CoA (propionyl-CoA), and is able to mediate protein crotonylation, butyrylation, 2-hydroxyisobutyrylation or propionylation, respectively (PubMed:17267393, PubMed:25818647, PubMed:29775581). Acts as a histone crotonyltransferase; crotonylation marks active promoters and enhancers and confers resistance to transcriptional repressors (PubMed:25818647). Histone crotonyltransferase activity is dependent on the concentration of (2E)-butenoyl-CoA (crotonyl-CoA) substrate and such activity is weak when (2E)-butenoyl-CoA (crotonyl-CoA) concentration is low (PubMed:25818647). Also acts as a histone butyryltransferase; butyrylation marks active promoters (PubMed:17267393). Acts as a protein-lysine 2-hydroxyisobutyryltransferase; regulates glycolysis by mediating 2-hydroxyisobutyrylation of glycolytic enzymes (PubMed:29775581). Functions as a transcriptional coactivator for SMAD4 in the TGF-beta signaling pathway (PubMed:25514493). Acetylates PCK1 and promotes PCK1 anaplerotic activity (PubMed:30193097). Acetylates RXRA and RXRG (PubMed:17761950). {ECO:0000250|UniProtKB:B2RWS6, ECO:0000269|PubMed:10733570, ECO:0000269|PubMed:11430825, ECO:0000269|PubMed:11701890, ECO:0000269|PubMed:12402037, ECO:0000269|PubMed:12586840, ECO:0000269|PubMed:12929931, ECO:0000269|PubMed:14645221, ECO:0000269|PubMed:15186775, ECO:0000269|PubMed:15890677, ECO:0000269|PubMed:16617102, ECO:0000269|PubMed:16762839, ECO:0000269|PubMed:17267393, ECO:0000269|PubMed:17761950, ECO:0000269|PubMed:18722353, ECO:0000269|PubMed:18995842, ECO:0000269|PubMed:20810990, ECO:0000269|PubMed:21030595, ECO:0000269|PubMed:23415232, ECO:0000269|PubMed:23911289, ECO:0000269|PubMed:23934153, ECO:0000269|PubMed:24939902, ECO:0000269|PubMed:25514493, ECO:0000269|PubMed:25818647, ECO:0000269|PubMed:29775581, ECO:0000269|PubMed:30193097, ECO:0000269|PubMed:8945521, ECO:0000305|PubMed:20955178}.; (Microbial infection) In case of HIV-1 infection, it is recruited by the viral protein Tat. Regulates Tat's transactivating activity and may help inducing chromatin remodeling of proviral genes. Binds to and may be involved in the transforming capacity of the adenovirus E1A protein. {ECO:0000269|PubMed:10545121, ECO:0000269|PubMed:11080476}. | R-HSA-1234158; R-HSA-1368082; R-HSA-156711; R-HSA-1912408; R-HSA-1989781; R-HSA-201722; R-HSA-210744; R-HSA-2122947; R-HSA-2197563; R-HSA-2644606; R-HSA-2894862; R-HSA-3134973; R-HSA-3214847; R-HSA-3371568; R-HSA-381340; R-HSA-3899300; R-HSA-400253; R-HSA-5250924; R-HSA-5617472; R-HSA-5621575; R-HSA-5689901; R-HSA-6781823; R-HSA-6781827; R-HSA-6782135; R-HSA-6782210; R-HSA-6804114; R-HSA-6804758; R-HSA-6804760; R-HSA-6811555; R-HSA-8866907; R-HSA-8936459; R-HSA-8939243; R-HSA-8941856; R-HSA-8941858; R-HSA-8951936; R-HSA-9013508; R-HSA-9013695; R-HSA-9018519; R-HSA-9029569; R-HSA-9031628; R-HSA-918233; R-HSA-933541; R-HSA-9614657; R-HSA-9616222; R-HSA-9617629; | NDF:1307, |
| Q13705 | AVR2B_HUMAN | Acvr2b | Activin receptor type-2B (EC 2.7.11.30) (Activin receptor type IIB) (ACTR-IIB) | 512 | Transmembrane serine/threonine kinase activin type-2 receptor forming an activin receptor complex with activin type-1 serine/threonine kinase receptors (ACVR1, ACVR1B or ACVR1c). Transduces the activin signal from the cell surface to the cytoplasm and is thus regulating many physiological and pathological processes including neuronal differentiation and neuronal survival, hair follicle development and cycling, FSH production by the pituitary gland, wound healing, extracellular matrix production, immunosuppression and carcinogenesis. Activin is also thought to have a paracrine or autocrine role in follicular development in the ovary. Within the receptor complex, the type-2 receptors act as a primary activin receptors (binds activin-A/INHBA, activin-B/INHBB as well as inhibin-A/INHA-INHBA). The type-1 receptors like ACVR1B act as downstream transducers of activin signals. Activin binds to type-2 receptor at the plasma membrane and activates its serine-threonine kinase. The activated receptor type-2 then phosphorylates and activates the type-1 receptor. Once activated, the type-1 receptor binds and phosphorylates the SMAD proteins SMAD2 and SMAD3, on serine residues of the C-terminal tail. Soon after their association with the activin receptor and subsequent phosphorylation, SMAD2 and SMAD3 are released into the cytoplasm where they interact with the common partner SMAD4. This SMAD complex translocates into the nucleus where it mediates activin-induced transcription. Inhibitory SMAD7, which is recruited to ACVR1B through FKBP1A, can prevent the association of SMAD2 and SMAD3 with the activin receptor complex, thereby blocking the activin signal. Activin signal transduction is also antagonized by the binding to the receptor of inhibin-B via the IGSF1 inhibin coreceptor. {ECO:0000269|PubMed:8622651}. | R-HSA-1181150; R-HSA-1433617; R-HSA-1502540; R-HSA-201451; | NDF:211, |
| Q14126 | DSG2_HUMAN | Dsg2 Cdhf5 | Desmoglein-2 (Cadherin family member 5) (HDGC) | 1118 | Component of intercellular desmosome junctions. Involved in the interaction of plaque proteins and intermediate filaments mediating cell-cell adhesion. | R-HSA-351906; R-HSA-6805567; R-HSA-6809371; | NYF:774, |
| Q14574 | DSC3_HUMAN | Dsc3 Cdhf3 Dsc4 | Desmocollin-3 (Cadherin family member 3) (Desmocollin-4) (HT-CP) | 896 | Component of intercellular desmosome junctions. Involved in the interaction of plaque proteins and intermediate filaments mediating cell-cell adhesion. May contribute to epidermal cell positioning (stratification) by mediating differential adhesiveness between cells that express different isoforms. | R-HSA-6805567; R-HSA-6809371; | QFF:331, NAF:367, |
| Q14686 | NCOA6_HUMAN | Ncoa6 Aib3 Kiaa0181 Rap250 Trbp | Nuclear receptor coactivator 6 (Activating signal cointegrator 2) (ASC-2) (Amplified in breast cancer protein 3) (Cancer-amplified transcriptional coactivator ASC-2) (Nuclear receptor coactivator RAP250) (NRC RAP250) (Nuclear receptor-activating protein, 250 kDa) (Peroxisome proliferator-activated receptor-interacting protein) (PPAR-interacting protein) (PRIP) (Thyroid hormone receptor-binding protein) | 2063 | Nuclear receptor coactivator that directly binds nuclear receptors and stimulates the transcriptional activities in a hormone-dependent fashion. Coactivates expression in an agonist- and AF2-dependent manner. Involved in the coactivation of different nuclear receptors, such as for steroids (GR and ERs), retinoids (RARs and RXRs), thyroid hormone (TRs), vitamin D3 (VDR) and prostanoids (PPARs). Probably functions as a general coactivator, rather than just a nuclear receptor coactivator. May also be involved in the coactivation of the NF-kappa-B pathway. May coactivate expression via a remodeling of chromatin and its interaction with histone acetyltransferase proteins. | R-HSA-1368082; R-HSA-1368108; R-HSA-1989781; R-HSA-2151201; R-HSA-2426168; R-HSA-381340; R-HSA-400206; R-HSA-400253; R-HSA-5617472; | NHF:844, NFA:1296, |
| Q14CN4 | K2C72_HUMAN | Krt72 K6irs2 Kb35 Krt6 Krt6irs2 | Keratin, type II cytoskeletal 72 (Cytokeratin-72) (CK-72) (Keratin-72) (K72) (Type II inner root sheath-specific keratin-K6irs2) (Type-II keratin Kb35) | 511 | Has a role in hair formation. Specific component of keratin intermediate filaments in the inner root sheath (IRS) of the hair follicle (Probable). {ECO:0000305}. | R-HSA-6805567; R-HSA-6809371; | KFF:258, |
| Q15910 | EZH2_HUMAN | Ezh2 Kmt6 | Histone-lysine N-methyltransferase EZH2 (EC 2.1.1.356) (ENX-1) (Enhancer of zeste homolog 2) (Lysine N-methyltransferase 6) | 746 | Polycomb group (PcG) protein. Catalytic subunit of the PRC2/EED-EZH2 complex, which methylates 'Lys-9' (H3K9me) and 'Lys-27' (H3K27me) of histone H3, leading to transcriptional repression of the affected target gene. Able to mono-, di- and trimethylate 'Lys-27' of histone H3 to form H3K27me1, H3K27me2 and H3K27me3, respectively. Displays a preference for substrates with less methylation, loses activity when progressively more methyl groups are incorporated into H3K27, H3K27me0 > H3K27me1 > H3K27me2 (PubMed:22323599, PubMed:30923826). Compared to EZH1-containing complexes, it is more abundant in embryonic stem cells and plays a major role in forming H3K27me3, which is required for embryonic stem cell identity and proper differentiation. The PRC2/EED-EZH2 complex may also serve as a recruiting platform for DNA methyltransferases, thereby linking two epigenetic repression systems. Genes repressed by the PRC2/EED-EZH2 complex include HOXC8, HOXA9, MYT1, CDKN2A and retinoic acid target genes. EZH2 can also methylate non-histone proteins such as the transcription factor GATA4 and the nuclear receptor RORA. Regulates the circadian clock via histone methylation at the promoter of the circadian genes. Essential for the CRY1/2-mediated repression of the transcriptional activation of PER1/2 by the CLOCK-ARNTL/BMAL1 heterodimer; involved in the di and trimethylation of 'Lys-27' of histone H3 on PER1/2 promoters which is necessary for the CRY1/2 proteins to inhibit transcription. {ECO:0000269|PubMed:14532106, ECO:0000269|PubMed:15225548, ECO:0000269|PubMed:15231737, ECO:0000269|PubMed:15385962, ECO:0000269|PubMed:16179254, ECO:0000269|PubMed:16357870, ECO:0000269|PubMed:16618801, ECO:0000269|PubMed:16717091, ECO:0000269|PubMed:16936726, ECO:0000269|PubMed:17210787, ECO:0000269|PubMed:17344414, ECO:0000269|PubMed:18285464, ECO:0000269|PubMed:19026781, ECO:0000269|PubMed:20935635, ECO:0000269|PubMed:22323599, ECO:0000269|PubMed:23063525, ECO:0000269|PubMed:24474760, ECO:0000269|PubMed:30923826}. | R-HSA-212300; R-HSA-2559580; R-HSA-3214841; R-HSA-5617472; R-HSA-8943724; R-HSA-8953750; R-HSA-9609690; | LFF:722, NDF:671, |
| Q3LHN0 | KR251_HUMAN | Krtap25-1 Kap25.1 | Keratin-associated protein 25-1 | 102 | In the hair cortex, hair keratin intermediate filaments are embedded in an interfilamentous matrix, consisting of hair keratin-associated proteins (KRTAP), which are essential for the formation of a rigid and resistant hair shaft through their extensive disulfide bond cross-linking with abundant cysteine residues of hair keratins. The matrix proteins include the high-sulfur and high-glycine-tyrosine keratins (By similarity). {ECO:0000250}. | R-HSA-6805567; | FFF:8, |
| Q3SY84 | K2C71_HUMAN | Krt71 K6irs1 Kb34 Krt6irs1 | Keratin, type II cytoskeletal 71 (Cytokeratin-71) (CK-71) (Keratin-71) (K71) (Type II inner root sheath-specific keratin-K6irs1) (Keratin 6 irs) (hK6irs) (hK6irs1) (Type-II keratin Kb34) | 523 | Plays a central role in hair formation. Essential component of keratin intermediate filaments in the inner root sheath (IRS) of the hair follicle. {ECO:0000269|PubMed:22592156}. | R-HSA-6805567; R-HSA-6809371; | KFF:263, |
| Q5VXI9 | LIPN_HUMAN | Lipn Lipl4 | Lipase member N (EC 3.1.1.-) (Lipase-like abhydrolase domain-containing protein 4) | 398 | Plays a highly specific role in the last step of keratinocyte differentiation. May have an essential function in lipid metabolism of the most differentiated epidermal layers. {ECO:0000269|PubMed:17562024}. | R-HSA-6809371; | NHF:372, NFA:196, |
| Q5VXJ0 | LIPK_HUMAN | Lipk Lipl2 | Lipase member K (EC 3.1.1.-) (Lipase-like abhydrolase domain-containing protein 2) | 399 | Plays a highly specific role in the last step of keratinocyte differentiation. May have an essential function in lipid metabolism of the most differentiated epidermal layers. {ECO:0000269|PubMed:17562024}. | R-HSA-6809371; | NFI:155, |
| Q5VYY2 | LIPM_HUMAN | Lipm Lipl3 | Lipase member M (EC 3.1.1.-) (Lipase-like abhydrolase domain-containing protein 3) | 423 | Plays a highly specific role in the last step of keratinocyte differentiation. May have an essential function in lipid metabolism of the most differentiated epidermal layers. {ECO:0000269|PubMed:17562024}. | R-HSA-6809371; | NFI:169, |
| Q6A163 | K1C39_HUMAN | Krt39 Ka35 | Keratin, type I cytoskeletal 39 (Cytokeratin-39) (CK-39) (Keratin-39) (K39) (Type I hair keratin Ka35) | 491 | May play a role in late hair differentiation. | R-HSA-6805567; R-HSA-6809371; | NNF:74, |
| Q7Z794 | K2C1B_HUMAN | Krt77 Krt1b | Keratin, type II cytoskeletal 1b (Cytokeratin-1B) (CK-1B) (Keratin-77) (K77) (Type-II keratin Kb39) | 578 |  | R-HSA-6805567; R-HSA-6809371; | NDF:266, |
| Q86SJ6 | DSG4_HUMAN | Dsg4 Cdhf13 | Desmoglein-4 (Cadherin family member 13) | 1040 | Component of intercellular desmosome junctions. Involved in the interaction of plaque proteins and intermediate filaments mediating cell-cell adhesion. Coordinates the transition from proliferation to differentiation in hair follicle keratinocytes (By similarity). {ECO:0000250}. | R-HSA-6805567; R-HSA-6809371; | LFF:4, NIF:496, NYF:874, |
| Q86Y46 | K2C73_HUMAN | Krt73 K6irs3 Kb36 Krt6irs3 | Keratin, type II cytoskeletal 73 (Cytokeratin-73) (CK-73) (Keratin-73) (K73) (Type II inner root sheath-specific keratin-K6irs3) (Type-II keratin Kb36) | 540 | Has a role in hair formation. Specific component of keratin intermediate filaments in the inner root sheath (IRS) of the hair follicle (Probable). {ECO:0000305}. | R-HSA-6805567; R-HSA-6809371; | KFF:265, |
| Q8N1N4 | K2C78_HUMAN | Krt78 K5b Kb40 | Keratin, type II cytoskeletal 78 (Cytokeratin-78) (CK-78) (Keratin-5b) (Keratin-78) (K78) (Type-II keratin Kb40) | 520 |  | R-HSA-6805567; R-HSA-6809371; | NDF:213, |
| Q8NER5 | ACV1C_HUMAN | Acvr1c Alk7 | Activin receptor type-1C (EC 2.7.11.30) (Activin receptor type IC) (ACTR-IC) (Activin receptor-like kinase 7) (ALK-7) | 493 | Serine/threonine protein kinase which forms a receptor complex on ligand binding. The receptor complex consisting of 2 type II and 2 type I transmembrane serine/threonine kinases. Type II receptors phosphorylate and activate type I receptors which autophosphorylate, then bind and activate SMAD transcriptional regulators, SMAD2 and SMAD3. Receptor for activin AB, activin B and NODAL. Plays a role in cell differentiation, growth arrest and apoptosis. {ECO:0000269|PubMed:12063393, ECO:0000269|PubMed:15531507}. | R-HSA-1181150; R-HSA-1433617; R-HSA-1502540; | NIF:381, |
| Q8NEZ4 | KMT2C_HUMAN | Kmt2c Halr Kiaa1506 Mll3 | Histone-lysine N-methyltransferase 2C (Lysine N-methyltransferase 2C) (EC 2.1.1.354) (Homologous to ALR protein) (Myeloid/lymphoid or mixed-lineage leukemia protein 3) | 4911 | Histone methyltransferase that methylates 'Lys-4' of histone H3 (PubMed:22266653). H3 'Lys-4' methylation represents a specific tag for epigenetic transcriptional activation. Central component of the MLL2/3 complex, a coactivator complex of nuclear receptors, involved in transcriptional coactivation. KMT2C/MLL3 may be a catalytic subunit of this complex. May be involved in leukemogenesis and developmental disorder. {ECO:0000269|PubMed:17500065, ECO:0000269|PubMed:22266653}. | R-HSA-3214841; R-HSA-5617472; R-HSA-8936459; | QFF:356, NFI:2592, |
| Q92817 | EVPL_HUMAN | Evpl | Envoplakin (210 kDa cornified envelope precursor protein) (210 kDa paraneoplastic pemphigus antigen) (p210) | 2033 | Component of the cornified envelope of keratinocytes. May link the cornified envelope to desmosomes and intermediate filaments. | R-HSA-6809371; | NNF:708, |
| Q96FX8 | PERP_HUMAN | Perp Kcp1 Krtcap1 Pigpc1 Thw | p53 apoptosis effector related to PMP-22 (Keratinocyte-associated protein 1) (KCP-1) (P53-induced protein PIGPC1) (Transmembrane protein THW) | 193 | Component of intercellular desmosome junctions. Plays a role in stratified epithelial integrity and cell-cell adhesion by promoting desmosome assembly. Plays a role as an effector in the TP53-dependent apoptotic pathway (By similarity). {ECO:0000250}. | R-HSA-6803205; R-HSA-6809371; | FFF:168, |
| Q99569 | PKP4_HUMAN | Pkp4 | Plakophilin-4 (p0071) | 1192 | Plays a role as a regulator of Rho activity during cytokinesis. May play a role in junctional plaques. {ECO:0000269|PubMed:17115030}. | R-HSA-6805567; R-HSA-6809371; | NHF:1009, |
| Q99814 | EPAS1_HUMAN | Epas1 Bhlhe73 Hif2a Mop2 Pasd2 | Endothelial PAS domain-containing protein 1 (EPAS-1) (Basic-helix-loop-helix-PAS protein MOP2) (Class E basic helix-loop-helix protein 73) (bHLHe73) (HIF-1-alpha-like factor) (HLF) (Hypoxia-inducible factor 2-alpha) (HIF-2-alpha) (HIF2-alpha) (Member of PAS protein 2) (PAS domain-containing protein 2) | 870 | Transcription factor involved in the induction of oxygen regulated genes. Heterodimerizes with ARNT; heterodimer binds to core DNA sequence 5'-TACGTG-3' within the hypoxia response element (HRE) of target gene promoters (By similarity). Regulates the vascular endothelial growth factor (VEGF) expression and seems to be implicated in the development of blood vessels and the tubular system of lung. May also play a role in the formation of the endothelium that gives rise to the blood brain barrier. Potent activator of the Tie-2 tyrosine kinase expression. Activation requires recruitment of transcriptional coactivators such as CREBBP and probably EP300. Interaction with redox regulatory protein APEX seems to activate CTAD (By similarity). {ECO:0000250, ECO:0000250|UniProtKB:P97481}. | R-HSA-1234158; R-HSA-1234174; R-HSA-1234176; R-HSA-452723; R-HSA-8849473; R-HSA-8951664; R-HSA-9664873; | DFF:167, NIF:569, |
| Q99959 | PKP2_HUMAN | Pkp2 | Plakophilin-2 | 881 | May play a role in junctional plaques. {ECO:0000269|PubMed:22781308}. | R-HSA-6805567; R-HSA-6809371; | NDF:95, |
| Q9NQ38 | ISK5_HUMAN | Spink5 | Serine protease inhibitor Kazal-type 5 (Lympho-epithelial Kazal-type-related inhibitor) (LEKTI) [Cleaved into: Hemofiltrate peptide HF6478; Hemofiltrate peptide HF7665] | 1064 | Serine protease inhibitor, probably important for the anti-inflammatory and/or antimicrobial protection of mucous epithelia. Contribute to the integrity and protective barrier function of the skin by regulating the activity of defense-activating and desquamation-involved proteases. Inhibits KLK5, it's major target, in a pH-dependent manner. Inhibits KLK7, KLK14 CASP14, and trypsin. {ECO:0000269|PubMed:10419450, ECO:0000269|PubMed:17596512, ECO:0000269|PubMed:20533828}. | R-HSA-6809371; | KFF:49, |
| Q9NSC2 | SALL1_HUMAN | Sall1 Sal1 Znf794 | Sal-like protein 1 (Spalt-like transcription factor 1) (Zinc finger protein 794) (Zinc finger protein SALL1) (Zinc finger protein Spalt-1) (HSal1) (Sal-1) | 1324 | Transcriptional repressor involved in organogenesis. Plays an essential role in ureteric bud invasion during kidney development. {ECO:0000250|UniProtKB:Q9ER74}. | R-HSA-2892247; | NFR:1308, |
| Q9Y5Q3 | MAFB_HUMAN | Mafb Krml | Transcription factor MafB (Maf-B) (V-maf musculoaponeurotic fibrosarcoma oncogene homolog B) | 323 | Acts as a transcriptional activator or repressor (PubMed:27181683). Plays a pivotal role in regulating lineage-specific hematopoiesis by repressing ETS1-mediated transcription of erythroid-specific genes in myeloid cells. Required for monocytic, macrophage, osteoclast, podocyte and islet beta cell differentiation. Involved in renal tubule survival and F4/80 maturation. Activates the insulin and glucagon promoters. Together with PAX6, transactivates weakly the glucagon gene promoter through the G1 element. SUMO modification controls its transcriptional activity and ability to specify macrophage fate. Binds element G1 on the glucagon promoter (By similarity). Involved either as an oncogene or as a tumor suppressor, depending on the cell context. Required for the transcriptional activation of HOXB3 in the rhombomere r5 in the hindbrain (By similarity). {ECO:0000250|UniProtKB:P54841, ECO:0000269|PubMed:19143053, ECO:0000269|PubMed:27181683}. | R-HSA-5617472; | NDF:22, |
| Q9Y5Y6 | ST14_HUMAN | St14 Prss14 Snc19 Tadg15 | **Suppressor of tumorigenicity 14 protein** | 855 | Degrades extracellular matrix. Proposed to play a role in breast cancer invasion and metastasis. Exhibits trypsin-like activity as defined by cleavage of synthetic substrates with Arg or Lys as the P1 site. Involved in the terminal differentiation of keratinocytes through prostasin (PRSS8) activation and filaggrin (FLG) processing. {ECO:0000269|PubMed:18843291}. | R-HSA-6809371; | KFF:383, NDF:704, |

**Table S2.**

| **Supplementary Table 2:** CRC stemness-related gene sets used in this study. | | | | | |
| --- | --- | --- | --- | --- | --- |
| **Pubmed ID** | **Publications** | **CRC stemness-related gene set** | **Number of Genes** | **Gene** | |
| 39246444 | Verhagen MP, Xu T, Stabile R, Joosten R, Tucci FA, van Royen M, Trerotola M, Alberti S, Sacchetti A, Fodde R. The SW480 cell line as a model of resident and migrating colon cancer stem cells. iScience. 2024 Aug 5;27 | resident cancer stem cells  (rCSCs) | 1571 | A1CF, A2M, ABCA3, ABCA4, ABCB1, ABCB10, ABCC2, ABCG1, ABHD1, ABHD17C, ABLIM2, ACADL, ACAP3, ACP4, ACSBG1, ACSS3, ACTN2, ACVR1C, ADAM23, ADAM32, ADAMTS12, ADAMTS19, ADAMTS2, ADAMTS6, ADAMTSL1, ADAMTSL2, ADAP1, ADARB2, ADAT2, ADCY1, ADCY10, ADCY2, ADCY5, ADGRB1, ADGRD1, ADGRG3, ADGRG5, ADGRL2, ADGRL3, ADGRV1, ADH1C, ADHFE1, ADM2, ADORA2A, ADRA2B, AEBP1, AFAP1L2, AGMAT, AGMO, AGPAT4, AGT, AHRR, AIFM3, AKAP3, AKAP6, AKR7L, ALB, ALDH1A2, ALDH1L2, ALDOB, ALOX5, ALOX5AP, ALPI, ALPK3, ALX4, AMBN, AMBP, AMER1, AMOT, AMZ1, ANGPT1, ANK3, ANKRD13B, ANKRD29, ANKRD34B, ANKRD46, ANKRD65, ANKS4B, AOAH, APBA2, APOBEC2, APOO, AQP3, ARG1, ARHGAP22, ARHGAP24, ARHGAP25, ARHGAP28, ARHGAP30, ARHGDIB, ARHGEF10L, ARL10, ARL4A, ARMC2, ARMCX2, ARRDC4, ARV1, ASCL5, ASIP, ASNS, ASPRV1, ASPSCR1, ASS1, ASTL, ASXL3, ATOH7, ATP10D, ATP13A5, ATP1A3, ATP2B2, ATP4A, ATP8A1, ATP8B1, ATP8B2, ATP8B4, ATXN7L3B, AUTS2, AVPR2, AZGP1, B3GALNT1, B3GAT2, B3GNT4, B4GALNT3, B4GALNT4, BAAT, BACH2, BAHCC1, BAIAP2L2, BANF2, BANK1, BARHL1, BASP1, BATF3, BCAT1, BCHE, BCL11A, BCL11B, BCL2, BCL2L11, BCO2, BDKRB2, BEST3, BIN1, BLK, BMP2, BMP5, BMP6, BMPR1B, BPGM, BPNT1, BRINP1, BTN3A1, BZW2, C10orf82, C10orf95, C11orf42, C12orf60, C15orf61, C19orf44, C1orf115, C1orf127, C1orf21, C1QTNF12, C1QTNF3, C2, C20orf204, C22orf15, C6orf118, CA6, CA8, CAB39L, CABLES1, CABP1, CABP4, CACNA1B, CACNA1C, CACNA1D, CACNA1E, CACNA1H, CACNA2D1, CACNB2, CADPS2, CALCB, CALHM1, CAMK4, CAMKK1, CAMKV, CAMSAP3, CAPN13, CAPN9, CASKIN1, CATSPER3, CAVIN4, CBLB, CBLN2, CCBE1, CCDC13, CCDC149, CCDC167, CCDC169, CCDC177, CCDC180, CCDC187, CCDC196, CCDC27, CCDC33, CCDC77, CCDC78, CCDC81, CCDC85A, CCM2L, CCNB1IP1, CCNB3, CCND2, CCSER1, CD101, CD226, CD320, CD36, CD5, CD6, CD8A, CD8B, CD8B2, CD93, CDH12, CDH16, CDH17, CDH18, CDH8, CDH9, CDHR1, CDHR2, CDK6, CDKL1, CDX1, CDYL2, CEACAM19, CEACAM20, CEACAM21, CEBPA, CELF2, CELF4, CEMIP, CEND1, CENPS, CERKL, CERS3, CES1, CES3, CFAP74, CFAP92, CFTR, CGNL1, CGREF1, CH25H, CHEK2, CHL1, CHN2, CHODL, CHRDL1, CHRFAM7A, CHRM3, CHRNA1, CHRNA3, CHRNA4, CHRNA7, CHRNG, CHST8, CHSY3, CIAO3, CILK1, CKM, CLDN19, CLDN8, CLIC6, CLRN3, CLYBL, CMKLR1, CMYA5, CNBD2, CNR1, CNTN1, CNTN4, CNTNAP2, CNTNAP4, COCH, COL14A1, COL15A1, COL1A2, COL20A1, COL4A1, COL4A5, COL4A6, COL5A2, COLCA2, COLQ, COMTD1, CORT, CPA2, CPE, CPNE7, CPVL, CPXM2, CR2, CREB5, CREG1, CRHR2, CRISPLD1, CROCC2, CRYBG1, CSGALNACT1, CSK, CSMD1, CSMD3, CSRNP3, CSRP2, CTCFL, CTSC, CTSH, CTSO, CTTNBP2, CUBN, CXCR1, CXCR3, CXXC1, CXXC4, CYB5A, CYBB, CYP11A1, CYP1A1, CYP26B1, CYP2E1, CYP2W1, CYP4F2, CYP4F3, DAAM2, DACH1, DAGLA, DBH, DCAF8L1, DCAF8L2, DDIT4, DDIT4L, DDN, DDR2, DECR1, DEF6, DEFA5, DEFA6, DEPTOR, DFFB, DGAT2, DHFR2, DIO2, DIO3, DIPK1B, DISP3, DIXDC1, DKK4, DLGAP1, DLGAP2, DLL3, DLL4, DLX1, DLX3, DMBT1, DMD, DMPK, DMRT1, DMRT2, DNAH10, DNAH11, DNAH14, DNAH6, DNAH7, DNAH9, DNAJC12, DNAJC5G, DNALI1, DNER, DNHD1, DNMT3B, DOC2B, DOK6, DPEP1, DPP6, DPYD, DPYSL3, DPYSL4, DRD2, DSC3, DSCAM, DSEL, DSG3, DTNA, DTX1, DUSP2, DUSP9, DVL1, DYNC1I1, DYRK1B, DYRK2, DYSF, DZIP1, EBF3, ECRG4, ECSIT, EEPD1, EFCAB10, EFR3B, EGFLAM, EHD3, ELAVL2, ELF3, ELMO1, ELMOD1, ELN, ELOVL6, EML1, EMX1, EMX2, ENAH, ENOX1, ENPEP, ENPP3, ENTPD1, EOMES, EPB41L3, EPHA3, EPHA5, EPHA8, EPHX3, ERBB4, ERMP1, ESRRB, ESRRG, ESYT3, ETNPPL, EVL, EVX1, EVX2, EYA1, EYA2, FABP5, FABP6, FAM124A, FAM131C, FAM13A, FAM13C, FAM151A, FAM163B, FAM167A, FAM174B, FAM178B, FAM184A, FAM189A1, FAM204A, FAM229B, FAM3B, FAM78B, FAM81A, FAM83B, FAM83E, FAM86B1, FAM86B2, FAM90A1, FAM90A20P, FAM9B, FANCF, FAT3, FAT4, FBLN2, FBN1, FBN2, FBRSL1, FBXL7, FBXO16, FBXO43, FBXW12, FDFT1, FECH, FEM1A, FGF20, FGF21, FGF9, FGFBP3, FGFR4, FGFRL1, FGGY, FGL2, FHIT, FKBP7, FLI1, FLRT3, FLT4, FMNL2, FMOD, FNDC10, FNDC7, FOLR1, FOXA1, FOXA2, FOXA3, FOXN1, FOXN4, FOXP1, FOXP2, FRAT2, FREM1, FREM2, FRK, FRRS1, FSCN3, FSIP2, FSTL4, FTCD, FXYD3, FYB2, FZD10, FZD8, G0S2, GABRA2, GABRB2, GABRB3, GABRG1, GABRR2, GAL, GAL3ST1, GALNT15, GALNT9, GAP43, GAREM1, GARIN1B, GAS2, GATA5, GATM, GBP2, GBP5, GDPD1, GDPD3, GEMIN2, GFPT2, GGT1, GGT7, GIPC2, GJA1, GJC3, GLIPR1, GLT1D1, GLYAT, GNAI1, GNAT1, GNAZ, GNB1L, GNG2, GNG4, GNRHR, GOLGA8A, GPAT2, GPC2, GPC3, GPC5, GPC6, GPER1, GPHA2, GPM6B, GPR119, GPR153, GPR155, GPR157, GPR182, GPR39, GPR62, GPR83, GPRIN3, GPT, GPT2, GPX2, GRAMD1B, GRAMD2A, GRB14, GREB1, GRIA4, GRIK1, GRIK2, GRIN2B, GRIN2C, GRM3, GRM4, GRM8, GSE1, GTF2IRD1, GUCY1A2, HACD4, HAGHL, HAL, HAO2, HAS3, HBA1, HBA2, HCN4, HCRTR2, HDC, HDDC3, HEATR4, HECW2, HELT, HES2, HES5, HES6, HEY1, HEY2, HEYL, HFM1, HGD, HGFAC, HHIPL2, HIPK2, HK2, HLA-DOB, HLA-DQB1, HLA-DRB1, HLF, HLX, HMCN1, HMGCLL1, HMGCS1, HMGN5, HMX2, HNF1A, HNF4A, HNF4G, HOXA10, HOXA13, HOXA2, HOXA3, HOXA5, HOXA6, HOXA9, HOXC4, HOXC5, HOXD13, HOXD9, HPCAL4, HPDL, HRH2, HS3ST3B1, HS3ST6, HS6ST3, HSD11B2, HSD17B13, HSD17B7, HSPA12A, HSPA12B, HTR2C, HYKK, ID1, ID4, IDH2, IFITM10, IGDCC3, IGF2, IGFALS, IGFBPL1, IGFN1, IGHV7-81, IGSF1, IGSF10, IGSF11, IGSF23, IGSF9, IKZF2, IKZF3, IL12A, IL17RB, IL1R2, IL31RA, IL9R, IMPA2, INHBE, INSC, INSM1, INSYN1, IQCH, IRAK1BP1, IRF4, IRGM, IRX2, ISL2, ISLR, ISOC1, ITGB7, IZUMO1, JAG2, JAKMIP1, JAKMIP2, JPH1, JPH4, KBTBD11, KCNA5, KCNAB2, KCNE4, KCNG2, KCNH2, KCNH3, KCNJ11, KCNJ2, KCNJ3, KCNK12, KCNK2, KCNK9, KCNMA1, KCNN1, KCNN2, KCNN3, KCNQ4, KCNS3, KCNT1, KCNV1, KCNV2, KCTD12, KCTD19, KCTD3, KDM4C, KEL, KIAA0408, KIAA1328, KIAA1549L, KIF19, KIF21B, KIF26A, KIF5A, KIF5C, KIF6, KIT, KITLG, KLF8, KLHL13, KLHL14, KLHL17, KLHL31, KLHL32, KLHL4, KLK14, KLRC1, KLRC2, KLRC3, KLRC4, KLRG1, KLRG2, KLRK1, KPTN, KRBA1, KREMEN1, KRT17, KRT20, KRT36, KRT40, KRT75, KRTAP1-1, KRTAP3-2, KRTAP3-3, L3MBTL3, L3MBTL4, LAMA2, LAMC3, LANCL3, LDB2, LDOC1, LEAP2, LFNG, LGALS7, LGALS7B, LGI2, LGR5, LGSN, LHFPL1, LHX3, LIAS, LILRA6, LILRB3, LIN7A, LINGO1, LINGO2, LINGO3, LIPC, LIX1, LKAAEAR1, LMNTD1, LNP1, LNX1, LPAR1, LPAR3, LPCAT1, LPL, LRAT, LRATD1, LRFN4, LRGUK, LRIG3, LRIT3, LRP3, LRRC14B, LRRC17, LRRC31, LRRC49, LRRC4C, LRRC63, LRRC7, LRRC9, LRRIQ1, LRRIQ3, LRRK2, LRRN1, LRRN2, LRRTM4, LRTM2, LSAMP, LSP1, LTA4H, LUM, LURAP1L, LVRN, LYG2, LYRM7, MAB21L3, MACC1, MAF, MAGEA8, MAGEC1, MAGEC2, MAGEC3, MAGEH1, MALRD1, MAN1A1, MAN2A1, MANEA, MAP1A, MAP2, MAP2K6, MAP3K21, MAPK15, MAPK4, MAPRE2, MARCKSL1, MARK1, MASP1, MAST1, MAT1A, MAZ, MBLAC2, MC4R, MCF2, MCF2L, MCRIP2, MCTP1, MDGA2, ME2, MECOM, MEF2C, MEGF11, MEGF6, MEIS1, MEP1B, MEST, MET, METTL7A, MEX3A, MEX3B, MFAP2, MFNG, MFSD4A, MGAT4A, MGST1, MICU3, MINAR1, MINAR2, MIPOL1, MIR200A, MIR200B, MIXL1, MKRN2OS, MLC1, MLLT11, MLXIPL, MMACHC, MME, MMP11, MMP16, MMP17, MMP21, MMP23B, MOCS1, MOGAT1, MPDZ, MPPED2, MROH5, MSX2, MT1G, MTARC1, MTARC2, MTFP1, MTHFD2, MTNR1A, MTTP, MTUS2, MUC5AC, MUC5B, MUC6, MVB12B, MXD4, MYB, MYC, MYCN, MYH7B, MYMK, MYO3B, MYO7A, MYOM1, MYRFL, MYRIP, MYT1, N4BP3, NA, NAALAD2, NAALADL1, NAP1L2, NAT8L, NCAM2, NCKAP5, NDST3, NECAB2, NECTIN1, NECTIN4, NEGR1, NETO1, NEU4, NEUROD1, NEXMIF, NFIA, NHSL2, NKAIN1, NKD1, NKD2, NKX2-1, NKX2-3, NKX2-5, NLGN1, NLRC3, NLRP14, NMB, NMNAT3, NOL4, NOTCH1, NOTCH3, NOTUM, NOVA1, NPC1L1, NPHP4, NPM3, NPR3, NPW, NPY4R, NR3C2, NR4A2, NR5A1, NR6A1, NRCAM, NREP, NRG4, NRN1, NRXN1, NRXN2, NRXN3, NTHL1, NTN3, NTNG1, NTS, NUDT11, NUTM2A, NUTM2B, NUTM2D, NUTM2E, NXF3, NXPH3, OCA2, OLFM2, OLFML3, ONECUT1, ONECUT2, OPCML, OPRD1, OPRK1, OPRL1, OR2H2, OR2T33, OR2T8, OR2W3, OTOF, OTOG, OTOGL, OTUD7A, OTX1, OVGP1, OVOL1, P2RX2, P2RX6, P2RY1, PACRG, PAIP2B, PAK1, PANK1, PARD6G, PAX3, PAX7, PAX9, PBX1, PCARE, PCBP3, PCDH10, PCDH15, PCDH17, PCDH19, PCDH7, PCDH9, PCDHB10, PCDHB11, PCDHB12, PCDHB13, PCDHB14, PCDHB16, PCDHB5, PCDHB6, PCDHB7, PCDHB9, PCDHGA1, PCDHGA2, PCDHGA4, PCDHGA5, PCDHGA7, PCDHGA9, PCDHGB1, PCDHGB6, PCK1, PCOLCE2, PCSK9, PCYT1B, PDCD4, PDE3A, PDE3B, PDE5A, PDE6C, PDGFC, PDGFD, PDK3, PDZD8, PELI1, PEX5, PGBD5, PGC, PHACTR1, PHEX, PHYHIPL, PI15, PID1, PIF1, PIK3R3, PIPOX, PITPNM3, PIWIL4, PKDREJ, PKHD1, PKHD1L1, PKIA, PLA1A, PLA2G12A, PLA2G3, PLA2G4E, PLAAT1, PLAC8L1, PLAG1, PLCB1, PLCH2, PLCL2, PLD5, PLEKHF1, PLEKHG4, PLEKHG5, PLOD2, PLPPR1, PLXDC2, PLXNA2, PMFBP1, PNLIPRP2, PNMT, POF1B, PON1, POSTN, POU2AF1, POU2AF2, POU2F2, POU3F2, POU3F3, POU6F2, PPAN, PPEF1, PPIF, PPM1L, PPP1R1B, PRDM16, PRDM8, PRDM9, PRIMA1, PRKAG3, PRKCQ, PRKCZ, PRKG1, PRKG2, PRKN, PRMT6, PROK2, PROM1, PROM2, PROS1, PROX1, PRR15L, PRR5, PRRT4, PRRX1, PRSS12, PRSS2, PRTG, PRUNE2, PRXL2B, PSD2, PSD3, PSTPIP1, PTAFR, PTBP2, PTCH2, PTCHD1, PTCHD4, PTGDR2, PTGR3, PTHLH, PTN, PTP4A3, PTPN13, PTPRC, PTPRD, PTPRM, PTPRN, PTPRO, PTPRT, PWWP3B, PYCR1, PZP, QRFP, RAB11FIP2, RAB25, RAB38, RAB3A, RAB3C, RAD51AP2, RADX, RAPGEFL1, RARB, RASGRF2, RASL11B, RASSF6, RASSF9, RBBP8NL, RBFA, RBM11, RBM20, RBM34, RCOR2, RCSD1, RDH13, RDH16, RETREG1, RFLNA, RGMA, RGS16, RGS2, RGS6, RHBDL1, RHOBTB1, RHOV, RIMBP2, RIMKLA, RIMS1, RNF122, RNF128, RNF133, RNF138, RNF144B, RNF148, RNF152, RNF223, RNVU1-27, ROBO1, ROBO2, RP1L1, RPL13A, RPP21, RPP25, RPRD1A, RPRM, RPS6KA6, RSPH4A, RSPO2, RTL5, RTL9, RTN1, RUNDC3B, RUNX1T1, RUNX3, RYR2, S100A14, SALL2, SALL3, SAMD11, SAMD5, SARDH, SAT1, SATB1, SBK1, SBSPON, SCART1, SCD, SCG5, SCGB3A1, SCIN, SCLY, SCML2, SCN1A, SCN2A, SCN3A, SCN9A, SDK2, SEC11C, SEC14L5, SELENOP, SEMA3A, SEMA3C, SEMA3D, SEMA3E, SEMA3F, SEMA3G, SEMA4C, SEMA4G, SEMA6A, SEMA6D, SERPINI1, SETBP1, SGCA, SGCD, SGCZ, SGK2, SGPP2, SH2B2, SH2D4B, SH2D7, SH3BGRL, SH3GL2, SH3RF3, SH3TC1, SHC3, SHF, SIK1, SIRT4, SIT1, SIX2, SKOR1, SLAIN1, SLC10A4, SLC10A5, SLC16A12, SLC16A14, SLC16A7, SLC16A9, SLC18A2, SLC18B1, SLC19A2, SLC19A3, SLC1A1, SLC22A1, SLC22A3, SLC25A21, SLC25A33, SLC27A5, SLC29A2, SLC2A13, SLC2A4, SLC2A7, SLC30A3, SLC35F4, SLC38A3, SLC38A8, SLC39A10, SLC39A5, SLC43A1, SLC43A3, SLC44A3, SLC44A5, SLC45A1, SLC45A2, SLC4A10, SLC4A5, SLC5A6, SLC6A19, SLC6A3, SLC6A7, SLC6A9, SLC7A2, SLC7A4, SLC9B2, SLCO3A1, SLCO4C1, SLCO5A1, SLCO6A1, SLFNL1, SLIT2, SLIT3, SLITRK5, SLITRK6, SMAD4, SMCO2, SMIM10L2A, SMIM17, SMOC1, SMOC2, SMTNL2, SMYD3, SNAI1, SNAI2, SNCAIP, SNORA14B, SNORA26, SNORD100, SNORD101, SNORD15B, SNORD99, SNTB1, SNX20, SNX22, SOAT2, SOBP, SORBS2, SORCS1, SORCS2, SORCS3, SORD, SOSTDC1, SOX1, SOX14, SOX5, SOX6, SP7, SP8, SPAG17, SPATA17, SPATC1, SPIB, SPINK5, SPIRE2, SPN, SPPL2B, SPSB4, SPTBN2, SRGAP3, SSBP2, SSBP4, SSPN, SSTR1, SSTR4, SSUH2, ST3GAL5, ST6GAL2, ST8SIA4, ST8SIA5, STAB2, STARD10, STK38L, STOM, STON2, STRA6, STRC, STUM, STYXL2, SUGCT, SULF1, SULT1A1, SULT2B1, SULT4A1, SV2B, SYCE2, SYNE1, SYNE4, SYNPO2, SYP, SYT11, SYT2, SYT6, SYT7, SYT9, TAF4B, TAFA2, TAGAP, TAL1, TARBP1, TAS1R1, TAS1R3, TAS2R10, TATDN3, TBC1D24, TBC1D32, TBX15, TBXAS1, TCAP, TCF23, TCF4, TCP10L, TDG, TDRD1, TECTA, TENM1, TENM4, TERT, TET1, TET2, TEX13C, TEX15, TEX45, TFF3, THBD, THBS4, THRB, THUMPD2, TIGD2, TIMM21, TIMM44, TM6SF1, TMC3, TMEM108, TMEM132A, TMEM144, TMEM150B, TMEM150C, TMEM176A, TMEM178B, TMEM190, TMEM232, TMEM238, TMEM240, TMEM38A, TMEM52, TMEM74, TMEM74B, TMPRSS15, TMPRSS2, TMPRSS3, TMTC1, TNFAIP8, TNFRSF25, TNFSF4, TNN, TNNT3, TNXB, TOGARAM2, TOX, TOX3, TP63, TPPP, TPPP3, TPRG1, TRAF3IP3, TRAF5, TRAM1L1, TRAPPC5, TRBV30, TRDC, TREX2, TRIM31, TRIM54, TRIM58, TRIM9, TRIQK, TRPM1, TRPV6, TSHZ3, TSLP, TSPAN12, TSPAN32, TSPAN7, TSPAN8, TSPO2, TSPOAP1, TTBK1, TTC6, TTLL10, TTLL2, TUBAL3, TUBE1, TUSC1, TXNL4A, UBASH3A, UBE2QL1, UCP1, ULBP1, UNC5C, UNC5CL, USH1C, USH2A, USP18, USP2, VASH2, VAV3, VEGFA, VGLL3, VIL1, VOPP1, VSTM2A, VSTM5, VWA1, VWA5A, VWA5B2, VWCE, WDR17, WDR72, WFIKKN1, WIPF1, WNT11, WNT2B, WNT8B, WRAP73, WT1, XBP1, XK, XKR6, XKRX, XYLT1, ZBTB18, ZBTB20, ZC3H12B, ZC3H8, ZCCHC2, ZDHHC1, ZDHHC11, ZDHHC11B, ZEB2, ZFP36L2, ZFPM1, ZFTA, ZNF121, ZNF239, ZNF385B, ZNF397, ZNF474, ZNF692, ZNF711, ZNF713, ZNF750, ZNF773, ZNF775, ZNF8, ZNF831, ZSCAN2, ZSWIM5 | |
|
| 39246444 | Verhagen MP, Xu T, Stabile R, Joosten R, Tucci FA, van Royen M, Trerotola M, Alberti S, Sacchetti A, Fodde R. The SW480 cell line as a model of resident and migrating colon cancer stem cells. iScience. 2024 Aug 5;27 | migratory cancer stem cells (mCSC) | 401 | A4GNT, ABCA1, ABCB4, ABCC8, ABCG2, ACKR3, ACTL6B, ADAM12, ADAMTS10, ADGRA2, ADRA1D, ADRB2, AFF2, AFF3, AMOTL1, ANKRD44, ANPEP, APBA1, APBB1, APLNR, APLP1, APOBEC4, APOD, AR, ARHGEF6, ARL14EPL, ARL15, ASTN2, ATCAY, ATP1A2, ATP8A2, BEX4, BMF, BMP4, BTG4, C10orf62, C10orf90, C14orf132, C1R, C1S, C3orf22, C3orf56, C4orf19, C4orf54, C5AR1, CA13, CABP5, CACNG4, CALD1, CAMK1D, CAMK2N2, CBLN1, CCDC92B, CD28, CD4, CD70, CDH11, CDK14, CELF5, CEMIP2, CERS4, CFAP161, CHAD, CHGB, CHRD, CHRNB2, CLCN1, CLEC11A, CLGN, CMPK2, CMTM3, CNTNAP3, CNTNAP3B, COL1A1, COL26A1, COL2A1, COL5A1, CPA4, CPA5, CPQ, CPXM1, CRACDL, CREB3L1, CRYAB, CSF1, CSF2RB, CSMD2, CXCL14, CYP46A1, DAB2, DACT1, DACT3, DAPK1, DCHS2, DCLK2, DENND2A, DIRAS1, DKKL1, DLGAP3, DNAAF11, DNAI3, DNAJC18, DPF3, DTX4, DUSP15, DUSP19, EDN3, EHF, ENHO, ENPP1, EPHB1, EPPIN, ERC2, ESAM, F10, FAM131B, FAM171A2, FAM181B, FBLN1, FCRL5, FERMT2, FGF1, FHL1, FLT1, FOXO1, FRMD6, FRMPD3, FRZB, FSTL1, GAB1, GABRE, GABRQ, GALNT16, GAS1, GAS7, GATA6, GCNT2, GFRA2, GGT5, GKAP1, GNAL, GPC4, GPNMB, GPR12, GPR162, GRAMD1C, GRAP, GRAP2, GRAPL, GREM1, GRIA1, GSC, GSTM2, GSTM4, GXYLT2, HDDC2, HHIP, HMGCS2, HOXD10, HOXD11, HOXD12, HOXD8, HPCA, HSD11B1, HSPB8, IGDCC4, IGFBP5, IL12RB2, INSYN2A, ISL1, ISLR2, ITGB3, ITIH3, ITK, ITPR1, JCAD, KCNA2, KCNC3, KCND3, KCNH1, KCNK15, KCNK3, KCNQ3, KCNQ5, KCTD7, KIAA0513, KLF7, KLHL24, KRT32, KRT34, KRT35, KRT4, KRTAP1-5, KRTAP4-16, KRTAP4-6, KRTAP4-7, KRTAP4-8, KRTAP4-9, LAMA1, LAYN, LGALS2, LGI4, LGR4, LHX2, LIX1L, LMO4, LMOD1, LONRF2, LRCOL1, LRP1, LRRC32, LRRC55, LTBP1, LYG1, LYPD1, MAEL, MAGEA2, MAGEA2B, MAPT, MARCHF3, MC5R, MEIG1, MFAP5, MIR10B, MMP7, MMP8, MYO7B, N4BP2L1, NALF1, NAV3, NDNF, NEK10, NFASC, NKX6-1, NOG, NOVA2, NPPC, NR5A2, NSG1, NTNG2, NYAP1, OGDHL, OGFRL1, OR10AD1, OR2A1, OR2A42, ORAI3, OSBPL6, OSR2, OTULINL, P2RX1, P3H2, PAPPA2, PAX2, PCDHGB7, PCDHGC4, PDE10A, PDE11A, PDE1C, PDE6A, PDGFRB, PDZRN3, PELI2, PEX5L, PGM5, PHF24, PIANP, PKD1L2, PKDCC, PLA2G4D, PLCB2, PLPPR2, PLPPR4, PNRC1, PODNL1, POGLUT2, PPARGC1A, PPFIBP2, PPM1H, PRDM6, PRICKLE1, PRICKLE2, PRKAR2B, PRKD1, PROX2, PRR9, PRRG3, PTPRG, PYGO1, QPRT, RAB30, RAB3D, RAB7B, RASGEF1B, RASGEF1C, RASGRF1, RET, RFTN1, RGS5, RGS7, RHOJ, RIMS4, RNASE1, RNASE6, ROPN1L, RSAD2, RTP4, SAMD14, SASH1, SCG3, SCRT1, SDR42E1, SELENOM, SEMA5A, SERPINA10, SERPING1, SESN3, SEZ6, SGIP1, SH3PXD2B, SHANK1, SHBG, SHOC1, SLA, SLC1A2, SLC24A3, SLC26A11, SLC29A4, SLC35D3, SLC40A1, SLC46A2, SLC47A1, SLC4A8, SLC5A9, SLC8A3, SLC9A3, SLC9A4, SLFN12, SMO, SMPD3, SNX18, SOD3, SPARC, SPTBN4, SPTLC3, SRPX, SSC4D, ST3GAL3, ST6GAL1, ST6GALNAC3, SUSD2, SV2A, SV2C, SYNPO, SYNPO2L, SYPL2, SYT12, SYT15, SYT15B, SYT17, SYT3, SYT5, TCAF2, TCIM, TG, TMEM132E, TMEM169, TMEM200A, TMEM37, TMIGD3, TNFRSF10C, TNFRSF11A, TNNC1, TNNT2, TPD52L1, TPK1, TREM2, TRIL, TRIM22, TRIM6, TRMT9B, TRPM3, TSHZ2, TTC28, TUBB4A, UNC13A, UPK3A, USP44, VASH1, WDFY4, WSCD1, XIRP1, YPEL1, YPEL2, YPEL4, ZEB1, ZNF385C, ZNF521, ZNF610, ZNF704, ZNF878 | |
| 21419747 | Merlos-Suárez A, Barriga FM, Jung P, Iglesias M, Céspedes MV, Rossell D, Sevillano M, Hernando-Momblona X, da Silva-Diz V, Muñoz P, Clevers H, Sancho E, Mangues R, Batlle E. The intestinal stem cell signature identifies colorectal cancer stem cells and predicts disease relapse. Cell Stem Cell. 2011 May 6 | intestinal stem cell signature | 54 | 4933406C10RIK, 5730471H19RIK, ARL4C, ASCL2, ASRGL1, BCL2, BEX1, BEX4, C13ORF15, CBS, CD44, CENPF, CHST11, CYP11A1, CYP2E1, DCLK1, E330027M22RIK, EPHB3, ESAM, FAM49A, FSTL1, FZD2, HEBP2, IGFBP4, KIF26B, KLHL8, LFNG, LGR5, LIMCH1, MARVELD1, MEX3A, MPP3, NAV1, PCDH8, PHGDH, PRICKLE1, PSRC1, PTHLH, PTPRO, PVT1, PXDN, RASSF5, SCN2B, SLC14A1, SLC1A2, SLCO3A1, SOAT1, SORCS2, ST3GAL3, TACC1, TEAD2, TGIF2, TUBB2B, VAV3 |
| 21419747 | Merlos-Suárez A, Barriga FM, Jung P, Iglesias M, Céspedes MV, Rossell D, Sevillano M, Hernando-Momblona X, da Silva-Diz V, Muñoz P, Clevers H, Sancho E, Mangues R, Batlle E. The intestinal stem cell signature identifies colorectal cancer stem cells and predicts disease relapse. Cell Stem Cell. 2011 May 6 | Lgr5 refined intestinal stem cell signature | 129 | 4933406C10RIK, A730054J21RIK, A930009A15RIK, ABTB2, AFAP1L1, AGR3, AKR1D1, ANGPT2, APCDD1, ARL4C, ASCL2, ASGR1, ASGR2, AXIN2, B230114P17RIK, BCL2, BEX1, BEX4, BHLHB9, BRAF, CAP2, CASP12, CBS, CCDC3, CCDC46, CCND3, CHST11, CHST2, CIB2, CLU, CYP2E1, DGKG, DLGAP1, DYNC2H1, E230029C05RIK, E330027M22RIK, EPHA4, ESRRG, EXPI, FAM115A, FAM188B, FAM20C, FAM43A, FAM49A, FAM64A, FAM65B, FGFR4, FMNL2 , FSCN1, FSTL1, GPER, GPR126, GRAMD1A, GZMA, HNMT, hypothetical 9130022E09, ID2, IGFBP4, IL17RD, IRS1, KIAA1199, KIF12, KIF26B, KLHL13, LDHB, LECT2, LGR5, LIMCH1, LOC100132413, MACC1, MARVELD1, MDFIC, MEX3A, MIPOL1, MNX1, MPP3, MYLK3, NAV1, NOTCH4, NR2E3, NRP1, ODZ4 , P4HTM, PCDH8, PGC, PI3, PIK3IP1, PLP1, PTGDS, PXDN, RASSF10, RASSF4, RASSF5, RGMB, RGS19, RGS7BP, RPE, SCN2B, SDR16C5, SECTM1, SEMA3C, Sept6, SERTAD4, SLC16A2, SLC1A2, SLC25A27, SLC38A4, SLCO3A1, SLIT2, SMARCD3, SMO, SOAT1, SORBS2, SORCS2, SOX4, SRPK2, TACC1, TF, TGTP, TMEM146, TMEM182, TNFRSF19, VAV3, VWA2, WWTR1, ZBTB16, ZFP58, ZNF383, ZNF618 |
| 34691191 | Wang W, Xu C, Ren Y, Wang S, Liao C, Fu X, Hu H. A Novel Cancer Stemness-Related Signature for Predicting Prognosis in Patients with Colon Adenocarcinoma. Stem Cells Int. 2021 Oct 15 | cancer stemness-related prognostic signature for colon adenocarcinoma | 15 | ADAM12, CCDC8, CCDC80, COL5A2, COL6A3, COLEC12, CRISPLD2, DPYSL3, ECM2, ISLR, NRP2, PCDH7, POSTN, RAB31, VGLL3 |
| 36169329 | Firdous S, Ghosh A, Saha S. BCSCdb: a database of biomarkers of cancer stem cells. Database (Oxford). 2022 Sep 28;2022:baac082 | cancer stem cell biomarkers in colon cancer | 48 | ABCB1, ABCC1, ABCG2, AKT1, ALCAM, ALDH1A1, ALDH1A2, ALDH1A3, BMI1, CCND1, CD24, CD24-, CD44, CDH2, CTNNB1, CXCR4, DCLK1, EPCAM, HSPA8, IGFBP3, IL6, IL8, ITGA6, ITGB1, JAG1, KLF4, LGR5, LIN28A, MSI1, MYC, NANOG, NOTCH1, NOTCH2, NOTCH3, POU5F1, PROM1, S100P, SCD, SHH, SNAI1, SNAI2, SOX2, SOX9, TGFB1, TWIST1, VIM, WNT1, YAP1 |

**Table S3.**

| **Supplementary Table 3:** List of genes identified as differentially expressed in SMYD3-KO HCT-116 and EM127-treated WT HCT-116 cells and overlapping with previously published CRC stemness-related gene sets. | | | | | | | | | |
| --- | --- | --- | --- | --- | --- | --- | --- | --- | --- |
| **Reference (Pubmed_ID)** |  |  | 39246444 | 39246444 | 21419747 | 21419747 | 34691191 | | 36169329 |
| **Number of genes in each gene set** |  |  | 1571 | 401 | 54 | 129 | 15 | | 48 |
| **Gene set name** |  |  | resident cancer stem cells (rCSCs) | migratory cancer stem cells (mCSC) | intestinal stem cell signature | Lgr5 refined intestinal stem cell signature | cancer stemness-related prognostic signature for colon adenocarcinoma | | cancer stem cell biomarkers in colon cancer |
| **ENSEMBL_Gene_ID** | **Gene symbol** | **Total number of overlappings with gene sets** | Overlapping with gene set | Overlapping with gene set | Overlapping with gene set | Overlapping with gene set | Overlapping with gene set | Overlapping with gene set | | |
| ENSG00000107796 | ACTA2 | 0 | 0 | 0 | 0 | 0 | 0 | 0 | | |
| ENSG00000197043 | ANXA6 | 0 | 0 | 0 | 0 | 0 | 0 | 0 | | |
| ENSG00000171791 | BCL2 | 3 | 1 | 0 | 1 | 1 | 0 | 0 | | |
| ENSG00000101144 | BMP7 | 0 | 0 | 0 | 0 | 0 | 0 | 0 | | |
| ENSG00000107984 | DKK1 | 0 | 0 | 0 | 0 | 0 | 0 | 0 | | |
| ENSG00000116106 | EPHA4 | 1 | 0 | 0 | 0 | 1 | 0 | 0 | | |
| ENSG00000162344 | FGF19 | 0 | 0 | 0 | 0 | 0 | 0 | 0 | | |
| ENSG00000102678 | FGF9 | 1 | 1 | 0 | 0 | 0 | 0 | 0 | | |
| ENSG00000110195 | FOLR1 | 1 | 1 | 0 | 0 | 0 | 0 | 0 | | |
| ENSG00000054598 | FOXC1 | 0 | 0 | 0 | 0 | 0 | 0 | 0 | | |
| ENSG00000107485 | GATA3 | 0 | 0 | 0 | 0 | 0 | 0 | 0 | | |
| ENSG00000144730 | IL17RD | 1 | 0 | 0 | 0 | 1 | 0 | 0 | | |
| ENSG00000163376 | KBTBD8 | 0 | 0 | 0 | 0 | 0 | 0 | 0 | | |
| ENSG00000168675 | LDLRAD4 | 0 | 0 | 0 | 0 | 0 | 0 | 0 | | |
| ENSG00000134013 | LOXL2 | 0 | 0 | 0 | 0 | 0 | 0 | 0 | | |
| ENSG00000225663 | MCRIP1 | 0 | 0 | 0 | 0 | 0 | 0 | 0 | | |
| ENSG00000081189 | MEF2C | 1 | 1 | 0 | 0 | 0 | 0 | 0 | | |
| ENSG00000136352 | NKX2-1 | 1 | 1 | 0 | 0 | 0 | 0 | 0 | | |
| ENSG00000183691 | NOG | 1 | 0 | 1 | 0 | 0 | 0 | 0 | | |
| ENSG00000157168 | NRG1 | 0 | 0 | 0 | 0 | 0 | 0 | 0 | | |
| ENSG00000099250 | NRP1 | 1 | 0 | 0 | 0 | 1 | 0 | 0 | | |
| ENSG00000118257 | NRP2 | 1 | 0 | 0 | 0 | 0 | 1 | 0 | | |
| ENSG00000130558 | OLFM1 | 0 | 0 | 0 | 0 | 0 | 0 | 0 | | |
| ENSG00000150593 | PDCD4 | 1 | 1 | 0 | 0 | 0 | 0 | 0 | | |
| ENSG00000139174 | PRICKLE1 | 2 | 0 | 1 | 1 | 0 | 0 | 0 | | |
| ENSG00000112183 | RBM24 | 0 | 0 | 0 | 0 | 0 | 0 | 0 | | |
| ENSG00000165731 | RET | 1 | 0 | 1 | 0 | 0 | 0 | 0 | | |
| ENSG00000196154 | S100A4 | 0 | 0 | 0 | 0 | 0 | 0 | 0 | | |
| ENSG00000075213 | SEMA3A | 1 | 1 | 0 | 0 | 0 | 0 | 0 | | |
| ENSG00000012171 | SEMA3B | 0 | 0 | 0 | 0 | 0 | 0 | 0 | | |
| ENSG00000153993 | SEMA3D | 1 | 1 | 0 | 0 | 0 | 0 | 0 | | |
| ENSG00000001617 | SEMA3F | 1 | 1 | 0 | 0 | 0 | 0 | 0 | | |
| ENSG00000095539 | SEMA4G | 1 | 1 | 0 | 0 | 0 | 0 | 0 | | |
| ENSG00000092421 | SEMA6A | 1 | 1 | 0 | 0 | 0 | 0 | 0 | | |
| ENSG00000167680 | SEMA6B | 0 | 0 | 0 | 0 | 0 | 0 | 0 | | |
| ENSG00000143434 | SEMA6C | 0 | 0 | 0 | 0 | 0 | 0 | 0 | | |
| ENSG00000005513 | SOX8 | 0 | 0 | 0 | 0 | 0 | 0 | 0 | | |
| ENSG00000105329 | TGFB1 | 1 | 0 | 0 | 0 | 0 | 0 | 1 | | |
| ENSG00000140682 | TGFB1|1 | 0 | 0 | 0 | 0 | 0 | 0 | 0 | | |
| ENSG00000156299 | TIAM1 | 0 | 0 | 0 | 0 | 0 | 0 | 0 | | |
| ENSG00000122691 | TWIST1 | 1 | 0 | 0 | 0 | 0 | 0 | 1 | | |
| ENSG00000002745 | WNT16 | 0 | 0 | 0 | 0 | 0 | 0 | 0 | |
| When a gene (row) is present in a CRC stemness-related gene set (column) the overlapping score is "1", otherwise it is "0". | | | | | | | | | | |

Table S4.

| **Supplementary Table 4:** Clinical data of CRC patients and molecular characteristics of CRC-SC samples. | | | | | | | | | | | | |
| --- | --- | --- | --- | --- | --- | --- | --- | --- | --- | --- | --- | --- |
| **CRC-SC sample ID** | **Age at diagnosis** | **Gender** | **Tumor type** | **Tumor (anatomic site)** | **TNM  classification** | **AJCC Stage  (8th edition)** | **Metastasis  (anatomic site)** | **Colon polyps  (anatomic site)** | **Somatic mutation** | **MMR system** | **SMYD3** | **CMS classification** |
| OG14 | 84 | Female | Colon adenocarcinoma | Colon (right) | pT2N0M0 | Stage I |  | No | No | MSS | High | CMS1 |
| OG17 | 70 | Male | Rectal adenocarcinoma | Rectum | pT4aN2bM1 | Stage IV | Yes (liver, lung) | No | KRAS (p.G12V) | MSS | High | CMS1 |
| OF90 | 78 | Female | Colorectal adenocarcinoma | Colon (sigma)/Rectum | pT3N0M1a | Stage IVA | Yes (liver) | No | KRAS (p.G12D) | MSS | High | CMS4 |
| OF96 | 90 | Male | Rectal adenocarcinoma | Rectum | pT3N0M0 | Stage IIA |  | No | No | MSS | High | CMS4 |
| OF99 | 57 | Male | Colon adenocarcinoma | Colon (transverse) | pT3N1aM0 | Stage IIIB |  | No | KRAS (p.G12V) | MSS | High | CMS4 |
| OG02 | 86 | Male | Rectal adenocarcinoma | Rectum | pT2N0M0 | Stage I |  | No | No | MSS | High | CMS4 |
| OG20 | 72 | Male | Colon adenocarcinoma | Colon (sigma) | pT2N0M0 | Stage I |  | No | No | MSS | High | CMS4 |
| OF81 | 72 | Male | Colon adenocarcinoma | Colon (sigma) | pT2N0M1a | Stage IVA | Yes (liver) | No | No | MSS | Low | CMS3 |
| OF84 | 57 | Male | Colon adenocarcinoma | Colon (transverse) | pT3N0M0 | Stage IIA |  | No | KRAS (p.G12R) | MSS | Low | CMS1 |
| OF87 | 77 | Female | Colon adenocarcinoma | Colon (right) | pT4aN0M0 | Stage IIB |  | No | BRAF (p.V600E/D) | MSI-H | Low | CMS1 |
| OG06 | 62 | Male | Rectal adenocarcinoma | Rectum | pT3N2bM0 | Stage IIIC |  | No | KRAS (p.G12D) | MSS | Low | CMS1 |
| OG08 | 83 | Female | Colon adenocarcinoma | Colon (transverse) | pT4aN0M0 | Stage IIB |  | No | No | MSI-H | Low | CMS3 |
| OG11 | 78 | Female | Colon adenocarcinoma | Colon (sigma) | pT3N1bM0 | Stage IIIB |  | No | BRAF (p.V600E) | MSS | Low | CMS2 |
| OF93 | 65 | Male | Colon adenocarcinoma | Colon (right) | pT4aN0M0 | Stage IIB |  | Yes (Sigma) | No | MSS | Low | CMS4 |

Abbreviations: AJCC Stage, American Joint Committee on Cancer Stage (8th edition); CMS, consensus molecular subtype (CMS1-4); CRC, colorectal cancer; CRC-SCs, colorectal cancer stem cells; MMR, mismatch repair; MSI, microsatellite instability; MSS, microsatellite stability; TNM, tumor-node-metastasis.

Table S5.

| **Supplementary Table 5:** List of the 57 c-MYC target genes with negative GSEA scores in EM127-treated CRC-SCs derived from patients with SMYD3-overexpressing cancers, when compared to untreated cells. The 10 genes that are relevant to stemness-related processes based on the experimental annotations from the ChEA database are indicated in bold (https://maayanlab.cloud/Harmonizome/dataset/CHEA+Transcription+Factor+Targets). | | | | | | | | | | |
| --- | --- | --- | --- | --- | --- | --- | --- | --- | --- | --- |
| **Ensembl unique ID+A2:L52** | **Gene symbol** | **log2(FC) (em127/NT)** | **Uniprot entry** | **Entry name** | **Protein names** | **Gene names** | **Length** | **Function [CC]** | **Gene ontology (biological processes)** | **Gene ontology IDs** |
| ENSG00000104738 | MCM4 | -0.01449957 | P33991 | MCM4_HUMAN | DNA replication licensing factor MCM4 (EC 3.6.4.12) (CDC21 homolog) (P1-CDC21) | MCM4 CDC21 | 863 | FUNCTION: Acts as component of the MCM2-7 complex (MCM complex) which is the replicative helicase essential for 'once per cell cycle' DNA replication initiation and elongation in eukaryotic cells. Core component of CDC45-MCM-GINS (CMG) helicase, the molecular machine that unwinds template DNA during replication, and around which the replisome is built (PubMed:32453425, PubMed:34694004, PubMed:34700328, PubMed:35585232, PubMed:16899510, PubMed:25661590, PubMed:9305914). The active ATPase sites in the MCM2-7 ring are formed through the interaction surfaces of two neighboring subunits such that a critical structure of a conserved arginine finger motif is provided in trans relative to the ATP-binding site of the Walker A box of the adjacent subunit. The six ATPase active sites, however, are likely to contribute differentially to the complex helicase activity (PubMed:32453425, PubMed:16899510, PubMed:25661590, PubMed:9305914). {ECO:0000269|PubMed:16899510, ECO:0000269|PubMed:25661590, ECO:0000269|PubMed:32453425, ECO:0000269|PubMed:34694004, ECO:0000269|PubMed:34700328, ECO:0000269|PubMed:35585232, ECO:0000269|PubMed:9305914}. | DNA replication [GO:0006260]; DNA strand elongation involved in DNA replication [GO:0006271]; DNA unwinding involved in DNA replication [GO:0006268]; double-strand break repair via break-induced replication [GO:0000727]; mitotic DNA replication initiation [GO:1902975]; regulation of DNA-templated DNA replication initiation [GO:0030174] | GO:0000727; GO:0000781; GO:0003678; GO:0003697; GO:0005524; GO:0005634; GO:0005654; GO:0006260; GO:0006268; GO:0006271; GO:0016020; GO:0016887; GO:0030174; GO:0042555; GO:0071162; GO:1902975 |
| ENSG00000135624 | CCT7 | -0.01449957 | Q99832 | TCPH_HUMAN | T-complex protein 1 subunit eta (TCP-1-eta) (CCT-eta) (HIV-1 Nef-interacting protein) [Cleaved into: T-complex protein 1 subunit eta, N-terminally processed] | CCT7 CCTH NIP7-1 | 543 | FUNCTION: Component of the chaperonin-containing T-complex (TRiC), a molecular chaperone complex that assists the folding of proteins upon ATP hydrolysis (PubMed:25467444). The TRiC complex mediates the folding of WRAP53/TCAB1, thereby regulating telomere maintenance (PubMed:25467444). The TRiC complex plays a role in the folding of actin and tubulin (Probable). {ECO:0000269|PubMed:25467444, ECO:0000305}. | binding of sperm to zona pellucida [GO:0007339]; chaperone-mediated protein folding [GO:0061077]; positive regulation of establishment of protein localization to telomere [GO:1904851]; positive regulation of protein localization to Cajal body [GO:1904871]; positive regulation of telomerase RNA localization to Cajal body [GO:1904874]; positive regulation of telomere maintenance via telomerase [GO:0032212]; protein folding [GO:0006457]; protein stabilization [GO:0050821] | GO:0005524; GO:0005737; GO:0005829; GO:0005832; GO:0005874; GO:0006457; GO:0007339; GO:0016887; GO:0032212; GO:0044183; GO:0044297; GO:0050821; GO:0051082; GO:0061077; GO:0070062; GO:0140662; GO:1904851; GO:1904871; GO:1904874 |
| ENSG00000160201 | U2AF1 | -0.01449957 | Q01081 | U2AF1_HUMAN | Splicing factor U2AF 35 kDa subunit (U2 auxiliary factor 35 kDa subunit) (U2 small nuclear RNA auxiliary factor 1) (U2 snRNP auxiliary factor small subunit) | U2AF1 U2AF35 U2AFBP FP793 | 240 | FUNCTION: Plays a critical role in both constitutive and enhancer-dependent splicing by mediating protein-protein interactions and protein-RNA interactions required for accurate 3'-splice site selection. Recruits U2 snRNP to the branch point. Directly mediates interactions between U2AF2 and proteins bound to the enhancers and thus may function as a bridge between U2AF2 and the enhancer complex to recruit it to the adjacent intron. {ECO:0000269|PubMed:22158538, ECO:0000269|PubMed:25311244, ECO:0000269|PubMed:8647433}. | mRNA processing [GO:0006397]; mRNA splicing, via spliceosome [GO:0000398]; RNA splicing [GO:0008380] | GO:0000398; GO:0003723; GO:0005654; GO:0005681; GO:0006397; GO:0008380; GO:0015030; GO:0016607; GO:0030628; GO:0046872; GO:0050733; GO:0071013; GO:0089701 |
| **ENSG00000277791** | **PSMB3** | **-0.01449957** | **P49720** | **PSB3_HUMAN** | **Proteasome subunit beta type-3 (Proteasome chain 13) (Proteasome component C10-II) (Proteasome theta chain)** | **PSMB3** | **205** | **FUNCTION: Non-catalytic component of the 20S core proteasome complex involved in the proteolytic degradation of most intracellular proteins. This complex plays numerous essential roles within the cell by associating with different regulatory particles. Associated with two 19S regulatory particles, forms the 26S proteasome and thus participates in the ATP-dependent degradation of ubiquitinated proteins. The 26S proteasome plays a key role in the maintenance of protein homeostasis by removing misfolded or damaged proteins that could impair cellular functions, and by removing proteins whose functions are no longer required. Associated with the PA200 or PA28, the 20S proteasome mediates ubiquitin-independent protein degradation. This type of proteolysis is required in several pathways including spermatogenesis (20S-PA200 complex) or generation of a subset of MHC class I-presented antigenic peptides (20S-PA28 complex). {ECO:0000269|PubMed:15244466, ECO:0000269|PubMed:27176742, ECO:0000269|PubMed:8610016}.** | **proteasomal protein catabolic process [GO:0010498]; proteasome-mediated ubiquitin-dependent protein catabolic process [GO:0043161]** | **GO:0000502; GO:0005634; GO:0005654; GO:0005737; GO:0005829; GO:0005839; GO:0010498; GO:0019774; GO:0043161; GO:0070062** |
| **ENSG00000070756** | **PABPC1** | **-0.029146346** | **P11940** | **PABP1_HUMAN** | **Polyadenylate-binding protein 1 (PABP-1) (Poly(A)-binding protein 1)** | **PABPC1 PAB1 PABP PABP1 PABPC2** | **636** | **FUNCTION: Binds the poly(A) tail of mRNA, including that of its own transcript, and regulates processes of mRNA metabolism such as pre-mRNA splicing and mRNA stability (PubMed:11051545, PubMed:17212783, PubMed:25480299). Its function in translational initiation regulation can either be enhanced by PAIP1 or repressed by PAIP2 (PubMed:11051545, PubMed:20573744). Can probably bind to cytoplasmic RNA sequences other than poly(A) in vivo. Binds to N6-methyladenosine (m6A)-containing mRNAs and contributes to MYC stability by binding to m6A-containing MYC mRNAs (PubMed:32245947). Involved in translationally coupled mRNA turnover (PubMed:11051545). Implicated with other RNA-binding proteins in the cytoplasmic deadenylation/translational and decay interplay of the FOS mRNA mediated by the major coding-region determinant of instability (mCRD) domain (PubMed:11051545). Involved in regulation of nonsense-mediated decay (NMD) of mRNAs containing premature stop codons; for the recognition of premature termination codons (PTC) and initiation of NMD a competitive interaction between UPF1 and PABPC1 with the ribosome-bound release factors is proposed (PubMed:18447585). By binding to long poly(A) tails, may protect them from uridylation by ZCCHC6/ZCCHC11 and hence contribute to mRNA stability (PubMed:25480299). {ECO:0000269|PubMed:11051545, ECO:0000269|PubMed:17212783, ECO:0000269|PubMed:18447585, ECO:0000269|PubMed:20573744, ECO:0000269|PubMed:25480299, ECO:0000269|PubMed:32245947}.; FUNCTION: (Microbial infection) Positively regulates the replication of dengue virus (DENV). {ECO:0000269|PubMed:26735137}.** | **CRD-mediated mRNA stabilization [GO:0070934]; mRNA polyadenylation [GO:0006378]; mRNA splicing, via spliceosome [GO:0000398]; mRNA stabilization [GO:0048255]; negative regulation of nuclear-transcribed mRNA catabolic process, deadenylation-dependent decay [GO:1900152]; negative regulation of nuclear-transcribed mRNA catabolic process, nonsense-mediated decay [GO:2000623]; nuclear-transcribed mRNA catabolic process, nonsense-mediated decay [GO:0000184]; positive regulation of cytoplasmic translation [GO:2000767]; positive regulation of nuclear-transcribed mRNA catabolic process, deadenylation-dependent decay [GO:1900153]; positive regulation of nuclear-transcribed mRNA poly(A) tail shortening [GO:0060213]; positive regulation of viral genome replication [GO:0045070]; regulatory ncRNA-mediated gene silencing [GO:0031047]** | **GO:0000184; GO:0000398; GO:0003723; GO:0003729; GO:0003730; GO:0005634; GO:0005737; GO:0005829; GO:0005925; GO:0006378; GO:0008143; GO:0008266; GO:0008494; GO:0010494; GO:0016020; GO:0030027; GO:0031047; GO:0031252; GO:0036464; GO:0045070; GO:0048255; GO:0060213; GO:0070062; GO:0070934; GO:0071013; GO:0106002; GO:1900152; GO:1900153; GO:1990904; GO:2000623; GO:2000767** |
| ENSG00000122566 | HNRNPA2B1 | -0.029146346 | P22626 | ROA2_HUMAN | Heterogeneous nuclear ribonucleoproteins A2/B1 (hnRNP A2/B1) | HNRNPA2B1 HNRPA2B1 | 353 | FUNCTION: Heterogeneous nuclear ribonucleoprotein (hnRNP) that associates with nascent pre-mRNAs, packaging them into hnRNP particles. The hnRNP particle arrangement on nascent hnRNA is non-random and sequence-dependent and serves to condense and stabilize the transcripts and minimize tangling and knotting. Packaging plays a role in various processes such as transcription, pre-mRNA processing, RNA nuclear export, subcellular location, mRNA translation and stability of mature mRNAs (PubMed:19099192). Forms hnRNP particles with at least 20 other different hnRNP and heterogeneous nuclear RNA in the nucleus. Involved in transport of specific mRNAs to the cytoplasm in oligodendrocytes and neurons: acts by specifically recognizing and binding the A2RE (21 nucleotide hnRNP A2 response element) or the A2RE11 (derivative 11 nucleotide oligonucleotide) sequence motifs present on some mRNAs, and promotes their transport to the cytoplasm (PubMed:10567417). Specifically binds single-stranded telomeric DNA sequences, protecting telomeric DNA repeat against endonuclease digestion (By similarity). Also binds other RNA molecules, such as primary miRNA (pri-miRNAs): acts as a nuclear 'reader' of the N6-methyladenosine (m6A) mark by specifically recognizing and binding a subset of nuclear m6A-containing pri-miRNAs. Binding to m6A-containing pri-miRNAs promotes pri-miRNA processing by enhancing binding of DGCR8 to pri-miRNA transcripts (PubMed:26321680). Involved in miRNA sorting into exosomes following sumoylation, possibly by binding (m6A)-containing pre-miRNAs (PubMed:24356509). Acts as a regulator of efficiency of mRNA splicing, possibly by binding to m6A-containing pre-mRNAs (PubMed:26321680). Plays a role in the splicing of pyruvate kinase PKM by binding repressively to sequences flanking PKM exon 9, inhibiting exon 9 inclusion and resulting in exon 10 inclusion and production of the PKM M2 isoform (PubMed:20010808). Also plays a role in the activation of the innate immune response (PubMed:31320558). Mechanistically, senses the presence of viral DNA in the nucleus, homodimerizes and is demethylated by JMJD6 (PubMed:31320558). In turn, translocates to the cytoplasm where it activates the TBK1-IRF3 pathway, leading to interferon alpha/beta production (PubMed:31320558). {ECO:0000250|UniProtKB:A7VJC2, ECO:0000269|PubMed:10567417, ECO:0000269|PubMed:20010808, ECO:0000269|PubMed:24356509, ECO:0000269|PubMed:26321680, ECO:0000303|PubMed:19099192}.; FUNCTION: (Microbial infection) Involved in the transport of HIV-1 genomic RNA out of the nucleus, to the microtubule organizing center (MTOC), and then from the MTOC to the cytoplasm: acts by specifically recognizing and binding the A2RE (21 nucleotide hnRNP A2 response element) sequence motifs present on HIV-1 genomic RNA, and promotes its transport. {ECO:0000269|PubMed:15294897, ECO:0000269|PubMed:17004321}. | G-quadruplex DNA unwinding [GO:0044806]; miRNA transport [GO:1990428]; mRNA export from nucleus [GO:0006406]; mRNA processing [GO:0006397]; mRNA splicing, via spliceosome [GO:0000398]; mRNA transport [GO:0051028]; negative regulation of mRNA splicing, via spliceosome [GO:0048025]; negative regulation of transcription by RNA polymerase II [GO:0000122]; positive regulation of telomerase RNA reverse transcriptase activity [GO:1905663]; positive regulation of telomere maintenance via telomere lengthening [GO:1904358]; primary miRNA processing [GO:0031053]; RNA transport [GO:0050658] | GO:0000122; GO:0000398; GO:0000781; GO:0003723; GO:0003730; GO:0005634; GO:0005654; GO:0005681; GO:0005737; GO:0006397; GO:0006406; GO:0015030; GO:0016020; GO:0016363; GO:0031053; GO:0035198; GO:0042802; GO:0043047; GO:0044806; GO:0048025; GO:0050658; GO:0051028; GO:0070062; GO:0071013; GO:0097157; GO:0098505; GO:0140693; GO:1904358; GO:1905663; GO:1990247; GO:1990428; GO:1990904 |
| ENSG00000166226 | CCT2 | -0.029146346 | P78371 | TCPB_HUMAN | T-complex protein 1 subunit beta (TCP-1-beta) (CCT-beta) | CCT2 99D8.1 CCTB | 535 | FUNCTION: Component of the chaperonin-containing T-complex (TRiC), a molecular chaperone complex that assists the folding of proteins upon ATP hydrolysis (PubMed:25467444). The TRiC complex mediates the folding of WRAP53/TCAB1, thereby regulating telomere maintenance (PubMed:25467444). As part of the TRiC complex may play a role in the assembly of BBSome, a complex involved in ciliogenesis regulating transports vesicles to the cilia (PubMed:20080638). The TRiC complex plays a role in the folding of actin and tubulin (Probable). {ECO:0000269|PubMed:20080638, ECO:0000269|PubMed:25467444, ECO:0000305}. | binding of sperm to zona pellucida [GO:0007339]; chaperone mediated protein folding independent of cofactor [GO:0051086]; chaperone-mediated protein complex assembly [GO:0051131]; chaperone-mediated protein folding [GO:0061077]; positive regulation of establishment of protein localization to telomere [GO:1904851]; positive regulation of protein localization to Cajal body [GO:1904871]; positive regulation of telomerase activity [GO:0051973]; positive regulation of telomerase RNA localization to Cajal body [GO:1904874]; positive regulation of telomere maintenance via telomerase [GO:0032212]; protein folding [GO:0006457]; protein stabilization [GO:0050821]; scaRNA localization to Cajal body [GO:0090666] | GO:0002199; GO:0005524; GO:0005576; GO:0005829; GO:0005832; GO:0005874; GO:0006457; GO:0007339; GO:0016887; GO:0031625; GO:0032212; GO:0035578; GO:0044183; GO:0044297; GO:0050821; GO:0051082; GO:0051086; GO:0051131; GO:0051973; GO:0061077; GO:0070062; GO:0090666; GO:0140662; GO:1904851; GO:1904871; GO:1904874 |
| ENSG00000176890 | TYMS | -0.029146346 | P04818 | TYSY_HUMAN | Thymidylate synthase (TS) (TSase) (EC 2.1.1.45) | TYMS TS OK/SW-cl.29 | 313 | FUNCTION: Catalyzes the reductive methylation of 2'-deoxyuridine 5'-monophosphate (dUMP) to thymidine 5'-monophosphate (dTMP), using the cosubstrate, 5,10- methylenetetrahydrofolate (CH2H4folate) as a 1-carbon donor and reductant and contributes to the de novo mitochondrial thymidylate biosynthesis pathway. {ECO:0000269|PubMed:11278511, ECO:0000269|PubMed:21876188}. | cartilage development [GO:0051216]; circadian rhythm [GO:0007623]; developmental growth [GO:0048589]; DNA biosynthetic process [GO:0071897]; dTMP biosynthetic process [GO:0006231]; dTTP biosynthetic process [GO:0006235]; intestinal epithelial cell maturation [GO:0060574]; liver regeneration [GO:0097421]; methylation [GO:0032259]; negative regulation of translation [GO:0017148]; response to cytokine [GO:0034097]; response to ethanol [GO:0045471]; response to folic acid [GO:0051593]; response to glucocorticoid [GO:0051384]; response to organophosphorus [GO:0046683]; response to progesterone [GO:0032570]; response to toxic substance [GO:0009636]; response to vitamin A [GO:0033189]; response to xenobiotic stimulus [GO:0009410]; tetrahydrofolate interconversion [GO:0035999]; uracil metabolic process [GO:0019860] | GO:0000900; GO:0004799; GO:0005542; GO:0005634; GO:0005737; GO:0005739; GO:0005743; GO:0005759; GO:0005829; GO:0006231; GO:0006235; GO:0007623; GO:0009410; GO:0009636; GO:0017148; GO:0019860; GO:0032259; GO:0032570; GO:0033189; GO:0034097; GO:0035999; GO:0042803; GO:0045471; GO:0046683; GO:0048589; GO:0051216; GO:0051384; GO:0051593; GO:0060574; GO:0071897; GO:0097421; GO:1990825 |
| ENSG00000239672 | NME1 | -0.029146346 | P15531 | NDKA_HUMAN | Nucleoside diphosphate kinase A (NDK A) (NDP kinase A) (EC 2.7.4.6) (Granzyme A-activated DNase) (GAAD) (Metastasis inhibition factor nm23) (NM23-H1) (Tumor metastatic process-associated protein) | NME1 NDPKA NM23 | 152 | FUNCTION: Major role in the synthesis of nucleoside triphosphates other than ATP. The ATP gamma phosphate is transferred to the NDP beta phosphate via a ping-pong mechanism, using a phosphorylated active-site intermediate. Possesses nucleoside-diphosphate kinase, serine/threonine-specific protein kinase, geranyl and farnesyl pyrophosphate kinase, histidine protein kinase and 3'-5' exonuclease activities. Involved in cell proliferation, differentiation and development, signal transduction, G protein-coupled receptor endocytosis, and gene expression. Required for neural development including neural patterning and cell fate determination. During GZMA-mediated cell death, works in concert with TREX1. NME1 nicks one strand of DNA and TREX1 removes bases from the free 3' end to enhance DNA damage and prevent DNA end reannealing and rapid repair. {ECO:0000269|PubMed:12628186, ECO:0000269|PubMed:16818237, ECO:0000269|PubMed:8810265}. | cell differentiation [GO:0030154]; CTP biosynthetic process [GO:0006241]; endocytosis [GO:0006897]; GTP biosynthetic process [GO:0006183]; lactation [GO:0007595]; negative regulation of cell population proliferation [GO:0008285]; nervous system development [GO:0007399]; phosphorylation [GO:0016310]; positive regulation of DNA binding [GO:0043388]; positive regulation of epithelial cell proliferation [GO:0050679]; regulation of apoptotic process [GO:0042981]; UTP biosynthetic process [GO:0006228] | GO:0000287; GO:0003723; GO:0004536; GO:0004550; GO:0005524; GO:0005525; GO:0005634; GO:0005737; GO:0005829; GO:0006183; GO:0006228; GO:0006241; GO:0006897; GO:0007399; GO:0007595; GO:0008285; GO:0016020; GO:0016310; GO:0030154; GO:0032587; GO:0042802; GO:0042981; GO:0043024; GO:0043388; GO:0050679; GO:0070062 |
| ENSG00000048828 | FAM120A | -0.043943348 | Q9NZB2 | F120A_HUMAN | Constitutive coactivator of PPAR-gamma-like protein 1 (Oxidative stress-associated SRC activator) (Protein FAM120A) | FAM120A C9orf10 KIAA0183 OSSA | 1118 | FUNCTION: Component of the oxidative stress-induced survival signaling. May regulate the activation of SRC family protein kinases (PubMed:19015244). May act as a scaffolding protein enabling SRC family protein kinases to phosphorylate and activate PI3-kinase (PubMed:19015244). Binds IGF2 RNA and promotes the production of IGF2 protein (PubMed:19015244). {ECO:0000269|PubMed:19015244}. |  | GO:0003723; GO:0005634; GO:0005829; GO:0005886; GO:0016020 |
| **ENSG00000124795** | **DEK** | **-0.043943348** | **P35659** | **DEK_HUMAN** | **Protein DEK** | **DEK** | **375** | **FUNCTION: Involved in chromatin organization. {ECO:0000269|PubMed:17524367}.** | **chromatin remodeling [GO:0006338]; positive regulation of histone acetylation [GO:0035066]; positive regulation of transcription by RNA polymerase I [GO:0045943]; positive regulation of transcription by RNA polymerase II [GO:0045944]; positive regulation of transcription by RNA polymerase III [GO:0045945]; regulation of double-strand break repair [GO:2000779]; regulation of double-strand break repair via nonhomologous end joining [GO:2001032]; regulation of transcription by RNA polymerase II [GO:0006357]; signal transduction [GO:0007165]; transcription by RNA polymerase II [GO:0006366]; viral genome replication [GO:0019079]** | **GO:0003677; GO:0003723; GO:0005634; GO:0005654; GO:0005730; GO:0006338; GO:0006357; GO:0006366; GO:0007165; GO:0019079; GO:0035066; GO:0042393; GO:0043292; GO:0045943; GO:0045944; GO:0045945; GO:0110016; GO:2000779; GO:2001032** |
| **ENSG00000136937** | **NCBP1** | **-0.043943348** | **Q09161** | **NCBP1_HUMAN** | **Nuclear cap-binding protein subunit 1 (80 kDa nuclear cap-binding protein) (CBP80) (NCBP 80 kDa subunit)** | **NCBP1 CBP80 NCBP** | **790** | **FUNCTION: Component of the cap-binding complex (CBC), which binds cotranscriptionally to the 5'-cap of pre-mRNAs and is involved in various processes such as pre-mRNA splicing, translation regulation, nonsense-mediated mRNA decay, RNA-mediated gene silencing (RNAi) by microRNAs (miRNAs) and mRNA export. The CBC complex is involved in mRNA export from the nucleus via its interaction with ALYREF/THOC4/ALY, leading to the recruitment of the mRNA export machinery to the 5'-end of mRNA and to mRNA export in a 5' to 3' direction through the nuclear pore. The CBC complex is also involved in mediating U snRNA and intronless mRNAs export from the nucleus. The CBC complex is essential for a pioneer round of mRNA translation, before steady state translation when the CBC complex is replaced by cytoplasmic cap-binding protein eIF4E. The pioneer round of mRNA translation mediated by the CBC complex plays a central role in nonsense-mediated mRNA decay (NMD), NMD only taking place in mRNAs bound to the CBC complex, but not on eIF4E-bound mRNAs. The CBC complex enhances NMD in mRNAs containing at least one exon-junction complex (EJC) via its interaction with UPF1, promoting the interaction between UPF1 and UPF2. The CBC complex is also involved in 'failsafe' NMD, which is independent of the EJC complex, while it does not participate in Staufen-mediated mRNA decay (SMD). During cell proliferation, the CBC complex is also involved in microRNAs (miRNAs) biogenesis via its interaction with SRRT/ARS2 and is required for miRNA-mediated RNA interference. The CBC complex also acts as a negative regulator of PARN, thereby acting as an inhibitor of mRNA deadenylation. In the CBC complex, NCBP1/CBP80 does not bind directly capped RNAs (m7GpppG-capped RNA) but is required to stabilize the movement of the N-terminal loop of NCBP2/CBP20 and lock the CBC into a high affinity cap-binding state with the cap structure. Associates with NCBP3 to form an alternative cap-binding complex (CBC) which plays a key role in mRNA export and is particularly important in cellular stress situations such as virus infections. The conventional CBC with NCBP2 binds both small nuclear RNA (snRNA) and messenger (mRNA) and is involved in their export from the nucleus whereas the alternative CBC with NCBP3 does not bind snRNA and associates only with mRNA thereby playing a role only in mRNA export. NCBP1/CBP80 is required for cell growth and viability (PubMed:26382858). {ECO:0000269|PubMed:11551508, ECO:0000269|PubMed:12093754, ECO:0000269|PubMed:15059963, ECO:0000269|PubMed:15361857, ECO:0000269|PubMed:16186820, ECO:0000269|PubMed:16317009, ECO:0000269|PubMed:17190602, ECO:0000269|PubMed:17873884, ECO:0000269|PubMed:18369367, ECO:0000269|PubMed:19632182, ECO:0000269|PubMed:19648179, ECO:0000269|PubMed:26382858, ECO:0000269|PubMed:7651522, ECO:0000269|PubMed:8069914}.** | **7-methylguanosine mRNA capping [GO:0006370]; alternative mRNA splicing, via spliceosome [GO:0000380]; cap-dependent translational initiation [GO:0002191]; defense response to virus [GO:0051607]; histone mRNA metabolic process [GO:0008334]; miRNA-mediated post-transcriptional gene silencing [GO:0035195]; mRNA 3'-end processing [GO:0031124]; mRNA export from nucleus [GO:0006406]; mRNA metabolic process [GO:0016071]; mRNA splicing, via spliceosome [GO:0000398]; mRNA transcription by RNA polymerase II [GO:0042789]; nuclear-transcribed mRNA catabolic process, nonsense-mediated decay [GO:0000184]; positive regulation of cell growth [GO:0030307]; positive regulation of mRNA 3'-end processing [GO:0031442]; positive regulation of mRNA splicing, via spliceosome [GO:0048026]; positive regulation of RNA binding [GO:1905216]; positive regulation of transcription elongation by RNA polymerase II [GO:0032968]; primary miRNA processing [GO:0031053]; regulation of mRNA processing [GO:0050684]; regulation of translational initiation [GO:0006446]; regulatory ncRNA-mediated post-transcriptional gene silencing [GO:0035194]; RNA catabolic process [GO:0006401]; RNA splicing [GO:0008380]; snRNA export from nucleus [GO:0006408]; spliceosomal complex assembly [GO:0000245]** | **GO:0000184; GO:0000245; GO:0000339; GO:0000340; GO:0000380; GO:0000398; GO:0002191; GO:0003723; GO:0003729; GO:0005634; GO:0005654; GO:0005737; GO:0005739; GO:0005829; GO:0005845; GO:0005846; GO:0006370; GO:0006401; GO:0006406; GO:0006408; GO:0006446; GO:0008334; GO:0008380; GO:0016071; GO:0030307; GO:0031053; GO:0031124; GO:0031442; GO:0032968; GO:0034518; GO:0035194; GO:0035195; GO:0042789; GO:0048026; GO:0050684; GO:0051607; GO:0060090; GO:1905216; GO:1990904** |
| ENSG00000162607 | USP1 | -0.043943348 | O94782 | UBP1_HUMAN | Ubiquitin carboxyl-terminal hydrolase 1 (EC 3.4.19.12) (Deubiquitinating enzyme 1) (hUBP) (Ubiquitin thioesterase 1) (Ubiquitin-specific-processing protease 1) [Cleaved into: Ubiquitin carboxyl-terminal hydrolase 1, N-terminal fragment] | USP1 | 785 | FUNCTION: Negative regulator of DNA damage repair which specifically deubiquitinates monoubiquitinated FANCD2 (PubMed:15694335). Also involved in PCNA-mediated translesion synthesis (TLS) by deubiquitinating monoubiquitinated PCNA (PubMed:16531995, PubMed:20147293). Has almost no deubiquitinating activity by itself and requires the interaction with WDR48 to have a high activity (PubMed:18082604, PubMed:26388029). {ECO:0000269|PubMed:15694335, ECO:0000269|PubMed:16531995, ECO:0000269|PubMed:18082604, ECO:0000269|PubMed:20147293, ECO:0000269|PubMed:26388029}. | DNA repair [GO:0006281]; monoubiquitinated protein deubiquitination [GO:0035520]; positive regulation of error-prone translesion synthesis [GO:1904333]; positive regulation of receptor signaling pathway via JAK-STAT [GO:0046427]; protein deubiquitination [GO:0016579]; regulation of DNA repair [GO:0006282]; response to UV [GO:0009411]; skeletal system development [GO:0001501] | GO:0001501; GO:0004197; GO:0004843; GO:0005634; GO:0005654; GO:0005829; GO:0006281; GO:0006282; GO:0008233; GO:0009411; GO:0016579; GO:0035520; GO:0046427; GO:1904333 |
| ENSG00000136450 | SRSF1 | -0.058893689 | Q07955 | SRSF1_HUMAN | Serine/arginine-rich splicing factor 1 (Alternative-splicing factor 1) (ASF-1) (Splicing factor, arginine/serine-rich 1) (pre-mRNA-splicing factor SF2, P33 subunit) | SRSF1 ASF SF2 SF2P33 SFRS1 OK/SW-cl.3 | 248 | FUNCTION: Plays a role in preventing exon skipping, ensuring the accuracy of splicing and regulating alternative splicing. Interacts with other spliceosomal components, via the RS domains, to form a bridge between the 5'- and 3'-splice site binding components, U1 snRNP and U2AF. Can stimulate binding of U1 snRNP to a 5'-splice site-containing pre-mRNA. Binds to purine-rich RNA sequences, either the octamer, 5'-RGAAGAAC-3' (r=A or G) or the decamers, AGGACAGAGC/AGGACGAAGC. Binds preferentially to the 5'-CGAGGCG-3' motif in vitro. Three copies of the octamer constitute a powerful splicing enhancer in vitro, the ASF/SF2 splicing enhancer (ASE) which can specifically activate ASE-dependent splicing. Isoform ASF-2 and isoform ASF-3 act as splicing repressors. May function as export adapter involved in mRNA nuclear export through the TAP/NXF1 pathway. {ECO:0000269|PubMed:8139654}. | alternative mRNA splicing, via spliceosome [GO:0000380]; liver regeneration [GO:0097421]; mRNA 5'-splice site recognition [GO:0000395]; mRNA processing [GO:0006397]; mRNA splice site recognition [GO:0006376]; mRNA splicing, via spliceosome [GO:0000398]; mRNA transport [GO:0051028]; oligodendrocyte differentiation [GO:0048709]; positive regulation of RNA splicing [GO:0033120]; regulation of RNA splicing [GO:0043484] | GO:0000380; GO:0000395; GO:0000398; GO:0003723; GO:0003729; GO:0005634; GO:0005654; GO:0005737; GO:0006376; GO:0006397; GO:0016607; GO:0033120; GO:0043422; GO:0043484; GO:0044547; GO:0048709; GO:0051028; GO:0071013; GO:0097421 |
| ENSG00000143727 | ACP1 | -0.058893689 | P24666 | PPAC_HUMAN | Low molecular weight phosphotyrosine protein phosphatase (LMW-PTP) (LMW-PTPase) (EC 3.1.3.48) (Adipocyte acid phosphatase) (Low molecular weight cytosolic acid phosphatase) (EC 3.1.3.2) (Red cell acid phosphatase 1) | ACP1 | 158 | FUNCTION: Acts on tyrosine phosphorylated proteins, low-MW aryl phosphates and natural and synthetic acyl phosphates with differences in substrate specificity between isoform 1 and isoform 2. {ECO:0000269|PubMed:10336608, ECO:0000269|PubMed:9705307}.; FUNCTION: [Isoform 3]: Does not possess phosphatase activity. {ECO:0000269|PubMed:10336608}. |  | GO:0003993; GO:0004725; GO:0004726; GO:0005737; GO:0005829; GO:0009898; GO:0042383; GO:0070062 |
| ENSG00000120948 | TARDBP | -0.074000581 | Q13148 | TADBP_HUMAN | TAR DNA-binding protein 43 (TDP-43) | TARDBP TDP43 | 414 | FUNCTION: RNA-binding protein that is involved in various steps of RNA biogenesis and processing (PubMed:23519609). Preferentially binds, via its two RNA recognition motifs RRM1 and RRM2, to GU-repeats on RNA molecules predominantly localized within long introns and in the 3'UTR of mRNAs (PubMed:23519609, PubMed:24240615, PubMed:24464995). In turn, regulates the splicing of many non-coding and protein-coding RNAs including proteins involved in neuronal survival, as well as mRNAs that encode proteins relevant for neurodegenerative diseases (PubMed:21358640, PubMed:29438978). Plays a role in maintaining mitochondrial homeostasis by regulating the processing of mitochondrial transcripts (PubMed:28794432). Regulates also mRNA stability by recruiting CNOT7/CAF1 deadenylase on mRNA 3'UTR leading to poly(A) tail deadenylation and thus shortening (PubMed:30520513). In response to oxidative insult, associates with stalled ribosomes localized to stress granules (SGs) and contributes to cell survival (PubMed:23398327, PubMed:19765185). Participates also in the normal skeletal muscle formation and regeneration, forming cytoplasmic myo-granules and binding mRNAs that encode sarcomeric proteins (PubMed:30464263). Plays a role in the maintenance of the circadian clock periodicity via stabilization of the CRY1 and CRY2 proteins in a FBXL3-dependent manner (PubMed:27123980). Negatively regulates the expression of CDK6 (PubMed:19760257). Regulates the expression of HDAC6, ATG7 and VCP in a PPIA/CYPA-dependent manner (PubMed:25678563). {ECO:0000269|PubMed:11285240, ECO:0000269|PubMed:17481916, ECO:0000269|PubMed:19760257, ECO:0000269|PubMed:19765185, ECO:0000269|PubMed:21358640, ECO:0000269|PubMed:23398327, ECO:0000269|PubMed:23519609, ECO:0000269|PubMed:24240615, ECO:0000269|PubMed:24464995, ECO:0000269|PubMed:25678563, ECO:0000269|PubMed:27123980, ECO:0000269|PubMed:28794432, ECO:0000269|PubMed:29438978, ECO:0000269|PubMed:30464263, ECO:0000269|PubMed:30520513}. | 3'-UTR-mediated mRNA destabilization [GO:0061158]; 3'-UTR-mediated mRNA stabilization [GO:0070935]; amyloid fibril formation [GO:1990000]; mRNA processing [GO:0006397]; negative regulation by host of viral transcription [GO:0043922]; negative regulation of gene expression [GO:0010629]; negative regulation of protein phosphorylation [GO:0001933]; nuclear inner membrane organization [GO:0071765]; positive regulation of insulin secretion [GO:0032024]; positive regulation of protein import into nucleus [GO:0042307]; regulation of apoptotic process [GO:0042981]; regulation of cell cycle [GO:0051726]; regulation of circadian rhythm [GO:0042752]; regulation of gene expression [GO:0010468]; regulation of protein stability [GO:0031647]; response to endoplasmic reticulum stress [GO:0034976]; rhythmic process [GO:0048511]; RNA splicing [GO:0008380] | GO:0000978; GO:0001933; GO:0003677; GO:0003690; GO:0003723; GO:0003730; GO:0005634; GO:0005654; GO:0005726; GO:0005739; GO:0006397; GO:0008289; GO:0008380; GO:0010468; GO:0010494; GO:0010629; GO:0016607; GO:0031647; GO:0032024; GO:0034976; GO:0035061; GO:0042307; GO:0042752; GO:0042802; GO:0042981; GO:0043232; GO:0043922; GO:0048511; GO:0051726; GO:0061158; GO:0070935; GO:0071765; GO:0097157; GO:0140693; GO:1990000 |
| ENSG00000132646 | PCNA | -0.074000581 | P12004 | PCNA_HUMAN | Proliferating cell nuclear antigen (PCNA) (Cyclin) | PCNA | 261 | FUNCTION: Auxiliary protein of DNA polymerase delta and epsilon, is involved in the control of eukaryotic DNA replication by increasing the polymerase's processibility during elongation of the leading strand (PubMed:35585232). Induces a robust stimulatory effect on the 3'-5' exonuclease and 3'-phosphodiesterase, but not apurinic-apyrimidinic (AP) endonuclease, APEX2 activities. Has to be loaded onto DNA in order to be able to stimulate APEX2. Plays a key role in DNA damage response (DDR) by being conveniently positioned at the replication fork to coordinate DNA replication with DNA repair and DNA damage tolerance pathways (PubMed:24939902). Acts as a loading platform to recruit DDR proteins that allow completion of DNA replication after DNA damage and promote postreplication repair: Monoubiquitinated PCNA leads to recruitment of translesion (TLS) polymerases, while 'Lys-63'-linked polyubiquitination of PCNA is involved in error-free pathway and employs recombination mechanisms to synthesize across the lesion (PubMed:24695737). {ECO:0000269|PubMed:18719106, ECO:0000269|PubMed:19443450, ECO:0000269|PubMed:24695737, ECO:0000269|PubMed:24939902, ECO:0000269|PubMed:35585232}. | base-excision repair, gap-filling [GO:0006287]; cellular response to hydrogen peroxide [GO:0070301]; cellular response to UV [GO:0034644]; cellular response to xenobiotic stimulus [GO:0071466]; epithelial cell differentiation [GO:0030855]; estrous cycle [GO:0044849]; heart development [GO:0007507]; leading strand elongation [GO:0006272]; liver regeneration [GO:0097421]; mismatch repair [GO:0006298]; mitotic telomere maintenance via semi-conservative replication [GO:1902990]; negative regulation of transcription by RNA polymerase II [GO:0000122]; positive regulation of deoxyribonuclease activity [GO:0032077]; positive regulation of DNA repair [GO:0045739]; positive regulation of DNA replication [GO:0045740]; positive regulation of DNA-directed DNA polymerase activity [GO:1900264]; replication fork processing [GO:0031297]; response to cadmium ion [GO:0046686]; response to dexamethasone [GO:0071548]; response to estradiol [GO:0032355]; response to L-glutamate [GO:1902065]; translesion synthesis [GO:0019985] | GO:0000122; GO:0000307; GO:0000701; GO:0000781; GO:0000785; GO:0001673; GO:0003682; GO:0003684; GO:0005634; GO:0005652; GO:0005654; GO:0005657; GO:0005813; GO:0006272; GO:0006287; GO:0006298; GO:0007507; GO:0016604; GO:0019899; GO:0019985; GO:0030331; GO:0030337; GO:0030855; GO:0030894; GO:0030971; GO:0031297; GO:0032077; GO:0032139; GO:0032355; GO:0032405; GO:0034644; GO:0035035; GO:0042802; GO:0043596; GO:0043626; GO:0044849; GO:0044877; GO:0045739; GO:0045740; GO:0046686; GO:0070062; GO:0070182; GO:0070301; GO:0070557; GO:0071466; GO:0071548; GO:0097421; GO:1900264; GO:1902065; GO:1902990 |
| ENSG00000143436 | MRPL9 | -0.074000581 | Q9BYD2 | RM09_HUMAN | Large ribosomal subunit protein bL9m (39S ribosomal protein L9, mitochondrial) (L9mt) (MRP-L9) | MRPL9 | 267 |  | mitochondrial translation [GO:0032543]; translation [GO:0006412] | GO:0003723; GO:0003735; GO:0005739; GO:0005743; GO:0005761; GO:0005762; GO:0006412; GO:0032543 |
| ENSG00000178952 | TUFM | -0.074000581 | P49411 | EFTU_HUMAN | Elongation factor Tu, mitochondrial (EF-Tu) (P43) | TUFM | 455 | FUNCTION: Promotes the GTP-dependent binding of aminoacyl-tRNA to the A-site of ribosomes during protein biosynthesis. Also plays a role in the regulation of autophagy and innate immunity. Recruits ATG5-ATG12 and NLRX1 at mitochondria and serves as a checkpoint of the RIGI-MAVS pathway. In turn, inhibits RLR-mediated type I interferon while promoting autophagy. {ECO:0000269|PubMed:22749352, ECO:0000269|PubMed:28407488}. | mitochondrial translational elongation [GO:0070125]; response to ethanol [GO:0045471]; translational elongation [GO:0006414] | GO:0003723; GO:0003746; GO:0003924; GO:0005525; GO:0005739; GO:0005741; GO:0006414; GO:0016020; GO:0042645; GO:0045202; GO:0045471; GO:0070062; GO:0070125 |
| ENSG00000099995 | SF3A1 | -0.089267338 | Q15459 | SF3A1_HUMAN | Splicing factor 3A subunit 1 (SF3a120) (Spliceosome-associated protein 114) (SAP 114) | SF3A1 SAP114 | 793 | FUNCTION: Involved in pre-mRNA splicing as a component of the splicing factor SF3A complex that contributes to the assembly of the 17S U2 snRNP, and the subsequent assembly of the pre-spliceosome 'E' complex and the pre-catalytic spliceosome 'A' complex (PubMed:10882114, PubMed:11533230). Involved in pre-mRNA splicing as a component of pre-catalytic spliceosome 'B' complexes (PubMed:29360106, PubMed:30315277). {ECO:0000269|PubMed:10882114, ECO:0000269|PubMed:11533230, ECO:0000269|PubMed:29360106, ECO:0000269|PubMed:30315277}. | mRNA 3'-splice site recognition [GO:0000389]; mRNA cis splicing, via spliceosome [GO:0045292]; mRNA processing [GO:0006397]; mRNA splicing, via spliceosome [GO:0000398]; U2-type prespliceosome assembly [GO:1903241] | GO:0000389; GO:0000398; GO:0003723; GO:0005634; GO:0005654; GO:0005681; GO:0005684; GO:0005686; GO:0006397; GO:0016607; GO:0045292; GO:0071004; GO:0071005; GO:0071013; GO:1903241 |
| ENSG00000115942 | ORC2 | -0.089267338 | Q13416 | ORC2_HUMAN | Origin recognition complex subunit 2 | ORC2 ORC2L | 577 | FUNCTION: Component of the origin recognition complex (ORC) that binds origins of replication. DNA-binding is ATP-dependent. The specific DNA sequences that define origins of replication have not been identified yet. ORC is required to assemble the pre-replication complex necessary to initiate DNA replication. Binds histone H3 and H4 trimethylation marks H3K9me3, H3K20me3 and H4K27me3. Stabilizes LRWD1, by protecting it from ubiquitin-mediated proteasomal degradation. Also stabilizes ORC3. {ECO:0000269|PubMed:22427655, ECO:0000269|PubMed:22935713}. | DNA replication initiation [GO:0006270]; negative regulation of transcription by RNA polymerase II [GO:0000122] | GO:0000122; GO:0000781; GO:0000792; GO:0000808; GO:0000939; GO:0003688; GO:0005634; GO:0005654; GO:0005664; GO:0005813; GO:0006270; GO:0016020 |
| **ENSG00000124614** | **RPS10** | **-0.089267338** | **P46783** | **RS10_HUMAN** | **Small ribosomal subunit protein eS10 (40S ribosomal protein S10)** | **RPS10** | **165** | **FUNCTION: Component of the 40S ribosomal subunit (PubMed:23636399). The ribosome is a large ribonucleoprotein complex responsible for the synthesis of proteins in the cell (PubMed:23636399). {ECO:0000269|PubMed:23636399}.** | **cytoplasmic translation [GO:0002181]; translation [GO:0006412]** | **GO:0002181; GO:0003723; GO:0003735; GO:0005654; GO:0005730; GO:0005737; GO:0005829; GO:0005840; GO:0005925; GO:0006412; GO:0016020; GO:0022626; GO:0022627** |
| ENSG00000164163 | ABCE1 | -0.089267338 | P61221 | ABCE1_HUMAN | ATP-binding cassette sub-family E member 1 (EC 3.6.5.-) (2'-5'-oligoadenylate-binding protein) (HuHP68) (RNase L inhibitor) (Ribonuclease 4 inhibitor) (RNS4I) | ABCE1 RLI RNASEL1 RNASELI RNS4I OK/SW-cl.40 | 599 | FUNCTION: Nucleoside-triphosphatase (NTPase) involved in ribosome recycling by mediating ribosome disassembly (PubMed:20122402, PubMed:21448132). Able to hydrolyze ATP, GTP, UTP and CTP (PubMed:20122402). Splits ribosomes into free 60S subunits and tRNA- and mRNA-bound 40S subunits (PubMed:20122402, PubMed:21448132). Acts either after canonical termination facilitated by release factors (ETF1/eRF1) or after recognition of stalled and vacant ribosomes by mRNA surveillance factors (PELO/Pelota) (PubMed:20122402, PubMed:21448132). Involved in the No-Go Decay (NGD) pathway: recruited to stalled ribosomes by the Pelota-HBS1L complex, and drives the disassembly of stalled ribosomes, followed by degradation of damaged mRNAs as part of the NGD pathway (PubMed:21448132). Also plays a role in quality control of translation of mitochondrial outer membrane-localized mRNA (PubMed:29861391). As part of the PINK1-regulated signaling, ubiquitinated by CNOT4 upon mitochondria damage; this modification generates polyubiquitin signals that recruit autophagy receptors to the mitochondrial outer membrane and initiate mitophagy (PubMed:29861391). RNASEL-specific protein inhibitor which antagonizes the binding of 2-5A (5'-phosphorylated 2',5'-linked oligoadenylates) to RNASEL (PubMed:9660177). Negative regulator of the anti-viral effect of the interferon-regulated 2-5A/RNASEL pathway (PubMed:9660177, PubMed:9847332, PubMed:11585831). {ECO:0000269|PubMed:11585831, ECO:0000269|PubMed:20122402, ECO:0000269|PubMed:21448132, ECO:0000269|PubMed:29861391, ECO:0000269|PubMed:9660177, ECO:0000269|PubMed:9847332}.; FUNCTION: (Microbial infection) May act as a chaperone for post-translational events during HIV-1 capsid assembly. {ECO:0000269|PubMed:9847332}.; FUNCTION: (Microbial infection) Plays a role in the down-regulation of the 2-5A/RNASEL pathway during encephalomyocarditis virus (EMCV) and HIV-1 infections. {ECO:0000269|PubMed:9660177}. | negative regulation of endoribonuclease activity [GO:0060702]; regulation of translation [GO:0006417]; rescue of stalled ribosome [GO:0072344]; ribosomal subunit export from nucleus [GO:0000054]; ribosome disassembly [GO:0032790]; translational initiation [GO:0006413]; translational termination [GO:0006415] | GO:0000054; GO:0003924; GO:0005506; GO:0005524; GO:0005737; GO:0005739; GO:0005759; GO:0005829; GO:0006413; GO:0006415; GO:0006417; GO:0016020; GO:0016887; GO:0017111; GO:0022626; GO:0032790; GO:0043024; GO:0043273; GO:0051539; GO:0060698; GO:0060702; GO:0072344 |
| ENSG00000167325 | RRM1 | -0.089267338 | P23921 | RIR1_HUMAN | Ribonucleoside-diphosphate reductase large subunit (EC 1.17.4.1) (Ribonucleoside-diphosphate reductase subunit M1) (Ribonucleotide reductase large subunit) | RRM1 RR1 | 792 | FUNCTION: Provides the precursors necessary for DNA synthesis. Catalyzes the biosynthesis of deoxyribonucleotides from the corresponding ribonucleotides. | 2'-deoxyribonucleotide biosynthetic process [GO:0009265]; cell proliferation in forebrain [GO:0021846]; deoxyribonucleotide biosynthetic process [GO:0009263]; DNA repair [GO:0006281]; DNA synthesis involved in DNA repair [GO:0000731]; male gonad development [GO:0008584]; mitochondrial DNA replication [GO:0006264]; positive regulation of G0 to G1 transition [GO:0070318]; positive regulation of G1/S transition of mitotic cell cycle [GO:1900087]; positive regulation of G2/M transition of mitotic cell cycle [GO:0010971]; protein heterotetramerization [GO:0051290]; pyrimidine nucleobase metabolic process [GO:0006206]; response to ionizing radiation [GO:0010212]; retina development in camera-type eye [GO:0060041]; ribonucleoside diphosphate metabolic process [GO:0009185] | GO:0000731; GO:0004748; GO:0005524; GO:0005635; GO:0005739; GO:0005829; GO:0005971; GO:0006206; GO:0006264; GO:0006281; GO:0008584; GO:0009185; GO:0009263; GO:0009265; GO:0010212; GO:0010971; GO:0021846; GO:0042802; GO:0042995; GO:0043025; GO:0051290; GO:0060041; GO:0061731; GO:0070318; GO:0097718; GO:1900087 |
| **ENSG00000041357** | **PSMA4** | **-0.104697379** | **P25789** | **PSA4_HUMAN** | **Proteasome subunit alpha type-4 (Macropain subunit C9) (Multicatalytic endopeptidase complex subunit C9) (Proteasome component C9) (Proteasome subunit L)** | **PSMA4 HC9 PSC9** | **261** | **FUNCTION: Component of the 20S core proteasome complex involved in the proteolytic degradation of most intracellular proteins. This complex plays numerous essential roles within the cell by associating with different regulatory particles. Associated with two 19S regulatory particles, forms the 26S proteasome and thus participates in the ATP-dependent degradation of ubiquitinated proteins. The 26S proteasome plays a key role in the maintenance of protein homeostasis by removing misfolded or damaged proteins that could impair cellular functions, and by removing proteins whose functions are no longer required. Associated with the PA200 or PA28, the 20S proteasome mediates ubiquitin-independent protein degradation. This type of proteolysis is required in several pathways including spermatogenesis (20S-PA200 complex) or generation of a subset of MHC class I-presented antigenic peptides (20S-PA28 complex). {ECO:0000269|PubMed:15244466, ECO:0000269|PubMed:27176742, ECO:0000269|PubMed:8610016}.** | **proteasomal protein catabolic process [GO:0010498]; proteasome-mediated ubiquitin-dependent protein catabolic process [GO:0043161]** | **GO:0000502; GO:0000932; GO:0005634; GO:0005654; GO:0005737; GO:0005829; GO:0005839; GO:0010498; GO:0019773; GO:0043161; GO:0043231; GO:0070062** |
| ENSG00000125743 | SNRPD2 | -0.104697379 | P62316 | SMD2_HUMAN | Small nuclear ribonucleoprotein Sm D2 (Sm-D2) (snRNP core protein D2) | SNRPD2 SNRPD1 | 118 | FUNCTION: Plays a role in pre-mRNA splicing as a core component of the spliceosomal U1, U2, U4 and U5 small nuclear ribonucleoproteins (snRNPs), the building blocks of the spliceosome (PubMed:11991638, PubMed:18984161, PubMed:19325628, PubMed:23333303, PubMed:25555158, PubMed:26912367, PubMed:28502770, PubMed:28781166, PubMed:28076346). Component of both the pre-catalytic spliceosome B complex and activated spliceosome C complexes (PubMed:11991638, PubMed:28502770, PubMed:28781166, PubMed:28076346). As a component of the minor spliceosome, involved in the splicing of U12-type introns in pre-mRNAs (PubMed:15146077). {ECO:0000269|PubMed:11991638, ECO:0000269|PubMed:15146077, ECO:0000269|PubMed:18984161, ECO:0000269|PubMed:19325628, ECO:0000269|PubMed:23333303, ECO:0000269|PubMed:25555158, ECO:0000269|PubMed:26912367, ECO:0000269|PubMed:28076346, ECO:0000269|PubMed:28502770, ECO:0000269|PubMed:28781166}. | 7-methylguanosine cap hypermethylation [GO:0036261]; mRNA splicing, via spliceosome [GO:0000398]; RNA splicing [GO:0008380]; spliceosomal complex assembly [GO:0000245]; spliceosomal snRNP assembly [GO:0000387]; U2-type prespliceosome assembly [GO:1903241] | GO:0000245; GO:0000387; GO:0000398; GO:0003723; GO:0005634; GO:0005654; GO:0005681; GO:0005682; GO:0005685; GO:0005686; GO:0005687; GO:0005689; GO:0005829; GO:0008380; GO:0030532; GO:0034709; GO:0034715; GO:0034719; GO:0036261; GO:0046540; GO:0070062; GO:0071005; GO:0071007; GO:0071011; GO:0071013; GO:1903241; GO:1990446 |
| ENSG00000144381 | HSPD1 | -0.104697379 | P10809 | CH60_HUMAN | 60 kDa heat shock protein, mitochondrial (EC 5.6.1.7) (60 kDa chaperonin) (Chaperonin 60) (CPN60) (Heat shock protein 60) (HSP-60) (Hsp60) (HuCHA60) (Mitochondrial matrix protein P1) (P60 lymphocyte protein) | HSPD1 HSP60 | 573 | FUNCTION: Chaperonin implicated in mitochondrial protein import and macromolecular assembly. Together with Hsp10, facilitates the correct folding of imported proteins. May also prevent misfolding and promote the refolding and proper assembly of unfolded polypeptides generated under stress conditions in the mitochondrial matrix (PubMed:1346131, PubMed:11422376). The functional units of these chaperonins consist of heptameric rings of the large subunit Hsp60, which function as a back-to-back double ring. In a cyclic reaction, Hsp60 ring complexes bind one unfolded substrate protein per ring, followed by the binding of ATP and association with 2 heptameric rings of the co-chaperonin Hsp10. This leads to sequestration of the substrate protein in the inner cavity of Hsp60 where, for a certain period of time, it can fold undisturbed by other cell components. Synchronous hydrolysis of ATP in all Hsp60 subunits results in the dissociation of the chaperonin rings and the release of ADP and the folded substrate protein (Probable). {ECO:0000269|PubMed:11422376, ECO:0000269|PubMed:1346131, ECO:0000305|PubMed:25918392}. | 'de novo' protein folding [GO:0006458]; activation of cysteine-type endopeptidase activity involved in apoptotic process [GO:0006919]; apoptotic mitochondrial changes [GO:0008637]; B cell activation [GO:0042113]; B cell proliferation [GO:0042100]; biological process involved in interaction with symbiont [GO:0051702]; cellular response to interleukin-7 [GO:0098761]; chaperone-mediated protein complex assembly [GO:0051131]; isotype switching to IgG isotypes [GO:0048291]; mitochondrial unfolded protein response [GO:0034514]; MyD88-dependent toll-like receptor signaling pathway [GO:0002755]; negative regulation of apoptotic process [GO:0043066]; positive regulation of apoptotic process [GO:0043065]; positive regulation of interferon-alpha production [GO:0032727]; positive regulation of interleukin-10 production [GO:0032733]; positive regulation of interleukin-12 production [GO:0032735]; positive regulation of interleukin-6 production [GO:0032755]; positive regulation of macrophage activation [GO:0043032]; positive regulation of T cell activation [GO:0050870]; positive regulation of T cell mediated immune response to tumor cell [GO:0002842]; positive regulation of type II interferon production [GO:0032729]; protein folding [GO:0006457]; protein import into mitochondrial intermembrane space [GO:0045041]; protein maturation [GO:0051604]; protein refolding [GO:0042026]; protein stabilization [GO:0050821]; response to cold [GO:0009409]; response to unfolded protein [GO:0006986]; T cell activation [GO:0042110] | GO:0001530; GO:0002039; GO:0002755; GO:0002842; GO:0003688; GO:0003697; GO:0003723; GO:0003725; GO:0005524; GO:0005615; GO:0005737; GO:0005739; GO:0005743; GO:0005759; GO:0005769; GO:0005829; GO:0005886; GO:0005905; GO:0006457; GO:0006458; GO:0006919; GO:0006986; GO:0008035; GO:0008637; GO:0009409; GO:0009986; GO:0016020; GO:0016853; GO:0016887; GO:0019899; GO:0030135; GO:0030141; GO:0031625; GO:0032727; GO:0032729; GO:0032733; GO:0032735; GO:0032755; GO:0032991; GO:0034185; GO:0034186; GO:0034514; GO:0042026; GO:0042100; GO:0042110; GO:0042113; GO:0043032; GO:0043065; GO:0043066; GO:0045041; GO:0046696; GO:0048291; GO:0050821; GO:0050870; GO:0051082; GO:0051087; GO:0051131; GO:0051604; GO:0051702; GO:0070062; GO:0097225; GO:0097524; GO:0098761; GO:0140494; GO:0140662 |
| ENSG00000177879 | AP3S1 | -0.104697379 | Q92572 | AP3S1_HUMAN | AP-3 complex subunit sigma-1 (AP-3 complex subunit sigma-3A) (Adaptor-related protein complex 3 subunit sigma-1) (Clathrin-associated/assembly/adaptor protein, small 3) (Sigma-3A-adaptin) (Sigma3A-adaptin) (Sigma-adaptin 3a) | AP3S1 CLAPS3 | 193 | FUNCTION: Part of the AP-3 complex, an adaptor-related complex which is not clathrin-associated. The complex is associated with the Golgi region as well as more peripheral structures. It facilitates the budding of vesicles from the Golgi membrane and may be directly involved in trafficking to lysosomes. In concert with the BLOC-1 complex, AP-3 is required to target cargos into vesicles assembled at cell bodies for delivery into neurites and nerve terminals. | anterograde axonal transport [GO:0008089]; anterograde synaptic vesicle transport [GO:0048490]; clathrin-coated vesicle cargo loading, AP-3-mediated [GO:0035654]; Golgi to vacuole transport [GO:0006896]; insulin receptor signaling pathway [GO:0008286]; intracellular protein transport [GO:0006886]; intracellular transport [GO:0046907]; melanosome assembly [GO:1903232]; platelet dense granule organization [GO:0060155]; synaptic vesicle coating [GO:0016183]; synaptic vesicle recycling [GO:0036465]; vesicle-mediated transport [GO:0016192] | GO:0005769; GO:0005794; GO:0006886; GO:0006896; GO:0008089; GO:0008286; GO:0016183; GO:0016192; GO:0030119; GO:0030123; GO:0030133; GO:0030659; GO:0035654; GO:0036465; GO:0043231; GO:0046907; GO:0048490; GO:0060155; GO:0098793; GO:1903232; GO:1904115 |
| ENSG00000065427 | KARS1 | -0.120294234 | Q15046 | SYK_HUMAN | Lysine--tRNA ligase (EC 2.7.7.-) (EC 6.1.1.6) (Lysyl-tRNA synthetase) (LysRS) | KARS1 KARS KIAA0070 | 597 | FUNCTION: Catalyzes the specific attachment of an amino acid to its cognate tRNA in a 2 step reaction: the amino acid (AA) is first activated by ATP to form AA-AMP and then transferred to the acceptor end of the tRNA (PubMed:9278442, PubMed:18029264, PubMed:18272479). When secreted, acts as a signaling molecule that induces immune response through the activation of monocyte/macrophages (PubMed:15851690). Catalyzes the synthesis of the signaling molecule diadenosine tetraphosphate (Ap4A), and thereby mediates disruption of the complex between HINT1 and MITF and the concomitant activation of MITF transcriptional activity (PubMed:5338216, PubMed:14975237, PubMed:19524539, PubMed:23159739). {ECO:0000269|PubMed:14975237, ECO:0000269|PubMed:15851690, ECO:0000269|PubMed:18029264, ECO:0000269|PubMed:19524539, ECO:0000269|PubMed:28887846, ECO:0000269|PubMed:5338216, ECO:0000269|PubMed:9278442}.; FUNCTION: (Microbial infection) Interacts with HIV-1 virus GAG protein, facilitating the selective packaging of tRNA(3)(Lys), the primer for reverse transcription initiation. {ECO:0000269|PubMed:15220430}. | basophil activation involved in immune response [GO:0002276]; diadenosine tetraphosphate biosynthetic process [GO:0015966]; ERK1 and ERK2 cascade [GO:0070371]; lysyl-tRNA aminoacylation [GO:0006430]; positive regulation of DNA-templated transcription [GO:0045893]; positive regulation of inflammatory response to antigenic stimulus [GO:0002863]; positive regulation of macrophage activation [GO:0043032]; response to X-ray [GO:0010165]; tRNA processing [GO:0008033] | GO:0000049; GO:0002276; GO:0002863; GO:0003877; GO:0004824; GO:0005524; GO:0005615; GO:0005634; GO:0005739; GO:0005759; GO:0005829; GO:0005886; GO:0006430; GO:0008033; GO:0010165; GO:0015966; GO:0016597; GO:0017101; GO:0042802; GO:0042803; GO:0043032; GO:0045893; GO:0070371 |
| ENSG00000077312 | SNRPA | -0.120294234 | P09012 | SNRPA_HUMAN | U1 small nuclear ribonucleoprotein A (U1 snRNP A) (U1-A) (U1A) | SNRPA | 282 | FUNCTION: Component of the spliceosomal U1 snRNP, which is essential for recognition of the pre-mRNA 5' splice-site and the subsequent assembly of the spliceosome. U1 snRNP is the first snRNP to interact with pre-mRNA. This interaction is required for the subsequent binding of U2 snRNP and the U4/U6/U5 tri-snRNP. SNRPA binds stem loop II of U1 snRNA. In a snRNP-free form (SF-A) may be involved in coupled pre-mRNA splicing and polyadenylation process. May bind preferentially to the 5'-UGCAC-3' motif on RNAs. {ECO:0000269|PubMed:9848648}. | mRNA splicing, via spliceosome [GO:0000398] | GO:0000398; GO:0003677; GO:0003723; GO:0005634; GO:0005654; GO:0005681; GO:0005685; GO:0030619; GO:0042802; GO:0046540; GO:1990446 |
| ENSG00000138385 | SSB | -0.120294234 | P05455 | LA_HUMAN | Lupus La protein (La autoantigen) (La ribonucleoprotein) (Sjoegren syndrome type B antigen) (SS-B) | SSB | 408 | FUNCTION: Binds to the 3' poly(U) terminus of nascent RNA polymerase III transcripts, protecting them from exonuclease digestion and facilitating their folding and maturation (PubMed:3192525, PubMed:2470590). In case of Coxsackievirus B3 infection, binds to the viral internal ribosome entry site (IRES) and stimulates the IRES-mediated translation (PubMed:12384597). {ECO:0000269|PubMed:12384597, ECO:0000269|PubMed:2470590, ECO:0000269|PubMed:3192525}. | histone mRNA metabolic process [GO:0008334]; IRES-dependent viral translational initiation [GO:0075522]; nuclear histone mRNA catabolic process [GO:0071045]; positive regulation of translation [GO:0045727]; protein localization to cytoplasmic stress granule [GO:1903608]; tRNA 3'-end processing [GO:0042780]; tRNA 5'-leader removal [GO:0001682]; tRNA export from nucleus [GO:0006409]; tRNA modification [GO:0006400]; tRNA processing [GO:0008033] | GO:0000049; GO:0000781; GO:0001682; GO:0003723; GO:0003729; GO:0005634; GO:0005737; GO:0005829; GO:0006400; GO:0006409; GO:0008033; GO:0008266; GO:0008334; GO:0010494; GO:0042780; GO:0045727; GO:0071045; GO:0075522; GO:1903608; GO:1990825; GO:1990904 |
| ENSG00000171793 | CTPS1 | -0.13606155 | P17812 | PYRG1_HUMAN | CTP synthase 1 (EC 6.3.4.2) (CTP synthetase 1) (UTP--ammonia ligase 1) | CTPS1 CTPS | 591 | FUNCTION: This enzyme is involved in the de novo synthesis of CTP, a precursor of DNA, RNA and phospholipids. Catalyzes the ATP-dependent amination of UTP to CTP with either L-glutamine or ammonia as a source of nitrogen. This enzyme and its product, CTP, play a crucial role in the proliferation of activated lymphocytes and therefore in immunity. {ECO:0000269|PubMed:16179339, ECO:0000269|PubMed:24870241}. | 'de novo' CTP biosynthetic process [GO:0044210]; B cell proliferation [GO:0042100]; CTP biosynthetic process [GO:0006241]; glutamine metabolic process [GO:0006541]; nucleobase-containing compound metabolic process [GO:0006139]; pyrimidine nucleobase biosynthetic process [GO:0019856]; response to xenobiotic stimulus [GO:0009410]; T cell proliferation [GO:0042098] | GO:0003883; GO:0005524; GO:0005737; GO:0005829; GO:0006139; GO:0006241; GO:0006541; GO:0009410; GO:0016020; GO:0019856; GO:0042098; GO:0042100; GO:0042802; GO:0044210; GO:0097268 |
| **ENSG00000083845** | **RPS5** | **-0.152003093** | **P46782** | **RS5_HUMAN** | **Small ribosomal subunit protein uS7 (40S ribosomal protein S5) [Cleaved into: Small ribosomal subunit protein uS7, N-terminally processed (40S ribosomal protein S5, N-terminally processed)]** | **RPS5** | **204** | **FUNCTION: Component of the small ribosomal subunit (PubMed:23636399). The ribosome is a large ribonucleoprotein complex responsible for the synthesis of proteins in the cell (PubMed:23636399). Part of the small subunit (SSU) processome, first precursor of the small eukaryotic ribosomal subunit. During the assembly of the SSU processome in the nucleolus, many ribosome biogenesis factors, an RNA chaperone and ribosomal proteins associate with the nascent pre-rRNA and work in concert to generate RNA folding, modifications, rearrangements and cleavage as well as targeted degradation of pre-ribosomal RNA by the RNA exosome (PubMed:34516797). {ECO:0000269|PubMed:23636399, ECO:0000269|PubMed:34516797}.** | **cytoplasmic translation [GO:0002181]; regulation of translational fidelity [GO:0006450]; ribosomal small subunit assembly [GO:0000028]; ribosomal small subunit biogenesis [GO:0042274]; translation [GO:0006412]; translational initiation [GO:0006413]** | **GO:0000028; GO:0002181; GO:0003723; GO:0003729; GO:0003735; GO:0005654; GO:0005730; GO:0005737; GO:0005829; GO:0005840; GO:0005925; GO:0006412; GO:0006413; GO:0006450; GO:0016020; GO:0019843; GO:0022626; GO:0022627; GO:0032040; GO:0042274; GO:0070062; GO:1990904** |
| ENSG00000122565 | CBX3 | -0.152003093 | Q13185 | CBX3_HUMAN | Chromobox protein homolog 3 (HECH) (Heterochromatin protein 1 homolog gamma) (HP1 gamma) (Modifier 2 protein) | CBX3 | 183 | FUNCTION: Seems to be involved in transcriptional silencing in heterochromatin-like complexes. Recognizes and binds histone H3 tails methylated at 'Lys-9', leading to epigenetic repression. May contribute to the association of the heterochromatin with the inner nuclear membrane through its interaction with lamin B receptor (LBR). Involved in the formation of functional kinetochore through interaction with MIS12 complex proteins. Contributes to the conversion of local chromatin to a heterochromatin-like repressive state through H3 'Lys-9' trimethylation, mediates the recruitment of the methyltransferases SUV39H1 and/or SUV39H2 by the PER complex to the E-box elements of the circadian target genes such as PER2 itself or PER1. Mediates the recruitment of NIPBL to sites of DNA damage at double-strand breaks (DSBs) (PubMed:28167679). {ECO:0000250|UniProtKB:P23198, ECO:0000269|PubMed:28167679}. | chromatin organization [GO:0006325]; chromatin remodeling [GO:0006338]; DNA damage response [GO:0006974]; heterochromatin formation [GO:0031507]; negative regulation of DNA-templated transcription [GO:0045892]; negative regulation of transcription by RNA polymerase II [GO:0000122]; rhythmic process [GO:0048511] | GO:0000122; GO:0000775; GO:0000779; GO:0000781; GO:0000785; GO:0000791; GO:0000792; GO:0001221; GO:0003682; GO:0005634; GO:0005635; GO:0005637; GO:0005654; GO:0005721; GO:0005819; GO:0006325; GO:0006338; GO:0006974; GO:0019899; GO:0019904; GO:0031507; GO:0035064; GO:0042802; GO:0045892; GO:0048511; GO:0061793; GO:0090575; GO:0090734; GO:1990226 |
| ENSG00000142864 | SERBP1 | -0.152003093 | Q8NC51 | SERB1_HUMAN | SERPINE1 mRNA-binding protein 1 (PAI1 RNA-binding protein 1) (PAI-RBP1) (Plasminogen activator inhibitor 1 RNA-binding protein) | SERBP1 PAIRBP1 CGI-55 | 408 | FUNCTION: Ribosome-binding protein that promotes ribosome hibernation, a process during which ribosomes are stabilized in an inactive state and preserved from proteasomal degradation (PubMed:36691768). Acts via its association with EEF2/eEF2 factor, sequestering EEF2/eEF2 at the A-site of the ribosome and promoting ribosome stabilization and storage in an inactive state (By similarity). May also play a role in the regulation of mRNA stability: binds to the 3'-most 134 nt of the SERPINE1/PAI1 mRNA, a region which confers cyclic nucleotide regulation of message decay (PubMed:11001948). Seems to play a role in PML-nuclear bodies formation (PubMed:28695742). {ECO:0000250|UniProtKB:Q9CY58, ECO:0000269|PubMed:11001948, ECO:0000269|PubMed:28695742, ECO:0000269|PubMed:36691768}. | negative regulation of translation [GO:0017148]; PML body organization [GO:0030578]; regulation of mRNA stability [GO:0043488]; ribosome hibernation [GO:0141014] | GO:0003723; GO:0003730; GO:0005634; GO:0005737; GO:0005829; GO:0016020; GO:0017148; GO:0030371; GO:0030578; GO:0032183; GO:0043022; GO:0043488; GO:0045296; GO:0048471; GO:0061770; GO:0070062; GO:0141014 |
| **ENSG00000196591** | **HDAC2** | **-0.152003093** | **Q92769** | **HDAC2_HUMAN** | **Histone deacetylase 2 (HD2) (EC 3.5.1.98) (Protein deacylase HDAC2) (EC 3.5.1.-)** | **HDAC2** | **488** | **FUNCTION: Histone deacetylase that catalyzes the deacetylation of lysine residues on the N-terminal part of the core histones (H2A, H2B, H3 and H4) (PubMed:28497810). Histone deacetylation gives a tag for epigenetic repression and plays an important role in transcriptional regulation, cell cycle progression and developmental events (By similarity). Histone deacetylases act via the formation of large multiprotein complexes (By similarity). Forms transcriptional repressor complexes by associating with MAD, SIN3, YY1 and N-COR (PubMed:12724404). Component of a RCOR/GFI/KDM1A/HDAC complex that suppresses, via histone deacetylase (HDAC) recruitment, a number of genes implicated in multilineage blood cell development (By similarity). Acts as a component of the histone deacetylase NuRD complex which participates in the remodeling of chromatin (PubMed:16428440, PubMed:28977666). Also deacetylates non-histone targets: deacetylates TSHZ3, thereby regulating its transcriptional repressor activity (PubMed:19343227). May be involved in the transcriptional repression of circadian target genes, such as PER1, mediated by CRY1 through histone deacetylation (By similarity). Involved in MTA1-mediated transcriptional corepression of TFF1 and CDKN1A (PubMed:21965678). In addition to protein deacetylase activity, also acts as protein-lysine deacylase by recognizing other acyl groups: catalyzes removal of (2E)-butenoyl (crotonyl) and 2-hydroxyisobutanoyl (2-hydroxyisobutyryl) acyl groups from lysine residues, leading to protein decrotonylation and de-2-hydroxyisobutyrylation, respectively (PubMed:28497810, PubMed:29192674). {ECO:0000250|UniProtKB:P70288, ECO:0000269|PubMed:12724404, ECO:0000269|PubMed:16428440, ECO:0000269|PubMed:19343227, ECO:0000269|PubMed:21965678, ECO:0000269|PubMed:28497810, ECO:0000269|PubMed:28977666, ECO:0000269|PubMed:29192674}.** | **behavioral response to ethanol [GO:0048149]; cardiac muscle hypertrophy [GO:0003300]; cellular response to dopamine [GO:1903351]; cellular response to heat [GO:0034605]; cellular response to hydrogen peroxide [GO:0070301]; cellular response to retinoic acid [GO:0071300]; cellular response to transforming growth factor beta stimulus [GO:0071560]; chromatin remodeling [GO:0006338]; circadian regulation of gene expression [GO:0032922]; dendrite development [GO:0016358]; embryonic digit morphogenesis [GO:0042733]; epidermal cell differentiation [GO:0009913]; eyelid development in camera-type eye [GO:0061029]; fungiform papilla formation [GO:0061198]; hair follicle placode formation [GO:0060789]; heterochromatin formation [GO:0031507]; negative regulation of apoptotic process [GO:0043066]; negative regulation of cell migration [GO:0030336]; negative regulation of dendritic spine development [GO:0061000]; negative regulation of DNA-binding transcription factor activity [GO:0043433]; negative regulation of DNA-templated transcription [GO:0045892]; negative regulation of MHC class II biosynthetic process [GO:0045347]; negative regulation of neuron projection development [GO:0010977]; negative regulation of peptidyl-lysine acetylation [GO:2000757]; negative regulation of stem cell population maintenance [GO:1902455]; negative regulation of transcription by competitive promoter binding [GO:0010944]; negative regulation of transcription by RNA polymerase II [GO:0000122]; negative regulation of transforming growth factor beta receptor signaling pathway [GO:0030512]; odontogenesis of dentin-containing tooth [GO:0042475]; positive regulation of cell population proliferation [GO:0008284]; positive regulation of collagen biosynthetic process [GO:0032967]; positive regulation of DNA-templated transcription [GO:0045893]; positive regulation of epithelial to mesenchymal transition [GO:0010718]; positive regulation of interleukin-1 production [GO:0032732]; positive regulation of male mating behavior [GO:1902437]; positive regulation of oligodendrocyte differentiation [GO:0048714]; positive regulation of proteolysis [GO:0045862]; positive regulation of signaling receptor activity [GO:2000273]; positive regulation of stem cell population maintenance [GO:1902459]; positive regulation of transcription by RNA polymerase II [GO:0045944]; positive regulation of tumor necrosis factor production [GO:0032760]; positive regulation of tyrosine phosphorylation of STAT protein [GO:0042531]; regulation of cell fate specification [GO:0042659]; regulation of stem cell differentiation [GO:2000736]; response to amphetamine [GO:0001975]; response to caffeine [GO:0031000]; response to cocaine [GO:0042220]; response to hyperoxia [GO:0055093]; response to lipopolysaccharide [GO:0032496]; response to nicotine [GO:0035094]; response to xenobiotic stimulus [GO:0009410]** | **GO:0000118; GO:0000122; GO:0000781; GO:0000785; GO:0001975; GO:0003300; GO:0003682; GO:0003723; GO:0004407; GO:0005634; GO:0005654; GO:0005737; GO:0006338; GO:0008284; GO:0009410; GO:0009913; GO:0010718; GO:0010944; GO:0010977; GO:0016358; GO:0016580; GO:0016581; GO:0019899; GO:0030336; GO:0030512; GO:0031000; GO:0031072; GO:0031507; GO:0032496; GO:0032732; GO:0032760; GO:0032922; GO:0032967; GO:0032991; GO:0033558; GO:0034605; GO:0035094; GO:0035098; GO:0042220; GO:0042393; GO:0042475; GO:0042531; GO:0042659; GO:0042733; GO:0042826; GO:0043066; GO:0043433; GO:0045347; GO:0045862; GO:0045892; GO:0045893; GO:0045944; GO:0048149; GO:0048714; GO:0051059; GO:0055093; GO:0060789; GO:0061000; GO:0061029; GO:0061198; GO:0061629; GO:0070301; GO:0071300; GO:0071560; GO:0160009; GO:0160010; GO:1902437; GO:1902455; GO:1902459; GO:1903351; GO:1990841; GO:2000273; GO:2000736; GO:2000757** |
| ENSG00000101361 | NOP56 | -0.168122759 | O00567 | NOP56_HUMAN | Nucleolar protein 56 (Nucleolar protein 5A) | NOP56 NOL5A | 594 | FUNCTION: Involved in the early to middle stages of 60S ribosomal subunit biogenesis. Core component of box C/D small nucleolar ribonucleoprotein (snoRNP) particles. Required for the biogenesis of box C/D snoRNAs such U3, U8 and U14 snoRNAs (PubMed:12777385, PubMed:15574333). Part of the small subunit (SSU) processome, first precursor of the small eukaryotic ribosomal subunit. During the assembly of the SSU processome in the nucleolus, many ribosome biogenesis factors, an RNA chaperone and ribosomal proteins associate with the nascent pre-rRNA and work in concert to generate RNA folding, modifications, rearrangements and cleavage as well as targeted degradation of pre-ribosomal RNA by the RNA exosome (PubMed:34516797). {ECO:0000269|PubMed:12777385, ECO:0000269|PubMed:15574333, ECO:0000269|PubMed:34516797}. | ribosomal small subunit biogenesis [GO:0042274]; rRNA processing [GO:0006364] | GO:0001650; GO:0003723; GO:0005654; GO:0005730; GO:0005732; GO:0005737; GO:0006364; GO:0016020; GO:0030515; GO:0031428; GO:0032040; GO:0042274; GO:0045296; GO:0070761; GO:1990226 |
| ENSG00000101911 | PRPS2 | -0.168122759 | P11908 | PRPS2_HUMAN | Ribose-phosphate pyrophosphokinase 2 (EC 2.7.6.1) (PPRibP) (Phosphoribosyl pyrophosphate synthase II) (PRS-II) | PRPS2 | 318 | FUNCTION: Catalyzes the synthesis of phosphoribosylpyrophosphate (PRPP) that is essential for nucleotide synthesis. | 5-phosphoribose 1-diphosphate biosynthetic process [GO:0006015]; nucleobase-containing compound metabolic process [GO:0006139]; pentose-phosphate shunt [GO:0006098]; phosphorylation [GO:0016310]; purine nucleotide biosynthetic process [GO:0006164]; ribonucleoside monophosphate biosynthetic process [GO:0009156] | GO:0000287; GO:0002189; GO:0004749; GO:0005524; GO:0005737; GO:0005829; GO:0006015; GO:0006098; GO:0006139; GO:0006164; GO:0009156; GO:0016301; GO:0016310; GO:0042802; GO:0042803 |
| ENSG00000106263 | EIF3B | -0.168122759 | P55884 | EIF3B_HUMAN | Eukaryotic translation initiation factor 3 subunit B (eIF3b) (Eukaryotic translation initiation factor 3 subunit 9) (Prt1 homolog) (hPrt1) (eIF-3-eta) (eIF3 p110) (eIF3 p116) | EIF3B EIF3S9 | 814 | FUNCTION: RNA-binding component of the eukaryotic translation initiation factor 3 (eIF-3) complex, which is required for several steps in the initiation of protein synthesis (PubMed:9388245, PubMed:17581632, PubMed:25849773, PubMed:27462815). The eIF-3 complex associates with the 40S ribosome and facilitates the recruitment of eIF-1, eIF-1A, eIF-2:GTP:methionyl-tRNAi and eIF-5 to form the 43S pre-initiation complex (43S PIC). The eIF-3 complex stimulates mRNA recruitment to the 43S PIC and scanning of the mRNA for AUG recognition. The eIF-3 complex is also required for disassembly and recycling of post-termination ribosomal complexes and subsequently prevents premature joining of the 40S and 60S ribosomal subunits prior to initiation (PubMed:9388245, PubMed:17581632). The eIF-3 complex specifically targets and initiates translation of a subset of mRNAs involved in cell proliferation, including cell cycling, differentiation and apoptosis, and uses different modes of RNA stem-loop binding to exert either translational activation or repression (PubMed:25849773). {ECO:0000255|HAMAP-Rule:MF_03001, ECO:0000269|PubMed:17581632, ECO:0000269|PubMed:25849773, ECO:0000269|PubMed:27462815, ECO:0000269|PubMed:9388245}.; FUNCTION: (Microbial infection) In case of FCV infection, plays a role in the ribosomal termination-reinitiation event leading to the translation of VP2 (PubMed:18056426). {ECO:0000269|PubMed:18056426}. | formation of cytoplasmic translation initiation complex [GO:0001732]; IRES-dependent viral translational initiation [GO:0075522]; regulation of translational initiation [GO:0006446]; translational initiation [GO:0006413]; viral translational termination-reinitiation [GO:0075525] | GO:0001732; GO:0003723; GO:0003743; GO:0005829; GO:0005852; GO:0006413; GO:0006446; GO:0010494; GO:0016282; GO:0031369; GO:0033290; GO:0045202; GO:0060090; GO:0070062; GO:0071541; GO:0075522; GO:0075525 |
| ENSG00000128951 | DUT | -0.168122759 | P33316 | DUT_HUMAN | Deoxyuridine 5'-triphosphate nucleotidohydrolase, mitochondrial (dUTPase) (EC 3.6.1.23) (dUTP pyrophosphatase) | DUT | 252 | FUNCTION: Catalyzes the cleavage of 2'-deoxyuridine 5'-triphosphate (dUTP) into 2'-deoxyuridine 5'-monophosphate (dUMP) and inorganic pyrophosphate and through its action efficiently prevents uracil misincorporation into DNA and at the same time provides dUMP, the substrate for de novo thymidylate biosynthesis (PubMed:17880943, PubMed:8631816, PubMed:8805593). Inhibits peroxisome proliferator-activated receptor (PPAR) activity by binding of its N-terminal to PPAR, preventing the latter's dimerization with retinoid X receptor (By similarity). Essential for embryonic development (By similarity). {ECO:0000250|UniProtKB:P70583, ECO:0000250|UniProtKB:Q9CQ43, ECO:0000269|PubMed:17880943, ECO:0000269|PubMed:8631816, ECO:0000269|PubMed:8805593}. | DNA replication [GO:0006260]; dTMP biosynthetic process [GO:0006231]; dUMP biosynthetic process [GO:0006226]; dUTP catabolic process [GO:0046081]; liver development [GO:0001889]; nucleobase-containing compound metabolic process [GO:0006139]; regulation of protein-containing complex assembly [GO:0043254]; response to organic cyclic compound [GO:0014070] | GO:0000287; GO:0001889; GO:0003723; GO:0004170; GO:0005634; GO:0005654; GO:0005739; GO:0006139; GO:0006226; GO:0006231; GO:0006260; GO:0014070; GO:0030547; GO:0032556; GO:0042802; GO:0042975; GO:0043254; GO:0046081; GO:0070062 |
| ENSG00000135316 | SYNCRIP | -0.168122759 | O60506 | HNRPQ_HUMAN | Heterogeneous nuclear ribonucleoprotein Q (hnRNP Q) (Glycine- and tyrosine-rich RNA-binding protein) (GRY-RBP) (NS1-associated protein 1) (Synaptotagmin-binding, cytoplasmic RNA-interacting protein) | SYNCRIP HNRPQ NSAP1 | 623 | FUNCTION: Heterogenous nuclear ribonucleoprotein (hnRNP) implicated in mRNA processing mechanisms. Component of the CRD-mediated complex that promotes MYC mRNA stability. Isoform 1, isoform 2 and isoform 3 are associated in vitro with pre-mRNA, splicing intermediates and mature mRNA protein complexes. Isoform 1 binds to apoB mRNA AU-rich sequences. Isoform 1 is part of the APOB mRNA editosome complex and may modulate the postranscriptional C to U RNA-editing of the APOB mRNA through either by binding to A1CF (APOBEC1 complementation factor), to APOBEC1 or to RNA itself. May be involved in translationally coupled mRNA turnover. Implicated with other RNA-binding proteins in the cytoplasmic deadenylation/translational and decay interplay of the FOS mRNA mediated by the major coding-region determinant of instability (mCRD) domain. Interacts in vitro preferentially with poly(A) and poly(U) RNA sequences. Isoform 3 may be involved in cytoplasmic vesicle-based mRNA transport through interaction with synaptotagmins. Component of the GAIT (gamma interferon-activated inhibitor of translation) complex which mediates interferon-gamma-induced transcript-selective translation inhibition in inflammation processes. Upon interferon-gamma activation assembles into the GAIT complex which binds to stem loop-containing GAIT elements in the 3'-UTR of diverse inflammatory mRNAs (such as ceruplasmin) and suppresses their translation; seems not to be essential for GAIT complex function. {ECO:0000269|PubMed:11051545, ECO:0000269|PubMed:11134005, ECO:0000269|PubMed:11352648, ECO:0000269|PubMed:11574476, ECO:0000269|PubMed:19029303, ECO:0000269|PubMed:23071094}. | cellular response to type II interferon [GO:0071346]; CRD-mediated mRNA stabilization [GO:0070934]; mRNA modification [GO:0016556]; mRNA splicing, via spliceosome [GO:0000398]; negative regulation of nuclear-transcribed mRNA catabolic process, deadenylation-dependent decay [GO:1900152]; negative regulation of nuclear-transcribed mRNA catabolic process, nonsense-mediated decay [GO:2000623]; negative regulation of translation [GO:0017148]; osteoblast differentiation [GO:0001649]; positive regulation of cytoplasmic translation [GO:2000767]; positive regulation of DNA demethylation [GO:1901537]; RNA processing [GO:0006396]; RNA splicing [GO:0008380] | GO:0000398; GO:0001649; GO:0003723; GO:0005634; GO:0005654; GO:0005783; GO:0005829; GO:0006396; GO:0008380; GO:0016020; GO:0016556; GO:0017148; GO:0045293; GO:0048027; GO:0070934; GO:0070937; GO:0071013; GO:0071204; GO:0071346; GO:0097452; GO:0106002; GO:1900152; GO:1901537; GO:1990904; GO:2000623; GO:2000767 |
| ENSG00000120438 | TCP1 | -0.184424571 | P17987 | TCPA_HUMAN | T-complex protein 1 subunit alpha (TCP-1-alpha) (CCT-alpha) | TCP1 CCT1 CCTA | 556 | FUNCTION: Component of the chaperonin-containing T-complex (TRiC), a molecular chaperone complex that assists the folding of proteins upon ATP hydrolysis (PubMed:25467444). The TRiC complex mediates the folding of WRAP53/TCAB1, thereby regulating telomere maintenance (PubMed:25467444). As part of the TRiC complex may play a role in the assembly of BBSome, a complex involved in ciliogenesis regulating transports vesicles to the cilia (PubMed:20080638). The TRiC complex plays a role in the folding of actin and tubulin (Probable). {ECO:0000269|PubMed:20080638, ECO:0000269|PubMed:25467444, ECO:0000305}. | binding of sperm to zona pellucida [GO:0007339]; chaperone-mediated protein folding [GO:0061077]; positive regulation of establishment of protein localization to telomere [GO:1904851]; positive regulation of protein localization to Cajal body [GO:1904871]; positive regulation of telomerase activity [GO:0051973]; positive regulation of telomerase RNA localization to Cajal body [GO:1904874]; positive regulation of telomere maintenance via telomerase [GO:0032212]; protein folding [GO:0006457]; protein stabilization [GO:0050821]; scaRNA localization to Cajal body [GO:0090666]; tubulin complex assembly [GO:0007021] | GO:0000242; GO:0000792; GO:0001669; GO:0002199; GO:0003723; GO:0005524; GO:0005794; GO:0005813; GO:0005829; GO:0005832; GO:0005874; GO:0006457; GO:0007021; GO:0007339; GO:0016887; GO:0031625; GO:0032212; GO:0044183; GO:0044297; GO:0050821; GO:0051082; GO:0051973; GO:0061077; GO:0070062; GO:0090666; GO:0140662; GO:1904851; GO:1904871; GO:1904874 |
| ENSG00000136527 | TRA2B | -0.184424571 | P62995 | TRA2B_HUMAN | Transformer-2 protein homolog beta (TRA-2 beta) (TRA2-beta) (hTRA2-beta) (Splicing factor, arginine/serine-rich 10) (Transformer-2 protein homolog B) | TRA2B SFRS10 | 288 | FUNCTION: Sequence-specific RNA-binding protein which participates in the control of pre-mRNA splicing. Can either activate or suppress exon inclusion. Acts additively with RBMX to promote exon 7 inclusion of the survival motor neuron SMN2. Activates the splicing of MAPT/Tau exon 10. Alters pre-mRNA splicing patterns by antagonizing the effects of splicing regulators, like RBMX. Binds to the AG-rich SE2 domain in the SMN exon 7 RNA. Binds to pre-mRNA. {ECO:0000269|PubMed:12165565, ECO:0000269|PubMed:12761049, ECO:0000269|PubMed:15009664, ECO:0000269|PubMed:9546399}. | cellular response to glucose stimulus [GO:0071333]; cerebral cortex regionalization [GO:0021796]; embryonic brain development [GO:1990403]; mRNA splicing, via spliceosome [GO:0000398]; positive regulation of mRNA splicing, via spliceosome [GO:0048026]; regulation of alternative mRNA splicing, via spliceosome [GO:0000381]; regulation of RNA splicing [GO:0043484]; RNA splicing, via transesterification reactions [GO:0000375] | GO:0000375; GO:0000381; GO:0000398; GO:0003723; GO:0003729; GO:0005634; GO:0005637; GO:0005654; GO:0005681; GO:0019904; GO:0021796; GO:0032991; GO:0036002; GO:0042802; GO:0043484; GO:0048026; GO:0071333; GO:1990403 |
| **ENSG00000089009** | **RPL6** | **-0.200912694** | **Q02878** | **RL6_HUMAN** | **Large ribosomal subunit protein eL6 (60S ribosomal protein L6) (Neoplasm-related protein C140) (Tax-responsive enhancer element-binding protein 107) (TaxREB107)** | **RPL6 TXREB1** | **288** | **FUNCTION: Component of the large ribosomal subunit (PubMed:12962325, PubMed:25957688, PubMed:25901680, PubMed:32669547, PubMed:23636399). The ribosome is a large ribonucleoprotein complex responsible for the synthesis of proteins in the cell (PubMed:12962325, PubMed:25957688, PubMed:25901680, PubMed:32669547, PubMed:23636399). {ECO:0000269|PubMed:23636399, ECO:0000269|PubMed:25901680, ECO:0000269|PubMed:25957688, ECO:0000269|PubMed:32669547, ECO:0000305|PubMed:12962325}.; FUNCTION: (Microbial infection) Specifically binds to domain C of the Tax-responsive enhancer element in the long terminal repeat of HTLV-I (PubMed:8457378). {ECO:0000269|PubMed:8457378}.** | **cytoplasmic translation [GO:0002181]; regulation of DNA-templated transcription [GO:0006355]; ribosomal large subunit assembly [GO:0000027]; translation [GO:0006412]** | **GO:0000027; GO:0002181; GO:0003677; GO:0003723; GO:0003735; GO:0005634; GO:0005737; GO:0005791; GO:0005829; GO:0005925; GO:0006355; GO:0006412; GO:0014069; GO:0016020; GO:0022625; GO:0022626; GO:0036464; GO:0042788; GO:0045296** |
| ENSG00000121022 | COPS5 | -0.200912694 | Q92905 | CSN5_HUMAN | COP9 signalosome complex subunit 5 (SGN5) (Signalosome subunit 5) (EC 3.4.-.-) (Jun activation domain-binding protein 1) | COPS5 CSN5 JAB1 | 334 | FUNCTION: Probable protease subunit of the COP9 signalosome complex (CSN), a complex involved in various cellular and developmental processes. The CSN complex is an essential regulator of the ubiquitin (Ubl) conjugation pathway by mediating the deneddylation of the cullin subunits of the SCF-type E3 ligase complexes, leading to decrease the Ubl ligase activity of SCF-type complexes such as SCF, CSA or DDB2. The complex is also involved in phosphorylation of p53/TP53, c-jun/JUN, IkappaBalpha/NFKBIA, ITPK1 and IRF8, possibly via its association with CK2 and PKD kinases. CSN-dependent phosphorylation of TP53 and JUN promotes and protects degradation by the Ubl system, respectively. In the complex, it probably acts as the catalytic center that mediates the cleavage of Nedd8 from cullins. It however has no metalloprotease activity by itself and requires the other subunits of the CSN complex. Interacts directly with a large number of proteins that are regulated by the CSN complex, confirming a key role in the complex. Promotes the proteasomal degradation of BRSK2. {ECO:0000269|PubMed:11285227, ECO:0000269|PubMed:11337588, ECO:0000269|PubMed:12628923, ECO:0000269|PubMed:12732143, ECO:0000269|PubMed:19214193, ECO:0000269|PubMed:20978819, ECO:0000269|PubMed:22609399, ECO:0000269|PubMed:9535219}. | exosomal secretion [GO:1990182]; negative regulation of apoptotic process [GO:0043066]; positive regulation of DNA-binding transcription factor activity [GO:0051091]; positive regulation of transcription by RNA polymerase II [GO:0045944]; post-translational protein modification [GO:0043687]; protein deneddylation [GO:0000338]; protein deubiquitination [GO:0016579]; protein neddylation [GO:0045116]; regulation of cell cycle [GO:0051726]; regulation of IRE1-mediated unfolded protein response [GO:1903894]; regulation of JNK cascade [GO:0046328]; regulation of protein neddylation [GO:2000434]; translation [GO:0006412] | GO:0000338; GO:0003713; GO:0003743; GO:0005634; GO:0005654; GO:0005737; GO:0005829; GO:0005852; GO:0006412; GO:0008021; GO:0008180; GO:0008237; GO:0016579; GO:0019784; GO:0019899; GO:0035718; GO:0043066; GO:0043687; GO:0045116; GO:0045944; GO:0046328; GO:0046872; GO:0048471; GO:0051091; GO:0051726; GO:0140492; GO:1903894; GO:1990182; GO:2000434 |
| ENSG00000165672 | PRDX3 | -0.200912694 | P30048 | PRDX3_HUMAN | Thioredoxin-dependent peroxide reductase, mitochondrial (EC 1.11.1.24) (Antioxidant protein 1) (AOP-1) (HBC189) (Peroxiredoxin III) (Prx-III) (Peroxiredoxin-3) (Protein MER5 homolog) (Thioredoxin-dependent peroxiredoxin 3) | PRDX3 AOP1 | 256 | FUNCTION: Thiol-specific peroxidase that catalyzes the reduction of hydrogen peroxide and organic hydroperoxides to water and alcohols, respectively. Plays a role in cell protection against oxidative stress by detoxifying peroxides (PubMed:7733872, PubMed:17707404, PubMed:29438714, PubMed:33889951). Acts synergistically with MAP3K13 to regulate the activation of NF-kappa-B in the cytosol (PubMed:12492477). Required for the maintenance of physical strength (By similarity). {ECO:0000250|UniProtKB:P20108, ECO:0000269|PubMed:12492477, ECO:0000269|PubMed:17707404, ECO:0000269|PubMed:29438714, ECO:0000269|PubMed:33889951, ECO:0000269|PubMed:7733872}. | cell redox homeostasis [GO:0045454]; cellular response to oxidative stress [GO:0034599]; cellular response to reactive oxygen species [GO:0034614]; hydrogen peroxide catabolic process [GO:0042744]; maternal placenta development [GO:0001893]; mitochondrion organization [GO:0007005]; myeloid cell differentiation [GO:0030099]; negative regulation of apoptotic process [GO:0043066]; negative regulation of kinase activity [GO:0033673]; peptidyl-cysteine oxidation [GO:0018171]; positive regulation of cell population proliferation [GO:0008284]; positive regulation of NF-kappaB transcription factor activity [GO:0051092]; regulation of mitochondrial membrane potential [GO:0051881]; response to hydrogen peroxide [GO:0042542]; response to lipopolysaccharide [GO:0032496]; response to oxidative stress [GO:0006979] | GO:0001893; GO:0005654; GO:0005737; GO:0005739; GO:0005759; GO:0005769; GO:0005829; GO:0005886; GO:0006979; GO:0007005; GO:0008284; GO:0008379; GO:0008785; GO:0018171; GO:0019901; GO:0030099; GO:0032496; GO:0032991; GO:0033673; GO:0034599; GO:0034614; GO:0042542; GO:0042744; GO:0042802; GO:0043027; GO:0043066; GO:0043231; GO:0045454; GO:0051092; GO:0051881 |
| ENSG00000096063 | SRPK1 | -0.217591435 | Q96SB4 | SRPK1_HUMAN | SRSF protein kinase 1 (EC 2.7.11.1) (SFRS protein kinase 1) (Serine/arginine-rich protein-specific kinase 1) (SR-protein-specific kinase 1) | SRPK1 | 655 | FUNCTION: Serine/arginine-rich protein-specific kinase which specifically phosphorylates its substrates at serine residues located in regions rich in arginine/serine dipeptides, known as RS domains and is involved in the phosphorylation of SR splicing factors and the regulation of splicing. Plays a central role in the regulatory network for splicing, controlling the intranuclear distribution of splicing factors in interphase cells and the reorganization of nuclear speckles during mitosis. Can influence additional steps of mRNA maturation, as well as other cellular activities, such as chromatin reorganization in somatic and sperm cells and cell cycle progression. Isoform 2 phosphorylates SFRS2, ZRSR2, LBR and PRM1. Isoform 2 phosphorylates SRSF1 using a directional (C-terminal to N-terminal) and a dual-track mechanism incorporating both processive phosphorylation (in which the kinase stays attached to the substrate after each round of phosphorylation) and distributive phosphorylation steps (in which the kinase and substrate dissociate after each phosphorylation event). The RS domain of SRSF1 binds first to a docking groove in the large lobe of the kinase domain of SRPK1. This induces certain structural changes in SRPK1 and/or RRM2 domain of SRSF1, allowing RRM2 to bind the kinase and initiate phosphorylation. The cycles continue for several phosphorylation steps in a processive manner (steps 1-8) until the last few phosphorylation steps (approximately steps 9-12). During that time, a mechanical stress induces the unfolding of the beta-4 motif in RRM2, which then docks at the docking groove of SRPK1. This also signals RRM2 to begin to dissociate, which facilitates SRSF1 dissociation after phosphorylation is completed. Isoform 2 can mediate hepatitis B virus (HBV) core protein phosphorylation. It plays a negative role in the regulation of HBV replication through a mechanism not involving the phosphorylation of the core protein but by reducing the packaging efficiency of the pregenomic RNA (pgRNA) without affecting the formation of the viral core particles. Isoform 1 and isoform 2 can induce splicing of exon 10 in MAPT/TAU. The ratio of isoform 1/isoform 2 plays a decisive role in determining cell fate in K-562 leukaemic cell line: isoform 2 favors proliferation where as isoform 1 favors differentiation. {ECO:0000269|PubMed:10049757, ECO:0000269|PubMed:10390541, ECO:0000269|PubMed:11509566, ECO:0000269|PubMed:12134018, ECO:0000269|PubMed:14555757, ECO:0000269|PubMed:15034300, ECO:0000269|PubMed:16122776, ECO:0000269|PubMed:16209947, ECO:0000269|PubMed:18155240, ECO:0000269|PubMed:18687337, ECO:0000269|PubMed:19240134, ECO:0000269|PubMed:19477182, ECO:0000269|PubMed:19886675, ECO:0000269|PubMed:20708644, ECO:0000269|PubMed:8208298, ECO:0000269|PubMed:9237760}. | chromosome segregation [GO:0007059]; innate immune response [GO:0045087]; intracellular signal transduction [GO:0035556]; negative regulation of viral genome replication [GO:0045071]; positive regulation of viral genome replication [GO:0045070]; protein phosphorylation [GO:0006468]; regulation of mRNA processing [GO:0050684]; regulation of mRNA splicing, via spliceosome [GO:0048024]; RNA splicing [GO:0008380]; sperm DNA condensation [GO:0035092]; spliceosomal complex assembly [GO:0000245] | GO:0000245; GO:0000287; GO:0000785; GO:0003723; GO:0004672; GO:0004674; GO:0005524; GO:0005634; GO:0005654; GO:0005737; GO:0005783; GO:0005829; GO:0005886; GO:0006468; GO:0007059; GO:0008380; GO:0016363; GO:0016607; GO:0035092; GO:0035556; GO:0045070; GO:0045071; GO:0045087; GO:0048024; GO:0050684; GO:0106310 |
| ENSG00000196419 | XRCC6 | -0.234465254 | P12956 | XRCC6_HUMAN | X-ray repair cross-complementing protein 6 (EC 3.6.4.-) (EC 4.2.99.-) (5'-deoxyribose-5-phosphate lyase Ku70) (5'-dRP lyase Ku70) (70 kDa subunit of Ku antigen) (ATP-dependent DNA helicase 2 subunit 1) (ATP-dependent DNA helicase II 70 kDa subunit) (CTC box-binding factor 75 kDa subunit) (CTC75) (CTCBF) (DNA repair protein XRCC6) (Lupus Ku autoantigen protein p70) (Ku70) (Thyroid-lupus autoantigen) (TLAA) (X-ray repair complementing defective repair in Chinese hamster cells 6) | XRCC6 G22P1 | 609 | FUNCTION: Single-stranded DNA-dependent ATP-dependent helicase that plays a key role in DNA non-homologous end joining (NHEJ) by recruiting DNA-PK to DNA (PubMed:7957065, PubMed:8621488, PubMed:12145306, PubMed:11493912, PubMed:20493174, PubMed:2466842, PubMed:9742108). Required for double-strand break repair and V(D)J recombination (PubMed:7957065, PubMed:8621488, PubMed:12145306, PubMed:11493912, PubMed:20493174, PubMed:2466842, PubMed:9742108). Also has a role in chromosome translocation (PubMed:7957065, PubMed:8621488, PubMed:12145306, PubMed:11493912, PubMed:20493174, PubMed:2466842, PubMed:9742108). Has a role in chromosome translocation (PubMed:7957065, PubMed:20493174, PubMed:2466842, PubMed:9742108, PubMed:8621488, PubMed:12145306, PubMed:11493912). The DNA helicase II complex binds preferentially to fork-like ends of double-stranded DNA in a cell cycle-dependent manner (PubMed:7957065, PubMed:8621488, PubMed:20493174, PubMed:2466842, PubMed:9742108, PubMed:12145306, PubMed:11493912). It works in the 3'-5' direction (PubMed:20493174, PubMed:2466842, PubMed:9742108, PubMed:7957065, PubMed:8621488, PubMed:12145306, PubMed:11493912). During NHEJ, the XRCC5-XRRC6 dimer performs the recognition step: it recognizes and binds to the broken ends of the DNA and protects them from further resection (PubMed:7957065, PubMed:8621488, PubMed:20493174, PubMed:2466842, PubMed:9742108, PubMed:12145306, PubMed:11493912). Binding to DNA may be mediated by XRCC6 (PubMed:20493174, PubMed:2466842, PubMed:9742108, PubMed:7957065, PubMed:8621488, PubMed:12145306, PubMed:11493912). The XRCC5-XRRC6 dimer acts as regulatory subunit of the DNA-dependent protein kinase complex DNA-PK by increasing the affinity of the catalytic subunit PRKDC to DNA by 100-fold (PubMed:7957065, PubMed:8621488, PubMed:12145306, PubMed:11493912, PubMed:20493174, PubMed:2466842, PubMed:9742108). The XRCC5-XRRC6 dimer is probably involved in stabilizing broken DNA ends and bringing them together (PubMed:7957065, PubMed:8621488, PubMed:12145306, PubMed:11493912, PubMed:20493174, PubMed:2466842, PubMed:9742108). The assembly of the DNA-PK complex to DNA ends is required for the NHEJ ligation step (PubMed:7957065, PubMed:8621488, PubMed:12145306, PubMed:11493912, PubMed:20493174, PubMed:2466842, PubMed:9742108). Probably also acts as a 5'-deoxyribose-5-phosphate lyase (5'-dRP lyase), by catalyzing the beta-elimination of the 5' deoxyribose-5-phosphate at an abasic site near double-strand breaks (PubMed:20383123). 5'-dRP lyase activity allows to 'clean' the termini of abasic sites, a class of nucleotide damage commonly associated with strand breaks, before such broken ends can be joined (PubMed:20383123). The XRCC5-XRRC6 dimer together with APEX1 acts as a negative regulator of transcription (PubMed:8621488). In association with NAA15, the XRCC5-XRRC6 dimer binds to the osteocalcin promoter and activates osteocalcin expression (PubMed:12145306). Plays a role in the regulation of DNA virus-mediated innate immune response by assembling into the HDP-RNP complex, a complex that serves as a platform for IRF3 phosphorylation and subsequent innate immune response activation through the cGAS-STING pathway (PubMed:28712728). {ECO:0000269|PubMed:11493912, ECO:0000269|PubMed:12145306, ECO:0000269|PubMed:20383123, ECO:0000269|PubMed:20493174, ECO:0000269|PubMed:2466842, ECO:0000269|PubMed:28712728, ECO:0000269|PubMed:7957065, ECO:0000269|PubMed:8621488, ECO:0000269|PubMed:9742108}. | activation of innate immune response [GO:0002218]; cellular hyperosmotic salinity response [GO:0071475]; cellular response to gamma radiation [GO:0071480]; cellular response to X-ray [GO:0071481]; DNA ligation [GO:0006266]; double-strand break repair via classical nonhomologous end joining [GO:0097680]; double-strand break repair via nonhomologous end joining [GO:0006303]; innate immune response [GO:0045087]; negative regulation of DNA-templated transcription [GO:0045892]; positive regulation of DNA-templated transcription [GO:0045893]; positive regulation of lymphocyte differentiation [GO:0045621]; positive regulation of protein kinase activity [GO:0045860]; positive regulation of transcription by RNA polymerase II [GO:0045944]; recombinational repair [GO:0000725]; regulation of smooth muscle cell proliferation [GO:0048660]; telomere maintenance [GO:0000723] | GO:0000723; GO:0000725; GO:0000781; GO:0000783; GO:0000976; GO:0002218; GO:0003677; GO:0003678; GO:0003684; GO:0003690; GO:0003723; GO:0005524; GO:0005576; GO:0005634; GO:0005654; GO:0005667; GO:0005730; GO:0005829; GO:0006266; GO:0006303; GO:0008094; GO:0016020; GO:0016887; GO:0030332; GO:0032991; GO:0032993; GO:0034774; GO:0042162; GO:0043564; GO:0044877; GO:0045087; GO:0045621; GO:0045860; GO:0045892; GO:0045893; GO:0045944; GO:0048660; GO:0051575; GO:0070418; GO:0070419; GO:0071475; GO:0071480; GO:0071481; GO:0097110; GO:0097680; GO:1904813 |
| ENSG00000204568 | MRPS18B | -0.234465254 | Q9Y676 | RT18B_HUMAN | Small ribosomal subunit protein mS40 (28S ribosomal protein S18-2, mitochondrial) (MRP-S18-2) (28S ribosomal protein S18b, mitochondrial) (MRP-S18-b) (Mrps18-b) (S18mt-b) (Small ribosomal subunit protein bS18b) | MRPS18B C6orf14 HSPC183 PTD017 | 258 |  | mitochondrial translation [GO:0032543]; translation [GO:0006412] | GO:0003735; GO:0005654; GO:0005739; GO:0005743; GO:0005763; GO:0006412; GO:0030054; GO:0032543 |
| ENSG00000104131 | EIF3J | -0.251538767 | O75822 | EIF3J_HUMAN | Eukaryotic translation initiation factor 3 subunit J (eIF3j) (Eukaryotic translation initiation factor 3 subunit 1) (eIF-3-alpha) (eIF3 p35) | EIF3J EIF3S1 PRO0391 | 258 | FUNCTION: Component of the eukaryotic translation initiation factor 3 (eIF-3) complex, which is required for several steps in the initiation of protein synthesis (PubMed:25849773, PubMed:27462815). The eIF-3 complex associates with the 40S ribosome and facilitates the recruitment of eIF-1, eIF-1A, eIF-2:GTP:methionyl-tRNAi and eIF-5 to form the 43S pre-initiation complex (43S PIC). The eIF-3 complex stimulates mRNA recruitment to the 43S PIC and scanning of the mRNA for AUG recognition. The eIF-3 complex is also required for disassembly and recycling of post-termination ribosomal complexes and subsequently prevents premature joining of the 40S and 60S ribosomal subunits prior to initiation. The eIF-3 complex specifically targets and initiates translation of a subset of mRNAs involved in cell proliferation, including cell cycling, differentiation and apoptosis, and uses different modes of RNA stem-loop binding to exert either translational activation or repression (PubMed:25849773). {ECO:0000269|PubMed:25849773, ECO:0000269|PubMed:27462815}. | formation of cytoplasmic translation initiation complex [GO:0001732]; translational initiation [GO:0006413] | GO:0001732; GO:0003743; GO:0005829; GO:0005852; GO:0006413; GO:0016282; GO:0033290; GO:0042802 |
| ENSG00000108424 | KPNB1 | -0.251538767 | Q14974 | IMB1_HUMAN | Importin subunit beta-1 (Importin-90) (Karyopherin subunit beta-1) (Nuclear factor p97) (Pore targeting complex 97 kDa subunit) (PTAC97) | KPNB1 NTF97 | 876 | FUNCTION: Functions in nuclear protein import, either in association with an adapter protein, like an importin-alpha subunit, which binds to nuclear localization signals (NLS) in cargo substrates, or by acting as autonomous nuclear transport receptor. Acting autonomously, serves itself as NLS receptor. Docking of the importin/substrate complex to the nuclear pore complex (NPC) is mediated by KPNB1 through binding to nucleoporin FxFG repeats and the complex is subsequently translocated through the pore by an energy requiring, Ran-dependent mechanism. At the nucleoplasmic side of the NPC, Ran binds to importin-beta and the three components separate and importin-alpha and -beta are re-exported from the nucleus to the cytoplasm where GTP hydrolysis releases Ran from importin. The directionality of nuclear import is thought to be conferred by an asymmetric distribution of the GTP- and GDP-bound forms of Ran between the cytoplasm and nucleus. Mediates autonomously the nuclear import of ribosomal proteins RPL23A, RPS7 and RPL5 (PubMed:11682607). In association with IPO7, mediates the nuclear import of H1 histone. In vitro, mediates nuclear import of H2A, H2B, H3 and H4 histones. In case of HIV-1 infection, binds and mediates the nuclear import of HIV-1 Rev. Imports SNAI1 and PRKCI into the nucleus. {ECO:0000269|PubMed:10228156, ECO:0000269|PubMed:11682607, ECO:0000269|PubMed:11891849, ECO:0000269|PubMed:19386897, ECO:0000269|PubMed:24699649, ECO:0000269|PubMed:9687515}. | astral microtubule organization [GO:0030953]; establishment of mitotic spindle localization [GO:0040001]; establishment of protein localization [GO:0045184]; mitotic chromosome movement towards spindle pole [GO:0007079]; mitotic metaphase chromosome alignment [GO:0007080]; mitotic spindle assembly [GO:0090307]; NLS-bearing protein import into nucleus [GO:0006607]; protein import into nucleus [GO:0006606]; Ran protein signal transduction [GO:0031291]; ribosomal protein import into nucleus [GO:0006610]; RNA import into nucleus [GO:0006404] | GO:0003723; GO:0005576; GO:0005634; GO:0005635; GO:0005643; GO:0005654; GO:0005737; GO:0005829; GO:0006404; GO:0006606; GO:0006607; GO:0006610; GO:0007079; GO:0007080; GO:0008139; GO:0008270; GO:0010494; GO:0016020; GO:0019899; GO:0019904; GO:0030953; GO:0031267; GO:0031291; GO:0031965; GO:0035580; GO:0040001; GO:0042564; GO:0045184; GO:0051879; GO:0061608; GO:0061676; GO:0070062; GO:0071782; GO:0090307; GO:1904813 |
| ENSG00000105202 | FBL | -0.304006187 | P22087 | FBRL_HUMAN | rRNA 2'-O-methyltransferase fibrillarin (EC 2.1.1.-) (34 kDa nucleolar scleroderma antigen) (Histone-glutamine methyltransferase) (U6 snRNA 2'-O-methyltransferase fibrillarin) | FBL FIB1 FLRN | 321 | FUNCTION: S-adenosyl-L-methionine-dependent methyltransferase that has the ability to methylate both RNAs and proteins (PubMed:24352239, PubMed:30540930, PubMed:32017898). Involved in pre-rRNA processing by catalyzing the site-specific 2'-hydroxyl methylation of ribose moieties in pre-ribosomal RNA (PubMed:30540930). Site specificity is provided by a guide RNA that base pairs with the substrate (By similarity). Methylation occurs at a characteristic distance from the sequence involved in base pairing with the guide RNA (By similarity). Probably catalyzes 2'-O-methylation of U6 snRNAs in box C/D RNP complexes (PubMed:32017898). U6 snRNA 2'-O-methylation is required for mRNA splicing fidelity (PubMed:32017898). Also acts as a protein methyltransferase by mediating methylation of 'Gln-105' of histone H2A (H2AQ104me), a modification that impairs binding of the FACT complex and is specifically present at 35S ribosomal DNA locus (PubMed:24352239, PubMed:30540930). Part of the small subunit (SSU) processome, first precursor of the small eukaryotic ribosomal subunit. During the assembly of the SSU processome in the nucleolus, many ribosome biogenesis factors, an RNA chaperone and ribosomal proteins associate with the nascent pre-rRNA and work in concert to generate RNA folding, modifications, rearrangements and cleavage as well as targeted degradation of pre-ribosomal RNA by the RNA exosome (PubMed:34516797). {ECO:0000250|UniProtKB:P15646, ECO:0000269|PubMed:24352239, ECO:0000269|PubMed:30540930, ECO:0000269|PubMed:32017898, ECO:0000269|PubMed:34516797}. | box C/D RNA 3'-end processing [GO:0000494]; osteoblast differentiation [GO:0001649]; ribosomal small subunit biogenesis [GO:0042274]; rRNA methylation [GO:0031167]; rRNA processing [GO:0006364]; snoRNA localization [GO:0048254] | GO:0000494; GO:0001094; GO:0001649; GO:0001650; GO:0001652; GO:0003723; GO:0005634; GO:0005654; GO:0005730; GO:0006364; GO:0008649; GO:0015030; GO:0016020; GO:0031167; GO:0031428; GO:0032040; GO:0042274; GO:0048254; GO:0051117; GO:0070062; GO:1990259 |
| ENSG00000112081 | SRSF3 | -0.304006187 | P84103 | SRSF3_HUMAN | Serine/arginine-rich splicing factor 3 (Pre-mRNA-splicing factor SRP20) (Splicing factor, arginine/serine-rich 3) | SRSF3 SFRS3 SRP20 | 164 | FUNCTION: Splicing factor that specifically promotes exon-inclusion during alternative splicing (PubMed:26876937). Interaction with YTHDC1, a RNA-binding protein that recognizes and binds N6-methyladenosine (m6A)-containing RNAs, promotes recruitment of SRSF3 to its mRNA-binding elements adjacent to m6A sites, leading to exon-inclusion during alternative splicing (PubMed:26876937). Also functions as export adapter involved in mRNA nuclear export (PubMed:11336712, PubMed:18364396, PubMed:28984244). Binds mRNA which is thought to be transferred to the NXF1-NXT1 heterodimer for export (TAP/NXF1 pathway); enhances NXF1-NXT1 RNA-binding activity (PubMed:11336712, PubMed:18364396). Involved in nuclear export of m6A-containing mRNAs via interaction with YTHDC1: interaction with YTHDC1 facilitates m6A-containing mRNA-binding to both SRSF3 and NXF1, promoting mRNA nuclear export (PubMed:28984244). RNA-binding is semi-sequence specific (PubMed:17036044). {ECO:0000269|PubMed:11336712, ECO:0000269|PubMed:17036044, ECO:0000269|PubMed:18364396, ECO:0000269|PubMed:26876937, ECO:0000269|PubMed:28984244}. | cellular response to leukemia inhibitory factor [GO:1990830]; mRNA export from nucleus [GO:0006406]; mRNA splicing, via spliceosome [GO:0000398]; primary miRNA processing [GO:0031053]; regulation of mRNA splicing, via spliceosome [GO:0048024] | GO:0000398; GO:0003723; GO:0005654; GO:0005737; GO:0006406; GO:0016607; GO:0031053; GO:0043274; GO:0048024; GO:0070878; GO:1990830 |
| ENSG00000145907 | G3BP1 | -0.321928095 | Q13283 | G3BP1_HUMAN | Ras GTPase-activating protein-binding protein 1 (G3BP-1) (EC 3.6.4.12) (EC 3.6.4.13) (ATP-dependent DNA helicase VIII) (hDH VIII) (GAP SH3 domain-binding protein 1) | G3BP1 G3BP | 466 | FUNCTION: Protein involved in various processes, such as stress granule formation and innate immunity (PubMed:12642610, PubMed:20180778, PubMed:23279204, PubMed:30510222, PubMed:30804210). Plays an essential role in stress granule formation (PubMed:12642610, PubMed:20180778, PubMed:23279204, PubMed:32302570, PubMed:32302571, PubMed:32302572, PubMed:36183834, PubMed:36279435, PubMed:34739333, PubMed:36692217, PubMed:37379838). Stress granules are membraneless compartments that store mRNAs and proteins, such as stalled translation pre-initiation complexes, in response to stress (PubMed:12642610, PubMed:20180778, PubMed:23279204, PubMed:27022092, PubMed:32302570, PubMed:32302571, PubMed:32302572, PubMed:36279435, PubMed:37379838). Promotes formation of stress granules phase-separated membraneless compartment by undergoing liquid-liquid phase separation (LLPS) upon unfolded RNA-binding: functions as a molecular switch that triggers RNA-dependent LLPS in response to a rise in intracellular free RNA concentrations (PubMed:32302570, PubMed:32302571, PubMed:32302572, PubMed:34739333, PubMed:36692217, PubMed:36279435). Also acts as an ATP- and magnesium-dependent helicase: unwinds DNA/DNA, RNA/DNA, and RNA/RNA substrates with comparable efficiency (PubMed:9889278). Acts unidirectionally by moving in the 5' to 3' direction along the bound single-stranded DNA (PubMed:9889278). Unwinds preferentially partial DNA and RNA duplexes having a 17 bp annealed portion and either a hanging 3' tail or hanging tails at both 5'- and 3'-ends (PubMed:9889278). Plays an essential role in innate immunity by promoting CGAS and RIGI activity (PubMed:30510222, PubMed:30804210). Participates in the DNA-triggered cGAS/STING pathway by promoting the DNA binding and activation of CGAS (PubMed:30510222). Triggers the condensation of cGAS, a process probably linked to the formation of membrane-less organelles (PubMed:34779554). Enhances also RIGI-induced type I interferon production probably by helping RIGI at sensing pathogenic RNA (PubMed:30804210). May also act as a phosphorylation-dependent sequence-specific endoribonuclease in vitro: Cleaves exclusively between cytosine and adenine and cleaves MYC mRNA preferentially at the 3'-UTR (PubMed:11604510). {ECO:0000269|PubMed:11604510, ECO:0000269|PubMed:12642610, ECO:0000269|PubMed:20180778, ECO:0000269|PubMed:23279204, ECO:0000269|PubMed:27022092, ECO:0000269|PubMed:30510222, ECO:0000269|PubMed:30804210, ECO:0000269|PubMed:32302570, ECO:0000269|PubMed:32302571, ECO:0000269|PubMed:32302572, ECO:0000269|PubMed:34739333, ECO:0000269|PubMed:34779554, ECO:0000269|PubMed:36183834, ECO:0000269|PubMed:36279435, ECO:0000269|PubMed:36692217, ECO:0000269|PubMed:37379838, ECO:0000269|PubMed:9889278}. | defense response to virus [GO:0051607]; innate immune response [GO:0045087]; negative regulation of canonical Wnt signaling pathway [GO:0090090]; positive regulation of stress granule assembly [GO:0062029]; positive regulation of type I interferon production [GO:0032481]; Ras protein signal transduction [GO:0007265]; stress granule assembly [GO:0034063] | GO:0003677; GO:0003678; GO:0003723; GO:0003724; GO:0003729; GO:0004519; GO:0005524; GO:0005634; GO:0005737; GO:0005829; GO:0005925; GO:0007265; GO:0010494; GO:0016887; GO:0032481; GO:0033677; GO:0034063; GO:0043024; GO:0043204; GO:0045087; GO:0051607; GO:0062029; GO:0090090; GO:0140693; GO:1990904 |
| ENSG00000187109 | NAP1L1 | -0.340075442 | P55209 | NP1L1_HUMAN | Nucleosome assembly protein 1-like 1 (NAP-1-related protein) (hNRP) | NAP1L1 NRP | 391 | FUNCTION: Histone chaperone that plays a role in the nuclear import of H2A-H2B and nucleosome assembly (PubMed:20002496, PubMed:21211722, PubMed:26841755). Participates also in several important DNA repair mechanisms: greatly enhances ERCC6-mediated chromatin remodeling which is essential for transcription-coupled nucleotide excision DNA repair (PubMed:28369616). Stimulates also homologous recombination (HR) by RAD51 and RAD54 which is essential in mitotic DNA double strand break (DSB) repair (PubMed:24798879). Plays a key role in the regulation of embryonic neurogenesis (By similarity). Promotes the proliferation of neural progenitors and inhibits neuronal differentiation during cortical development (By similarity). Regulates neurogenesis via the modulation of RASSF10; regulates RASSF10 expression by promoting SETD1A-mediated H3K4 methylation at the RASSF10 promoter (By similarity). {ECO:0000250|UniProtKB:P28656, ECO:0000269|PubMed:20002496, ECO:0000269|PubMed:21211722, ECO:0000269|PubMed:24798879, ECO:0000269|PubMed:26841755, ECO:0000269|PubMed:28369616}.; FUNCTION: (Microbial infection) Positively regulates Epstein-Barr virus reactivation in epithelial cells through the induction of viral BZLF1 expression. {ECO:0000269|PubMed:23691099}.; FUNCTION: (Microbial infection) Together with human herpesvirus 8 protein LANA1, assists the proper assembly of the nucleosome on the replicated viral DNA. {ECO:0000269|PubMed:27599637}. | DNA replication [GO:0006260]; nervous system development [GO:0007399]; nucleosome assembly [GO:0006334]; positive regulation of cell population proliferation [GO:0008284]; positive regulation of neural precursor cell proliferation [GO:2000179]; positive regulation of neurogenesis [GO:0050769] | GO:0000785; GO:0003682; GO:0003723; GO:0005634; GO:0005737; GO:0006260; GO:0006334; GO:0007399; GO:0008284; GO:0016020; GO:0042393; GO:0042470; GO:0050769; GO:0140713; GO:2000179 |
| ENSG00000143321 | HDGF | -0.415037499 | P51858 | HDGF_HUMAN | Hepatoma-derived growth factor (HDGF) (High mobility group protein 1-like 2) (HMG-1L2) | HDGF HMG1L2 | 240 | FUNCTION: [Isoform 1]: Acts as a transcriptional repressor (PubMed:17974029). Has mitogenic activity for fibroblasts (PubMed:11751870, PubMed:26845719). Heparin-binding protein (PubMed:15491618). {ECO:0000269|PubMed:11751870, ECO:0000269|PubMed:15491618, ECO:0000269|PubMed:17974029, ECO:0000269|PubMed:26845719}.; FUNCTION: [Isoform 2]: Does not have mitogenic activity for fibroblasts (PubMed:26845719). Does not bind heparin (PubMed:26845719). {ECO:0000269|PubMed:26845719}.; FUNCTION: [Isoform 3]: Has mitogenic activity for fibroblasts (PubMed:26845719). Heparin-binding protein (PubMed:26845719). {ECO:0000269|PubMed:26845719}. | chromatin remodeling [GO:0006338]; negative regulation of transcription by RNA polymerase II [GO:0000122]; positive regulation of cell division [GO:0051781]; positive regulation of transcription by RNA polymerase II [GO:0045944]; protein localization to nucleus [GO:0034504]; signal transduction [GO:0007165] | GO:0000122; GO:0000166; GO:0000978; GO:0001222; GO:0001227; GO:0003714; GO:0003723; GO:0003779; GO:0005576; GO:0005615; GO:0005634; GO:0005654; GO:0005737; GO:0006338; GO:0007165; GO:0008083; GO:0008201; GO:0015631; GO:0017053; GO:0034504; GO:0045944; GO:0051781; GO:0062023 |
| **ENSG00000103342** | **GSPT1** | **-0.556393349** | **P15170** | **ERF3A_HUMAN** | **Eukaryotic peptide chain release factor GTP-binding subunit ERF3A (Eukaryotic peptide chain release factor subunit 3a) (eRF3a) (EC 3.6.5.-) (G1 to S phase transition protein 1 homolog)** | **GSPT1 ERF3A** | **499** | **FUNCTION: GTPase component of the eRF1-eRF3-GTP ternary complex, a ternary complex that mediates translation termination in response to the termination codons UAA, UAG and UGA (PubMed:2511002, PubMed:15987998, PubMed:19417105, PubMed:27863242). GSPT1/ERF3A mediates ETF1/ERF1 delivery to stop codons: The eRF1-eRF3-GTP complex binds to a stop codon in the ribosomal A-site (PubMed:27863242). GTP hydrolysis by GSPT1/ERF3A induces a conformational change that leads to its dissociation, permitting ETF1/ERF1 to accommodate fully in the A-site (PubMed:16777602, PubMed:27863242). Component of the transient SURF complex which recruits UPF1 to stalled ribosomes in the context of nonsense-mediated decay (NMD) of mRNAs containing premature stop codons (PubMed:24486019). Required for SHFL-mediated translation termination which inhibits programmed ribosomal frameshifting (-1PRF) of mRNA from viruses and cellular genes (PubMed:30682371). {ECO:0000269|PubMed:15987998, ECO:0000269|PubMed:16777602, ECO:0000269|PubMed:19417105, ECO:0000269|PubMed:24486019, ECO:0000269|PubMed:2511002, ECO:0000269|PubMed:27863242, ECO:0000269|PubMed:30682371}.** | **G1/S transition of mitotic cell cycle [GO:0000082]; nuclear-transcribed mRNA catabolic process, nonsense-mediated decay [GO:0000184]; protein methylation [GO:0006479]; regulation of translational termination [GO:0006449]; translation [GO:0006412]; translational termination [GO:0006415]** | **GO:0000082; GO:0000184; GO:0003723; GO:0003747; GO:0003924; GO:0005525; GO:0005737; GO:0005829; GO:0006412; GO:0006415; GO:0006449; GO:0006479; GO:0018444; GO:0022626** |

Table S6.

| **Supplementary Table 6:** List of the 111 c-MYC target genes with positive GSEA scores in EM127-treated CRC-SCs derived from patients with SMYD3-overexpressing cancers, when compared to untreated CRC-SCs. The 24 genes that are relevant to stemness-related processes based on the experimental annotations from the ChEA database are indicated in bold (https://maayanlab.cloud/Harmonizome/dataset/CHEA+Transcription+Factor+Targets). | | | | | | | | | | |
| --- | --- | --- | --- | --- | --- | --- | --- | --- | --- | --- |
| **Ensembl unique ID** | **Gene symbol** | **log2(FC) (em127/NT)** | **Uniprot entry** | **Entry name** | **Protein names** | **Gene names** | **Length** | **Function [CC]** | **Gene ontology (biological processes)** | **Gene ontology IDs** |
| ENSG00000006652 | IFRD1 | 0.163498732 | O00458 | IFRD1_HUMAN | Interferon-related developmental regulator 1 (Nerve growth factor-inducible protein PC4) | IFRD1 | 451 | FUNCTION: Could play a role in regulating gene activity in the proliferative and/or differentiative pathways induced by NGF. May be an autocrine factor that attenuates or amplifies the initial ligand-induced signal (By similarity). {ECO:0000250}. | muscle cell differentiation [GO:0042692];myoblast fate determination [GO:0007518];negative regulation of axon extension [GO:0030517];negative regulation of collateral sprouting [GO:0048671];skeletal muscle tissue regeneration [GO:0043403];striated muscle tissue development [GO:0014706] | GO:0005634;GO:0007518;GO:0014706;GO:0016528;GO:0030517;GO:0042692;GO:0043403;GO:0048671 |
| ENSG00000004779 | NDUFAB1 | 0.097610797 | O14561 | ACPM_HUMAN | Acyl carrier protein, mitochondrial (ACP) (CI-SDAP) (NADH-ubiquinone oxidoreductase 9.6 kDa subunit) | NDUFAB1 | 156 | FUNCTION: Carrier of the growing fatty acid chain in fatty acid biosynthesis (By similarity) (PubMed:27626371). Accessory and non-catalytic subunit of the mitochondrial membrane respiratory chain NADH dehydrogenase (Complex I), which functions in the transfer of electrons from NADH to the respiratory chain (PubMed:27626371). Accessory protein, of the core iron-sulfur cluster (ISC) assembly complex, that regulates, in association with LYRM4, the stability and the cysteine desulfurase activity of NFS1 and participates in the [2Fe-2S] clusters assembly on the scaffolding protein ISCU (PubMed:31664822). The core iron-sulfur cluster (ISC) assembly complex is involved in the de novo synthesis of a [2Fe-2S] cluster, the first step of the mitochondrial iron-sulfur protein biogenesis. This process is initiated by the cysteine desulfurase complex (NFS1:LYRM4:NDUFAB1) that produces persulfide which is delivered on the scaffold protein ISCU in a FXN-dependent manner. Then this complex is stabilized by FDX2 which provides reducing equivalents to accomplish the [2Fe-2S] cluster assembly. Finally, the [2Fe-2S] cluster is transferred from ISCU to chaperone proteins, including HSCB, HSPA9 and GLRX5 (By similarity). {ECO:0000250|UniProtKB:P52505, ECO:0000250|UniProtKB:Q9H1K1, ECO:0000269|PubMed:27626371, ECO:0000269|PubMed:31664822}. | [2Fe-2S] cluster assembly [GO:0044571];aerobic respiration [GO:0009060];fatty acid biosynthetic process [GO:0006633];iron-sulfur cluster assembly [GO:0016226];mitochondrial electron transport, NADH to ubiquinone [GO:0006120];mitochondrial respiratory chain complex I assembly [GO:0032981];protein lipoylation [GO:0009249];proton motive force-driven mitochondrial ATP synthesis [GO:0042776] | GO:0000035;GO:0000036;GO:0005504;GO:0005509;GO:0005654;GO:0005739;GO:0005743;GO:0005747;GO:0005759;GO:0006120;GO:0006633;GO:0009060;GO:0009249;GO:0016226;GO:0031966;GO:0032981;GO:0042776;GO:0044571;GO:0140978;GO:1990229 |
| **ENSG00000013275** | **PSMC4** | **0.163498732** | **P43686** | **PRS6B_HUMAN** | **26S proteasome regulatory subunit 6B (26S proteasome AAA-ATPase subunit RPT3) (MB67-interacting protein) (MIP224) (Proteasome 26S subunit ATPase 4) (Tat-binding protein 7) (TBP-7)** | **PSMC4 MIP224 TBP7** | **418** | **FUNCTION: Component of the 26S proteasome, a multiprotein complex involved in the ATP-dependent degradation of ubiquitinated proteins. This complex plays a key role in the maintenance of protein homeostasis by removing misfolded or damaged proteins, which could impair cellular functions, and by removing proteins whose functions are no longer required. Therefore, the proteasome participates in numerous cellular processes, including cell cycle progression, apoptosis, or DNA damage repair. PSMC4 belongs to the heterohexameric ring of AAA (ATPases associated with diverse cellular activities) proteins that unfolds ubiquitinated target proteins that are concurrently translocated into a proteolytic chamber and degraded into peptides. {ECO:0000269|PubMed:1317798, ECO:0000269|PubMed:8060531}.** | **blastocyst development [GO:0001824];proteasome-mediated ubiquitin-dependent protein catabolic process [GO:0043161];proteolysis [GO:0006508]** | **GO:0000502;GO:0001824;GO:0005524;GO:0005634;GO:0005654;GO:0005829;GO:0006508;GO:0008540;GO:0016020;GO:0016887;GO:0022624;GO:0036402;GO:0043161** |
| ENSG00000055130 | CUL1 | 0.070389328 | Q13616 | CUL1_HUMAN | Cullin-1 (CUL-1) | CUL1 | 776 | FUNCTION: Core component of multiple cullin-RING-based SCF (SKP1-CUL1-F-box protein) E3 ubiquitin-protein ligase complexes, which mediate the ubiquitination of proteins involved in cell cycle progression, signal transduction and transcription. SCF complexes and ARIH1 collaborate in tandem to mediate ubiquitination of target proteins (PubMed:27565346, PubMed:22017875, PubMed:22017877). In the SCF complex, serves as a rigid scaffold that organizes the SKP1-F-box protein and RBX1 subunits. May contribute to catalysis through positioning of the substrate and the ubiquitin-conjugating enzyme. The E3 ubiquitin-protein ligase activity of the complex is dependent on the neddylation of the cullin subunit and exchange of the substrate recognition component is mediated by TIP120A/CAND1. The functional specificity of the SCF complex depends on the F-box protein as substrate recognition component. SCF(BTRC) and SCF(FBXW11) direct ubiquitination of CTNNB1 and participate in Wnt signaling. SCF(FBXW11) directs ubiquitination of phosphorylated NFKBIA. SCF(BTRC) directs ubiquitination of NFKBIB, NFKBIE, ATF4, SMAD3, SMAD4, CDC25A, FBXO5 and probably NFKB2. SCF(BTRC) and/or SCF(FBXW11) direct ubiquitination of CEP68 (PubMed:25704143, PubMed:25503564). SCF(SKP2) directs ubiquitination of phosphorylated CDKN1B/p27kip and is involved in regulation of G1/S transition. SCF(SKP2) directs ubiquitination of ORC1, CDT1, RBL2, ELF4, CDKN1A, RAG2, FOXO1A, and probably MYC and TAL1. SCF(FBXW7) directs ubiquitination of CCNE1, NOTCH1 released notch intracellular domain (NICD), and probably PSEN1. SCF(FBXW2) directs ubiquitination of GCM1. SCF(FBXO32) directs ubiquitination of MYOD1. SCF(FBXO7) directs ubiquitination of BIRC2 and DLGAP5. SCF(FBXO33) directs ubiquitination of YBX1. SCF(FBXO1) directs ubiquitination of BCL6 and DTL but does not seem to direct ubiquitination of TP53. SCF(BTRC) mediates the ubiquitination of NFKBIA at 'Lys-21' and 'Lys-22';the degradation frees the associated NFKB1-RELA dimer to translocate into the nucleus and to activate transcription. SCF(CCNF) directs ubiquitination of CCP110. SCF(FBXL3) and SCF(FBXL21) direct ubiquitination of CRY1 and CRY2. SCF(FBXO9) directs ubiquitination of TTI1 and TELO2. SCF(FBXO10) directs ubiquitination of BCL2. {ECO:0000269|PubMed:15531760, ECO:0000269|PubMed:15640526, ECO:0000269|PubMed:18644861, ECO:0000269|PubMed:19679664, ECO:0000269|PubMed:22017875, ECO:0000269|PubMed:22017877, ECO:0000269|PubMed:22113614, ECO:0000269|PubMed:22405651, ECO:0000269|PubMed:23263282, ECO:0000269|PubMed:23431138, ECO:0000269|PubMed:25503564, ECO:0000269|PubMed:25704143, ECO:0000269|PubMed:27565346, ECO:0000269|PubMed:9663463}. | animal organ morphogenesis [GO:0009887];cell population proliferation [GO:0008283];G1/S transition of mitotic cell cycle [GO:0000082];intrinsic apoptotic signaling pathway [GO:0097193];positive regulation of canonical NF-kappaB signal transduction [GO:0043123];proteasome-mediated ubiquitin-dependent protein catabolic process [GO:0043161];protein ubiquitination [GO:0016567];SCF-dependent proteasomal ubiquitin-dependent protein catabolic process [GO:0031146] | GO:0000082;GO:0005634;GO:0005654;GO:0005737;GO:0005829;GO:0005886;GO:0008283;GO:0009887;GO:0016567;GO:0019005;GO:0030674;GO:0031146;GO:0031461;GO:0031625;GO:0043123;GO:0043161;GO:0097193;GO:0160072;GO:1990452 |
| **ENSG00000063177** | **RPL18** | **0.042644337** | **Q07020** | **RL18_HUMAN** | **Large ribosomal subunit protein eL18 (60S ribosomal protein L18)** | **RPL18** | **188** | **FUNCTION: Component of the large ribosomal subunit (PubMed:12962325, PubMed:23636399, PubMed:25957688, PubMed:25901680, PubMed:32669547). The ribosome is a large ribonucleoprotein complex responsible for the synthesis of proteins in the cell (PubMed:12962325, PubMed:23636399, PubMed:25957688, PubMed:25901680, PubMed:32669547). {ECO:0000269|PubMed:23636399, ECO:0000269|PubMed:25901680, ECO:0000269|PubMed:25957688, ECO:0000269|PubMed:32669547, ECO:0000305|PubMed:12962325}.** | **cytoplasmic translation [GO:0002181];translation [GO:0006412]** | **GO:0002181;GO:0003723;GO:0003735;GO:0005634;GO:0005730;GO:0005737;GO:0005783;GO:0005791;GO:0005829;GO:0005925;GO:0006412;GO:0016020;GO:0022625;GO:0022626;GO:0042788** |
| ENSG00000074201 | CLNS1A | 0.137503524 | P54105 | ICLN_HUMAN | Methylosome subunit pICln (Chloride channel, nucleotide sensitive 1A) (Chloride conductance regulatory protein ICln) (I(Cln)) (Chloride ion current inducer protein) (ClCI) (Reticulocyte pICln) | CLNS1A CLCI ICLN | 237 | FUNCTION: Involved in both the assembly of spliceosomal snRNPs and the methylation of Sm proteins (PubMed:21081503, PubMed:18984161, PubMed:10330151, PubMed:11713266). Chaperone that regulates the assembly of spliceosomal U1, U2, U4 and U5 small nuclear ribonucleoproteins (snRNPs), the building blocks of the spliceosome, and thereby plays an important role in the splicing of cellular pre-mRNAs (PubMed:18984161, PubMed:10330151). Most spliceosomal snRNPs contain a common set of Sm proteins SNRPB, SNRPD1, SNRPD2, SNRPD3, SNRPE, SNRPF and SNRPG that assemble in a heptameric protein ring on the Sm site of the small nuclear RNA to form the core snRNP (Sm core) (PubMed:10330151). In the cytosol, the Sm proteins SNRPD1, SNRPD2, SNRPE, SNRPF and SNRPG are trapped in an inactive 6S pICln-Sm complex by the chaperone CLNS1A that controls the assembly of the core snRNP (PubMed:10330151, PubMed:18984161). Dissociation by the SMN complex of CLNS1A from the trapped Sm proteins and their transfer to an SMN-Sm complex triggers the assembly of core snRNPs and their transport to the nucleus (PubMed:10330151, PubMed:18984161). {ECO:0000269|PubMed:10330151, ECO:0000269|PubMed:11713266, ECO:0000269|PubMed:18984161, ECO:0000269|PubMed:21081503}. | cell volume homeostasis [GO:0006884];chloride transport [GO:0006821];mRNA cis splicing, via spliceosome [GO:0045292];positive regulation of mRNA splicing, via spliceosome [GO:0048026];spliceosomal snRNP assembly [GO:0000387] | GO:0000387;GO:0003723;GO:0005634;GO:0005654;GO:0005681;GO:0005829;GO:0005856;GO:0005886;GO:0006821;GO:0006884;GO:0034709;GO:0034715;GO:0045292;GO:0048026 |
| ENSG00000075415 | SLC25A3 | 0 | Q00325 | S25A3_HUMAN | Solute carrier family 25 member 3 (Phosphate carrier protein, mitochondrial) (Phosphate transport protein) (PTP) | SLC25A3 PHC OK/SW-cl.48 | 362 | FUNCTION: Inorganic ion transporter that transports phosphate or copper ions across the mitochondrial inner membrane into the matrix compartment (By similarity) (PubMed:17273968, PubMed:29237729). Mediates proton-coupled symport of phosphate ions necessary for mitochondrial oxidative phosphorylation of ADP to ATP (By similarity) (PubMed:17273968). Transports copper ions probably in the form of anionic copper(I) complexes to maintain mitochondrial matrix copper pool and to supply copper for cytochrome C oxidase complex assembly (PubMed:29237729). May also play a role in regulation of the mitochondrial permeability transition pore (mPTP) (By similarity). {ECO:0000250|UniProtKB:P12234, ECO:0000250|UniProtKB:P16036, ECO:0000269|PubMed:17273968, ECO:0000269|PubMed:29237729}. | mitochondrial phosphate ion transmembrane transport [GO:1990547];phosphate ion transmembrane transport [GO:0035435] | GO:0005315;GO:0005739;GO:0005743;GO:0005886;GO:0015317;GO:0016020;GO:0035435;GO:0044877;GO:0070062;GO:1990547 |
| ENSG00000078668 | VDAC3 | 0.070389328 | Q9Y277 | VDAC3_HUMAN | Voltage-dependent anion-selective channel protein 3 (VDAC-3) (hVDAC3) (Outer mitochondrial membrane protein porin 3) | VDAC3 | 283 | FUNCTION: Forms a channel through the mitochondrial outer membrane that allows diffusion of small hydrophilic molecules (By similarity). Involved in male fertility and sperm mitochondrial sheath formation (By similarity). {ECO:0000250|UniProtKB:P21796, ECO:0000250|UniProtKB:Q60931}. | adenine transport [GO:0015853];sperm mitochondrial sheath assembly [GO:0120317];spermatogenesis [GO:0007283] | GO:0000166;GO:0005634;GO:0005739;GO:0005741;GO:0007283;GO:0008308;GO:0015288;GO:0015853;GO:0016020;GO:0046930;GO:0070062;GO:0120317 |
| ENSG00000082898 | XPO1 | 0.014355293 | O14980 | XPO1_HUMAN | Exportin-1 (Exp1) (Chromosome region maintenance 1 protein homolog) | XPO1 CRM1 | 1071 | FUNCTION: Mediates the nuclear export of cellular proteins (cargos) bearing a leucine-rich nuclear export signal (NES) and of RNAs. In the nucleus, in association with RANBP3, binds cooperatively to the NES on its target protein and to the GTPase RAN in its active GTP-bound form (Ran-GTP). Docking of this complex to the nuclear pore complex (NPC) is mediated through binding to nucleoporins. Upon transit of a nuclear export complex into the cytoplasm, disassembling of the complex and hydrolysis of Ran-GTP to Ran-GDP (induced by RANBP1 and RANGAP1, respectively) cause release of the cargo from the export receptor. The directionality of nuclear export is thought to be conferred by an asymmetric distribution of the GTP- and GDP-bound forms of Ran between the cytoplasm and nucleus. Involved in U3 snoRNA transport from Cajal bodies to nucleoli. Binds to late precursor U3 snoRNA bearing a TMG cap. {ECO:0000269|PubMed:15574332, ECO:0000269|PubMed:20921223, ECO:0000269|PubMed:9311922, ECO:0000269|PubMed:9323133};FUNCTION: (Microbial infection) Mediates the export of unspliced or incompletely spliced RNAs out of the nucleus from different viruses including HIV-1, HTLV-1 and influenza A. Interacts with, and mediates the nuclear export of HIV-1 Rev and HTLV-1 Rex proteins. Involved in HTLV-1 Rex multimerization. {ECO:0000269|PubMed:14612415, ECO:0000269|PubMed:9837918}. | mRNA export from nucleus [GO:0006406];nucleocytoplasmic transport [GO:0006913];protein export from nucleus [GO:0006611];protein localization to nucleus [GO:0034504];regulation of centrosome duplication [GO:0010824];regulation of proteasomal ubiquitin-dependent protein catabolic process [GO:0032434];regulation of protein export from nucleus [GO:0046825];ribosomal large subunit export from nucleus [GO:0000055];ribosomal small subunit export from nucleus [GO:0000056];ribosomal subunit export from nucleus [GO:0000054];ribosome biogenesis [GO:0042254] | GO:0000054;GO:0000055;GO:0000056;GO:0000776;GO:0003723;GO:0005049;GO:0005634;GO:0005635;GO:0005642;GO:0005654;GO:0005730;GO:0005737;GO:0005829;GO:0006406;GO:0006611;GO:0006913;GO:0010824;GO:0015030;GO:0016020;GO:0031267;GO:0031965;GO:0032434;GO:0032991;GO:0034504;GO:0042254;GO:0043231;GO:0046825;GO:1990904 |
| ENSG00000084090 | STARD7 | 0.310340121 | Q9NQZ5 | STAR7_HUMAN | StAR-related lipid transfer protein 7, mitochondrial (Gestational trophoblastic tumor protein 1) (START domain-containing protein 7) (StARD7) | STARD7 GTT1 | 370 | FUNCTION: May play a protective role in mucosal tissues by preventing exaggerated allergic responses. {ECO:0000250|UniProtKB:Q8R1R3}. |  | GO:0005741;GO:0008289 |
| ENSG00000088205 | DDX18 | 0.056583528 | Q9NVP1 | DDX18_HUMAN | ATP-dependent RNA helicase DDX18 (EC 3.6.4.13) (DEAD box protein 18) (Myc-regulated DEAD box protein) (MrDb) | DDX18 cPERP-D | 670 | FUNCTION: Probable RNA-dependent helicase. | maturation of LSU-rRNA from tricistronic rRNA transcript (SSU-rRNA, 5.8S rRNA, LSU-rRNA) [GO:0000463] | GO:0000463;GO:0003723;GO:0003724;GO:0005524;GO:0005694;GO:0005730;GO:0016020;GO:0016887 |
| **ENSG00000089157** | **RPLP0** | **0.056583528** | **P05388** | **RLA0_HUMAN** | **Large ribosomal subunit protein uL10 (60S acidic ribosomal protein P0) (60S ribosomal protein L10E)** | **RPLP0** | **317** | **FUNCTION: Ribosomal protein P0 is the functional equivalent of E.coli protein L10.** | **cytoplasmic translation [GO:0002181];ribosomal large subunit assembly [GO:0000027];translation [GO:0006412]** | **GO:0000027;GO:0002181;GO:0003723;GO:0003735;GO:0005634;GO:0005737;GO:0005783;GO:0005829;GO:0005925;GO:0006412;GO:0014069;GO:0016020;GO:0022625;GO:0022626;GO:0036464;GO:0070062;GO:0070180;GO:0098794;GO:1990904** |
| ENSG00000090621 | PABPC4 | 0.137503524 | Q13310 | PABP4_HUMAN | Polyadenylate-binding protein 4 (PABP-4) (Poly(A)-binding protein 4) (Activated-platelet protein 1) (APP-1) (Inducible poly(A)-binding protein) (iPABP) | PABPC4 APP1 PABP4 | 644 | FUNCTION: Binds the poly(A) tail of mRNA. May be involved in cytoplasmic regulatory processes of mRNA metabolism. Can probably bind to cytoplasmic RNA sequences other than poly(A) in vivo (By similarity). {ECO:0000250}. | blood coagulation [GO:0007596];myeloid cell development [GO:0061515];regulation of mRNA stability [GO:0043488];RNA catabolic process [GO:0006401];RNA processing [GO:0006396];translation [GO:0006412] | GO:0003723;GO:0003730;GO:0005634;GO:0005737;GO:0005829;GO:0006396;GO:0006401;GO:0006412;GO:0007596;GO:0008143;GO:0008266;GO:0010494;GO:0043488;GO:0061515;GO:1990904 |
| ENSG00000092199 | HNRNPC | 0.464668267 | P07910 | HNRPC_HUMAN | Heterogeneous nuclear ribonucleoproteins C1/C2 (hnRNP C1/C2) | HNRNPC HNRPC | 306 | FUNCTION: Binds pre-mRNA and nucleates the assembly of 40S hnRNP particles (PubMed:8264621). Interacts with poly-U tracts in the 3'-UTR or 5'-UTR of mRNA and modulates the stability and the level of translation of bound mRNA molecules (PubMed:12509468, PubMed:16010978, PubMed:7567451, PubMed:8264621). Single HNRNPC tetramers bind 230-240 nucleotides. Trimers of HNRNPC tetramers bind 700 nucleotides (PubMed:8264621). May play a role in the early steps of spliceosome assembly and pre-mRNA splicing. N6-methyladenosine (m6A) has been shown to alter the local structure in mRNAs and long non-coding RNAs (lncRNAs) via a mechanism named 'm(6)A-switch', facilitating binding of HNRNPC, leading to regulation of mRNA splicing (PubMed:25719671). {ECO:0000269|PubMed:12509468, ECO:0000269|PubMed:16010978, ECO:0000269|PubMed:25719671, ECO:0000269|PubMed:7567451, ECO:0000269|PubMed:8264621}. | 3'-UTR-mediated mRNA stabilization [GO:0070935];chromatin remodeling [GO:0006338];mRNA splicing, via spliceosome [GO:0000398];negative regulation of telomere maintenance via telomerase [GO:0032211];osteoblast differentiation [GO:0001649];RNA splicing [GO:0008380] | GO:0000398;GO:0000785;GO:0001649;GO:0003723;GO:0003730;GO:0005576;GO:0005634;GO:0005654;GO:0005681;GO:0005697;GO:0005829;GO:0006338;GO:0008266;GO:0008380;GO:0015629;GO:0016020;GO:0031492;GO:0032211;GO:0032991;GO:0042802;GO:0070034;GO:0070062;GO:0070935;GO:0071013;GO:1990247 |
| **ENSG00000096384** | **HSP90AB1** | **0.584962501** | **P08238** | **HS90B_HUMAN** | **Heat shock protein HSP 90-beta (HSP 90) (Heat shock 84 kDa) (HSP 84) (HSP84)** | **HSP90AB1 HSP90B HSPC2 HSPCB** | **724** | **FUNCTION: Molecular chaperone that promotes the maturation, structural maintenance and proper regulation of specific target proteins involved for instance in cell cycle control and signal transduction. Undergoes a functional cycle linked to its ATPase activity. This cycle probably induces conformational changes in the client proteins, thereby causing their activation. Interacts dynamically with various co-chaperones that modulate its substrate recognition, ATPase cycle and chaperone function (PubMed:16478993, PubMed:19696785). Engages with a range of client protein classes via its interaction with various co-chaperone proteins or complexes, that act as adapters, simultaneously able to interact with the specific client and the central chaperone itself. Recruitment of ATP and co-chaperone followed by client protein forms a functional chaperone. After the completion of the chaperoning process, properly folded client protein and co-chaperone leave HSP90 in an ADP-bound partially open conformation and finally, ADP is released from HSP90 which acquires an open conformation for the next cycle (PubMed:27295069, PubMed:26991466). Apart from its chaperone activity, it also plays a role in the regulation of the transcription machinery. HSP90 and its co-chaperones modulate transcription at least at three different levels. They first alter the steady-state levels of certain transcription factors in response to various physiological cues. Second, they modulate the activity of certain epigenetic modifiers, such as histone deacetylases or DNA methyl transferases, and thereby respond to the change in the environment. Third, they participate in the eviction of histones from the promoter region of certain genes and thereby turn on gene expression (PubMed:25973397). Antagonizes STUB1-mediated inhibition of TGF-beta signaling via inhibition of STUB1-mediated SMAD3 ubiquitination and degradation (PubMed:24613385). Promotes cell differentiation by chaperoning BIRC2 and thereby protecting from auto-ubiquitination and degradation by the proteasomal machinery (PubMed:18239673). Main chaperone involved in the phosphorylation/activation of the STAT1 by chaperoning both JAK2 and PRKCE under heat shock and in turn, activates its own transcription (PubMed:20353823). Involved in the translocation into ERGIC (endoplasmic reticulum-Golgi intermediate compartment) of leaderless cargos (lacking the secretion signal sequence) such as the interleukin 1/IL-1;the translocation process is mediated by the cargo receptor TMED10 (PubMed:32272059). {ECO:0000269|PubMed:16478993, ECO:0000269|PubMed:18239673, ECO:0000269|PubMed:19696785, ECO:0000269|PubMed:20353823, ECO:0000269|PubMed:24613385, ECO:0000269|PubMed:32272059, ECO:0000303|PubMed:25973397, ECO:0000303|PubMed:26991466, ECO:0000303|PubMed:27295069};FUNCTION: (Microbial infection) Binding to N.meningitidis NadA stimulates monocytes (PubMed:21949862). Seems to interfere with N.meningitidis NadA-mediated invasion of human cells (Probable). {ECO:0000269|PubMed:21949862, ECO:0000305|PubMed:22066472}.** | **axon extension [GO:0048675];cellular response to heat [GO:0034605];cellular response to interleukin-4 [GO:0071353];central nervous system neuron axonogenesis [GO:0021955];chaperone-mediated protein complex assembly [GO:0051131];establishment of cell polarity [GO:0030010];negative regulation of apoptotic process [GO:0043066];negative regulation of proteasomal protein catabolic process [GO:1901799];negative regulation of proteasomal ubiquitin-dependent protein catabolic process [GO:0032435];negative regulation of protein metabolic process [GO:0051248];negative regulation of transforming growth factor beta activation [GO:1901389];placenta development [GO:0001890];positive regulation of cell differentiation [GO:0045597];positive regulation of cyclin-dependent protein kinase activity [GO:1904031];positive regulation of nitric oxide biosynthetic process [GO:0045429];positive regulation of peptidyl-serine phosphorylation [GO:0033138];positive regulation of phosphatidylinositol 3-kinase signaling/protein kinase B signal transduction [GO:0051897];positive regulation of phosphoprotein phosphatase activity [GO:0032516];positive regulation of protein localization to cell surface [GO:2000010];positive regulation of tau-protein kinase activity [GO:1902949];positive regulation of telomerase activity [GO:0051973];positive regulation of transforming growth factor beta receptor signaling pathway [GO:0030511];protein folding [GO:0006457];protein stabilization [GO:0050821];regulation of cell cycle [GO:0051726];regulation of protein localization [GO:0032880];regulation of protein ubiquitination [GO:0031396];response to unfolded protein [GO:0006986];supramolecular fiber organization [GO:0097435];telomerase holoenzyme complex assembly [GO:1905323];telomere maintenance via telomerase [GO:0007004];virion attachment to host cell [GO:0019062]** | **GO:0001890;GO:0003723;GO:0003725;GO:0005524;GO:0005576;GO:0005634;GO:0005654;GO:0005737;GO:0005739;GO:0005829;GO:0005886;GO:0006457;GO:0006986;GO:0007004;GO:0009986;GO:0016020;GO:0016887;GO:0019062;GO:0019887;GO:0019900;GO:0019901;GO:0021955;GO:0023026;GO:0030010;GO:0030235;GO:0030511;GO:0030911;GO:0031072;GO:0031396;GO:0031625;GO:0032435;GO:0032516;GO:0032880;GO:0032991;GO:0033138;GO:0034605;GO:0034751;GO:0034774;GO:0042277;GO:0042470;GO:0042802;GO:0042803;GO:0042826;GO:0043008;GO:0043025;GO:0043066;GO:0044294;GO:0044295;GO:0045296;GO:0045429;GO:0045597;GO:0046983;GO:0048156;GO:0048471;GO:0048675;GO:0050821;GO:0051082;GO:0051131;GO:0051248;GO:0051726;GO:0051897;GO:0051973;GO:0070062;GO:0070182;GO:0071353;GO:0097435;GO:0097718;GO:0120293;GO:0140662;GO:1901389;GO:1901799;GO:1902949;GO:1904031;GO:1904813;GO:1905323;GO:1990226;GO:1990565;GO:2000010** |
| **ENSG00000099341** | **PSMD8** | **0.35614381** | **P48556** | **PSMD8_HUMAN** | **26S proteasome non-ATPase regulatory subunit 8 (26S proteasome regulatory subunit RPN12) (26S proteasome regulatory subunit S14) (p31)** | **PSMD8** | **350** | **FUNCTION: Component of the 26S proteasome, a multiprotein complex involved in the ATP-dependent degradation of ubiquitinated proteins. This complex plays a key role in the maintenance of protein homeostasis by removing misfolded or damaged proteins, which could impair cellular functions, and by removing proteins whose functions are no longer required. Therefore, the proteasome participates in numerous cellular processes, including cell cycle progression, apoptosis, or DNA damage repair. {ECO:0000269|PubMed:1317798}.** | **proteasome-mediated ubiquitin-dependent protein catabolic process [GO:0043161]** | **GO:0000502;GO:0005634;GO:0005654;GO:0005829;GO:0005838;GO:0008541;GO:0022624;GO:0043161** |
| ENSG00000100028 | SNRPD3 | 0.097610797 | P62318 | SMD3_HUMAN | Small nuclear ribonucleoprotein Sm D3 (Sm-D3) (snRNP core protein D3) | SNRPD3 | 126 | FUNCTION: Plays a role in pre-mRNA splicing as a core component of the spliceosomal U1, U2, U4 and U5 small nuclear ribonucleoproteins (snRNPs), the building blocks of the spliceosome (PubMed:11991638, PubMed:18984161, PubMed:19325628, PubMed:25555158, PubMed:26912367, PubMed:28502770, PubMed:28781166, PubMed:28076346). Component of both the pre-catalytic spliceosome B complex and activated spliceosome C complexes (PubMed:11991638, PubMed:28502770, PubMed:28781166, PubMed:28076346). As a component of the minor spliceosome, involved in the splicing of U12-type introns in pre-mRNAs (PubMed:15146077). As part of the U7 snRNP it is involved in histone pre-mRNA 3'-end processing (By similarity). {ECO:0000250|UniProtKB:P62320, ECO:0000269|PubMed:11991638, ECO:0000269|PubMed:15146077, ECO:0000269|PubMed:18984161, ECO:0000269|PubMed:19325628, ECO:0000269|PubMed:25555158, ECO:0000269|PubMed:26912367, ECO:0000269|PubMed:28076346, ECO:0000269|PubMed:28502770, ECO:0000269|PubMed:28781166}. | 7-methylguanosine cap hypermethylation [GO:0036261];mRNA splicing, via spliceosome [GO:0000398];protein methylation [GO:0006479];RNA splicing [GO:0008380];spliceosomal snRNP assembly [GO:0000387];U2-type prespliceosome assembly [GO:1903241] | GO:0000243;GO:0000387;GO:0000398;GO:0003723;GO:0005634;GO:0005654;GO:0005681;GO:0005682;GO:0005683;GO:0005685;GO:0005686;GO:0005687;GO:0005689;GO:0005697;GO:0005829;GO:0006479;GO:0008380;GO:0016604;GO:0019899;GO:0030532;GO:0034709;GO:0034715;GO:0034719;GO:0036261;GO:0046540;GO:0070034;GO:0071005;GO:0071007;GO:0071011;GO:0071013;GO:0071208;GO:0071209;GO:0097526;GO:1903241 |
| ENSG00000100353 | EIF3D | 0.070389328 | O15371 | EIF3D_HUMAN | Eukaryotic translation initiation factor 3 subunit D (eIF3d) (Eukaryotic translation initiation factor 3 subunit 7) (eIF-3-zeta) (eIF3 p66) | EIF3D EIF3S7 | 548 | FUNCTION: mRNA cap-binding component of the eukaryotic translation initiation factor 3 (eIF-3) complex, a complex required for several steps in the initiation of protein synthesis of a specialized repertoire of mRNAs (PubMed:27462815). The eIF-3 complex associates with the 40S ribosome and facilitates the recruitment of eIF-1, eIF-1A, eIF-2:GTP:methionyl-tRNAi and eIF-5 to form the 43S pre-initiation complex (43S PIC). The eIF-3 complex stimulates mRNA recruitment to the 43S PIC and scanning of the mRNA for AUG recognition. The eIF-3 complex is also required for disassembly and recycling of post-termination ribosomal complexes and subsequently prevents premature joining of the 40S and 60S ribosomal subunits prior to initiation (PubMed:18599441, PubMed:25849773). The eIF-3 complex specifically targets and initiates translation of a subset of mRNAs involved in cell proliferation, including cell cycling, differentiation and apoptosis, and uses different modes of RNA stem-loop binding to exert either translational activation or repression (PubMed:25849773). In the eIF-3 complex, EIF3D specifically recognizes and binds the 7-methylguanosine cap of a subset of mRNAs (PubMed:27462815). {ECO:0000269|PubMed:18599441, ECO:0000269|PubMed:25849773, ECO:0000269|PubMed:27462815};FUNCTION: (Microbial infection) In case of FCV infection, plays a role in the ribosomal termination-reinitiation event leading to the translation of VP2 (PubMed:18056426). {ECO:0000269|PubMed:18056426}. | cap-dependent translational initiation [GO:0002191];formation of cytoplasmic translation initiation complex [GO:0001732];IRES-dependent viral translational initiation [GO:0075522];positive regulation of mRNA binding [GO:1902416];positive regulation of translation [GO:0045727];translational initiation [GO:0006413];viral translational termination-reinitiation [GO:0075525] | GO:0001732;GO:0002191;GO:0003723;GO:0003743;GO:0005829;GO:0005852;GO:0006413;GO:0016020;GO:0016282;GO:0033290;GO:0045202;GO:0045727;GO:0071541;GO:0075522;GO:0075525;GO:0098808;GO:1902416 |
| **ENSG00000100519** | **PSMC6** | **0.042644337** | **P62333** | **PRS10_HUMAN** | **26S proteasome regulatory subunit 10B (26S proteasome AAA-ATPase subunit RPT4) (Proteasome 26S subunit ATPase 6) (Proteasome subunit p42)** | **PSMC6 SUG2** | **389** | **FUNCTION: Component of the 26S proteasome, a multiprotein complex involved in the ATP-dependent degradation of ubiquitinated proteins. This complex plays a key role in the maintenance of protein homeostasis by removing misfolded or damaged proteins, which could impair cellular functions, and by removing proteins whose functions are no longer required. Therefore, the proteasome participates in numerous cellular processes, including cell cycle progression, apoptosis, or DNA damage repair. PSMC6 belongs to the heterohexameric ring of AAA (ATPases associated with diverse cellular activities) proteins that unfolds ubiquitinated target proteins that are concurrently translocated into a proteolytic chamber and degraded into peptides. {ECO:0000269|PubMed:1317798}.** | **positive regulation of inclusion body assembly [GO:0090261];positive regulation of proteasomal protein catabolic process [GO:1901800];positive regulation of RNA polymerase II transcription preinitiation complex assembly [GO:0045899];proteasome-mediated ubiquitin-dependent protein catabolic process [GO:0043161];ubiquitin-dependent ERAD pathway [GO:0030433]** | **GO:0000502;GO:0005524;GO:0005634;GO:0005654;GO:0005829;GO:0008540;GO:0016020;GO:0016234;GO:0016887;GO:0022624;GO:0030433;GO:0030674;GO:0031597;GO:0036402;GO:0042802;GO:0043161;GO:0045899;GO:0070062;GO:0090261;GO:1901800** |
| ENSG00000100632 | ERH | 0.464668267 | P84090 | ERH_HUMAN | Enhancer of rudimentary homolog | ERH | 104 | FUNCTION: May have a role in the cell cycle. | cell cycle [GO:0007049];nucleobase-containing compound metabolic process [GO:0006139];pyrimidine nucleoside metabolic process [GO:0006213] | GO:0003723;GO:0005634;GO:0006139;GO:0006213;GO:0007049;GO:0008327;GO:0030496;GO:0034709 |
| **ENSG00000101182** | **PSMA7** | **0.286881148** | **O14818** | **PSA7_HUMAN** | **Proteasome subunit alpha type-7 (Proteasome subunit RC6-1) (Proteasome subunit XAPC7)** | **PSMA7 HSPC** | **248** | **FUNCTION: Component of the 20S core proteasome complex involved in the proteolytic degradation of most intracellular proteins. This complex plays numerous essential roles within the cell by associating with different regulatory particles. Associated with two 19S regulatory particles, forms the 26S proteasome and thus participates in the ATP-dependent degradation of ubiquitinated proteins. The 26S proteasome plays a key role in the maintenance of protein homeostasis by removing misfolded or damaged proteins that could impair cellular functions, and by removing proteins whose functions are no longer required. Associated with the PA200 or PA28, the 20S proteasome mediates ubiquitin-independent protein degradation. This type of proteolysis is required in several pathways including spermatogenesis (20S-PA200 complex) or generation of a subset of MHC class I-presented antigenic peptides (20S-PA28 complex). Inhibits the transactivation function of HIF-1A under both normoxic and hypoxia-mimicking conditions. The interaction with EMAP2 increases the proteasome-mediated HIF-1A degradation under the hypoxic conditions. Plays a role in hepatitis C virus internal ribosome entry site-mediated translation. Mediates nuclear translocation of the androgen receptor (AR) and thereby enhances androgen-mediated transactivation. Promotes MAVS degradation and thereby negatively regulates MAVS-mediated innate immune response. {ECO:0000269|PubMed:11389899, ECO:0000269|PubMed:11713272, ECO:0000269|PubMed:12119296, ECO:0000269|PubMed:15244466, ECO:0000269|PubMed:19442227, ECO:0000269|PubMed:19734229, ECO:0000269|PubMed:27176742, ECO:0000269|PubMed:8610016}.** | **proteasomal protein catabolic process [GO:0010498];proteasome-mediated ubiquitin-dependent protein catabolic process [GO:0043161]** | **GO:0000502;GO:0005634;GO:0005654;GO:0005737;GO:0005829;GO:0005839;GO:0010498;GO:0019773;GO:0042802;GO:0043161;GO:0070062;GO:0098794** |
| ENSG00000102144 | PGK1 | 0.056583528 | P00558 | PGK1_HUMAN | Phosphoglycerate kinase 1 (EC 2.7.2.3) (Cell migration-inducing gene 10 protein) (Primer recognition protein 2) (PRP 2) | PGK1 PGKA MIG10 OK/SW-cl.110 | 417 | FUNCTION: Catalyzes one of the two ATP producing reactions in the glycolytic pathway via the reversible conversion of 1,3-diphosphoglycerate to 3-phosphoglycerate (PubMed:30323285, PubMed:7391028). In addition to its role as a glycolytic enzyme, it seems that PGK-1 acts as a polymerase alpha cofactor protein (primer recognition protein) (PubMed:2324090). May play a role in sperm motility (PubMed:26677959). {ECO:0000269|PubMed:2324090, ECO:0000269|PubMed:26677959, ECO:0000269|PubMed:30323285, ECO:0000269|PubMed:7391028}. | canonical glycolysis [GO:0061621];cellular response to hypoxia [GO:0071456];epithelial cell differentiation [GO:0030855];gluconeogenesis [GO:0006094];glycolytic process [GO:0006096];negative regulation of angiogenesis [GO:0016525];phosphorylation [GO:0016310];plasminogen activation [GO:0031639] | GO:0004618;GO:0005524;GO:0005615;GO:0005829;GO:0006094;GO:0006096;GO:0016020;GO:0016310;GO:0016525;GO:0030855;GO:0031639;GO:0043531;GO:0045121;GO:0047134;GO:0061621;GO:0070062;GO:0071456 |
| **ENSG00000103035** | **PSMD7** | **0.042644337** | **P51665** | **PSMD7_HUMAN** | **26S proteasome non-ATPase regulatory subunit 7 (26S proteasome regulatory subunit RPN8) (26S proteasome regulatory subunit S12) (Mov34 protein homolog) (Proteasome subunit p40)** | **PSMD7 MOV34L** | **324** | **FUNCTION: Component of the 26S proteasome, a multiprotein complex involved in the ATP-dependent degradation of ubiquitinated proteins. This complex plays a key role in the maintenance of protein homeostasis by removing misfolded or damaged proteins, which could impair cellular functions, and by removing proteins whose functions are no longer required. Therefore, the proteasome participates in numerous cellular processes, including cell cycle progression, apoptosis, or DNA damage repair. {ECO:0000269|PubMed:1317798}.** | **proteasome-mediated ubiquitin-dependent protein catabolic process [GO:0043161]** | **GO:0000502;GO:0005576;GO:0005634;GO:0005654;GO:0005829;GO:0005838;GO:0016020;GO:0034774;GO:0042803;GO:0043161;GO:0070062;GO:1904813** |
| ENSG00000105618 | PRPF31 | 0.150559677 | Q8WWY3 | PRP31_HUMAN | U4/U6 small nuclear ribonucleoprotein Prp31 (Pre-mRNA-processing factor 31) (Serologically defined breast cancer antigen NY-BR-99) (U4/U6 snRNP 61 kDa protein) (Protein 61K) (hPrp31) | PRPF31 PRP31 | 499 | FUNCTION: Involved in pre-mRNA splicing as component of the spliceosome (PubMed:11867543, PubMed:28781166). Required for the assembly of the U4/U5/U6 tri-snRNP complex, one of the building blocks of the spliceosome (PubMed:11867543). {ECO:0000269|PubMed:11867543, ECO:0000269|PubMed:28781166}. | mRNA splicing, via spliceosome [GO:0000398];ribonucleoprotein complex localization [GO:0071166];spliceosomal tri-snRNP complex assembly [GO:0000244] | GO:0000244;GO:0000398;GO:0003723;GO:0005634;GO:0005654;GO:0005684;GO:0005687;GO:0005690;GO:0015030;GO:0016607;GO:0030621;GO:0030622;GO:0042802;GO:0043021;GO:0046540;GO:0070990;GO:0071005;GO:0071011;GO:0071166;GO:0071339;GO:0097526 |
| ENSG00000106028 | SSBP1 | 0.485426827 | Q04837 | SSBP_HUMAN | Single-stranded DNA-binding protein, mitochondrial (Mt-SSB) (MtSSB) (PWP1-interacting protein 17) | SSBP1 SSBP | 148 | FUNCTION: Binds preferentially and cooperatively to pyrimidine rich single-stranded DNA (ss-DNA) (PubMed:21953457, PubMed:23290262, PubMed:31550240). In vitro, required to maintain the copy number of mitochondrial DNA (mtDNA) and plays a crucial role during mtDNA replication by stimulating the activity of the replisome components POLG and TWNK at the replication fork (PubMed:21953457, PubMed:12975372, PubMed:26446790, PubMed:15167897, PubMed:31550240). Promotes the activity of the gamma complex polymerase POLG, largely by organizing the template DNA and eliminating secondary structures to favor ss-DNA conformations that facilitate POLG activity (PubMed:26446790, PubMed:21953457, PubMed:31550240). In addition it is able to promote the 5'-3' unwinding activity of the mtDNA helicase TWNK (PubMed:12975372). May also function in mtDNA repair (PubMed:23290262). {ECO:0000269|PubMed:12975372, ECO:0000269|PubMed:15167897, ECO:0000269|PubMed:21953457, ECO:0000269|PubMed:23290262, ECO:0000269|PubMed:26446790, ECO:0000269|PubMed:31550240}. | DNA unwinding involved in DNA replication [GO:0006268];mitochondrial DNA replication [GO:0006264];mitochondrion morphogenesis [GO:0070584];positive regulation of helicase activity [GO:0051096];positive regulation of mitochondrial DNA replication [GO:0090297];protein homotetramerization [GO:0051289] | GO:0003682;GO:0003697;GO:0003723;GO:0005634;GO:0005739;GO:0005759;GO:0006264;GO:0006268;GO:0042645;GO:0042802;GO:0042803;GO:0051096;GO:0051289;GO:0070062;GO:0070584;GO:0090297 |
| ENSG00000106682 | EIF4H | 0.070389328 | Q15056 | IF4H_HUMAN | Eukaryotic translation initiation factor 4H (eIF-4H) (Williams-Beuren syndrome chromosomal region 1 protein) | EIF4H KIAA0038 WBSCR1 WSCR1 | 248 | FUNCTION: Stimulates the RNA helicase activity of EIF4A in the translation initiation complex. Binds weakly mRNA. {ECO:0000269|PubMed:10585411, ECO:0000269|PubMed:11418588}. | developmental growth [GO:0048589];regulation of translational initiation [GO:0006446];sexual reproduction [GO:0019953] | GO:0003723;GO:0003743;GO:0005829;GO:0005844;GO:0006446;GO:0008135;GO:0016020;GO:0016281;GO:0019953;GO:0045296;GO:0048471;GO:0048589 |
| **ENSG00000108344** | **PSMD3** | **0.150559677** | **O43242** | **PSMD3_HUMAN** | **26S proteasome non-ATPase regulatory subunit 3 (26S proteasome regulatory subunit RPN3) (26S proteasome regulatory subunit S3) (Proteasome subunit p58)** | **PSMD3** | **534** | **FUNCTION: Component of the 26S proteasome, a multiprotein complex involved in the ATP-dependent degradation of ubiquitinated proteins. This complex plays a key role in the maintenance of protein homeostasis by removing misfolded or damaged proteins, which could impair cellular functions, and by removing proteins whose functions are no longer required. Therefore, the proteasome participates in numerous cellular processes, including cell cycle progression, apoptosis, or DNA damage repair. {ECO:0000269|PubMed:1317798}.** | **proteasome-mediated ubiquitin-dependent protein catabolic process [GO:0043161];regulation of protein catabolic process [GO:0042176];ubiquitin-dependent protein catabolic process [GO:0006511]** | **GO:0000502;GO:0005576;GO:0005634;GO:0005654;GO:0005829;GO:0006511;GO:0008541;GO:0016020;GO:0022624;GO:0030234;GO:0034774;GO:0042176;GO:0043161;GO:0070062;GO:1904813** |
| ENSG00000108953 | YWHAE | 0.321928095 | P62258 | 1433E_HUMAN | 14-3-3 protein epsilon (14-3-3E) | YWHAE | 255 | FUNCTION: Adapter protein implicated in the regulation of a large spectrum of both general and specialized signaling pathways. Binds to a large number of partners, usually by recognition of a phosphoserine or phosphothreonine motif. Binding generally results in the modulation of the activity of the binding partner (By similarity). Positively regulates phosphorylated protein HSF1 nuclear export to the cytoplasm (PubMed:12917326). {ECO:0000250|UniProtKB:P62261, ECO:0000269|PubMed:12917326}. | cellular response to heat [GO:0034605];cerebral cortex development [GO:0021987];hippocampus development [GO:0021766];intracellular signal transduction [GO:0035556];MAPK cascade [GO:0000165];membrane repolarization during cardiac muscle cell action potential [GO:0086013];negative regulation of calcium ion export across plasma membrane [GO:1905913];negative regulation of calcium ion transmembrane transporter activity [GO:1901020];negative regulation of peptidyl-serine dephosphorylation [GO:1902309];neuron migration [GO:0001764];positive regulation of protein export from nucleus [GO:0046827];protein localization to nucleus [GO:0034504];protein targeting [GO:0006605];regulation of cytosolic calcium ion concentration [GO:0051480];regulation of heart rate by cardiac conduction [GO:0086091];regulation of heart rate by hormone [GO:0003064];regulation of membrane repolarization [GO:0060306];regulation of mitotic cell cycle [GO:0007346];regulation of potassium ion transmembrane transporter activity [GO:1901016];signal transduction [GO:0007165];substantia nigra development [GO:0021762] | GO:0000165;GO:0001764;GO:0003064;GO:0003723;GO:0005246;GO:0005634;GO:0005737;GO:0005829;GO:0005925;GO:0006605;GO:0007165;GO:0007346;GO:0015459;GO:0016020;GO:0019899;GO:0019903;GO:0019904;GO:0021762;GO:0021766;GO:0021987;GO:0023026;GO:0031625;GO:0034504;GO:0034605;GO:0035556;GO:0042470;GO:0042802;GO:0042826;GO:0044325;GO:0045296;GO:0046827;GO:0046982;GO:0050815;GO:0051219;GO:0051480;GO:0060306;GO:0070062;GO:0086013;GO:0086091;GO:0097110;GO:0140311;GO:1901016;GO:1901020;GO:1902309;GO:1905913 |
| **ENSG00000109475** | **RPL34** | **0.042644337** | **P49207** | **RL34_HUMAN** | **Large ribosomal subunit protein eL34 (60S ribosomal protein L34)** | **RPL34** | **117** | **FUNCTION: Component of the large ribosomal subunit (PubMed:12962325, PubMed:23636399, PubMed:25957688, PubMed:25901680, PubMed:32669547). The ribosome is a large ribonucleoprotein complex responsible for the synthesis of proteins in the cell (PubMed:12962325, PubMed:23636399, PubMed:25957688, PubMed:25901680, PubMed:32669547{ECO:0000269|PubMed:23636399, ECO:0000269|PubMed:25901680, ECO:0000269|PubMed:25957688, ECO:0000269|PubMed:32669547, ECO:0000305|PubMed:12962325}.** | **cytoplasmic translation [GO:0002181];translation [GO:0006412]** | **GO:0002181;GO:0003723;GO:0003735;GO:0005730;GO:0005737;GO:0005783;GO:0005829;GO:0006412;GO:0022625;GO:0022626;GO:0045202;GO:0045296;GO:0070062** |
| ENSG00000109606 | DHX15 | 0.070389328 | O43143 | DHX15_HUMAN | ATP-dependent RNA helicase DHX15 (EC 3.6.4.13) (ATP-dependent RNA helicase #46) (DEAH box protein 15) (Splicing factor Prp43) (hPrp43) | DHX15 DBP1 DDX15 | 795 | FUNCTION: RNA helicase involved in mRNA processing and antiviral innate immunity (PubMed:19432882, PubMed:19103666, PubMed:32179686, PubMed:24990078, PubMed:24782566, PubMed:34161762). Pre-mRNA processing factor involved in disassembly of spliceosomes after the release of mature mRNA (PubMed:19103666). In cooperation with TFIP11 seem to be involved in the transition of the U2, U5 and U6 snRNP-containing IL complex to the snRNP-free IS complex leading to efficient debranching and turnover of excised introns (PubMed:19103666). Plays a key role in antiviral innate immunity by promoting both MAVS-dependent signaling and NLRP6 inflammasome (PubMed:24990078, PubMed:24782566, PubMed:34161762). Acts as an RNA virus sensor: recognizes and binds viral double stranded RNA (dsRNA) and activates the MAVS-dependent signaling to produce interferon-beta and interferon lambda-3 (IFNL3) (PubMed:24990078, PubMed:24782566, PubMed:34161762). Involved in intestinal antiviral innate immunity together with NLRP6: recognizes and binds viral dsRNA and promotes activation of the NLRP6 inflammasome in intestinal epithelial cells to restrict infection by enteric viruses (PubMed:34161762). The NLRP6 inflammasome acts by promoting maturation and secretion of IL18 in the extracellular milieu (PubMed:34161762). Also involved in antibacterial innate immunity by promoting Wnt-induced antimicrobial protein expression in Paneth cells (By similarity). {ECO:0000250|UniProtKB:O35286, ECO:0000269|PubMed:19103666, ECO:0000269|PubMed:19432882, ECO:0000269|PubMed:24782566, ECO:0000269|PubMed:24990078, ECO:0000269|PubMed:32179686, ECO:0000269|PubMed:34161762}. | antiviral innate immune response [GO:0140374];defense response to bacterium [GO:0042742];defense response to virus [GO:0051607];mRNA processing [GO:0006397];mRNA splicing, via spliceosome [GO:0000398];positive regulation of canonical NF-kappaB signal transduction [GO:0043123];response to alkaloid [GO:0043279];response to toxic substance [GO:0009636];RNA splicing [GO:0008380] | GO:0000398;GO:0003723;GO:0003724;GO:0003725;GO:0004386;GO:0005524;GO:0005634;GO:0005654;GO:0005681;GO:0005689;GO:0005730;GO:0006397;GO:0008186;GO:0008380;GO:0009636;GO:0016607;GO:0016887;GO:0042742;GO:0043123;GO:0043279;GO:0051607;GO:0140374 |
| ENSG00000110321 | EIF4G2 | 0.137503524 | P78344 | IF4G2_HUMAN | Eukaryotic translation initiation factor 4 gamma 2 (eIF-4-gamma 2) (eIF-4G 2) (eIF4G 2) (Death-associated protein 5) (DAP-5) (p97) | EIF4G2 DAP5 OK/SW-cl.75 | 907 | FUNCTION: Appears to play a role in the switch from cap-dependent to IRES-mediated translation during mitosis, apoptosis and viral infection. Cleaved by some caspases and viral proteases. {ECO:0000269|PubMed:11511540, ECO:0000269|PubMed:11943866, ECO:0000269|PubMed:9032289, ECO:0000269|PubMed:9049310}. | cell death [GO:0008219];heart development [GO:0007507];macromolecule biosynthetic process [GO:0009059];negative regulation of autophagy [GO:0010507];positive regulation of axon extension [GO:0045773];positive regulation of cell growth [GO:0030307];positive regulation of dendritic spine development [GO:0060999];positive regulation of translation [GO:0045727];regulation of cell cycle [GO:0051726];regulation of translational initiation [GO:0006446] | GO:0003723;GO:0003729;GO:0003743;GO:0005829;GO:0005912;GO:0006446;GO:0007507;GO:0008135;GO:0008219;GO:0009059;GO:0010507;GO:0016020;GO:0016281;GO:0030307;GO:0030424;GO:0045296;GO:0045727;GO:0045773;GO:0051726;GO:0060999 |
| ENSG00000110958 | PTGES3 | 0.23878686 | Q15185 | TEBP_HUMAN | Prostaglandin E synthase 3 (EC 5.3.99.3) (Cytosolic prostaglandin E2 synthase) (cPGES) (Hsp90 co-chaperone) (Progesterone receptor complex p23) (Telomerase-binding protein p23) | PTGES3 P23 TEBP | 160 | FUNCTION: Cytosolic prostaglandin synthase that catalyzes the oxidoreduction of prostaglandin endoperoxide H2 (PGH2) to prostaglandin E2 (PGE2) (PubMed:10922363). Molecular chaperone that localizes to genomic response elements in a hormone-dependent manner and disrupts receptor-mediated transcriptional activation, by promoting disassembly of transcriptional regulatory complexes (PubMed:11274138, PubMed:12077419). Facilitates HIF alpha proteins hydroxylation via interaction with EGLN1/PHD2, leading to recruit EGLN1/PHD2 to the HSP90 pathway (PubMed:24711448). {ECO:0000269|PubMed:10922363, ECO:0000269|PubMed:11274138, ECO:0000269|PubMed:12077419, ECO:0000269|PubMed:24711448}. | chaperone cofactor-dependent protein refolding [GO:0051085];chaperone-mediated protein complex assembly [GO:0051131];cyclooxygenase pathway [GO:0019371];fibroblast proliferation [GO:0048144];glucocorticoid receptor signaling pathway [GO:0042921];glycogen biosynthetic process [GO:0005978];lung saccule development [GO:0060430];positive regulation of phosphorylation [GO:0042327];positive regulation of telomerase activity [GO:0051973];prostaglandin biosynthetic process [GO:0001516];protein folding [GO:0006457];protein stabilization [GO:0050821];signal transduction [GO:0007165];skin development [GO:0043588];telomerase holoenzyme complex assembly [GO:1905323];telomere maintenance [GO:0000723];telomere maintenance via telomerase [GO:0007004] | GO:0000723;GO:0000781;GO:0001516;GO:0003720;GO:0005634;GO:0005654;GO:0005697;GO:0005829;GO:0005978;GO:0006457;GO:0007004;GO:0007165;GO:0019371;GO:0032991;GO:0042327;GO:0042921;GO:0043588;GO:0048144;GO:0050220;GO:0050821;GO:0051082;GO:0051085;GO:0051087;GO:0051131;GO:0051879;GO:0051973;GO:0060430;GO:0070182;GO:0101031;GO:1905323 |
| ENSG00000111906 | HDDC2 | 0.042644337 | Q7Z4H3 | HDDC2_HUMAN | 5'-deoxynucleotidase HDDC2 (EC 3.1.3.89) (HD domain-containing protein 2) (Hepatitis C virus NS5A-transactivated protein 2) (HCV NS5A-transactivated protein 2) | HDDC2 C6orf74 NS5ATP2 CGI-130 | 204 | FUNCTION: Catalyzes the dephosphorylation of the nucleoside 5'-monophosphates deoxyadenosine monophosphate (dAMP), deoxycytidine monophosphate (dCMP), deoxyguanosine monophosphate (dGMP) and deoxythymidine monophosphate (dTMP). {ECO:0000250|UniProtKB:P53144}. |  | GO:0002953;GO:0046872 |
| **ENSG00000114503** | **NCBP2** | **0.028569152** | **P52298** | **NCBP2_HUMAN** | **Nuclear cap-binding protein subunit 2 (20 kDa nuclear cap-binding protein) (Cell proliferation-inducing gene 55 protein) (NCBP 20 kDa subunit) (CBP20) (NCBP-interacting protein 1) (NIP1)** | **NCBP2 CBP20 PIG55** | **156** | **FUNCTION: Component of the cap-binding complex (CBC), which binds co-transcriptionally to the 5' cap of pre-mRNAs and is involved in various processes such as pre-mRNA splicing, translation regulation, nonsense-mediated mRNA decay, RNA-mediated gene silencing (RNAi) by microRNAs (miRNAs) and mRNA export. The CBC complex is involved in mRNA export from the nucleus via its interaction with ALYREF/THOC4/ALY, leading to the recruitment of the mRNA export machinery to the 5' end of mRNA and to mRNA export in a 5' to 3' direction through the nuclear pore. The CBC complex is also involved in mediating U snRNA and intronless mRNAs export from the nucleus. The CBC complex is essential for a pioneer round of mRNA translation, before steady state translation when the CBC complex is replaced by cytoplasmic cap-binding protein eIF4E. The pioneer round of mRNA translation mediated by the CBC complex plays a central role in nonsense-mediated mRNA decay (NMD), NMD only taking place in mRNAs bound to the CBC complex, but not on eIF4E-bound mRNAs. The CBC complex enhances NMD in mRNAs containing at least one exon-junction complex (EJC) via its interaction with UPF1, promoting the interaction between UPF1 and UPF2. The CBC complex is also involved in 'failsafe' NMD, which is independent of the EJC complex, while it does not participate in Staufen-mediated mRNA decay (SMD). During cell proliferation, the CBC complex is also involved in microRNAs (miRNAs) biogenesis via its interaction with SRRT/ARS2, thereby being required for miRNA-mediated RNA interference. The CBC complex also acts as a negative regulator of PARN, thereby acting as an inhibitor of mRNA deadenylation. In the CBC complex, NCBP2/CBP20 recognizes and binds capped RNAs (m7GpppG-capped RNA) but requires NCBP1/CBP80 to stabilize the movement of its N-terminal loop and lock the CBC into a high affinity cap-binding state with the cap structure. The conventional cap-binding complex with NCBP2 binds both small nuclear RNA (snRNA) and messenger (mRNA) and is involved in their export from the nucleus (PubMed:26382858). {ECO:0000269|PubMed:11551508, ECO:0000269|PubMed:15361857, ECO:0000269|PubMed:17190602, ECO:0000269|PubMed:17363367, ECO:0000269|PubMed:17873884, ECO:0000269|PubMed:18369367, ECO:0000269|PubMed:19632182, ECO:0000269|PubMed:26382858}.** | **alternative mRNA splicing, via spliceosome [GO:0000380];cap-dependent translational initiation [GO:0002191];histone mRNA metabolic process [GO:0008334];miRNA-mediated post-transcriptional gene silencing [GO:0035195];mRNA 3'-end processing [GO:0031124];mRNA cis splicing, via spliceosome [GO:0045292];mRNA export from nucleus [GO:0006406];mRNA metabolic process [GO:0016071];mRNA splicing, via spliceosome [GO:0000398];mRNA transcription by RNA polymerase II [GO:0042789];nuclear-transcribed mRNA catabolic process, nonsense-mediated decay [GO:0000184];positive regulation of mRNA 3'-end processing [GO:0031442];positive regulation of RNA export from nucleus [GO:0046833];positive regulation of transcription elongation by RNA polymerase II [GO:0032968];primary miRNA processing [GO:0031053];regulation of translational initiation [GO:0006446];regulatory ncRNA-mediated post-transcriptional gene silencing [GO:0035194];RNA splicing [GO:0008380];snRNA export from nucleus [GO:0006408]** | **GO:0000184;GO:0000339;GO:0000340;GO:0000380;GO:0000398;GO:0002191;GO:0003677;GO:0003723;GO:0003729;GO:0005634;GO:0005654;GO:0005737;GO:0005829;GO:0005845;GO:0005846;GO:0006406;GO:0006408;GO:0006446;GO:0008334;GO:0008380;GO:0016071;GO:0017069;GO:0031053;GO:0031124;GO:0031442;GO:0032968;GO:0034518;GO:0035194;GO:0035195;GO:0042789;GO:0045292;GO:0046833** |
| ENSG00000114767 | RRP9 | 0.189033824 | O43818 | U3IP2_HUMAN | U3 small nucleolar RNA-interacting protein 2 (RRP9 homolog) (U3 small nucleolar ribonucleoprotein-associated 55 kDa protein) (U3 snoRNP-associated 55 kDa protein) (U3-55K) | RRP9 RNU3IP2 U355K | 475 | FUNCTION: Component of a nucleolar small nuclear ribonucleoprotein particle (snoRNP) thought to participate in the processing and modification of pre-ribosomal RNA (pre-rRNA) (PubMed:26867678). Part of the small subunit (SSU) processome, first precursor of the small eukaryotic ribosomal subunit. During the assembly of the SSU processome in the nucleolus, many ribosome biogenesis factors, an RNA chaperone and ribosomal proteins associate with the nascent pre-rRNA and work in concert to generate RNA folding, modifications, rearrangements and cleavage as well as targeted degradation of pre-ribosomal RNA by the RNA exosome (PubMed:34516797). {ECO:0000269|PubMed:26867678, ECO:0000269|PubMed:34516797}. | ribosomal small subunit biogenesis [GO:0042274];rRNA processing [GO:0006364] | GO:0003723;GO:0005654;GO:0005730;GO:0006364;GO:0030515;GO:0031428;GO:0032040;GO:0034511;GO:0042274 |
| ENSG00000114942 | EEF1B2 | 0.111031312 | P24534 | EF1B_HUMAN | Elongation factor 1-beta (EF-1-beta) | EEF1B2 EEF1B EF1B | 225 | FUNCTION: EF-1-beta and EF-1-delta stimulate the exchange of GDP bound to EF-1-alpha to GTP. | translational elongation [GO:0006414] | GO:0003746;GO:0005085;GO:0005737;GO:0005829;GO:0005853;GO:0006414 |
| **ENSG00000115233** | **PSMD14** | **0.464668267** | **O00487** | **PSDE_HUMAN** | **26S proteasome non-ATPase regulatory subunit 14 (EC 3.4.19.-) (26S proteasome regulatory subunit RPN11) (26S proteasome-associated PAD1 homolog 1)** | **PSMD14 POH1** | **310** | **FUNCTION: Component of the 26S proteasome, a multiprotein complex involved in the ATP-dependent degradation of ubiquitinated proteins. This complex plays a key role in the maintenance of protein homeostasis by removing misfolded or damaged proteins, which could impair cellular functions, and by removing proteins whose functions are no longer required. Therefore, the proteasome participates in numerous cellular processes, including cell cycle progression, apoptosis, or DNA damage repair. The PSMD14 subunit is a metalloprotease that specifically cleaves 'Lys-63'-linked polyubiquitin chains within the complex. Plays a role in response to double-strand breaks (DSBs): acts as a regulator of non-homologous end joining (NHEJ) by cleaving 'Lys-63'-linked polyubiquitin, thereby promoting retention of JMJD2A/KDM4A on chromatin and restricting TP53BP1 accumulation. Also involved in homologous recombination repair by promoting RAD51 loading. {ECO:0000269|PubMed:1317798, ECO:0000269|PubMed:22909820, ECO:0000269|PubMed:9374539}.** | **double-strand break repair via homologous recombination [GO:0000724];double-strand break repair via nonhomologous end joining [GO:0006303];proteasome-mediated ubiquitin-dependent protein catabolic process [GO:0043161];protein K63-linked deubiquitination [GO:0070536];regulation of proteasomal protein catabolic process [GO:0061136];response to ethanol [GO:0045471];ubiquitin-dependent protein catabolic process [GO:0006511]** | **GO:0000502;GO:0000724;GO:0005576;GO:0005634;GO:0005654;GO:0005829;GO:0006303;GO:0006511;GO:0008237;GO:0008541;GO:0022624;GO:0031597;GO:0034774;GO:0043161;GO:0045471;GO:0046872;GO:0061133;GO:0061136;GO:0061578;GO:0070536;GO:0070628;GO:0140492;GO:1904813** |
| ENSG00000115241 | PPM1G | 0.137503524 | O15355 | PPM1G_HUMAN | Protein phosphatase 1G (EC 3.1.3.16) (Protein phosphatase 1C) (Protein phosphatase 2C isoform gamma) (PP2C-gamma) (Protein phosphatase magnesium-dependent 1 gamma) | PPM1G PPM1C | 546 |  | peptidyl-threonine dephosphorylation [GO:0035970];protein dephosphorylation [GO:0006470];regulation of cell cycle [GO:0051726] | GO:0004722;GO:0005634;GO:0005654;GO:0005737;GO:0006470;GO:0016020;GO:0017018;GO:0035970;GO:0046872;GO:0051726 |
| ENSG00000115484 | CCT4 | 0.201633861 | P50991 | TCPD_HUMAN | T-complex protein 1 subunit delta (TCP-1-delta) (CCT-delta) (Stimulator of TAR RNA-binding) | CCT4 CCTD SRB | 539 | FUNCTION: Component of the chaperonin-containing T-complex (TRiC), a molecular chaperone complex that assists the folding of proteins upon ATP hydrolysis (PubMed:25467444). The TRiC complex mediates the folding of WRAP53/TCAB1, thereby regulating telomere maintenance (PubMed:25467444). As part of the TRiC complex may play a role in the assembly of BBSome, a complex involved in ciliogenesis regulating transports vesicles to the cilia (PubMed:20080638). The TRiC complex plays a role in the folding of actin and tubulin (Probable). {ECO:0000269|PubMed:20080638, ECO:0000269|PubMed:25467444, ECO:0000305}. | binding of sperm to zona pellucida [GO:0007339];chaperone-mediated protein folding [GO:0061077];positive regulation of establishment of protein localization to telomere [GO:1904851];positive regulation of protein localization to Cajal body [GO:1904871];positive regulation of telomerase activity [GO:0051973];positive regulation of telomerase RNA localization to Cajal body [GO:1904874];positive regulation of telomere maintenance via telomerase [GO:0032212];protein folding [GO:0006457];protein stabilization [GO:0050821];scaRNA localization to Cajal body [GO:0090666] | GO:0002199;GO:0003723;GO:0005524;GO:0005654;GO:0005813;GO:0005829;GO:0005832;GO:0005874;GO:0006457;GO:0007339;GO:0016887;GO:0032212;GO:0042470;GO:0042995;GO:0044183;GO:0044297;GO:0050821;GO:0051082;GO:0051973;GO:0061077;GO:0070062;GO:0090666;GO:0140662;GO:1904851;GO:1904871;GO:1904874 |
| ENSG00000115541 | HSPE1 | 0.014355293 | P61604 | CH10_HUMAN | 10 kDa heat shock protein, mitochondrial (Hsp10) (10 kDa chaperonin) (Chaperonin 10) (CPN10) (Early-pregnancy factor) (EPF) | HSPE1 | 102 | FUNCTION: Co-chaperonin implicated in mitochondrial protein import and macromolecular assembly. Together with Hsp60, facilitates the correct folding of imported proteins. May also prevent misfolding and promote the refolding and proper assembly of unfolded polypeptides generated under stress conditions in the mitochondrial matrix (PubMed:7912672, PubMed:1346131, PubMed:11422376). The functional units of these chaperonins consist of heptameric rings of the large subunit Hsp60, which function as a back-to-back double ring. In a cyclic reaction, Hsp60 ring complexes bind one unfolded substrate protein per ring, followed by the binding of ATP and association with 2 heptameric rings of the co-chaperonin Hsp10. This leads to sequestration of the substrate protein in the inner cavity of Hsp60 where, for a certain period of time, it can fold undisturbed by other cell components. Synchronous hydrolysis of ATP in all Hsp60 subunits results in the dissociation of the chaperonin rings and the release of ADP and the folded substrate protein (Probable). {ECO:0000269|PubMed:11422376, ECO:0000269|PubMed:1346131, ECO:0000269|PubMed:7912672, ECO:0000305|PubMed:25918392}. | activation of cysteine-type endopeptidase activity involved in apoptotic process [GO:0006919];chaperone cofactor-dependent protein refolding [GO:0051085];osteoblast differentiation [GO:0001649];protein folding [GO:0006457];response to unfolded protein [GO:0006986] | GO:0001649;GO:0003723;GO:0005524;GO:0005739;GO:0005759;GO:0006457;GO:0006919;GO:0006986;GO:0016020;GO:0044183;GO:0046872;GO:0051082;GO:0051085;GO:0051087;GO:0070062 |
| ENSG00000115758 | ODC1 | 0.056583528 | P11926 | DCOR_HUMAN | Ornithine decarboxylase (ODC) (EC 4.1.1.17) | ODC1 | 461 | FUNCTION: Catalyzes the first and rate-limiting step of polyamine biosynthesis that converts ornithine into putrescine, which is the precursor for the polyamines, spermidine and spermine. Polyamines are essential for cell proliferation and are implicated in cellular processes, ranging from DNA replication to apoptosis. {ECO:0000269|PubMed:17900240}. | cell population proliferation [GO:0008283];kidney development [GO:0001822];polyamine metabolic process [GO:0006595];positive regulation of cell population proliferation [GO:0008284];putrescine biosynthetic process from ornithine [GO:0033387];regulation of protein catabolic process [GO:0042176];response to virus [GO:0009615] | GO:0001822;GO:0004586;GO:0005737;GO:0005829;GO:0006595;GO:0008283;GO:0008284;GO:0009615;GO:0033387;GO:0042176;GO:0042803 |
| ENSG00000115875 | SRSF7 | 0.028569152 | Q16629 | SRSF7_HUMAN | Serine/arginine-rich splicing factor 7 (Splicing factor 9G8) (Splicing factor, arginine/serine-rich 7) | SRSF7 SFRS7 | 238 | FUNCTION: Required for pre-mRNA splicing. Can also modulate alternative splicing in vitro. Represses the splicing of MAPT/Tau exon 10. May function as export adapter involved in mRNA nuclear export such as of histone H2A. Binds mRNA which is thought to be transferred to the NXF1-NXT1 heterodimer for export (TAP/NXF1 pathway);enhances NXF1-NXT1 RNA-binding activity. RNA-binding is semi-sequence specific. {ECO:0000269|PubMed:11336712, ECO:0000269|PubMed:12667464, ECO:0000269|PubMed:15009664, ECO:0000269|PubMed:18364396}. | cellular response to leukemia inhibitory factor [GO:1990830];mRNA processing [GO:0006397];mRNA splicing, via spliceosome [GO:0000398];mRNA transport [GO:0051028];negative regulation of mRNA splicing, via spliceosome [GO:0048025];RNA splicing [GO:0008380] | GO:0000398;GO:0003723;GO:0003729;GO:0005634;GO:0005654;GO:0005737;GO:0006397;GO:0008270;GO:0008380;GO:0016607;GO:0019904;GO:0048025;GO:0051028;GO:0070062;GO:1990830 |
| **ENSG00000116251** | **RPL22** | **0.23878686** | **P35268** | **RL22_HUMAN** | **Large ribosomal subunit protein eL22 (60S ribosomal protein L22) (EBER-associated protein) (EAP) (Epstein-Barr virus small RNA-associated protein) (Heparin-binding protein HBp15)** | **RPL22** | **128** | **FUNCTION: Component of the large ribosomal subunit (PubMed:23636399, PubMed:32669547). The ribosome is a large ribonucleoprotein complex responsible for the synthesis of proteins in the cell (PubMed:23636399, PubMed:32669547). {ECO:0000269|PubMed:23636399, ECO:0000269|PubMed:32669547}.** | **alpha-beta T cell differentiation [GO:0046632];cytoplasmic translation [GO:0002181];translation [GO:0006412];translation at presynapse [GO:0140236]** | **GO:0002181;GO:0003723;GO:0003735;GO:0005634;GO:0005737;GO:0005829;GO:0005925;GO:0006412;GO:0008201;GO:0022625;GO:0022626;GO:0042802;GO:0046632;GO:0070062;GO:0098793;GO:0098978;GO:0140236;GO:1990904** |
| ENSG00000116649 | SRM | 0.201633861 | P19623 | SPEE_HUMAN | Spermidine synthase (SPDSY) (EC 2.5.1.16) (Putrescine aminopropyltransferase) | SRM SPS1 SRML1 | 302 | FUNCTION: Catalyzes the production of spermidine from putrescine and decarboxylated S-adenosylmethionine (dcSAM). Has a strong preference for putrescine as substrate, and has very low activity towards 1,3-diaminopropane. Has extremely low activity towards spermidine. {ECO:0000269|PubMed:17585781}. | cellular response to leukemia inhibitory factor [GO:1990830];polyamine metabolic process [GO:0006595];spermidine biosynthetic process [GO:0008295] | GO:0004766;GO:0005829;GO:0006595;GO:0008295;GO:0008757;GO:0042802;GO:0042803;GO:1990830 |
| ENSG00000119318 | RAD23B | 0.286881148 | P54727 | RD23B_HUMAN | UV excision repair protein RAD23 homolog B (HR23B) (hHR23B) (XP-C repair-complementing complex 58 kDa protein) (p58) | RAD23B | 409 | FUNCTION: Multiubiquitin chain receptor involved in modulation of proteasomal degradation. Binds to polyubiquitin chains. Proposed to be capable to bind simultaneously to the 26S proteasome and to polyubiquitinated substrates and to deliver ubiquitinated proteins to the proteasome. May play a role in endoplasmic reticulum-associated degradation (ERAD) of misfolded glycoproteins by association with PNGase and delivering deglycosylated proteins to the proteasome;FUNCTION: Involved in global genome nucleotide excision repair (GG-NER) by acting as component of the XPC complex. Cooperatively with CETN2 appears to stabilize XPC. May protect XPC from proteasomal degradation;FUNCTION: The XPC complex is proposed to represent the first factor bound at the sites of DNA damage and together with other core recognition factors, XPA, RPA and the TFIIH complex, is part of the pre-incision (or initial recognition) complex. The XPC complex recognizes a wide spectrum of damaged DNA characterized by distortions of the DNA helix such as single-stranded loops, mismatched bubbles or single-stranded overhangs. The orientation of XPC complex binding appears to be crucial for inducing a productive NER. XPC complex is proposed to recognize and to interact with unpaired bases on the undamaged DNA strand which is followed by recruitment of the TFIIH complex and subsequent scanning for lesions in the opposite strand in a 5'-to-3' direction by the NER machinery. Cyclobutane pyrimidine dimers (CPDs) which are formed upon UV-induced DNA damage esacpe detection by the XPC complex due to a low degree of structural perurbation. Instead they are detected by the UV-DDB complex which in turn recruits and cooperates with the XPC complex in the respective DNA repair. In vitro, the XPC:RAD23B dimer is sufficient to initiate NER;it preferentially binds to cisplatin and UV-damaged double-stranded DNA and also binds to a variety of chemically and structurally diverse DNA adducts. XPC:RAD23B contacts DNA both 5' and 3' of a cisplatin lesion with a preference for the 5' side. XPC:RAD23B induces a bend in DNA upon binding. XPC:RAD23B stimulates the activity of DNA glycosylases TDG and SMUG1. | cellular response to interleukin-7 [GO:0098761];nucleotide-excision repair [GO:0006289];proteasome-mediated ubiquitin-dependent protein catabolic process [GO:0043161];regulation of proteasomal ubiquitin-dependent protein catabolic process [GO:0032434];spermatogenesis [GO:0007283] | GO:0000502;GO:0000978;GO:0003684;GO:0003697;GO:0005634;GO:0005654;GO:0005829;GO:0006289;GO:0007283;GO:0031593;GO:0032434;GO:0043130;GO:0043161;GO:0061629;GO:0070628;GO:0071942;GO:0098761;GO:0140612 |
| ENSG00000119335 | SET | 0.555816155 | Q01105 | SET_HUMAN | Protein SET (HLA-DR-associated protein II) (Inhibitor of granzyme A-activated DNase) (IGAAD) (PHAPII) (Phosphatase 2A inhibitor I2PP2A) (I-2PP2A) (Template-activating factor I) (TAF-I) | SET | 290 | FUNCTION: Multitasking protein, involved in apoptosis, transcription, nucleosome assembly and histone chaperoning. Isoform 2 anti-apoptotic activity is mediated by inhibition of the GZMA-activated DNase, NME1. In the course of cytotoxic T-lymphocyte (CTL)-induced apoptosis, GZMA cleaves SET, disrupting its binding to NME1 and releasing NME1 inhibition. Isoform 1 and isoform 2 are potent inhibitors of protein phosphatase 2A. Isoform 1 and isoform 2 inhibit EP300/CREBBP and PCAF-mediated acetylation of histones (HAT) and nucleosomes, most probably by masking the accessibility of lysines of histones to the acetylases. The predominant target for inhibition is histone H4. HAT inhibition leads to silencing of HAT-dependent transcription and prevents active demethylation of DNA. Both isoforms stimulate DNA replication of the adenovirus genome complexed with viral core proteins;however, isoform 2 specific activity is higher. {ECO:0000269|PubMed:11555662, ECO:0000269|PubMed:12628186}. | DNA replication [GO:0006260];negative regulation of DNA-templated transcription [GO:0045892];negative regulation of histone acetylation [GO:0035067];negative regulation of neuron apoptotic process [GO:0043524];nucleosome assembly [GO:0006334];nucleosome disassembly [GO:0006337] | GO:0000785;GO:0003677;GO:0003682;GO:0004864;GO:0005634;GO:0005654;GO:0005737;GO:0005783;GO:0005811;GO:0005829;GO:0006260;GO:0006334;GO:0006337;GO:0019888;GO:0032991;GO:0035067;GO:0042393;GO:0043524;GO:0045892;GO:0048471 |
| **ENSG00000120705** | **ETF1** | **0** | **P62495** | **ERF1_HUMAN** | **Eukaryotic peptide chain release factor subunit 1 (Eukaryotic release factor 1) (eRF1) (Protein Cl1) (TB3-1)** | **ETF1 ERF1 RF1 SUP45L1** | **437** | **FUNCTION: Component of the eRF1-eRF3-GTP ternary complex, a ternary complex that mediates translation termination in response to the termination codons (PubMed:7990965, PubMed:10676813, PubMed:16777602, PubMed:24486019, PubMed:26245381, PubMed:27863242, PubMed:36638793). The eRF1-eRF3-GTP complex binds to a stop codon in the ribosomal A-site (PubMed:26245381, PubMed:27863242, PubMed:36638793). ETF1/ERF1 is responsible for stop codon recognition and inducing hydrolysis of peptidyl-tRNA (PubMed:26245381, PubMed:27863242, PubMed:36638793). Following GTP hydrolysis, eRF3 (GSPT1/ERF3A or GSPT2/ERF3B) dissociates, permitting ETF1/eRF1 to accommodate fully in the A-site and mediate hydrolysis of peptidyl-tRNA (PubMed:10676813, PubMed:16777602, PubMed:26245381, PubMed:27863242). Component of the transient SURF complex which recruits UPF1 to stalled ribosomes in the context of nonsense-mediated decay (NMD) of mRNAs containing premature stop codons (PubMed:19417104). Required for SHFL-mediated translation termination which inhibits programmed ribosomal frameshifting (-1PRF) of mRNA from viruses and cellular genes (PubMed:30682371). {ECO:0000269|PubMed:10676813, ECO:0000269|PubMed:16777602, ECO:0000269|PubMed:19417104, ECO:0000269|PubMed:24486019, ECO:0000269|PubMed:26245381, ECO:0000269|PubMed:27863242, ECO:0000269|PubMed:30682371, ECO:0000269|PubMed:36638793, ECO:0000269|PubMed:7990965}.** | **cytoplasmic translational termination [GO:0002184];nuclear-transcribed mRNA catabolic process, nonsense-mediated decay [GO:0000184];protein methylation [GO:0006479];regulation of translational termination [GO:0006449];translational termination [GO:0006415]** | **GO:0000184;GO:0002184;GO:0003723;GO:0003747;GO:0004045;GO:0005737;GO:0005829;GO:0006415;GO:0006449;GO:0006479;GO:0008079;GO:0016149;GO:0018444;GO:0022626;GO:0043022;GO:1990825** |
| ENSG00000123131 | PRDX4 | 0.042644337 | Q13162 | PRDX4_HUMAN | Peroxiredoxin-4 (EC 1.11.1.24) (Antioxidant enzyme AOE372) (AOE37-2) (Peroxiredoxin IV) (Prx-IV) (Thioredoxin peroxidase AO372) (Thioredoxin-dependent peroxide reductase A0372) (Thioredoxin-dependent peroxiredoxin 4) | PRDX4 | 271 | FUNCTION: Thiol-specific peroxidase that catalyzes the reduction of hydrogen peroxide and organic hydroperoxides to water and alcohols, respectively. Plays a role in cell protection against oxidative stress by detoxifying peroxides and as sensor of hydrogen peroxide-mediated signaling events. Regulates the activation of NF-kappa-B in the cytosol by a modulation of I-kappa-B-alpha phosphorylation. {ECO:0000269|PubMed:9388242}. | cell redox homeostasis [GO:0045454];extracellular matrix organization [GO:0030198];hydrogen peroxide catabolic process [GO:0042744];I-kappaB phosphorylation [GO:0007252];male gonad development [GO:0008584];negative regulation of male germ cell proliferation [GO:2000255];protein maturation by protein folding [GO:0022417];response to oxidative stress [GO:0006979];spermatogenesis [GO:0007283] | GO:0005576;GO:0005634;GO:0005783;GO:0005829;GO:0006979;GO:0007252;GO:0007283;GO:0008379;GO:0008584;GO:0022417;GO:0030198;GO:0034774;GO:0042744;GO:0042802;GO:0045454;GO:0060090;GO:0070062;GO:0140313;GO:1904813;GO:2000255 |
| ENSG00000124767 | GLO1 | 0.286881148 | Q04760 | LGUL_HUMAN | Lactoylglutathione lyase (EC 4.4.1.5) (Aldoketomutase) (Glyoxalase I) (Glx I) (Ketone-aldehyde mutase) (Methylglyoxalase) (S-D-lactoylglutathione methylglyoxal lyase) | GLO1 | 184 | FUNCTION: Catalyzes the conversion of hemimercaptal, formed from methylglyoxal and glutathione, to S-lactoylglutathione (PubMed:20454679, PubMed:9705294, PubMed:23122816). Involved in the regulation of TNF-induced transcriptional activity of NF-kappa-B (PubMed:19199007). Required for normal osteoclastogenesis (By similarity). {ECO:0000250|UniProtKB:Q9CPU0, ECO:0000269|PubMed:19199007, ECO:0000269|PubMed:20454679, ECO:0000269|PubMed:23122816, ECO:0000269|PubMed:9705294}. | carbohydrate metabolic process [GO:0005975];glutathione metabolic process [GO:0006749];methylglyoxal metabolic process [GO:0009438];negative regulation of apoptotic process [GO:0043066];osteoclast differentiation [GO:0030316];regulation of transcription by RNA polymerase II [GO:0006357] | GO:0004462;GO:0005654;GO:0005737;GO:0005829;GO:0005886;GO:0005975;GO:0006357;GO:0006749;GO:0008270;GO:0009438;GO:0030316;GO:0043066;GO:0070062 |
| ENSG00000125166 | GOT2 | 0.214124805 | P00505 | AATM_HUMAN | Aspartate aminotransferase, mitochondrial (mAspAT) (EC 2.6.1.1) (EC 2.6.1.7) (Fatty acid-binding protein) (FABP-1) (Glutamate oxaloacetate transaminase 2) (Kynurenine aminotransferase 4) (Kynurenine aminotransferase IV) (Kynurenine--oxoglutarate transaminase 4) (Kynurenine--oxoglutarate transaminase IV) (Plasma membrane-associated fatty acid-binding protein) (FABPpm) (Transaminase A) | GOT2 KYAT4 | 430 | FUNCTION: Catalyzes the irreversible transamination of the L-tryptophan metabolite L-kynurenine to form kynurenic acid (KA). As a member of the malate-aspartate shuttle, it has a key role in the intracellular NAD(H) redox balance. Is important for metabolite exchange between mitochondria and cytosol, and for amino acid metabolism. Facilitates cellular uptake of long-chain free fatty acids. {ECO:0000269|PubMed:31422819, ECO:0000269|PubMed:9537447}. | 2-oxoglutarate metabolic process [GO:0006103];4-hydroxyproline catabolic process [GO:0019470];aspartate biosynthetic process [GO:0006532];aspartate catabolic process [GO:0006533];aspartate metabolic process [GO:0006531];fatty acid transport [GO:0015908];glutamate catabolic process to 2-oxoglutarate [GO:0019551];glutamate catabolic process to aspartate [GO:0019550];glutamate metabolic process [GO:0006536];oxaloacetate metabolic process [GO:0006107];response to ethanol [GO:0045471] | GO:0003723;GO:0004069;GO:0005739;GO:0005759;GO:0005886;GO:0006103;GO:0006107;GO:0006531;GO:0006532;GO:0006533;GO:0006536;GO:0015908;GO:0016212;GO:0019470;GO:0019550;GO:0019551;GO:0030170;GO:0045471;GO:0070062 |
| ENSG00000125870 | SNRPB2 | 0.22650853 | P08579 | RU2B_HUMAN | U2 small nuclear ribonucleoprotein B'' (U2 snRNP B'') | SNRPB2 | 225 | FUNCTION: Involved in pre-mRNA splicing as component of the spliceosome (PubMed:11991638, PubMed:28502770, PubMed:28781166, PubMed:28076346). Associated with sn-RNP U2, where it contributes to the binding of stem loop IV of U2 snRNA (PubMed:9716128). {ECO:0000269|PubMed:11991638, ECO:0000269|PubMed:28076346, ECO:0000269|PubMed:28502770, ECO:0000269|PubMed:28781166, ECO:0000269|PubMed:9716128}. | mRNA splicing, via spliceosome [GO:0000398];U2-type prespliceosome assembly [GO:1903241] | GO:0000398;GO:0001650;GO:0005634;GO:0005654;GO:0005681;GO:0005685;GO:0005686;GO:0016607;GO:0030619;GO:0036464;GO:0070990;GO:0071005;GO:0071007;GO:0071013;GO:1903241 |
| ENSG00000125944 | HNRNPR | 0.028569152 | O43390 | HNRPR_HUMAN | Heterogeneous nuclear ribonucleoprotein R (hnRNP R) | HNRNPR HNRPR | 633 | FUNCTION: Component of ribonucleosomes, which are complexes of at least 20 other different heterogeneous nuclear ribonucleoproteins (hnRNP). hnRNP play an important role in processing of precursor mRNA in the nucleus. | circadian rhythm [GO:0007623];mRNA destabilization [GO:0061157];mRNA processing [GO:0006397];mRNA splicing, via spliceosome [GO:0000398] | GO:0000398;GO:0003723;GO:0003729;GO:0003730;GO:0005634;GO:0005654;GO:0005681;GO:0005783;GO:0006397;GO:0007623;GO:0030425;GO:0030426;GO:0043679;GO:0061157;GO:0071013;GO:1990904 |
| ENSG00000125977 | EIF2S2 | 0.275007047 | P20042 | IF2B_HUMAN | Eukaryotic translation initiation factor 2 subunit 2 (Eukaryotic translation initiation factor 2 subunit beta) (eIF-2-beta) | EIF2S2 EIF2B | 333 | FUNCTION: Component of the eIF2 complex that functions in the early steps of protein synthesis by forming a ternary complex with GTP and initiator tRNA. This complex binds to a 40S ribosomal subunit, followed by mRNA binding to form the 43S pre-initiation complex (43S PIC). Junction of the 60S ribosomal subunit to form the 80S initiation complex is preceded by hydrolysis of the GTP bound to eIF2 and release of an eIF2-GDP binary complex. In order for eIF2 to recycle and catalyze another round of initiation, the GDP bound to eIF2 must exchange with GTP by way of a reaction catalyzed by eIF-2B. {ECO:0000250|UniProtKB:P05198}. | formation of cytoplasmic translation initiation complex [GO:0001732];formation of translation preinitiation complex [GO:0001731];in utero embryonic development [GO:0001701];male germ cell proliferation [GO:0002176];male gonad development [GO:0008584];translational initiation [GO:0006413] | GO:0001701;GO:0001731;GO:0001732;GO:0002176;GO:0003723;GO:0003729;GO:0003743;GO:0005737;GO:0005829;GO:0005850;GO:0006413;GO:0008135;GO:0008584;GO:0031369;GO:0045202;GO:0046872 |
| **ENSG00000126067** | **PSMB2** | **0.35614381** | **P49721** | **PSB2_HUMAN** | **Proteasome subunit beta type-2 (Macropain subunit C7-I) (Multicatalytic endopeptidase complex subunit C7-I) (Proteasome component C7-I)** | **PSMB2** | **201** | **FUNCTION: Non-catalytic component of the 20S core proteasome complex involved in the proteolytic degradation of most intracellular proteins. This complex plays numerous essential roles within the cell by associating with different regulatory particles. Associated with two 19S regulatory particles, forms the 26S proteasome and thus participates in the ATP-dependent degradation of ubiquitinated proteins. The 26S proteasome plays a key role in the maintenance of protein homeostasis by removing misfolded or damaged proteins that could impair cellular functions, and by removing proteins whose functions are no longer required. Associated with the PA200 or PA28, the 20S proteasome mediates ubiquitin-independent protein degradation. This type of proteolysis is required in several pathways including spermatogenesis (20S-PA200 complex) or generation of a subset of MHC class I-presented antigenic peptides (20S-PA28 complex). {ECO:0000269|PubMed:15244466, ECO:0000269|PubMed:27176742, ECO:0000269|PubMed:8610016}.** | **proteasomal protein catabolic process [GO:0010498];proteasome-mediated ubiquitin-dependent protein catabolic process [GO:0043161];response to organic cyclic compound [GO:0014070];response to organonitrogen compound [GO:0010243]** | **GO:0000502;GO:0005634;GO:0005654;GO:0005737;GO:0005829;GO:0005839;GO:0010243;GO:0010498;GO:0014070;GO:0016020;GO:0019774;GO:0043161;GO:0070062** |
| ENSG00000126261 | UBA2 | 0.084064265 | Q9UBT2 | SAE2_HUMAN | SUMO-activating enzyme subunit 2 (EC 2.3.2.-) (Anthracycline-associated resistance ARX) (Ubiquitin-like 1-activating enzyme E1B) (Ubiquitin-like modifier-activating enzyme 2) | UBA2 SAE2 UBLE1B HRIHFB2115 | 640 | FUNCTION: The heterodimer acts as an E1 ligase for SUMO1, SUMO2, SUMO3, and probably SUMO4. It mediates ATP-dependent activation of SUMO proteins followed by formation of a thioester bond between a SUMO protein and a conserved active site cysteine residue on UBA2/SAE2. {ECO:0000269|PubMed:11451954, ECO:0000269|PubMed:11481243, ECO:0000269|PubMed:15660128, ECO:0000269|PubMed:17643372, ECO:0000269|PubMed:19443651, ECO:0000269|PubMed:20164921}. | positive regulation of protein sumoylation [GO:0033235];protein sumoylation [GO:0016925] | GO:0000287;GO:0005524;GO:0005654;GO:0005737;GO:0016740;GO:0016925;GO:0019948;GO:0031510;GO:0032183;GO:0033235;GO:0044388;GO:0044390;GO:0046982 |
| ENSG00000127022 | CANX | 0.028569152 | P27824 | CALX_HUMAN | Calnexin (IP90) (Major histocompatibility complex class I antigen-binding protein p88) (p90) | CANX | 592 | FUNCTION: Calcium-binding protein that interacts with newly synthesized monoglucosylated glycoproteins in the endoplasmic reticulum. It may act in assisting protein assembly and/or in the retention within the ER of unassembled protein subunits. It seems to play a major role in the quality control apparatus of the ER by the retention of incorrectly folded proteins. Associated with partial T-cell antigen receptor complexes that escape the ER of immature thymocytes, it may function as a signaling complex regulating thymocyte maturation. Additionally it may play a role in receptor-mediated endocytosis at the synapse. | clathrin-dependent endocytosis [GO:0072583];protein folding [GO:0006457];protein folding in endoplasmic reticulum [GO:0034975];protein secretion [GO:0009306];synaptic vesicle endocytosis [GO:0048488];ubiquitin-dependent ERAD pathway [GO:0030433];viral protein processing [GO:0019082] | GO:0003723;GO:0005509;GO:0005783;GO:0005788;GO:0005789;GO:0006457;GO:0009306;GO:0016020;GO:0019082;GO:0030246;GO:0030433;GO:0031965;GO:0034975;GO:0042470;GO:0044233;GO:0044322;GO:0048488;GO:0051082;GO:0070062;GO:0072583;GO:0098553;GO:0098793 |
| **ENSG00000129084** | **PSMA1** | **0.137503524** | **P25786** | **PSA1_HUMAN** | **Proteasome subunit alpha type-1 (30 kDa prosomal protein) (PROS-30) (Macropain subunit C2) (Multicatalytic endopeptidase complex subunit C2) (Proteasome component C2) (Proteasome nu chain)** | **PSMA1 HC2 NU PROS30 PSC2** | **263** | **FUNCTION: Component of the 20S core proteasome complex involved in the proteolytic degradation of most intracellular proteins. This complex plays numerous essential roles within the cell by associating with different regulatory particles. Associated with two 19S regulatory particles, forms the 26S proteasome and thus participates in the ATP-dependent degradation of ubiquitinated proteins. The 26S proteasome plays a key role in the maintenance of protein homeostasis by removing misfolded or damaged proteins that could impair cellular functions, and by removing proteins whose functions are no longer required. Associated with the PA200 or PA28, the 20S proteasome mediates ubiquitin-independent protein degradation. This type of proteolysis is required in several pathways including spermatogenesis (20S-PA200 complex) or generation of a subset of MHC class I-presented antigenic peptides (20S-PA28 complex). {ECO:0000269|PubMed:15244466, ECO:0000269|PubMed:27176742, ECO:0000269|PubMed:8610016}.** | **immune system process [GO:0002376];negative regulation of inflammatory response to antigenic stimulus [GO:0002862];proteasomal protein catabolic process [GO:0010498];proteasome-mediated ubiquitin-dependent protein catabolic process [GO:0043161]** | **GO:0000502;GO:0001530;GO:0002376;GO:0002862;GO:0003723;GO:0005634;GO:0005654;GO:0005737;GO:0005813;GO:0005829;GO:0005839;GO:0005844;GO:0010498;GO:0019773;GO:0043161;GO:0070062** |
| ENSG00000132341 | RAN | 0.23878686 | P62826 | RAN_HUMAN | GTP-binding nuclear protein Ran (EC 3.6.5.-) (Androgen receptor-associated protein 24) (GTPase Ran) (Ras-like protein TC4) (Ras-related nuclear protein) | RAN ARA24 OK/SW-cl.81 | 216 | FUNCTION: GTPase involved in nucleocytoplasmic transport, participating both to the import and the export from the nucleus of proteins and RNAs (PubMed:10400640, PubMed:8276887, PubMed:8896452, PubMed:8636225, PubMed:8692944, PubMed:9351834, PubMed:9428644, PubMed:9822603, PubMed:17209048, PubMed:26272610, PubMed:27306458). Switches between a cytoplasmic GDP- and a nuclear GTP-bound state by nucleotide exchange and GTP hydrolysis (PubMed:7819259, PubMed:8896452, PubMed:8636225, PubMed:8692944, PubMed:9351834, PubMed:9428644, PubMed:9822603, PubMed:29040603, PubMed:11336674, PubMed:26272610). Nuclear import receptors such as importin beta bind their substrates only in the absence of GTP-bound RAN and release them upon direct interaction with GTP-bound RAN, while export receptors behave in the opposite way. Thereby, RAN controls cargo loading and release by transport receptors in the proper compartment and ensures the directionality of the transport (PubMed:8896452, PubMed:9351834, PubMed:9428644). Interaction with RANBP1 induces a conformation change in the complex formed by XPO1 and RAN that triggers the release of the nuclear export signal of cargo proteins (PubMed:20485264). RAN (GTP-bound form) triggers microtubule assembly at mitotic chromosomes and is required for normal mitotic spindle assembly and chromosome segregation (PubMed:10408446, PubMed:29040603). Required for normal progress through mitosis (PubMed:8421051, PubMed:12194828, PubMed:29040603). The complex with BIRC5/survivin plays a role in mitotic spindle formation by serving as a physical scaffold to help deliver the RAN effector molecule TPX2 to microtubules (PubMed:18591255). Acts as a negative regulator of the kinase activity of VRK1 and VRK2 (PubMed:18617507). Enhances AR-mediated transactivation. Transactivation decreases as the poly-Gln length within AR increases (PubMed:10400640). {ECO:0000269|PubMed:10400640, ECO:0000269|PubMed:10408446, ECO:0000269|PubMed:11336674, ECO:0000269|PubMed:12194828, ECO:0000269|PubMed:17209048, ECO:0000269|PubMed:18591255, ECO:0000269|PubMed:18617507, ECO:0000269|PubMed:20485264, ECO:0000269|PubMed:26272610, ECO:0000269|PubMed:27306458, ECO:0000269|PubMed:29040603, ECO:0000269|PubMed:7819259, ECO:0000269|PubMed:8276887, ECO:0000269|PubMed:8421051, ECO:0000269|PubMed:8636225, ECO:0000269|PubMed:8692944, ECO:0000269|PubMed:8896452, ECO:0000269|PubMed:9351834, ECO:0000269|PubMed:9428644, ECO:0000269|PubMed:9822603, ECO:0000305|PubMed:26272610}. | actin cytoskeleton organization [GO:0030036];cell division [GO:0051301];cellular response to mineralocorticoid stimulus [GO:0071389];DNA metabolic process [GO:0006259];GTP metabolic process [GO:0046039];hippocampus development [GO:0021766];mitotic cell cycle [GO:0000278];mitotic sister chromatid segregation [GO:0000070];mitotic spindle organization [GO:0007052];positive regulation of protein binding [GO:0032092];positive regulation of protein import into nucleus [GO:0042307];pre-miRNA export from nucleus [GO:0035281];protein export from nucleus [GO:0006611];protein import into nucleus [GO:0006606];protein localization to nucleolus [GO:1902570];ribosomal large subunit export from nucleus [GO:0000055];ribosomal small subunit export from nucleus [GO:0000056];ribosomal subunit export from nucleus [GO:0000054];snRNA import into nucleus [GO:0061015];spermatid development [GO:0007286];viral process [GO:0016032] | GO:0000054;GO:0000055;GO:0000056;GO:0000070;GO:0000278;GO:0000287;GO:0001673;GO:0002177;GO:0003682;GO:0003723;GO:0003924;GO:0003925;GO:0005525;GO:0005634;GO:0005635;GO:0005643;GO:0005654;GO:0005730;GO:0005737;GO:0005814;GO:0005829;GO:0006259;GO:0006606;GO:0006611;GO:0007052;GO:0007286;GO:0016020;GO:0016032;GO:0019003;GO:0019904;GO:0021766;GO:0030036;GO:0030496;GO:0032092;GO:0032991;GO:0035281;GO:0036126;GO:0042307;GO:0042470;GO:0042565;GO:0045296;GO:0045505;GO:0046039;GO:0046982;GO:0051301;GO:0055037;GO:0061015;GO:0061676;GO:0070062;GO:0071389;GO:1902570 |
| ENSG00000134001 | EIF2S1 | 0.028569152 | P05198 | IF2A_HUMAN | Eukaryotic translation initiation factor 2 subunit 1 (Eukaryotic translation initiation factor 2 subunit alpha) (eIF-2-alpha) (eIF-2A) (eIF-2alpha) (eIF2-alpha) | EIF2S1 EIF2A | 315 | FUNCTION: Member of the eIF2 complex that functions in the early steps of protein synthesis by forming a ternary complex with GTP and initiator tRNA (PubMed:16289705). This complex binds to a 40S ribosomal subunit, followed by mRNA binding to form a 43S pre-initiation complex (43S PIC) (PubMed:16289705). Junction of the 60S ribosomal subunit to form the 80S initiation complex is preceded by hydrolysis of the GTP bound to eIF2 and release of an eIF2-GDP binary complex (PubMed:16289705). In order for eIF2 to recycle and catalyze another round of initiation, the GDP bound to eIF2 must exchange with GTP by way of a reaction catalyzed by eIF-2B (PubMed:16289705). EIF2S1/eIF2-alpha is a key component of the integrated stress response (ISR), required for adaptation to various stress: phosphorylation by metabolic-stress sensing protein kinases (EIF2AK1/HRI, EIF2AK2/PKR, EIF2AK3/PERK and EIF2AK4/GCN2) in response to stress converts EIF2S1/eIF2-alpha in a global protein synthesis inhibitor, leading to an attenuation of cap-dependent translation, while concomitantly initiating the preferential translation of ISR-specific mRNAs, such as the transcriptional activators ATF4 and QRICH1, and hence allowing ATF4- and QRICH1-mediated reprogramming (PubMed:19131336, PubMed:33384352). {ECO:0000269|PubMed:16289705, ECO:0000269|PubMed:19131336, ECO:0000269|PubMed:33384352}. | cellular response to amino acid starvation [GO:0034198];cellular response to heat [GO:0034605];cellular response to oxidative stress [GO:0034599];cellular response to UV [GO:0034644];negative regulation of translational initiation in response to stress [GO:0032057];PERK-mediated unfolded protein response [GO:0036499];positive regulation of type B pancreatic cell apoptotic process [GO:2000676];regulation of translation in response to endoplasmic reticulum stress [GO:0036490];regulation of translational initiation in response to stress [GO:0043558];response to endoplasmic reticulum stress [GO:0034976];response to kainic acid [GO:1904373];response to manganese-induced endoplasmic reticulum stress [GO:1990737];stress granule assembly [GO:0034063];translational initiation [GO:0006413] | GO:0003723;GO:0003743;GO:0005634;GO:0005829;GO:0005844;GO:0005850;GO:0006413;GO:0010494;GO:0016020;GO:0032057;GO:0033290;GO:0034063;GO:0034198;GO:0034599;GO:0034605;GO:0034644;GO:0034976;GO:0036490;GO:0036499;GO:0043022;GO:0043558;GO:0044207;GO:0045202;GO:0070062;GO:0097451;GO:1904373;GO:1990737;GO:2000676 |
| ENSG00000134308 | YWHAQ | 0.298658316 | P27348 | 1433T_HUMAN | 14-3-3 protein theta (14-3-3 protein T-cell) (14-3-3 protein tau) (Protein HS1) | YWHAQ | 245 | FUNCTION: Adapter protein implicated in the regulation of a large spectrum of both general and specialized signaling pathways. Binds to a large number of partners, usually by recognition of a phosphoserine or phosphothreonine motif. Binding generally results in the modulation of the activity of the binding partner. Negatively regulates the kinase activity of PDPK1. {ECO:0000269|PubMed:12177059}. | negative regulation of DNA-templated transcription [GO:0045892];negative regulation of monoatomic ion transmembrane transport [GO:0034766];protein targeting [GO:0006605];signal transduction [GO:0007165];small GTPase mediated signal transduction [GO:0007264];substantia nigra development [GO:0021762] | GO:0005737;GO:0005829;GO:0005925;GO:0006605;GO:0007165;GO:0007264;GO:0016020;GO:0019904;GO:0021762;GO:0032991;GO:0034766;GO:0042802;GO:0044325;GO:0045202;GO:0045892;GO:0070062;GO:0071889 |
| ENSG00000134333 | LDHA | 0.275007047 | P00338 | LDHA_HUMAN | L-lactate dehydrogenase A chain (LDH-A) (EC 1.1.1.27) (Cell proliferation-inducing gene 19 protein) (LDH muscle subunit) (LDH-M) (Renal carcinoma antigen NY-REN-59) | LDHA PIG19 | 332 | FUNCTION: Interconverts simultaneously and stereospecifically pyruvate and lactate with concomitant interconversion of NADH and NAD(+). {ECO:0000269|PubMed:11276087}. | glycolytic process [GO:0006096];lactate metabolic process [GO:0006089];pyruvate metabolic process [GO:0006090];substantia nigra development [GO:0021762] | GO:0004459;GO:0005634;GO:0005739;GO:0005829;GO:0006089;GO:0006090;GO:0006096;GO:0016020;GO:0021762;GO:0042802;GO:0045296;GO:0070062;GO:1990204 |
| **ENSG00000135446** | **CDK4** | **0.23878686** | **P11802** | **CDK4_HUMAN** | **Cyclin-dependent kinase 4 (EC 2.7.11.22) (Cell division protein kinase 4) (PSK-J3)** | **CDK4** | **303** | **FUNCTION: Ser/Thr-kinase component of cyclin D-CDK4 (DC) complexes that phosphorylate and inhibit members of the retinoblastoma (RB) protein family including RB1 and regulate the cell-cycle during G(1)/S transition. Phosphorylation of RB1 allows dissociation of the transcription factor E2F from the RB/E2F complexes and the subsequent transcription of E2F target genes which are responsible for the progression through the G(1) phase. Hypophosphorylates RB1 in early G(1) phase. Cyclin D-CDK4 complexes are major integrators of various mitogenenic and antimitogenic signals. Also phosphorylates SMAD3 in a cell-cycle-dependent manner and represses its transcriptional activity. Component of the ternary complex, cyclin D/CDK4/CDKN1B, required for nuclear translocation and activity of the cyclin D-CDK4 complex. {ECO:0000269|PubMed:15241418, ECO:0000269|PubMed:18827403, ECO:0000269|PubMed:9003781}.** | **cell division [GO:0051301];cellular response to interleukin-4 [GO:0071353];cellular response to ionomycin [GO:1904637];cellular response to lipopolysaccharide [GO:0071222];cellular response to phorbol 13-acetate 12-myristate [GO:1904628];G1/S transition of mitotic cell cycle [GO:0000082];positive regulation of cell population proliferation [GO:0008284];positive regulation of fibroblast proliferation [GO:0048146];positive regulation of G2/M transition of mitotic cell cycle [GO:0010971];protein phosphorylation [GO:0006468];regulation of cell cycle [GO:0051726];regulation of G2/M transition of mitotic cell cycle [GO:0010389];regulation of gene expression [GO:0010468];regulation of transcription initiation by RNA polymerase II [GO:0060260];regulation of type B pancreatic cell proliferation [GO:0061469];response to organic substance [GO:0010033];response to xenobiotic stimulus [GO:0009410];signal transduction [GO:0007165]** | **GO:0000082;GO:0000307;GO:0000785;GO:0004693;GO:0005524;GO:0005634;GO:0005654;GO:0005667;GO:0005730;GO:0005737;GO:0005829;GO:0005923;GO:0006468;GO:0007165;GO:0008284;GO:0009410;GO:0010033;GO:0010389;GO:0010468;GO:0010971;GO:0016538;GO:0030332;GO:0031965;GO:0048146;GO:0051301;GO:0051726;GO:0060260;GO:0061469;GO:0071222;GO:0071353;GO:0097128;GO:0097129;GO:0097130;GO:0106310;GO:1904628;GO:1904637** |
| ENSG00000135486 | HNRNPA1 | 0.344828497 | P09651 | ROA1_HUMAN | Heterogeneous nuclear ribonucleoprotein A1 (hnRNP A1) (Helix-destabilizing protein) (Single-strand RNA-binding protein) (hnRNP core protein A1) [Cleaved into: Heterogeneous nuclear ribonucleoprotein A1, N-terminally processed] | HNRNPA1 HNRPA1 | 372 | FUNCTION: Involved in the packaging of pre-mRNA into hnRNP particles, transport of poly(A) mRNA from the nucleus to the cytoplasm and modulation of splice site selection (PubMed:17371836). Plays a role in the splicing of pyruvate kinase PKM by binding repressively to sequences flanking PKM exon 9, inhibiting exon 9 inclusion and resulting in exon 10 inclusion and production of the PKM M2 isoform (PubMed:20010808). Binds to the IRES and thereby inhibits the translation of the apoptosis protease activating factor APAF1 (PubMed:31498791). May bind to specific miRNA hairpins (PubMed:28431233). {ECO:0000269|PubMed:17371836, ECO:0000269|PubMed:20010808, ECO:0000269|PubMed:28431233, ECO:0000269|PubMed:31498791};FUNCTION: (Microbial infection) May play a role in HCV RNA replication. {ECO:0000269|PubMed:17229681};FUNCTION: (Microbial infection) Cleavage by Enterovirus 71 protease 3C results in increased translation of apoptosis protease activating factor APAF1, leading to apoptosis. {ECO:0000269|PubMed:17229681}. | amyloid fibril formation [GO:1990000];cellular response to glucose starvation [GO:0042149];cellular response to sodium arsenite [GO:1903936];import into nucleus [GO:0051170];localization [GO:0051179];mRNA splicing, via spliceosome [GO:0000398];mRNA transport [GO:0051028];negative regulation of telomere maintenance via telomerase [GO:0032211];nuclear export [GO:0051168];positive regulation of telomere maintenance via telomerase [GO:0032212];regulation of alternative mRNA splicing, via spliceosome [GO:0000381];regulation of RNA splicing [GO:0043484];RNA export from nucleus [GO:0006405] | GO:0000381;GO:0000398;GO:0003677;GO:0003697;GO:0003723;GO:0003727;GO:0003730;GO:0005634;GO:0005654;GO:0005681;GO:0005737;GO:0005829;GO:0006405;GO:0016020;GO:0019904;GO:0032211;GO:0032212;GO:0035198;GO:0036002;GO:0042149;GO:0042802;GO:0043232;GO:0043484;GO:0051028;GO:0051168;GO:0051170;GO:0051179;GO:0061752;GO:0070062;GO:0071013;GO:0098505;GO:0140693;GO:1903936;GO:1990000;GO:1990904 |
| ENSG00000136045 | PWP1 | 0.176322773 | Q13610 | PWP1_HUMAN | Periodic tryptophan protein 1 homolog (Keratinocyte protein IEF SSP 9502) | PWP1 | 501 | FUNCTION: Chromatin-associated factor that regulates transcription (PubMed:29065309). Regulates Pol I-mediated rRNA biogenesis and, probably, Pol III-mediated transcription (PubMed:29065309). Regulates the epigenetic status of rDNA (PubMed:29065309). {ECO:0000269|PubMed:29065309}. | DNA-templated transcription [GO:0006351];negative regulation of peptidyl-serine phosphorylation of STAT protein [GO:0033140];positive regulation of stem cell differentiation [GO:2000738];positive regulation of transcription of nucleolar large rRNA by RNA polymerase I [GO:1901838];rRNA processing [GO:0006364] | GO:0005634;GO:0005694;GO:0005730;GO:0005794;GO:0006351;GO:0006364;GO:0033140;GO:0140713;GO:1901838;GO:1990889;GO:2000738 |
| ENSG00000136628 | EPRS1 | 0.201633861 | P07814 | SYEP_HUMAN | Bifunctional glutamate/proline--tRNA ligase (Bifunctional aminoacyl-tRNA synthetase) (Cell proliferation-inducing gene 32 protein) (Glutamatyl-prolyl-tRNA synthetase) [Includes: Glutamate--tRNA ligase (EC 6.1.1.17) (Glutamyl-tRNA synthetase) (GluRS);Proline--tRNA ligase (EC 6.1.1.15) (Prolyl-tRNA synthetase)] | EPRS1 EPRS GLNS PARS QARS QPRS PIG32 | 1512 | FUNCTION: Multifunctional protein which is primarily part of the aminoacyl-tRNA synthetase multienzyme complex, also know as multisynthetase complex, that catalyzes the attachment of the cognate amino acid to the corresponding tRNA in a two-step reaction: the amino acid is first activated by ATP to form a covalent intermediate with AMP and is then transferred to the acceptor end of the cognate tRNA (PubMed:1756734, PubMed:24100331, PubMed:23263184). The phosphorylation of EPRS1, induced by interferon-gamma, dissociates the protein from the aminoacyl-tRNA synthetase multienzyme complex and recruits it to the GAIT complex that binds to stem loop-containing GAIT elements in the 3'-UTR of diverse inflammatory mRNAs (such as ceruplasmin), suppressing their translation. Interferon-gamma can therefore redirect, in specific cells, the EPRS1 function from protein synthesis to translation inhibition (PubMed:15479637, PubMed:23071094). Also functions as an effector of the mTORC1 signaling pathway by promoting, through SLC27A1, the uptake of long-chain fatty acid by adipocytes. Thereby, it also plays a role in fat metabolism and more indirectly influences lifespan (PubMed:28178239). {ECO:0000269|PubMed:15479637, ECO:0000269|PubMed:1756734, ECO:0000269|PubMed:23071094, ECO:0000269|PubMed:23263184, ECO:0000269|PubMed:24100331, ECO:0000269|PubMed:28178239}. | cellular response to insulin stimulus [GO:0032869];cellular response to type II interferon [GO:0071346];glutamyl-tRNA aminoacylation [GO:0006424];negative regulation of translation [GO:0017148];prolyl-tRNA aminoacylation [GO:0006433];protein-containing complex assembly [GO:0065003];regulation of long-chain fatty acid import into cell [GO:0140212];tRNA aminoacylation for protein translation [GO:0006418] | GO:0004818;GO:0004827;GO:0005524;GO:0005737;GO:0005829;GO:0005886;GO:0006418;GO:0006424;GO:0006433;GO:0008270;GO:0016020;GO:0017101;GO:0017148;GO:0032869;GO:0035613;GO:0042802;GO:0042803;GO:0051020;GO:0065003;GO:0071346;GO:0097452;GO:0140212;GO:1990904 |
| **ENSG00000137154** | **RPS6** | **0.454175893** | **P62753** | **RS6_HUMAN** | **Small ribosomal subunit protein eS6 (40S ribosomal protein S6) (Phosphoprotein NP33)** | **RPS6 OK/SW-cl.2** | **249** | **FUNCTION: Component of the 40S small ribosomal subunit (PubMed:8706699, PubMed:23636399). Plays an important role in controlling cell growth and proliferation through the selective translation of particular classes of mRNA (PubMed:17220279). Part of the small subunit (SSU) processome, first precursor of the small eukaryotic ribosomal subunit. During the assembly of the SSU processome in the nucleolus, many ribosome biogenesis factors, an RNA chaperone and ribosomal proteins associate with the nascent pre-rRNA and work in concert to generate RNA folding, modifications, rearrangements and cleavage as well as targeted degradation of pre-ribosomal RNA by the RNA exosome (PubMed:34516797). {ECO:0000269|PubMed:17220279, ECO:0000269|PubMed:23636399, ECO:0000269|PubMed:34516797, ECO:0000269|PubMed:8706699}.** | **activation-induced cell death of T cells [GO:0006924];cytoplasmic translation [GO:0002181];erythrocyte development [GO:0048821];G1/S transition of mitotic cell cycle [GO:0000082];gastrulation [GO:0007369];glucose homeostasis [GO:0042593];mammalian oogenesis stage [GO:0022605];negative regulation of apoptotic process [GO:0043066];placenta development [GO:0001890];positive regulation of apoptotic process [GO:0043065];positive regulation of cell population proliferation [GO:0008284];ribosomal small subunit biogenesis [GO:0042274];rRNA processing [GO:0006364];T cell differentiation in thymus [GO:0033077];T cell proliferation involved in immune response [GO:0002309];TOR signaling [GO:0031929];translation [GO:0006412]** | **GO:0000082;GO:0001890;GO:0002181;GO:0002309;GO:0003723;GO:0003735;GO:0005634;GO:0005654;GO:0005730;GO:0005737;GO:0005783;GO:0005829;GO:0005844;GO:0006364;GO:0006412;GO:0006924;GO:0007369;GO:0008284;GO:0015935;GO:0016020;GO:0019901;GO:0022605;GO:0022626;GO:0022627;GO:0030425;GO:0031929;GO:0032040;GO:0033077;GO:0036464;GO:0042274;GO:0042593;GO:0043065;GO:0043066;GO:0044297;GO:0048471;GO:0048821;GO:1990904** |
| ENSG00000138668 | HNRNPD | 0.137503524 | Q14103 | HNRPD_HUMAN | Heterogeneous nuclear ribonucleoprotein D0 (hnRNP D0) (AU-rich element RNA-binding protein 1) | HNRNPD AUF1 HNRPD | 355 | FUNCTION: Binds with high affinity to RNA molecules that contain AU-rich elements (AREs) found within the 3'-UTR of many proto-oncogenes and cytokine mRNAs. Also binds to double- and single-stranded DNA sequences in a specific manner and functions a transcription factor. Each of the RNA-binding domains specifically can bind solely to a single-stranded non-monotonous 5'-UUAG-3' sequence and also weaker to the single-stranded 5'-TTAGGG-3' telomeric DNA repeat. Binds RNA oligonucleotides with 5'-UUAGGG-3' repeats more tightly than the telomeric single-stranded DNA 5'-TTAGGG-3' repeats. Binding of RRM1 to DNA inhibits the formation of DNA quadruplex structure which may play a role in telomere elongation. May be involved in translationally coupled mRNA turnover. Implicated with other RNA-binding proteins in the cytoplasmic deadenylation/translational and decay interplay of the FOS mRNA mediated by the major coding-region determinant of instability (mCRD) domain. May play a role in the regulation of the rhythmic expression of circadian clock core genes. Directly binds to the 3'UTR of CRY1 mRNA and induces CRY1 rhythmic translation. May also be involved in the regulation of PER2 translation. {ECO:0000269|PubMed:10080887, ECO:0000269|PubMed:11051545, ECO:0000269|PubMed:24423872}. | 3'-UTR-mediated mRNA destabilization [GO:0061158];cellular response to amino acid stimulus [GO:0071230];cellular response to estradiol stimulus [GO:0071392];cellular response to nitric oxide [GO:0071732];cellular response to putrescine [GO:1904586];cerebellum development [GO:0021549];circadian regulation of translation [GO:0097167];CRD-mediated mRNA stabilization [GO:0070934];hepatocyte dedifferentiation [GO:1990828];liver development [GO:0001889];mRNA transcription by RNA polymerase II [GO:0042789];negative regulation of nuclear-transcribed mRNA catabolic process, deadenylation-dependent decay [GO:1900152];positive regulation of cytoplasmic translation [GO:2000767];positive regulation of DNA-templated transcription [GO:0045893];positive regulation of telomerase RNA reverse transcriptase activity [GO:1905663];positive regulation of telomere capping [GO:1904355];positive regulation of transcription by RNA polymerase II [GO:0045944];positive regulation of translation [GO:0045727];regulation of circadian rhythm [GO:0042752];regulation of DNA-templated transcription [GO:0006355];regulation of gene expression [GO:0010468];regulation of telomere maintenance [GO:0032204];response to calcium ion [GO:0051592];response to electrical stimulus [GO:0051602];response to rapamycin [GO:1901355];response to sodium phosphate [GO:1904383];RNA catabolic process [GO:0006401];RNA processing [GO:0006396] | GO:0001889;GO:0003680;GO:0003682;GO:0003723;GO:0005634;GO:0005654;GO:0005829;GO:0006355;GO:0006396;GO:0006401;GO:0010468;GO:0014069;GO:0021549;GO:0032204;GO:0035925;GO:0042162;GO:0042752;GO:0042789;GO:0042826;GO:0045727;GO:0045893;GO:0045944;GO:0051592;GO:0051602;GO:0061158;GO:0070934;GO:0071230;GO:0071392;GO:0071732;GO:0097167;GO:0098978;GO:0106002;GO:1900152;GO:1901355;GO:1904355;GO:1904383;GO:1904586;GO:1905663;GO:1990828;GO:1990904;GO:2000767 |
| **ENSG00000140988** | **RPS2** | **0.124328135** | **P15880** | **RS2_HUMAN** | **Small ribosomal subunit protein uS5 (40S ribosomal protein S2) (40S ribosomal protein S4) (Protein LLRep3)** | **RPS2 RPS4** | **293** | **FUNCTION: Component of the ribosome, a large ribonucleoprotein complex responsible for the synthesis of proteins in the cell (PubMed:23636399). The small ribosomal subunit (SSU) binds messenger RNAs (mRNAs) and translates the encoded message by selecting cognate aminoacyl-transfer RNA (tRNA) molecules (PubMed:23636399). The large subunit (LSU) contains the ribosomal catalytic site termed the peptidyl transferase center (PTC), which catalyzes the formation of peptide bonds, thereby polymerizing the amino acids delivered by tRNAs into a polypeptide chain (PubMed:23636399). The nascent polypeptides leave the ribosome through a tunnel in the LSU and interact with protein factors that function in enzymatic processing, targeting, and the membrane insertion of nascent chains at the exit of the ribosomal tunnel (PubMed:23636399). Plays a role in the assembly and function of the 40S ribosomal subunit (By similarity). Mutations in this protein affects the control of translational fidelity (By similarity). Involved in nucleolar processing of pre-18S ribosomal RNA and ribosome assembly (By similarity). {ECO:0000250|UniProtKB:P25443, ECO:0000269|PubMed:23636399}.** | **cytoplasmic translation [GO:0002181];positive regulation of ubiquitin-protein transferase activity [GO:0051443];translation [GO:0006412]** | **GO:0002181;GO:0003723;GO:0003729;GO:0003735;GO:0005634;GO:0005654;GO:0005737;GO:0005829;GO:0005925;GO:0006412;GO:0016020;GO:0017134;GO:0019899;GO:0022626;GO:0022627;GO:0045296;GO:0051443;GO:0070062** |
| ENSG00000141759 | TXNL4A | 0.214124805 | P83876 | TXN4A_HUMAN | Thioredoxin-like protein 4A (DIM1 protein homolog) (Spliceosomal U5 snRNP-specific 15 kDa protein) (Thioredoxin-like U5 snRNP protein U5-15kD) | TXNL4A DIM1 TXNL4 | 142 | FUNCTION: Plays a role in pre-mRNA splicing as component of the U5 snRNP and U4/U6-U5 tri-snRNP complexes that are involved in spliceosome assembly, and as component of the precatalytic spliceosome (spliceosome B complex). {ECO:0000269|PubMed:28781166, ECO:0000305|PubMed:10610776, ECO:0000305|PubMed:26912367}. | cell cycle [GO:0007049];cell division [GO:0051301];mRNA splicing, via spliceosome [GO:0000398];RNA splicing, via transesterification reactions [GO:0000375];spliceosomal complex assembly [GO:0000245] | GO:0000245;GO:0000375;GO:0000398;GO:0005634;GO:0005654;GO:0005681;GO:0005682;GO:0005829;GO:0007049;GO:0046540;GO:0051301;GO:0071005 |
| ENSG00000143621 | ILF2 | 0.042644337 | Q12905 | ILF2_HUMAN | Interleukin enhancer-binding factor 2 (Nuclear factor of activated T-cells 45 kDa) | ILF2 NF45 PRO3063 | 390 | FUNCTION: Chromatin-interacting protein that forms a stable heterodimer with interleukin enhancer-binding factor 3/ILF3 and plays a role in several biological processes including transcription, innate immunity or cell growth (PubMed:18458058, PubMed:31212927). Essential for the efficient reshuttling of ILF3 (isoform 1 and isoform 2) into the nucleus. Together with ILF3, forms an RNA-binding complex that is required for mitotic progression and cytokinesis by regulating the expression of a cluster of mitotic genes. Mechanistically, competes with STAU1/STAU2-mediated mRNA decay (PubMed:32433969). Also plays a role in the inhibition of various viruses including Japanese encephalitis virus or enterovirus 71. {ECO:0000269|PubMed:10574923, ECO:0000269|PubMed:11739746, ECO:0000269|PubMed:18458058, ECO:0000269|PubMed:21123651, ECO:0000269|PubMed:31212927, ECO:0000269|PubMed:32433969, ECO:0000269|PubMed:9442054};FUNCTION: (Microbial infection) Plays a positive role in HIV-1 virus production by binding to and thereby stabilizing HIV-1 RNA, together with ILF3. {ECO:0000269|PubMed:26891316}. | positive regulation of DNA-templated transcription [GO:0045893] | GO:0003677;GO:0003723;GO:0003725;GO:0005576;GO:0005634;GO:0005654;GO:0005730;GO:0005829;GO:0016020;GO:0035580;GO:0045893;GO:1904724;GO:1904813;GO:1990904 |
| ENSG00000143977 | SNRPG | 0.310340121 | P62308 | RUXG_HUMAN | Small nuclear ribonucleoprotein G (snRNP-G) (Sm protein G) (Sm-G) (SmG) | SNRPG PBSCG | 76 | FUNCTION: Plays a role in pre-mRNA splicing as a core component of the spliceosomal U1, U2, U4 and U5 small nuclear ribonucleoproteins (snRNPs), the building blocks of the spliceosome (PubMed:11991638, PubMed:18984161, PubMed:19325628, PubMed:23333303, PubMed:25555158, PubMed:26912367, PubMed:28502770, PubMed:28781166, PubMed:28076346). Component of both the pre-catalytic spliceosome B complex and activated spliceosome C complexes (PubMed:11991638, PubMed:28502770, PubMed:28781166, PubMed:28076346). As a component of the minor spliceosome, involved in the splicing of U12-type introns in pre-mRNAs (PubMed:15146077). As part of the U7 snRNP it is involved in histone 3'-end processing (PubMed:12975319). {ECO:0000269|PubMed:11991638, ECO:0000269|PubMed:12975319, ECO:0000269|PubMed:15146077, ECO:0000269|PubMed:18984161, ECO:0000269|PubMed:19325628, ECO:0000269|PubMed:23333303, ECO:0000269|PubMed:25555158, ECO:0000269|PubMed:26912367, ECO:0000269|PubMed:28076346, ECO:0000269|PubMed:28502770, ECO:0000269|PubMed:28781166}. | 7-methylguanosine cap hypermethylation [GO:0036261];mRNA splicing, via spliceosome [GO:0000398];RNA splicing [GO:0008380];spliceosomal complex assembly [GO:0000245];spliceosomal snRNP assembly [GO:0000387];U2-type prespliceosome assembly [GO:1903241] | GO:0000245;GO:0000387;GO:0000398;GO:0003723;GO:0005634;GO:0005654;GO:0005681;GO:0005682;GO:0005683;GO:0005685;GO:0005686;GO:0005687;GO:0005689;GO:0005829;GO:0008380;GO:0030532;GO:0034709;GO:0034719;GO:0036261;GO:0043186;GO:0046540;GO:0071004;GO:0071005;GO:0071007;GO:0071011;GO:0071013;GO:0097526;GO:1903241 |
| ENSG00000145912 | NHP2 | 0.150559677 | Q9NX24 | NHP2_HUMAN | H/ACA ribonucleoprotein complex subunit 2 (Nucleolar protein family A member 2) (snoRNP protein NHP2) | NHP2 NOLA2 HSPC286 | 153 | FUNCTION: Required for ribosome biogenesis and telomere maintenance. Part of the H/ACA small nucleolar ribonucleoprotein (H/ACA snoRNP) complex, which catalyzes pseudouridylation of rRNA. This involves the isomerization of uridine such that the ribose is subsequently attached to C5, instead of the normal N1. Each rRNA can contain up to 100 pseudouridine ('psi') residues, which may serve to stabilize the conformation of rRNAs. May also be required for correct processing or intranuclear trafficking of TERC, the RNA component of the telomerase reverse transcriptase (TERT) holoenzyme. {ECO:0000269|PubMed:15044956}. | maturation of LSU-rRNA [GO:0000470];positive regulation of telomerase RNA localization to Cajal body [GO:1904874];rRNA pseudouridine synthesis [GO:0031118];snRNA pseudouridine synthesis [GO:0031120];telomere maintenance via telomerase [GO:0007004] | GO:0000470;GO:0000781;GO:0003723;GO:0005654;GO:0005697;GO:0005732;GO:0007004;GO:0022625;GO:0031118;GO:0031120;GO:0031429;GO:0034513;GO:0070034;GO:0072589;GO:0090661;GO:1904874 |
| ENSG00000148229 | POLE3 | 0.028569152 | Q9NRF9 | DPOE3_HUMAN | DNA polymerase epsilon subunit 3 (Arsenic-transactivated protein) (AsTP) (Chromatin accessibility complex 17 kDa protein) (CHRAC-17) (HuCHRAC17) (DNA polymerase II subunit 3) (DNA polymerase epsilon subunit p17) | POLE3 CHRAC17 | 147 | FUNCTION: Accessory component of the DNA polymerase epsilon complex (PubMed:10801849). Participates in DNA repair and in chromosomal DNA replication (By similarity). Forms a complex with CHRAC1 and binds naked DNA, which is then incorporated into chromatin, aided by the nucleosome-remodeling activity of ISWI/SNF2H and ACF1 (PubMed:10801849). Does not enhance nucleosome sliding activity of the ACF-5 ISWI chromatin remodeling complex (PubMed:14759371). {ECO:0000250|UniProtKB:Q04603, ECO:0000269|PubMed:10801849, ECO:0000269|PubMed:14759371}. | chromatin remodeling [GO:0006338];DNA damage response [GO:0006974];DNA replication [GO:0006260];DNA-templated DNA replication [GO:0006261];heterochromatin formation [GO:0031507];leading strand elongation [GO:0006272];negative regulation of transcription by RNA polymerase II [GO:0000122];nucleosome assembly [GO:0006334];regulation of DNA replication [GO:0006275] | GO:0000122;GO:0003887;GO:0005634;GO:0005654;GO:0005721;GO:0006260;GO:0006261;GO:0006272;GO:0006275;GO:0006334;GO:0006338;GO:0006974;GO:0008622;GO:0008623;GO:0031490;GO:0031507;GO:0046982;GO:0140672 |
| **ENSG00000149273** | **RPS3** | **0.056583528** | **P23396** | **RS3_HUMAN** | **Small ribosomal subunit protein uS3 (40S ribosomal protein S3) (EC 4.2.99.18)** | **RPS3 OK/SW-cl.26** | **243** | **FUNCTION: Component of the small ribosomal subunit (PubMed:8706699, PubMed:23636399). The ribosome is a large ribonucleoprotein complex responsible for the synthesis of proteins in the cell (PubMed:8706699, PubMed:23636399). Has endonuclease activity and plays a role in repair of damaged DNA (PubMed:7775413). Cleaves phosphodiester bonds of DNAs containing altered bases with broad specificity and cleaves supercoiled DNA more efficiently than relaxed DNA (PubMed:15707971). Displays high binding affinity for 7,8-dihydro-8-oxoguanine (8-oxoG), a common DNA lesion caused by reactive oxygen species (ROS) (PubMed:14706345). Has also been shown to bind with similar affinity to intact and damaged DNA (PubMed:18610840). Stimulates the N-glycosylase activity of the base excision protein OGG1 (PubMed:15518571). Enhances the uracil excision activity of UNG1 (PubMed:18973764). Also stimulates the cleavage of the phosphodiester backbone by APEX1 (PubMed:18973764). When located in the mitochondrion, reduces cellular ROS levels and mitochondrial DNA damage (PubMed:23911537). Has also been shown to negatively regulate DNA repair in cells exposed to hydrogen peroxide (PubMed:17049931). Plays a role in regulating transcription as part of the NF-kappa-B p65-p50 complex where it binds to the RELA/p65 subunit, enhances binding of the complex to DNA and promotes transcription of target genes (PubMed:18045535). Represses its own translation by binding to its cognate mRNA (PubMed:20217897). Binds to and protects TP53/p53 from MDM2-mediated ubiquitination (PubMed:19656744). Involved in spindle formation and chromosome movement during mitosis by regulating microtubule polymerization (PubMed:23131551). Involved in induction of apoptosis through its role in activation of CASP8 (PubMed:14988002). Induces neuronal apoptosis by interacting with the E2F1 transcription factor and acting synergistically with it to up-regulate pro-apoptotic proteins BCL2L11/BIM and HRK/Dp5 (PubMed:20605787). Interacts with TRADD following exposure to UV radiation and induces apoptosis by caspase-dependent JNK activation (PubMed:22510408). {ECO:0000269|PubMed:14706345, ECO:0000269|PubMed:14988002, ECO:0000269|PubMed:15518571, ECO:0000269|PubMed:15707971, ECO:0000269|PubMed:17049931, ECO:0000269|PubMed:18045535, ECO:0000269|PubMed:18610840, ECO:0000269|PubMed:18973764, ECO:0000269|PubMed:19656744, ECO:0000269|PubMed:20217897, ECO:0000269|PubMed:20605787, ECO:0000269|PubMed:22510408, ECO:0000269|PubMed:23131551, ECO:0000269|PubMed:23636399, ECO:0000269|PubMed:23911537, ECO:0000269|PubMed:7775413, ECO:0000269|PubMed:8706699}.** | **apoptotic process [GO:0006915];base-excision repair [GO:0006284];cell division [GO:0051301];cellular response to hydrogen peroxide [GO:0070301];cellular response to reactive oxygen species [GO:0034614];cellular response to tumor necrosis factor [GO:0071356];chromosome segregation [GO:0007059];cytoplasmic translation [GO:0002181];DNA damage response [GO:0006974];DNA repair [GO:0006281];negative regulation of DNA repair [GO:0045738];negative regulation of protein ubiquitination [GO:0031397];negative regulation of translation [GO:0017148];positive regulation of activated T cell proliferation [GO:0042104];positive regulation of apoptotic signaling pathway [GO:2001235];positive regulation of base-excision repair [GO:1905053];positive regulation of cysteine-type endopeptidase activity involved in execution phase of apoptosis [GO:2001272];positive regulation of DNA repair [GO:0045739];positive regulation of endodeoxyribonuclease activity [GO:0032079];positive regulation of gene expression [GO:0010628];positive regulation of interleukin-2 production [GO:0032743];positive regulation of intrinsic apoptotic signaling pathway in response to DNA damage [GO:1902231];positive regulation of JUN kinase activity [GO:0043507];positive regulation of microtubule polymerization [GO:0031116];positive regulation of NF-kappaB transcription factor activity [GO:0051092];positive regulation of non-canonical NF-kappaB signal transduction [GO:1901224];positive regulation of protein-containing complex assembly [GO:0031334];positive regulation of T cell receptor signaling pathway [GO:0050862];regulation of apoptotic process [GO:0042981];response to TNF agonist [GO:0061481];spindle assembly [GO:0051225];translation [GO:0006412];translational initiation [GO:0006413]** | **GO:0002181;GO:0003677;GO:0003684;GO:0003723;GO:0003729;GO:0003735;GO:0003906;GO:0004520;GO:0005634;GO:0005654;GO:0005730;GO:0005737;GO:0005743;GO:0005759;GO:0005783;GO:0005829;GO:0005840;GO:0005844;GO:0005886;GO:0005925;GO:0006281;GO:0006284;GO:0006412;GO:0006413;GO:0006915;GO:0006974;GO:0007059;GO:0008017;GO:0010628;GO:0014069;GO:0015631;GO:0016020;GO:0017148;GO:0019899;GO:0019900;GO:0019901;GO:0022626;GO:0022627;GO:0030544;GO:0031116;GO:0031334;GO:0031397;GO:0032079;GO:0032357;GO:0032358;GO:0032587;GO:0032743;GO:0034614;GO:0042104;GO:0042981;GO:0043507;GO:0044390;GO:0044877;GO:0045738;GO:0045739;GO:0050862;GO:0051018;GO:0051092;GO:0051225;GO:0051301;GO:0051536;GO:0051879;GO:0061481;GO:0070062;GO:0070181;GO:0070301;GO:0071159;GO:0071356;GO:0072686;GO:0097100;GO:0140078;GO:0140297;GO:1901224;GO:1902231;GO:1905053;GO:1990904;GO:2001235;GO:2001272** |
| ENSG00000150753 | CCT5 | 0.176322773 | P48643 | TCPE_HUMAN | T-complex protein 1 subunit epsilon (TCP-1-epsilon) (CCT-epsilon) | CCT5 CCTE KIAA0098 | 541 | FUNCTION: Component of the chaperonin-containing T-complex (TRiC), a molecular chaperone complex that assists the folding of proteins upon ATP hydrolysis (PubMed:25467444). The TRiC complex mediates the folding of WRAP53/TCAB1, thereby regulating telomere maintenance (PubMed:25467444). As part of the TRiC complex may play a role in the assembly of BBSome, a complex involved in ciliogenesis regulating transports vesicles to the cilia (PubMed:20080638). The TRiC complex plays a role in the folding of actin and tubulin (Probable). {ECO:0000269|PubMed:20080638, ECO:0000269|PubMed:25467444, ECO:0000305}. | binding of sperm to zona pellucida [GO:0007339];chaperone-mediated protein folding [GO:0061077];positive regulation of establishment of protein localization to telomere [GO:1904851];positive regulation of protein localization to Cajal body [GO:1904871];positive regulation of telomerase RNA localization to Cajal body [GO:1904874];positive regulation of telomere maintenance via telomerase [GO:0032212];protein folding [GO:0006457];protein stabilization [GO:0050821];response to virus [GO:0009615] | GO:0003730;GO:0005524;GO:0005813;GO:0005829;GO:0005832;GO:0005874;GO:0006457;GO:0007339;GO:0009615;GO:0016887;GO:0031681;GO:0032212;GO:0044183;GO:0044297;GO:0048027;GO:0048487;GO:0050821;GO:0051082;GO:0061077;GO:0070062;GO:0140662;GO:1904851;GO:1904871;GO:1904874 |
| ENSG00000151247 | EIF4E | 0.014355293 | P06730 | IF4E_HUMAN | Eukaryotic translation initiation factor 4E (eIF-4E) (eIF4E) (eIF-4F 25 kDa subunit) (mRNA cap-binding protein) | EIF4E EIF4EL1 EIF4F | 217 | FUNCTION: Acts in the cytoplasm to initiate and regulate protein synthesis and is required in the nucleus for export of a subset of mRNAs from the nucleus to the cytoplasm which promotes processes such as RNA capping, processing and splicing (PubMed:11606200, PubMed:24335285, PubMed:29987188, PubMed:22684010, PubMed:22578813). Component of the protein complex eIF4F, which is involved in the recognition of the mRNA cap, ATP-dependent unwinding of 5'-terminal secondary structure and recruitment of mRNA to the ribosome (By similarity). This protein recognizes and binds the 7-methylguanosine (m7G)-containing mRNA cap during an early step in the initiation of protein synthesis and facilitates ribosome binding by inducing the unwinding of the mRNAs secondary structures (PubMed:16271312, PubMed:22578813). Together with EIF4G1, antagonizes the scanning promoted by EIF1-EIF4G1 and is required for TISU translation, a process where the TISU element recognition makes scanning unnecessary (PubMed:29987188). In addition to its role in translation initiation, also acts as a regulator of translation and stability in the cytoplasm (PubMed:24335285). Component of the CYFIP1-EIF4E-FMR1 complex which binds to the mRNA cap and mediates translational repression: in the complex, EIF4E mediates the binding to the mRNA cap (By similarity). Component of a multiprotein complex that sequesters and represses translation of proneurogenic factors during neurogenesis (By similarity). In P-bodies, component of a complex that mediates the storage of translationally inactive mRNAs in the cytoplasm and prevents their degradation (PubMed:24335285). May play an important role in spermatogenesis through translational regulation of stage-specific mRNAs during germ cell development (By similarity). As well as its roles in translation, also involved in mRNA nucleocytoplasmic transport (By similarity). Its role in mRNA export from the nucleus to the cytoplasm relies on its ability to bind the m7G cap of RNAs and on the presence of the 50-nucleotide EIF4E sensitivity element (4ESE) in the 3'UTR of sensitive transcripts (By similarity). Interaction with the 4ESE is mediated by LRPPRC which binds simultaneously to both EIF4E and the 4ESE, thereby acting as a platform for assembly for the RNA export complex (By similarity). EIF4E-dependent mRNA export is independent of ongoing protein or RNA synthesis and is also NFX1-independent but is XPO1-dependent with LRPPRC interacting with XPO1 to form an EIF4E-dependent mRNA export complex (By similarity). Alters the composition of the cytoplasmic face of the nuclear pore to promote RNA export by reducing RANBP2 expression, relocalizing nucleoporin NUP214 and increasing expression of RANBP1 and RNA export factors DDX19 and GLE1 (By similarity). Promotes the nuclear export of cyclin CCND1 mRNA (By similarity). Promotes the nuclear export of NOS2/iNOS mRNA (PubMed:23471078). Promotes the nuclear export of MDM2 mRNA (PubMed:22684010). Promotes the export of additional mRNAs, including others involved in the cell cycle (By similarity). In the nucleus, binds to capped splice factor-encoding mRNAs and stimulates their nuclear export to enhance splice factor production by increasing their cytoplasmic availability to the translation machinery (By similarity). May also regulate splicing through interaction with the spliceosome in an RNA and m7G cap-dependent manner (By similarity). Also binds to some pre-mRNAs and may play a role in their recruitment to the spliceosome (By similarity). Promotes steady-state capping of a subset of coding and non-coding RNAs by mediating nuclear export of capping machinery mRNAs including RNMT, RNGTT and RAMAC to enhance their translation (By similarity). Stimulates mRNA 3'-end processing by promoting the expression of several core cleavage complex factors required for mRNA cleavage and polyadenylation, and may also have a direct effect through its interaction with the CPSF3 cleavage enzyme (By similarity). Rescues cells from apoptosis by promoting activation of serine/threonine-protein kinase AKT1 through mRNA export of NBS1 which potentiates AKT1 phosphorylation and also through mRNA export of AKT1 effectors, allowing for increased production of these proteins (By similarity). {ECO:0000250|UniProtKB:P63073, ECO:0000250|UniProtKB:P63074, ECO:0000269|PubMed:11606200, ECO:0000269|PubMed:16271312, ECO:0000269|PubMed:22578813, ECO:0000269|PubMed:22684010, ECO:0000269|PubMed:23471078, ECO:0000269|PubMed:24335285, ECO:0000269|PubMed:29987188}. | behavioral fear response [GO:0001662];cellular response to dexamethasone stimulus [GO:0071549];G1/S transition of mitotic cell cycle [GO:0000082];negative regulation of neuron differentiation [GO:0045665];negative regulation of translation [GO:0017148];neuron differentiation [GO:0030182];nuclear export [GO:0051168];positive regulation of mitotic cell cycle [GO:0045931];regulation of translation [GO:0006417];regulation of translation at postsynapse, modulating synaptic transmission [GO:0099578];stem cell population maintenance [GO:0019827];translational initiation [GO:0006413] | GO:0000082;GO:0000339;GO:0000340;GO:0000932;GO:0001662;GO:0003723;GO:0003743;GO:0005634;GO:0005737;GO:0005829;GO:0005845;GO:0006413;GO:0006417;GO:0010494;GO:0016281;GO:0016442;GO:0016607;GO:0017148;GO:0019827;GO:0019899;GO:0030182;GO:0031370;GO:0033391;GO:0036464;GO:0045665;GO:0045931;GO:0048471;GO:0051168;GO:0070062;GO:0071549;GO:0098794;GO:0098978;GO:0099578;GO:0140297 |
| ENSG00000153187 | HNRNPU | 0.176322773 | Q00839 | HNRPU_HUMAN | Heterogeneous nuclear ribonucleoprotein U (hnRNP U) (GRIP120) (Nuclear p120 ribonucleoprotein) (Scaffold-attachment factor A) (SAF-A) (p120) (pp120) | HNRNPU C1orf199 HNRPU SAFA U21.1 | 825 | FUNCTION: DNA- and RNA-binding protein involved in several cellular processes such as nuclear chromatin organization, telomere-length regulation, transcription, mRNA alternative splicing and stability, Xist-mediated transcriptional silencing and mitotic cell progression (PubMed:10490622, PubMed:18082603, PubMed:19029303, PubMed:22325991, PubMed:25986610, PubMed:28622508). Plays a role in the regulation of interphase large-scale gene-rich chromatin organization through chromatin-associated RNAs (caRNAs) in a transcription-dependent manner, and thereby maintains genomic stability (PubMed:1324173, PubMed:8174554, PubMed:28622508). Required for the localization of the long non-coding Xist RNA on the inactive chromosome X (Xi) and the subsequent initiation and maintenance of X-linked transcriptional gene silencing during X-inactivation (By similarity). Plays a role as a RNA polymerase II (Pol II) holoenzyme transcription regulator (PubMed:8174554, PubMed:9353307, PubMed:10490622, PubMed:15711563, PubMed:19617346, PubMed:23811339). Promotes transcription initiation by direct association with the core-TFIIH basal transcription factor complex for the assembly of a functional pre-initiation complex with Pol II in a actin-dependent manner (PubMed:10490622, PubMed:15711563). Blocks Pol II transcription elongation activity by inhibiting the C-terminal domain (CTD) phosphorylation of Pol II and dissociates from Pol II pre-initiation complex prior to productive transcription elongation (PubMed:10490622). Positively regulates CBX5-induced transcriptional gene silencing and retention of CBX5 in the nucleus (PubMed:19617346). Negatively regulates glucocorticoid-mediated transcriptional activation (PubMed:9353307). Key regulator of transcription initiation and elongation in embryonic stem cells upon leukemia inhibitory factor (LIF) signaling (By similarity). Involved in the long non-coding RNA H19-mediated Pol II transcriptional repression (PubMed:23811339). Participates in the circadian regulation of the core clock component BMAL1 transcription (By similarity). Plays a role in the regulation of telomere length (PubMed:18082603). Plays a role as a global pre-mRNA alternative splicing modulator by regulating U2 small nuclear ribonucleoprotein (snRNP) biogenesis (PubMed:22325991). Plays a role in mRNA stability (PubMed:17174306, PubMed:17289661, PubMed:19029303). Component of the CRD-mediated complex that promotes MYC mRNA stabilization (PubMed:19029303). Enhances the expression of specific genes, such as tumor necrosis factor TNFA, by regulating mRNA stability, possibly through binding to the 3'-untranslated region (UTR) (PubMed:17174306). Plays a role in mitotic cell cycle regulation (PubMed:21242313, PubMed:25986610). Involved in the formation of stable mitotic spindle microtubules (MTs) attachment to kinetochore, spindle organization and chromosome congression (PubMed:21242313). Phosphorylation at Ser-59 by PLK1 is required for chromosome alignement and segregation and progression through mitosis (PubMed:25986610). Contributes also to the targeting of AURKA to mitotic spindle MTs (PubMed:21242313). Binds to double- and single-stranded DNA and RNA, poly(A), poly(C) and poly(G) oligoribonucleotides (PubMed:1628625, PubMed:8068679, PubMed:8174554, PubMed:9204873, PubMed:9405365). Binds to chromatin-associated RNAs (caRNAs) (PubMed:28622508). Associates with chromatin to scaffold/matrix attachment region (S/MAR) elements in a chromatin-associated RNAs (caRNAs)-dependent manner (PubMed:7509195, PubMed:1324173, PubMed:9204873, PubMed:9405365, PubMed:10671544, PubMed:11003645, PubMed:11909954, PubMed:28622508). Binds to the Xist RNA (PubMed:26244333). Binds the long non-coding H19 RNA (PubMed:23811339). Binds to SMN1/2 pre-mRNAs at G/U-rich regions (PubMed:22325991). Binds to small nuclear RNAs (snRNAs) (PubMed:22325991). Binds to the 3'-UTR of TNFA mRNA (PubMed:17174306). Binds (via RNA-binding RGG-box region) to the long non-coding Xist RNA;this binding is direct and bridges the Xist RNA and the inactive chromosome X (Xi) (By similarity). Also negatively regulates embryonic stem cell differentiation upon LIF signaling (By similarity). Required for embryonic development (By similarity). Binds to brown fat long non-coding RNA 1 (Blnc1);facilitates the recruitment of Blnc1 by ZBTB7B required to drive brown and beige fat development and thermogenesis (By similarity). {ECO:0000250|UniProtKB:Q8VEK3, ECO:0000269|PubMed:10490622, ECO:0000269|PubMed:10671544, ECO:0000269|PubMed:11003645, ECO:0000269|PubMed:11909954, ECO:0000269|PubMed:1324173, ECO:0000269|PubMed:15711563, ECO:0000269|PubMed:1628625, ECO:0000269|PubMed:17174306, ECO:0000269|PubMed:17289661, ECO:0000269|PubMed:18082603, ECO:0000269|PubMed:19029303, ECO:0000269|PubMed:19617346, ECO:0000269|PubMed:21242313, ECO:0000269|PubMed:22325991, ECO:0000269|PubMed:23811339, ECO:0000269|PubMed:25986610, ECO:0000269|PubMed:26244333, ECO:0000269|PubMed:28622508, ECO:0000269|PubMed:7509195, ECO:0000269|PubMed:8068679, ECO:0000269|PubMed:8174554, ECO:0000269|PubMed:9204873, ECO:0000269|PubMed:9353307, ECO:0000269|PubMed:9405365};FUNCTION: (Microbial infection) Negatively regulates immunodeficiency virus type 1 (HIV-1) replication by preventing the accumulation of viral mRNA transcripts in the cytoplasm. {ECO:0000269|PubMed:16916646}. | adaptive thermogenesis [GO:1990845];cardiac muscle cell development [GO:0055013];cell cycle [GO:0007049];cell division [GO:0051301];cellular response to glucocorticoid stimulus [GO:0071385];cellular response to leukemia inhibitory factor [GO:1990830];chromatin organization [GO:0006325];circadian regulation of gene expression [GO:0032922];CRD-mediated mRNA stabilization [GO:0070934];dendritic transport of messenger ribonucleoprotein complex [GO:0098963];dosage compensation by inactivation of X chromosome [GO:0009048];maintenance of protein location in nucleus [GO:0051457];mRNA splicing, via spliceosome [GO:0000398];mRNA stabilization [GO:0048255];negative regulation of kinase activity [GO:0033673];negative regulation of nuclear-transcribed mRNA catabolic process, deadenylation-dependent decay [GO:1900152];negative regulation of stem cell differentiation [GO:2000737];negative regulation of telomere maintenance via telomerase [GO:0032211];negative regulation of transcription by RNA polymerase II [GO:0000122];negative regulation of transcription elongation by RNA polymerase II [GO:0034244];osteoblast differentiation [GO:0001649];positive regulation of attachment of mitotic spindle microtubules to kinetochore [GO:1902425];positive regulation of brown fat cell differentiation [GO:0090336];positive regulation of cytoplasmic translation [GO:2000767];positive regulation of DNA topoisomerase (ATP-hydrolyzing) activity [GO:2000373];positive regulation of stem cell proliferation [GO:2000648];positive regulation of transcription by RNA polymerase II [GO:0045944];protein localization to spindle microtubule [GO:1902889];regulation of alternative mRNA splicing, via spliceosome [GO:0000381];regulation of chromatin organization [GO:1902275];regulation of mitotic cell cycle [GO:0007346];regulation of mitotic spindle assembly [GO:1901673];RNA localization to chromatin [GO:1990280];RNA processing [GO:0006396] | GO:0000122;GO:0000228;GO:0000381;GO:0000398;GO:0000776;GO:0000922;GO:0000978;GO:0000993;GO:0001097;GO:0001649;GO:0003677;GO:0003682;GO:0003690;GO:0003697;GO:0003714;GO:0003723;GO:0003725;GO:0003727;GO:0003730;GO:0003779;GO:0005524;GO:0005634;GO:0005654;GO:0005697;GO:0005813;GO:0005829;GO:0006325;GO:0006396;GO:0007049;GO:0007346;GO:0008143;GO:0009048;GO:0009986;GO:0016020;GO:0016363;GO:0016607;GO:0017069;GO:0017130;GO:0030496;GO:0031490;GO:0032211;GO:0032839;GO:0032922;GO:0032991;GO:0033673;GO:0034046;GO:0034244;GO:0036002;GO:0036464;GO:0042802;GO:0043021;GO:0044877;GO:0045944;GO:0048255;GO:0051301;GO:0051457;GO:0055013;GO:0070034;GO:0070934;GO:0070937;GO:0071013;GO:0071385;GO:0072686;GO:0090336;GO:0098577;GO:0098963;GO:0099122;GO:0106222;GO:1900152;GO:1901673;GO:1902275;GO:1902425;GO:1902889;GO:1990023;GO:1990280;GO:1990498;GO:1990830;GO:1990837;GO:1990841;GO:1990845;GO:1990904;GO:2000373;GO:2000648;GO:2000737;GO:2000767 |
| ENSG00000154174 | TOMM70 | 0.111031312 | O94826 | TOM70_HUMAN | Mitochondrial import receptor subunit TOM70 (Mitochondrial precursor proteins import receptor) (Translocase of outer membrane 70 kDa subunit) (Translocase of outer mitochondrial membrane protein 70) | TOMM70 KIAA0719 TOM70 TOMM70A | 608 | FUNCTION: Acts as receptor of the preprotein translocase complex of the outer mitochondrial membrane (TOM complex) (PubMed:12526792). Recognizes and mediates the translocation of mitochondrial preproteins from the cytosol into the mitochondria in a chaperone dependent manner (PubMed:12526792, PubMed:35025629). Mediates TBK1 and IRF3 activation induced by MAVS in response to Sendai virus infection and promotes host antiviral responses during virus infection (PubMed:20628368, PubMed:25609812, PubMed:32728199). Upon Sendai virus infection, recruits HSP90AA1:IRF3:BAX in mitochondrion and the complex induces apoptosis (PubMed:25609812). {ECO:0000269|PubMed:12526792, ECO:0000269|PubMed:20628368, ECO:0000269|PubMed:25609812, ECO:0000269|PubMed:32728199, ECO:0000269|PubMed:35025629}. | activation of innate immune response [GO:0002218];cellular response to virus [GO:0098586];negative regulation of cell growth involved in cardiac muscle cell development [GO:0061052];positive regulation of defense response to virus by host [GO:0002230];positive regulation of interferon-beta production [GO:0032728];positive regulation of protein targeting to mitochondrion [GO:1903955];protein import into mitochondrial matrix [GO:0030150];protein insertion into mitochondrial inner membrane [GO:0045039];protein insertion into mitochondrial outer membrane [GO:0045040];protein targeting to mitochondrion [GO:0006626];regulation of apoptotic process [GO:0042981];response to thyroxine [GO:0097068] | GO:0002218;GO:0002230;GO:0005739;GO:0005741;GO:0005742;GO:0006626;GO:0008320;GO:0016020;GO:0030150;GO:0030943;GO:0031966;GO:0032728;GO:0042981;GO:0045039;GO:0045040;GO:0060090;GO:0061052;GO:0070062;GO:0097068;GO:0098586;GO:1903955 |
| ENSG00000154473 | BUB3 | 0.344828497 | O43684 | BUB3_HUMAN | Mitotic checkpoint protein BUB3 | BUB3 | 328 | FUNCTION: Has a dual function in spindle-assembly checkpoint signaling and in promoting the establishment of correct kinetochore-microtubule (K-MT) attachments. Promotes the formation of stable end-on bipolar attachments. Necessary for kinetochore localization of BUB1. Regulates chromosome segregation during oocyte meiosis. The BUB1/BUB3 complex plays a role in the inhibition of anaphase-promoting complex or cyclosome (APC/C) when spindle-assembly checkpoint is activated and inhibits the ubiquitin ligase activity of APC/C by phosphorylating its activator CDC20. This complex can also phosphorylate MAD1L1. {ECO:0000269|PubMed:10198256, ECO:0000269|PubMed:15525512, ECO:0000269|PubMed:18199686}. | attachment of spindle microtubules to kinetochore [GO:0008608];cell division [GO:0051301];meiotic cell cycle [GO:0051321];mitotic spindle assembly checkpoint signaling [GO:0007094];protein localization to kinetochore [GO:0034501] | GO:0000776;GO:0005654;GO:0005829;GO:0007094;GO:0008608;GO:0033597;GO:0034501;GO:0043130;GO:0051301;GO:0051321;GO:1990298 |
| ENSG00000155959 | VBP1 | 0 | P61758 | PFD3_HUMAN | Prefoldin subunit 3 (HIBBJ46) (von Hippel-Lindau-binding protein 1) (VBP-1) (VHL-binding protein 1) | VBP1 PFDN3 | 197 | FUNCTION: Binds specifically to cytosolic chaperonin (c-CPN) and transfers target proteins to it. Binds to nascent polypeptide chain and promotes folding in an environment in which there are many competing pathways for nonnative proteins. {ECO:0000269|PubMed:9630229}. | chaperone-mediated protein folding [GO:0061077];microtubule-based process [GO:0007017];negative regulation of amyloid fibril formation [GO:1905907];protein folding [GO:0006457];tubulin complex assembly [GO:0007021] | GO:0001540;GO:0005634;GO:0005737;GO:0005829;GO:0005844;GO:0006457;GO:0007017;GO:0007021;GO:0015631;GO:0016272;GO:0043231;GO:0051082;GO:0061077;GO:1905907 |
| ENSG00000161547 | SRSF2 | 0.321928095 | Q01130 | SRSF2_HUMAN | Serine/arginine-rich splicing factor 2 (Protein PR264) (Splicing component, 35 kDa) (Splicing factor SC35) (SC-35) (Splicing factor, arginine/serine-rich 2) | SRSF2 SFRS2 | 221 | FUNCTION: Necessary for the splicing of pre-mRNA. It is required for formation of the earliest ATP-dependent splicing complex and interacts with spliceosomal components bound to both the 5'- and 3'-splice sites during spliceosome assembly. It also is required for ATP-dependent interactions of both U1 and U2 snRNPs with pre-mRNA. Interacts with other spliceosomal components, via the RS domains, to form a bridge between the 5'- and 3'-splice site binding components, U1 snRNP and U2AF. Binds to purine-rich RNA sequences, either 5'-AGSAGAGTA-3' (S=C or G) or 5'-GTTCGAGTA-3'. Can bind to beta-globin mRNA and commit it to the splicing pathway. The phosphorylated form (by SRPK2) is required for cellular apoptosis in response to cisplatin treatment. {ECO:0000269|PubMed:19592491, ECO:0000269|PubMed:21157427}. | mRNA processing [GO:0006397];mRNA splicing, via spliceosome [GO:0000398];RNA splicing [GO:0008380] | GO:0000398;GO:0003714;GO:0003723;GO:0005634;GO:0005654;GO:0005829;GO:0006397;GO:0008380;GO:0016607 |
| ENSG00000163468 | CCT3 | 0.263034406 | P49368 | TCPG_HUMAN | T-complex protein 1 subunit gamma (TCP-1-gamma) (CCT-gamma) (hTRiC5) | CCT3 CCTG TRIC5 | 545 | FUNCTION: Component of the chaperonin-containing T-complex (TRiC), a molecular chaperone complex that assists the folding of proteins upon ATP hydrolysis (PubMed:25467444). The TRiC complex mediates the folding of WRAP53/TCAB1, thereby regulating telomere maintenance (PubMed:25467444). As part of the TRiC complex may play a role in the assembly of BBSome, a complex involved in ciliogenesis regulating transports vesicles to the cilia (PubMed:20080638). The TRiC complex plays a role in the folding of actin and tubulin (Probable). {ECO:0000269|PubMed:20080638, ECO:0000269|PubMed:25467444, ECO:0000305}. | binding of sperm to zona pellucida [GO:0007339];chaperone-mediated protein folding [GO:0061077];positive regulation of establishment of protein localization to telomere [GO:1904851];positive regulation of protein localization to Cajal body [GO:1904871];positive regulation of telomerase RNA localization to Cajal body [GO:1904874];positive regulation of telomere maintenance via telomerase [GO:0032212];protein folding [GO:0006457];protein stabilization [GO:0050821] | GO:0002199;GO:0003723;GO:0005524;GO:0005829;GO:0005832;GO:0005856;GO:0005874;GO:0006457;GO:0007339;GO:0016887;GO:0032212;GO:0044183;GO:0044297;GO:0050821;GO:0051082;GO:0061077;GO:0070062;GO:0140662;GO:1904851;GO:1904871;GO:1904874 |
| ENSG00000164032 | H2AZ1 | 0.189033824 | P0C0S5 | H2AZ_HUMAN | Histone H2A.Z (H2A/z) | H2AZ1 H2AFZ H2AZ | 128 | FUNCTION: Variant histone H2A which replaces conventional H2A in a subset of nucleosomes. Nucleosomes wrap and compact DNA into chromatin, limiting DNA accessibility to the cellular machineries which require DNA as a template. Histones thereby play a central role in transcription regulation, DNA repair, DNA replication and chromosomal stability. DNA accessibility is regulated via a complex set of post-translational modifications of histones, also called histone code, and nucleosome remodeling. May be involved in the formation of constitutive heterochromatin. May be required for chromosome segregation during cell division. {ECO:0000269|PubMed:15878876}. | cellular response to estradiol stimulus [GO:0071392];chromatin organization [GO:0006325];positive regulation of transcription by RNA polymerase II [GO:0045944] | GO:0000786;GO:0000791;GO:0000792;GO:0000978;GO:0000979;GO:0003677;GO:0005634;GO:0006325;GO:0030527;GO:0031490;GO:0031492;GO:0045944;GO:0046982;GO:0070062;GO:0071392 |
| ENSG00000165704 | HPRT1 | 0.111031312 | P00492 | HPRT_HUMAN | Hypoxanthine-guanine phosphoribosyltransferase (HGPRT) (HGPRTase) (EC 2.4.2.8) | HPRT1 HPRT | 218 | FUNCTION: Converts guanine to guanosine monophosphate, and hypoxanthine to inosine monophosphate. Transfers the 5-phosphoribosyl group from 5-phosphoribosylpyrophosphate onto the purine. Plays a central role in the generation of purine nucleotides through the purine salvage pathway. | adenine metabolic process [GO:0046083];AMP salvage [GO:0044209];central nervous system neuron development [GO:0021954];cerebral cortex neuron differentiation [GO:0021895];dendrite morphogenesis [GO:0048813];dopamine metabolic process [GO:0042417];dopaminergic neuron differentiation [GO:0071542];GMP catabolic process [GO:0046038];GMP salvage [GO:0032263];grooming behavior [GO:0007625];guanine salvage [GO:0006178];hypoxanthine metabolic process [GO:0046100];hypoxanthine salvage [GO:0043103];IMP metabolic process [GO:0046040];IMP salvage [GO:0032264];locomotory behavior [GO:0007626];lymphocyte proliferation [GO:0046651];positive regulation of dopamine metabolic process [GO:0045964];protein homotetramerization [GO:0051289];purine nucleotide biosynthetic process [GO:0006164];purine ribonucleoside salvage [GO:0006166];response to amphetamine [GO:0001975];striatum development [GO:0021756];T cell mediated cytotoxicity [GO:0001913] | GO:0000166;GO:0000287;GO:0001913;GO:0001975;GO:0004422;GO:0005737;GO:0005829;GO:0006164;GO:0006166;GO:0006178;GO:0007625;GO:0007626;GO:0021756;GO:0021895;GO:0021954;GO:0032263;GO:0032264;GO:0042417;GO:0042802;GO:0043103;GO:0044209;GO:0045964;GO:0046038;GO:0046040;GO:0046083;GO:0046100;GO:0046651;GO:0048813;GO:0051289;GO:0052657;GO:0070062;GO:0071542 |
| ENSG00000165732 | DDX21 | 0.454175893 | Q9NR30 | DDX21_HUMAN | Nucleolar RNA helicase 2 (EC 3.6.4.13) (DEAD box protein 21) (Gu-alpha) (Nucleolar RNA helicase Gu) (Nucleolar RNA helicase II) (RH II/Gu) | DDX21 | 783 | FUNCTION: RNA helicase that acts as a sensor of the transcriptional status of both RNA polymerase (Pol) I and II: promotes ribosomal RNA (rRNA) processing and transcription from polymerase II (Pol II) (PubMed:25470060, PubMed:28790157). Binds various RNAs, such as rRNAs, snoRNAs, 7SK and, at lower extent, mRNAs (PubMed:25470060). In the nucleolus, localizes to rDNA locus, where it directly binds rRNAs and snoRNAs, and promotes rRNA transcription, processing and modification. Required for rRNA 2'-O-methylation, possibly by promoting the recruitment of late-acting snoRNAs SNORD56 and SNORD58 with pre-ribosomal complexes (PubMed:25470060, PubMed:25477391). In the nucleoplasm, binds 7SK RNA and is recruited to the promoters of Pol II-transcribed genes: acts by facilitating the release of P-TEFb from inhibitory 7SK snRNP in a manner that is dependent on its helicase activity, thereby promoting transcription of its target genes (PubMed:25470060). Functions as cofactor for JUN-activated transcription: required for phosphorylation of JUN at 'Ser-77' (PubMed:11823437, PubMed:25260534). Can unwind double-stranded RNA (helicase) and can fold or introduce a secondary structure to a single-stranded RNA (foldase) (PubMed:9461305). Together with SIRT7, required to prevent R-loop-associated DNA damage and transcription-associated genomic instability: deacetylation by SIRT7 activates the helicase activity, thereby overcoming R-loop-mediated stalling of RNA polymerases (PubMed:28790157). Involved in rRNA processing (PubMed:14559904, PubMed:18180292). May bind to specific miRNA hairpins (PubMed:28431233). Component of a multi-helicase-TICAM1 complex that acts as a cytoplasmic sensor of viral double-stranded RNA (dsRNA) and plays a role in the activation of a cascade of antiviral responses including the induction of pro-inflammatory cytokines via the adapter molecule TICAM1 (By similarity). {ECO:0000250|UniProtKB:Q9JIK5, ECO:0000269|PubMed:11823437, ECO:0000269|PubMed:14559904, ECO:0000269|PubMed:18180292, ECO:0000269|PubMed:25260534, ECO:0000269|PubMed:25470060, ECO:0000269|PubMed:25477391, ECO:0000269|PubMed:28431233, ECO:0000269|PubMed:28790157, ECO:0000269|PubMed:9461305}. | chromatin remodeling [GO:0006338];defense response to virus [GO:0051607];innate immune response [GO:0045087];negative regulation of transcription by RNA polymerase I [GO:0016479];osteoblast differentiation [GO:0001649];positive regulation of canonical NF-kappaB signal transduction [GO:0043123];positive regulation of histone acetylation [GO:0035066];positive regulation of myeloid dendritic cell cytokine production [GO:0002735];positive regulation of transcription by RNA polymerase I [GO:0045943];positive regulation of transcription by RNA polymerase II [GO:0045944];positive regulation of transcription by RNA polymerase III [GO:0045945];R-loop processing [GO:0062176];response to exogenous dsRNA [GO:0043330];rRNA processing [GO:0006364];transcription by RNA polymerase II [GO:0006366] | GO:0001649;GO:0002735;GO:0003723;GO:0003724;GO:0003725;GO:0005524;GO:0005654;GO:0005694;GO:0005730;GO:0005739;GO:0005829;GO:0006338;GO:0006364;GO:0006366;GO:0016020;GO:0016479;GO:0016887;GO:0019843;GO:0030515;GO:0035066;GO:0035198;GO:0042802;GO:0043123;GO:0043330;GO:0045087;GO:0045943;GO:0045944;GO:0045945;GO:0051607;GO:0062176;GO:0097322;GO:0110016;GO:0140870 |
| ENSG00000166197 | NOLC1 | 0.23878686 | Q14978 | NOLC1_HUMAN | Nucleolar and coiled-body phosphoprotein 1 (140 kDa nucleolar phosphoprotein) (Nopp140) (Hepatitis C virus NS5A-transactivated protein 13) (HCV NS5A-transactivated protein 13) (Nucleolar 130 kDa protein) (Nucleolar phosphoprotein p130) | NOLC1 KIAA0035 NS5ATP13 | 699 | FUNCTION: Nucleolar protein that acts as a regulator of RNA polymerase I by connecting RNA polymerase I with enzymes responsible for ribosomal processing and modification (PubMed:10567578, PubMed:26399832). Required for neural crest specification: following monoubiquitination by the BCR(KBTBD8) complex, associates with TCOF1 and acts as a platform to connect RNA polymerase I with enzymes responsible for ribosomal processing and modification, leading to remodel the translational program of differentiating cells in favor of neural crest specification (PubMed:26399832). Involved in nucleologenesis, possibly by playing a role in the maintenance of the fundamental structure of the fibrillar center and dense fibrillar component in the nucleolus (PubMed:9016786). It has intrinsic GTPase and ATPase activities (PubMed:9016786). {ECO:0000269|PubMed:10567578, ECO:0000269|PubMed:26399832, ECO:0000269|PubMed:9016786}. | mitotic cell cycle [GO:0000278];neural crest cell development [GO:0014032];neural crest formation [GO:0014029];nucleolus organization [GO:0007000];regulation of translation [GO:0006417];rRNA processing [GO:0006364] | GO:0000278;GO:0001650;GO:0003723;GO:0005524;GO:0005525;GO:0005654;GO:0005730;GO:0005737;GO:0006364;GO:0006417;GO:0007000;GO:0014029;GO:0014032;GO:0015030;GO:0030674;GO:0046982;GO:0140678 |
| ENSG00000167088 | SNRPD1 | 0.137503524 | P62314 | SMD1_HUMAN | Small nuclear ribonucleoprotein Sm D1 (Sm-D1) (Sm-D autoantigen) (snRNP core protein D1) | SNRPD1 | 119 | FUNCTION: Plays a role in pre-mRNA splicing as a core component of the spliceosomal U1, U2, U4 and U5 small nuclear ribonucleoproteins (snRNPs), the building blocks of the spliceosome (PubMed:11991638, PubMed:18984161, PubMed:19325628, PubMed:23333303, PubMed:25555158, PubMed:26912367, PubMed:28502770, PubMed:28781166, PubMed:28076346). Component of both the pre-catalytic spliceosome B complex and activated spliceosome C complexes (PubMed:11991638, PubMed:26912367, PubMed:28502770, PubMed:28781166, PubMed:28076346). As a component of the minor spliceosome, involved in the splicing of U12-type introns in pre-mRNAs (PubMed:15146077). May act as a charged protein scaffold to promote snRNP assembly or strengthen snRNP-snRNP interactions through non-specific electrostatic contacts with RNA (Probable). {ECO:0000269|PubMed:11991638, ECO:0000269|PubMed:15146077, ECO:0000269|PubMed:18984161, ECO:0000269|PubMed:19325628, ECO:0000269|PubMed:23333303, ECO:0000269|PubMed:25555158, ECO:0000269|PubMed:26912367, ECO:0000269|PubMed:28076346, ECO:0000269|PubMed:28502770, ECO:0000269|PubMed:28781166, ECO:0000305|PubMed:23333303}. | 7-methylguanosine cap hypermethylation [GO:0036261];mRNA splicing, via spliceosome [GO:0000398];RNA splicing [GO:0008380];spliceosomal complex assembly [GO:0000245];spliceosomal snRNP assembly [GO:0000387];U2-type prespliceosome assembly [GO:1903241] | GO:0000243;GO:0000245;GO:0000387;GO:0000398;GO:0003723;GO:0005634;GO:0005654;GO:0005681;GO:0005682;GO:0005685;GO:0005686;GO:0005687;GO:0005689;GO:0005829;GO:0008380;GO:0030532;GO:0034709;GO:0034715;GO:0034719;GO:0036261;GO:0046540;GO:0071005;GO:0071007;GO:0071011;GO:0071013;GO:0097526;GO:1903241 |
| ENSG00000169564 | PCBP1 | 0.137503524 | Q15365 | PCBP1_HUMAN | Poly(rC)-binding protein 1 (Alpha-CP1) (Heterogeneous nuclear ribonucleoprotein E1) (hnRNP E1) (Nucleic acid-binding protein SUB2.3) | PCBP1 | 356 | FUNCTION: Single-stranded nucleic acid binding protein that binds preferentially to oligo dC (PubMed:7607214, PubMed:7556077, PubMed:8152927, PubMed:15731341). Together with PCBP2, required for erythropoiesis, possibly by regulating mRNA splicing (By similarity). {ECO:0000250|UniProtKB:P60335, ECO:0000269|PubMed:15731341, ECO:0000269|PubMed:7556077, ECO:0000269|PubMed:7607214, ECO:0000269|PubMed:8152927};FUNCTION: (Microbial infection) In case of infection by poliovirus, plays a role in initiation of viral RNA replication in concert with the viral protein 3CD. {ECO:0000269|PubMed:12414943}. | positive regulation of transcription by RNA polymerase II [GO:0045944];viral RNA genome replication [GO:0039694] | GO:0000981;GO:0003697;GO:0003723;GO:0003729;GO:0005634;GO:0005654;GO:0005737;GO:0005829;GO:0014069;GO:0016020;GO:0016607;GO:0036464;GO:0039694;GO:0045296;GO:0045944;GO:0070062;GO:0098847;GO:1990904 |
| ENSG00000169714 | CNBP | 0.084064265 | P62633 | CNBP_HUMAN | CCHC-type zinc finger nucleic acid binding protein (Cellular nucleic acid-binding protein) (CNBP) (Zinc finger protein 9) | CNBP RNF163 ZNF9 | 177 | FUNCTION: Single-stranded DNA-binding protein that preferentially binds to the sterol regulatory element (SRE) sequence 5'-GTGCGGTG-3', and thereby mediates transcriptional repression (PubMed:2562787). Has a role as transactivator of the Myc promoter (By similarity). Binds single-stranded RNA in a sequence-specific manner (By similarity). {ECO:0000250|UniProtKB:P53996, ECO:0000250|UniProtKB:P62634, ECO:0000269|PubMed:2562787};FUNCTION: [Isoform 1]: Binds G-rich elements in target mRNA coding sequences (PubMed:28329689). Prevents G-quadruplex structure formation in vitro, suggesting a role in supporting translation by resolving stable structures on mRNAs (PubMed:28329689). {ECO:0000269|PubMed:28329689};FUNCTION: [Isoform 2]: Binds to RNA. {ECO:0000269|PubMed:28329689};FUNCTION: [Isoform 4]: Binds to RNA. {ECO:0000269|PubMed:28329689};FUNCTION: [Isoform 5]: Binds to RNA. {ECO:0000269|PubMed:28329689};FUNCTION: [Isoform 6]: Binds to RNA. {ECO:0000269|PubMed:28329689};FUNCTION: [Isoform 8]: Binds to RNA. {ECO:0000269|PubMed:28329689}. | cholesterol homeostasis [GO:0042632];G-quadruplex DNA formation [GO:0071919];negative regulation of transcription by RNA polymerase II [GO:0000122];positive regulation of cell population proliferation [GO:0008284];positive regulation of cytoplasmic translation [GO:2000767];positive regulation of DNA-templated transcription [GO:0045893];positive regulation of transcription by RNA polymerase II [GO:0045944];regulation of DNA-templated transcription [GO:0006355] | GO:0000122;GO:0003723;GO:0003727;GO:0003729;GO:0005737;GO:0005783;GO:0005829;GO:0006355;GO:0008270;GO:0008284;GO:0042632;GO:0045182;GO:0045893;GO:0045944;GO:0051880;GO:0071919;GO:2000767 |
| ENSG00000170142 | UBE2E1 | 0.189033824 | P51965 | UB2E1_HUMAN | Ubiquitin-conjugating enzyme E2 E1 (EC 2.3.2.23) ((E3-independent) E2 ubiquitin-conjugating enzyme E1) (EC 2.3.2.24) (E2 ubiquitin-conjugating enzyme E1) (UbcH6) (Ubiquitin carrier protein E1) (Ubiquitin-protein ligase E1) | UBE2E1 UBCH6 | 193 | FUNCTION: Accepts ubiquitin from the E1 complex and catalyzes its covalent attachment to other proteins. Catalyzes the covalent attachment of ISG15 to other proteins. Mediates the selective degradation of short-lived and abnormal proteins. In vitro also catalyzes 'Lys-48'-linked polyubiquitination. {ECO:0000269|PubMed:16428300, ECO:0000269|PubMed:20061386}. | ISG15-protein conjugation [GO:0032020];positive regulation of transcription by RNA polymerase II [GO:0045944];protein K48-linked ubiquitination [GO:0070936];protein polyubiquitination [GO:0000209];protein ubiquitination [GO:0016567];ubiquitin-dependent protein catabolic process [GO:0006511] | GO:0000151;GO:0000209;GO:0004842;GO:0005524;GO:0005634;GO:0005654;GO:0005829;GO:0006511;GO:0016567;GO:0032020;GO:0042296;GO:0045944;GO:0061631;GO:0070936 |
| ENSG00000170144 | HNRNPA3 | 0.163498732 | P51991 | ROA3_HUMAN | Heterogeneous nuclear ribonucleoprotein A3 (hnRNP A3) | HNRNPA3 HNRPA3 | 378 | FUNCTION: Plays a role in cytoplasmic trafficking of RNA. Binds to the cis-acting response element, A2RE. May be involved in pre-mRNA splicing. {ECO:0000269|PubMed:11886857}. | mRNA splicing, via spliceosome [GO:0000398] | GO:0000398;GO:0003723;GO:0003730;GO:0005634;GO:0005654;GO:0071013;GO:1990904 |
| ENSG00000170515 | PA2G4 | 0.042644337 | Q9UQ80 | PA2G4_HUMAN | Proliferation-associated protein 2G4 (Cell cycle protein p38-2G4 homolog) (hG4-1) (ErbB3-binding protein 1) | PA2G4 EBP1 | 394 | FUNCTION: May play a role in a ERBB3-regulated signal transduction pathway. Seems be involved in growth regulation. Acts a corepressor of the androgen receptor (AR) and is regulated by the ERBB3 ligand neuregulin-1/heregulin (HRG). Inhibits transcription of some E2F1-regulated promoters, probably by recruiting histone acetylase (HAT) activity. Binds RNA. Associates with 28S, 18S and 5.8S mature rRNAs, several rRNA precursors and probably U3 small nucleolar RNA. May be involved in regulation of intermediate and late steps of rRNA processing. May be involved in ribosome assembly. Mediates cap-independent translation of specific viral IRESs (internal ribosomal entry site) (By similarity). Regulates cell proliferation, differentiation, and survival. Isoform 1 suppresses apoptosis whereas isoform 2 promotes cell differentiation (By similarity). {ECO:0000250|UniProtKB:P50580, ECO:0000250|UniProtKB:Q6AYD3, ECO:0000269|PubMed:11268000, ECO:0000269|PubMed:12682367, ECO:0000269|PubMed:15064750, ECO:0000269|PubMed:15583694, ECO:0000269|PubMed:16832058}. | negative regulation of apoptotic process [GO:0043066];negative regulation of DNA-templated transcription [GO:0045892];positive regulation of cell differentiation [GO:0045597];regulation of translation [GO:0006417];rRNA processing [GO:0006364] | GO:0003676;GO:0003714;GO:0003723;GO:0005576;GO:0005634;GO:0005730;GO:0005737;GO:0006364;GO:0006417;GO:0016020;GO:0031625;GO:0035578;GO:0043066;GO:0045597;GO:0045892;GO:0070062;GO:1990904 |
| ENSG00000171490 | RSL1D1 | 0.014355293 | O76021 | RL1D1_HUMAN | Ribosomal L1 domain-containing protein 1 (CATX-11) (Cellular senescence-inhibited gene protein) (Protein PBK1) | RSL1D1 CATX11 CSIG PBK1 L12 | 490 | FUNCTION: Regulates cellular senescence through inhibition of PTEN translation. Acts as a pro-apoptotic regulator in response to DNA damage. {ECO:0000269|PubMed:18678645, ECO:0000269|PubMed:22419112}. | maturation of LSU-rRNA [GO:0000470];osteoblast differentiation [GO:0001649];regulation of apoptotic process [GO:0042981];regulation of cellular senescence [GO:2000772];regulation of protein localization [GO:0032880] | GO:0000470;GO:0001649;GO:0003723;GO:0003730;GO:0005694;GO:0005730;GO:0016020;GO:0022625;GO:0030686;GO:0032880;GO:0042981;GO:0045296;GO:0048027;GO:2000772 |
| ENSG00000173473 | SMARCC1 | 0.422233001 | Q92922 | SMRC1_HUMAN | SWI/SNF complex subunit SMARCC1 (BRG1-associated factor 155) (BAF155) (SWI/SNF complex 155 kDa subunit) (SWI/SNF-related matrix-associated actin-dependent regulator of chromatin subfamily C member 1) | SMARCC1 BAF155 | 1105 | FUNCTION: Involved in transcriptional activation and repression of select genes by chromatin remodeling (alteration of DNA-nucleosome topology). Component of SWI/SNF chromatin remodeling complexes that carry out key enzymatic activities, changing chromatin structure by altering DNA-histone contacts within a nucleosome in an ATP-dependent manner. May stimulate the ATPase activity of the catalytic subunit of the complex (PubMed:10078207, PubMed:29374058). Belongs to the neural progenitors-specific chromatin remodeling complex (npBAF complex) and the neuron-specific chromatin remodeling complex (nBAF complex). During neural development a switch from a stem/progenitor to a postmitotic chromatin remodeling mechanism occurs as neurons exit the cell cycle and become committed to their adult state. The transition from proliferating neural stem/progenitor cells to postmitotic neurons requires a switch in subunit composition of the npBAF and nBAF complexes. As neural progenitors exit mitosis and differentiate into neurons, npBAF complexes which contain ACTL6A/BAF53A and PHF10/BAF45A, are exchanged for homologous alternative ACTL6B/BAF53B and DPF1/BAF45B or DPF3/BAF45C subunits in neuron-specific complexes (nBAF). The npBAF complex is essential for the self-renewal/proliferative capacity of the multipotent neural stem cells. The nBAF complex along with CREST plays a role regulating the activity of genes essential for dendrite growth (By similarity). {ECO:0000250|UniProtKB:P97496, ECO:0000269|PubMed:10078207, ECO:0000269|PubMed:11018012, ECO:0000269|PubMed:29374058, ECO:0000303|PubMed:22952240, ECO:0000303|PubMed:26601204}. | animal organ morphogenesis [GO:0009887];chromatin remodeling [GO:0006338];insulin receptor signaling pathway [GO:0008286];negative regulation of cell differentiation [GO:0045596];negative regulation of proteasomal ubiquitin-dependent protein catabolic process [GO:0032435];nervous system development [GO:0007399];nucleosome disassembly [GO:0006337];positive regulation of cell differentiation [GO:0045597];positive regulation of cell population proliferation [GO:0008284];positive regulation of DNA-templated transcription [GO:0045893];positive regulation of double-strand break repair [GO:2000781];positive regulation of myoblast differentiation [GO:0045663];positive regulation of stem cell population maintenance [GO:1902459];positive regulation of T cell differentiation [GO:0045582];positive regulation of transcription by RNA polymerase II [GO:0045944];prostate gland development [GO:0030850];regulation of G0 to G1 transition [GO:0070316];regulation of G1/S transition of mitotic cell cycle [GO:2000045];regulation of mitotic metaphase/anaphase transition [GO:0030071];regulation of nucleotide-excision repair [GO:2000819];regulation of transcription by RNA polymerase II [GO:0006357] | GO:0000776;GO:0000785;GO:0001673;GO:0001741;GO:0003682;GO:0003713;GO:0005634;GO:0005654;GO:0005737;GO:0006337;GO:0006338;GO:0006357;GO:0007399;GO:0008284;GO:0008286;GO:0009887;GO:0016363;GO:0016514;GO:0016586;GO:0030071;GO:0030850;GO:0032435;GO:0032991;GO:0035060;GO:0042393;GO:0045582;GO:0045596;GO:0045597;GO:0045663;GO:0045893;GO:0045944;GO:0070316;GO:0071564;GO:0071565;GO:0140288;GO:1902459;GO:2000045;GO:2000781;GO:2000819 |
| ENSG00000173674 | EIF1AX | 0.056583528 | P47813 | IF1AX_HUMAN | Eukaryotic translation initiation factor 1A, X-chromosomal (eIF-1A X isoform) (eIF1A X isoform) (Eukaryotic translation initiation factor 4C) (eIF-4C) | EIF1AX EIF1A EIF4C | 144 | FUNCTION: Component of the 43S pre-initiation complex (43S PIC), which binds to the mRNA cap-proximal region, scans mRNA 5'-untranslated region, and locates the initiation codon (PubMed:9732867). This protein enhances formation of the cap-proximal complex (PubMed:9732867). Together with EIF1, facilitates scanning, start codon recognition, promotion of the assembly of 48S complex at the initiation codon (43S PIC becomes 48S PIC after the start codon is reached), and dissociation of aberrant complexes (PubMed:9732867). After start codon location, together with EIF5B orients the initiator methionine-tRNA in a conformation that allows 60S ribosomal subunit joining to form the 80S initiation complex (PubMed:35732735). Is released after 80S initiation complex formation, just after GTP hydrolysis by EIF5B, and before release of EIF5B (PubMed:35732735). Its globular part is located in the A site of the 40S ribosomal subunit (PubMed:35732735). Its interaction with EIF5 during scanning contribute to the maintenance of EIF1 within the open 43S PIC (PubMed:24319994). In contrast to yeast orthologs, does not bind EIF1 (PubMed:24319994). {ECO:0000269|PubMed:24319994, ECO:0000269|PubMed:35732735, ECO:0000269|PubMed:9732867}. | ribosome assembly [GO:0042255];translational initiation [GO:0006413] | GO:0000049;GO:0003723;GO:0003743;GO:0005737;GO:0005829;GO:0006413;GO:0008135;GO:0016282;GO:0033290;GO:0042255;GO:0043614 |
| **ENSG00000173692** | **PSMD1** | **0.310340121** | **Q99460** | **PSMD1_HUMAN** | **26S proteasome non-ATPase regulatory subunit 1 (26S proteasome regulatory subunit RPN2) (26S proteasome regulatory subunit S1) (26S proteasome subunit p112)** | **PSMD1** | **953** | **FUNCTION: Component of the 26S proteasome, a multiprotein complex involved in the ATP-dependent degradation of ubiquitinated proteins. This complex plays a key role in the maintenance of protein homeostasis by removing misfolded or damaged proteins, which could impair cellular functions, and by removing proteins whose functions are no longer required. Therefore, the proteasome participates in numerous cellular processes, including cell cycle progression, apoptosis, or DNA damage repair. {ECO:0000269|PubMed:1317798}.** | **proteasome-mediated ubiquitin-dependent protein catabolic process [GO:0043161];regulation of protein catabolic process [GO:0042176]** | **GO:0000502;GO:0005576;GO:0005634;GO:0005654;GO:0005829;GO:0005838;GO:0008540;GO:0016020;GO:0022624;GO:0030234;GO:0031625;GO:0034515;GO:0035578;GO:0042176;GO:0043161** |
| ENSG00000178741 | COX5A | 0.367371066 | P20674 | COX5A_HUMAN | Cytochrome c oxidase subunit 5A, mitochondrial (Cytochrome c oxidase polypeptide Va) | COX5A | 150 | FUNCTION: Component of the cytochrome c oxidase, the last enzyme in the mitochondrial electron transport chain which drives oxidative phosphorylation. The respiratory chain contains 3 multisubunit complexes succinate dehydrogenase (complex II, CII), ubiquinol-cytochrome c oxidoreductase (cytochrome b-c1 complex, complex III, CIII) and cytochrome c oxidase (complex IV, CIV), that cooperate to transfer electrons derived from NADH and succinate to molecular oxygen, creating an electrochemical gradient over the inner membrane that drives transmembrane transport and the ATP synthase. Cytochrome c oxidase is the component of the respiratory chain that catalyzes the reduction of oxygen to water. Electrons originating from reduced cytochrome c in the intermembrane space (IMS) are transferred via the dinuclear copper A center (CU(A)) of subunit 2 and heme A of subunit 1 to the active site in subunit 1, a binuclear center (BNC) formed by heme A3 and copper B (CU(B)). The BNC reduces molecular oxygen to 2 water molecules using 4 electrons from cytochrome c in the IMS and 4 protons from the mitochondrial matrix. {ECO:0000250|UniProtKB:P00427}. | cellular respiration [GO:0045333];mitochondrial electron transport, cytochrome c to oxygen [GO:0006123] | GO:0004129;GO:0005743;GO:0005751;GO:0006123;GO:0009055;GO:0031966;GO:0045333;GO:0046872 |
| ENSG00000181163 | NPM1 | 0.137503524 | P06748 | NPM_HUMAN | Nucleophosmin (NPM) (Nucleolar phosphoprotein B23) (Nucleolar protein NO38) (Numatrin) | NPM1 NPM | 294 | FUNCTION: Involved in diverse cellular processes such as ribosome biogenesis, centrosome duplication, protein chaperoning, histone assembly, cell proliferation, and regulation of tumor suppressors p53/TP53 and ARF. Binds ribosome presumably to drive ribosome nuclear export. Associated with nucleolar ribonucleoprotein structures and bind single-stranded nucleic acids. Acts as a chaperonin for the core histones H3, H2B and H4. Stimulates APEX1 endonuclease activity on apurinic/apyrimidinic (AP) double-stranded DNA but inhibits APEX1 endonuclease activity on AP single-stranded RNA. May exert a control of APEX1 endonuclease activity within nucleoli devoted to repair AP on rDNA and the removal of oxidized rRNA molecules. In concert with BRCA2, regulates centrosome duplication. Regulates centriole duplication: phosphorylation by PLK2 is able to trigger centriole replication. Negatively regulates the activation of EIF2AK2/PKR and suppresses apoptosis through inhibition of EIF2AK2/PKR autophosphorylation. Antagonizes the inhibitory effect of ATF5 on cell proliferation and relieves ATF5-induced G2/M blockade (PubMed:22528486). In complex with MYC enhances the transcription of MYC target genes (PubMed:25956029). May act as chaperonin or cotransporter in the nucleolar localization of transcription termination factor TTF1 (By similarity). {ECO:0000250|UniProtKB:Q61937, ECO:0000269|PubMed:12882984, ECO:0000269|PubMed:16107701, ECO:0000269|PubMed:17015463, ECO:0000269|PubMed:18809582, ECO:0000269|PubMed:19188445, ECO:0000269|PubMed:20352051, ECO:0000269|PubMed:21084279, ECO:0000269|PubMed:22002061, ECO:0000269|PubMed:22528486, ECO:0000269|PubMed:25956029}. | cell volume homeostasis [GO:0006884];cellular response to UV [GO:0034644];cellular senescence [GO:0090398];centrosome cycle [GO:0007098];chromatin remodeling [GO:0006338];DNA repair [GO:0006281];intracellular protein transport [GO:0006886];negative regulation of apoptotic process [GO:0043066];negative regulation of cell population proliferation [GO:0008285];negative regulation of centrosome duplication [GO:0010826];negative regulation of mRNA splicing, via spliceosome [GO:0048025];negative regulation of protein kinase activity by regulation of protein phosphorylation [GO:0044387];nucleocytoplasmic transport [GO:0006913];nucleosome assembly [GO:0006334];positive regulation of cell cycle G2/M phase transition [GO:1902751];positive regulation of cell population proliferation [GO:0008284];positive regulation of centrosome duplication [GO:0010825];positive regulation of DNA-templated transcription [GO:0045893];positive regulation of NF-kappaB transcription factor activity [GO:0051092];positive regulation of protein localization to nucleolus [GO:1904751];positive regulation of protein ubiquitination [GO:0031398];positive regulation of transcription by RNA polymerase II [GO:0045944];positive regulation of translation [GO:0045727];protein localization [GO:0008104];protein stabilization [GO:0050821];regulation of cell growth [GO:0001558];regulation of centriole replication [GO:0046599];regulation of centrosome duplication [GO:0010824];regulation of DNA damage response, signal transduction by p53 class mediator [GO:0043516];regulation of eIF2 alpha phosphorylation by dsRNA [GO:0060735];regulation of endodeoxyribonuclease activity [GO:0032071];regulation of endoribonuclease activity [GO:0060699];regulation of mRNA stability involved in cellular response to UV [GO:1902629];ribosomal large subunit biogenesis [GO:0042273];ribosomal large subunit export from nucleus [GO:0000055];ribosomal small subunit biogenesis [GO:0042274];ribosomal small subunit export from nucleus [GO:0000056];ribosome assembly [GO:0042255];rRNA export from nucleus [GO:0006407];signal transduction [GO:0007165] | GO:0000055;GO:0000056;GO:0001046;GO:0001558;GO:0001652;GO:0003682;GO:0003713;GO:0003723;GO:0004860;GO:0005634;GO:0005654;GO:0005730;GO:0005737;GO:0005813;GO:0005829;GO:0005925;GO:0006281;GO:0006334;GO:0006338;GO:0006407;GO:0006884;GO:0006886;GO:0006913;GO:0007098;GO:0007165;GO:0008104;GO:0008284;GO:0008285;GO:0010824;GO:0010825;GO:0010826;GO:0015934;GO:0015935;GO:0016020;GO:0016607;GO:0019843;GO:0019901;GO:0030957;GO:0031398;GO:0031616;GO:0032071;GO:0032991;GO:0032993;GO:0034644;GO:0042255;GO:0042273;GO:0042274;GO:0042393;GO:0042803;GO:0043023;GO:0043024;GO:0043066;GO:0043516;GO:0044387;GO:0045727;GO:0045893;GO:0045944;GO:0046599;GO:0048025;GO:0050821;GO:0051059;GO:0051082;GO:0051092;GO:0060699;GO:0060735;GO:0090398;GO:0140297;GO:0140693;GO:1902629;GO:1902751;GO:1904751;GO:1990904 |
| ENSG00000182481 | KPNA2 | 0.124328135 | P52292 | IMA1_HUMAN | Importin subunit alpha-1 (Karyopherin subunit alpha-2) (RAG cohort protein 1) (SRP1-alpha) | KPNA2 RCH1 SRP1 | 529 | FUNCTION: Functions in nuclear protein import as an adapter protein for nuclear receptor KPNB1. Binds specifically and directly to substrates containing either a simple or bipartite NLS motif. Docking of the importin/substrate complex to the nuclear pore complex (NPC) is mediated by KPNB1 through binding to nucleoporin FxFG repeats and the complex is subsequently translocated through the pore by an energy requiring, Ran-dependent mechanism. At the nucleoplasmic side of the NPC, Ran binds to importin-beta and the three components separate and importin-alpha and -beta are re-exported from the nucleus to the cytoplasm where GTP hydrolysis releases Ran from importin. The directionality of nuclear import is thought to be conferred by an asymmetric distribution of the GTP- and GDP-bound forms of Ran between the cytoplasm and nucleus. | DNA metabolic process [GO:0006259];entry of viral genome into host nucleus through nuclear pore complex via importin [GO:0075506];NLS-bearing protein import into nucleus [GO:0006607];positive regulation of viral life cycle [GO:1903902];protein import into nucleus [GO:0006606];regulation of DNA recombination [GO:0000018] | GO:0000018;GO:0000139;GO:0003723;GO:0005634;GO:0005654;GO:0005737;GO:0005789;GO:0005829;GO:0006259;GO:0006606;GO:0006607;GO:0008139;GO:0016020;GO:0031965;GO:0042564;GO:0042826;GO:0043657;GO:0061608;GO:0075506;GO:1903902 |
| ENSG00000184575 | XPOT | 0.189033824 | O43592 | XPOT_HUMAN | Exportin-T (Exportin(tRNA)) (tRNA exportin) | XPOT | 962 | FUNCTION: Mediates the nuclear export of aminoacylated tRNAs. In the nucleus binds to tRNA and to the GTPase Ran in its active GTP-bound form. Docking of this trimeric complex to the nuclear pore complex (NPC) is mediated through binding to nucleoporins. Upon transit of a nuclear export complex into the cytoplasm, disassembling of the complex and hydrolysis of Ran-GTP to Ran-GDP (induced by RANBP1 and RANGAP1, respectively) cause release of the tRNA from the export receptor. XPOT then return to the nuclear compartment and mediate another round of transport. The directionality of nuclear export is thought to be conferred by an asymmetric distribution of the GTP- and GDP-bound forms of Ran between the cytoplasm and nucleus. {ECO:0000269|PubMed:12138183, ECO:0000269|PubMed:9512417, ECO:0000269|PubMed:9660920}. | intracellular protein transport [GO:0006886];tRNA export from nucleus [GO:0006409];tRNA re-export from nucleus [GO:0071528] | GO:0000049;GO:0005643;GO:0005654;GO:0005737;GO:0005829;GO:0006409;GO:0006886;GO:0016363;GO:0031267;GO:0071528 |
| ENSG00000185651 | UBE2L3 | 0.014355293 | P68036 | UB2L3_HUMAN | Ubiquitin-conjugating enzyme E2 L3 (EC 2.3.2.23) (E2 ubiquitin-conjugating enzyme L3) (L-UBC) (UbcH7) (Ubiquitin carrier protein L3) (Ubiquitin-conjugating enzyme E2-F1) (Ubiquitin-protein ligase L3) | UBE2L3 UBCE7 UBCH7 | 154 | FUNCTION: Ubiquitin-conjugating enzyme E2 that specifically acts with HECT-type and RBR family E3 ubiquitin-protein ligases. Does not function with most RING-containing E3 ubiquitin-protein ligases because it lacks intrinsic E3-independent reactivity with lysine: in contrast, it has activity with the RBR family E3 enzymes, such as PRKN, RNF31 and ARIH1, that function like RING-HECT hybrids. Accepts ubiquitin from the E1 complex and catalyzes its covalent attachment to other proteins. In vitro catalyzes 'Lys-11'-linked polyubiquitination. Involved in the selective degradation of short-lived and abnormal proteins. Down-regulated during the S-phase it is involved in progression through the cell cycle. Regulates nuclear hormone receptors transcriptional activity. May play a role in myelopoiesis. {ECO:0000269|PubMed:10888878, ECO:0000269|PubMed:15367689, ECO:0000269|PubMed:17003263, ECO:0000269|PubMed:18946090, ECO:0000269|PubMed:19340006, ECO:0000269|PubMed:20061386, ECO:0000269|PubMed:21532592, ECO:0000269|PubMed:35294289}. | cell cycle phase transition [GO:0044770];cell population proliferation [GO:0008283];cellular response to glucocorticoid stimulus [GO:0071385];cellular response to steroid hormone stimulus [GO:0071383];positive regulation of protein targeting to mitochondrion [GO:1903955];positive regulation of protein ubiquitination [GO:0031398];positive regulation of ubiquitin-protein transferase activity [GO:0051443];protein K11-linked ubiquitination [GO:0070979];protein modification process [GO:0036211];protein polyubiquitination [GO:0000209];protein ubiquitination [GO:0016567];regulation of DNA-templated transcription [GO:0006355];ubiquitin-dependent protein catabolic process [GO:0006511] | GO:0000151;GO:0000209;GO:0003713;GO:0003723;GO:0004842;GO:0005524;GO:0005634;GO:0005654;GO:0005737;GO:0005829;GO:0006355;GO:0006511;GO:0008283;GO:0016567;GO:0019899;GO:0031398;GO:0031625;GO:0036211;GO:0044770;GO:0051443;GO:0061631;GO:0070979;GO:0071383;GO:0071385;GO:0097027;GO:1903955 |
| **ENSG00000188846** | **RPL14** | **0.111031312** | **P50914** | **RL14_HUMAN** | **Large ribosomal subunit protein eL14 (60S ribosomal protein L14) (CAG-ISL 7)** | **RPL14** | **215** | **FUNCTION: Component of the large ribosomal subunit (PubMed:12962325, PubMed:23636399, PubMed:32669547). The ribosome is a large ribonucleoprotein complex responsible for the synthesis of proteins in the cell (PubMed:12962325, PubMed:23636399, PubMed:32669547). {ECO:0000269|PubMed:23636399, ECO:0000269|PubMed:32669547, ECO:0000305|PubMed:12962325}.** | **cytoplasmic translation [GO:0002181];ribosomal large subunit biogenesis [GO:0042273];rRNA processing [GO:0006364];translation [GO:0006412]** | **GO:0002181;GO:0003723;GO:0003735;GO:0005737;GO:0005829;GO:0006364;GO:0006412;GO:0014069;GO:0016020;GO:0022625;GO:0022626;GO:0042273;GO:0045296;GO:0070062** |
| ENSG00000189091 | SF3B3 | 0.411426246 | Q15393 | SF3B3_HUMAN | Splicing factor 3B subunit 3 (Pre-mRNA-splicing factor SF3b 130 kDa subunit) (SF3b130) (STAF130) (Spliceosome-associated protein 130) (SAP 130) | SF3B3 KIAA0017 SAP130 | 1217 | FUNCTION: Involved in pre-mRNA splicing as a component of the splicing factor SF3B complex, a constituent of the spliceosome (PubMed:10490618, PubMed:10882114, PubMed:27720643, PubMed:28781166). SF3B complex is required for 'A' complex assembly formed by the stable binding of U2 snRNP to the branchpoint sequence (BPS) in pre-mRNA. Sequence independent binding of SF3A/SF3B complex upstream of the branch site is essential, it may anchor U2 snRNP to the pre-mRNA (PubMed:12234937). May also be involved in the assembly of the 'E' complex (PubMed:10882114). As a component of the minor spliceosome, involved in the splicing of U12-type introns in pre-mRNAs (PubMed:15146077) (Probable). {ECO:0000269|PubMed:10490618, ECO:0000269|PubMed:10882114, ECO:0000269|PubMed:12234937, ECO:0000269|PubMed:15146077, ECO:0000269|PubMed:27720643, ECO:0000269|PubMed:28781166, ECO:0000305|PubMed:33509932}. | mRNA splicing, via spliceosome [GO:0000398];negative regulation of protein catabolic process [GO:0042177];positive regulation of DNA-templated transcription [GO:0045893];regulation of DNA repair [GO:0006282];regulation of RNA splicing [GO:0043484];RNA splicing [GO:0008380];RNA splicing, via transesterification reactions [GO:0000375];U2-type prespliceosome assembly [GO:1903241] | GO:0000124;GO:0000375;GO:0000398;GO:0005634;GO:0005654;GO:0005681;GO:0005684;GO:0005686;GO:0005689;GO:0005730;GO:0006282;GO:0008380;GO:0030620;GO:0042177;GO:0043484;GO:0044877;GO:0045893;GO:0071005;GO:0071013;GO:1903241 |
| ENSG00000196262 | PPIA | 0.23878686 | P62937 | PPIA_HUMAN | Peptidyl-prolyl cis-trans isomerase A (PPIase A) (EC 5.2.1.8) (Cyclophilin A) (Cyclosporin A-binding protein) (Rotamase A) [Cleaved into: Peptidyl-prolyl cis-trans isomerase A, N-terminally processed] | PPIA CYPA | 165 | FUNCTION: Catalyzes the cis-trans isomerization of proline imidic peptide bonds in oligopeptides (PubMed:2001362, PubMed:20676357, PubMed:21245143, PubMed:25678563, PubMed:21593166). Exerts a strong chemotactic effect on leukocytes partly through activation of one of its membrane receptors BSG/CD147, initiating a signaling cascade that culminates in MAPK/ERK activation (PubMed:11943775, PubMed:21245143). Activates endothelial cells (ECs) in a pro-inflammatory manner by stimulating activation of NF-kappa-B and ERK, JNK and p38 MAP-kinases and by inducing expression of adhesion molecules including SELE and VCAM1 (PubMed:15130913). Induces apoptosis in ECs by promoting the FOXO1-dependent expression of CCL2 and BCL2L11 which are involved in EC chemotaxis and apoptosis (PubMed:31063815). In response to oxidative stress, initiates proapoptotic and antiapoptotic signaling in ECs via activation of NF-kappa-B and AKT1 and up-regulation of antiapoptotic protein BCL2 (PubMed:23180369). Negatively regulates MAP3K5/ASK1 kinase activity, autophosphorylation and oxidative stress-induced apoptosis mediated by MAP3K5/ASK1 (PubMed:26095851). Necessary for the assembly of TARDBP in heterogeneous nuclear ribonucleoprotein (hnRNP) complexes and regulates TARDBP binding to RNA UG repeats and TARDBP-dependent expression of HDAC6, ATG7 and VCP which are involved in clearance of protein aggregates (PubMed:25678563). Plays an important role in platelet activation and aggregation (By similarity). Regulates calcium mobilization and integrin ITGA2B:ITGB3 bidirectional signaling via increased ROS production as well as by facilitating the interaction between integrin and the cell cytoskeleton (By similarity). Binds heparan sulfate glycosaminoglycans (PubMed:11943775). Inhibits replication of influenza A virus (IAV) (PubMed:19207730). Inhibits ITCH/AIP4-mediated ubiquitination of matrix protein 1 (M1) of IAV by impairing the interaction of ITCH/AIP4 with M1, followed by the suppression of the nuclear export of M1, and finally reduction of the replication of IAV (PubMed:30328013, PubMed:22347431). {ECO:0000250|UniProtKB:P17742, ECO:0000269|PubMed:11943775, ECO:0000269|PubMed:15130913, ECO:0000269|PubMed:19207730, ECO:0000269|PubMed:2001362, ECO:0000269|PubMed:20676357, ECO:0000269|PubMed:21245143, ECO:0000269|PubMed:21593166, ECO:0000269|PubMed:22347431, ECO:0000269|PubMed:23180369, ECO:0000269|PubMed:25678563, ECO:0000269|PubMed:26095851, ECO:0000269|PubMed:30328013, ECO:0000269|PubMed:31063815};FUNCTION: (Microbial infection) May act as a mediator between human SARS coronavirus nucleoprotein and BSG/CD147 in the process of invasion of host cells by the virus (PubMed:15688292). {ECO:0000269|PubMed:15688292};FUNCTION: (Microbial infection) Stimulates RNA-binding ability of HCV NS5A in a peptidyl-prolyl cis-trans isomerase activity-dependent manner. {ECO:0000269|PubMed:21593166}. | activation of protein kinase B activity [GO:0032148];apoptotic process [GO:0006915];cell adhesion molecule production [GO:0060352];cellular response to oxidative stress [GO:0034599];endothelial cell activation [GO:0042118];leukocyte chemotaxis [GO:0030595];lipid droplet organization [GO:0034389];negative regulation of oxidative stress-induced intrinsic apoptotic signaling pathway [GO:1902176];negative regulation of protein K48-linked ubiquitination [GO:0061944];negative regulation of protein kinase activity [GO:0006469];negative regulation of protein phosphorylation [GO:0001933];negative regulation of stress-activated MAPK cascade [GO:0032873];negative regulation of viral life cycle [GO:1903901];neutrophil chemotaxis [GO:0030593];platelet activation [GO:0030168];platelet aggregation [GO:0070527];positive regulation of MAPK cascade [GO:0043410];positive regulation of NF-kappaB transcription factor activity [GO:0051092];positive regulation of protein dephosphorylation [GO:0035307];positive regulation of protein phosphorylation [GO:0001934];positive regulation of protein secretion [GO:0050714];positive regulation of viral genome replication [GO:0045070];protein folding [GO:0006457];protein peptidyl-prolyl isomerization [GO:0000413];regulation of apoptotic signaling pathway [GO:2001233];regulation of viral genome replication [GO:0045069];viral release from host cell [GO:0019076] | GO:0000413;GO:0001933;GO:0001934;GO:0003723;GO:0003755;GO:0005178;GO:0005576;GO:0005615;GO:0005634;GO:0005737;GO:0005829;GO:0005925;GO:0006457;GO:0006469;GO:0006915;GO:0016018;GO:0016020;GO:0019076;GO:0030168;GO:0030593;GO:0030595;GO:0031982;GO:0032148;GO:0032873;GO:0032991;GO:0034389;GO:0034599;GO:0034774;GO:0035307;GO:0042118;GO:0043410;GO:0045069;GO:0045070;GO:0046790;GO:0050714;GO:0051082;GO:0051092;GO:0060352;GO:0061944;GO:0070062;GO:0070527;GO:1902176;GO:1903901;GO:1904399;GO:1904813;GO:2001233 |
| ENSG00000196305 | IARS1 | 0.056583528 | P41252 | SYIC_HUMAN | Isoleucine--tRNA ligase, cytoplasmic (EC 6.1.1.5) (Isoleucyl-tRNA synthetase) (IRS) (IleRS) | IARS1 IARS | 1262 | FUNCTION: Catalyzes the specific attachment of an amino acid to its cognate tRNA in a 2 step reaction: the amino acid (AA) is first activated by ATP to form AA-AMP and then transferred to the acceptor end of the tRNA. {ECO:0000269|PubMed:8052601}. | isoleucyl-tRNA aminoacylation [GO:0006428];osteoblast differentiation [GO:0001649];tRNA aminoacylation for protein translation [GO:0006418] | GO:0000049;GO:0001649;GO:0002161;GO:0004822;GO:0005524;GO:0005654;GO:0005737;GO:0005829;GO:0006418;GO:0006428;GO:0016020;GO:0017101;GO:0051020;GO:0070062 |
| **ENSG00000198176** | **TFDP1** | **0.028569152** | **Q14186** | **TFDP1_HUMAN** | **Transcription factor Dp-1 (DRTF1-polypeptide 1) (DRTF1) (E2F dimerization partner 1)** | **TFDP1 DP1** | **410** | **FUNCTION: Can stimulate E2F-dependent transcription. Binds DNA cooperatively with E2F family members through the E2 recognition site, 5'-TTTC[CG]CGC-3', found in the promoter region of a number of genes whose products are involved in cell cycle regulation or in DNA replication (PubMed:8405995, PubMed:7739537). The E2F1:DP complex appears to mediate both cell proliferation and apoptosis. Blocks adipocyte differentiation by repressing CEBPA binding to its target gene promoters (PubMed:20176812). {ECO:0000269|PubMed:20176812, ECO:0000269|PubMed:7739537, ECO:0000269|PubMed:8405995}.** | **anoikis [GO:0043276];epidermis development [GO:0008544];negative regulation of fat cell proliferation [GO:0070345];positive regulation of DNA-binding transcription factor activity [GO:0051091];positive regulation of G1/S transition of mitotic cell cycle [GO:1900087];positive regulation of transcription by RNA polymerase II [GO:0045944];regulation of DNA biosynthetic process [GO:2000278];regulation of transcription by RNA polymerase II [GO:0006357];regulation of transcription involved in G1/S transition of mitotic cell cycle [GO:0000083];transcription by RNA polymerase II [GO:0006366]** | **GO:0000083;GO:0000785;GO:0000981;GO:0000987;GO:0003700;GO:0005634;GO:0005654;GO:0005737;GO:0005829;GO:0006357;GO:0006366;GO:0008544;GO:0019904;GO:0035189;GO:0043276;GO:0045944;GO:0051091;GO:0070345;GO:0090575;GO:0140297;GO:1900087;GO:2000278** |
| ENSG00000204392 | LSM2 | 0.056583528 | Q9Y333 | LSM2_HUMAN | U6 snRNA-associated Sm-like protein LSm2 (Protein G7b) (Small nuclear ribonuclear protein D homolog) (snRNP core Sm-like protein Sm-x5) | LSM2 C6orf28 G7B | 95 | FUNCTION: Plays a role in pre-mRNA splicing as component of the U4/U6-U5 tri-snRNP complex that is involved in spliceosome assembly, and as component of the precatalytic spliceosome (spliceosome B complex) (PubMed:28781166). The heptameric LSM2-8 complex binds specifically to the 3'-terminal U-tract of U6 snRNA (PubMed:10523320). {ECO:0000269|PubMed:10523320, ECO:0000269|PubMed:28781166}. | mRNA catabolic process [GO:0006402];mRNA splicing, via spliceosome [GO:0000398];spliceosomal tri-snRNP complex assembly [GO:0000244] | GO:0000244;GO:0000398;GO:0000932;GO:0003723;GO:0005634;GO:0005654;GO:0005688;GO:0005737;GO:0005829;GO:0006402;GO:0017070;GO:0031267;GO:0046540;GO:0071005;GO:0071011;GO:0071013;GO:0120115;GO:1990726 |
| ENSG00000204628 | RACK1 | 0.22650853 | P63244 | RACK1_HUMAN | Small ribosomal subunit protein RACK1 (Cell proliferation-inducing gene 21 protein) (Guanine nucleotide-binding protein subunit beta-2-like 1) (Guanine nucleotide-binding protein subunit beta-like protein 12.3) (Human lung cancer oncogene 7 protein) (HLC-7) (Receptor for activated C kinase) (Receptor of activated protein C kinase 1) [Cleaved into: Small ribosomal subunit protein RACK1, N-terminally processed (Guanine nucleotide-binding protein subunit beta-2-like 1, N-terminally processed) (Receptor of activated protein C kinase 1, N-terminally processed)] | RACK1 GNB2L1 HLC7 PIG21 | 317 | FUNCTION: Scaffolding protein involved in the recruitment, assembly and/or regulation of a variety of signaling molecules. Interacts with a wide variety of proteins and plays a role in many cellular processes. Component of the 40S ribosomal subunit involved in translational repression (PubMed:23636399). Involved in the initiation of the ribosome quality control (RQC), a pathway that takes place when a ribosome has stalled during translation, by promoting ubiquitination of a subset of 40S ribosomal subunits (PubMed:28132843). Binds to and stabilizes activated protein kinase C (PKC), increasing PKC-mediated phosphorylation. May recruit activated PKC to the ribosome, leading to phosphorylation of EIF6. Inhibits the activity of SRC kinases including SRC, LCK and YES1. Inhibits cell growth by prolonging the G0/G1 phase of the cell cycle. Enhances phosphorylation of BMAL1 by PRKCA and inhibits transcriptional activity of the BMAL1-CLOCK heterodimer. Facilitates ligand-independent nuclear translocation of AR following PKC activation, represses AR transactivation activity and is required for phosphorylation of AR by SRC. Modulates IGF1R-dependent integrin signaling and promotes cell spreading and contact with the extracellular matrix. Involved in PKC-dependent translocation of ADAM12 to the cell membrane. Promotes the ubiquitination and proteasome-mediated degradation of proteins such as CLEC1B and HIF1A. Required for VANGL2 membrane localization, inhibits Wnt signaling, and regulates cellular polarization and oriented cell division during gastrulation. Required for PTK2/FAK1 phosphorylation and dephosphorylation. Regulates internalization of the muscarinic receptor CHRM2. Promotes apoptosis by increasing oligomerization of BAX and disrupting the interaction of BAX with the anti-apoptotic factor BCL2L. Inhibits TRPM6 channel activity. Regulates cell surface expression of some GPCRs such as TBXA2R. Plays a role in regulation of FLT1-mediated cell migration. Involved in the transport of ABCB4 from the Golgi to the apical bile canalicular membrane (PubMed:19674157). Promotes migration of breast carcinoma cells by binding to and activating RHOA (PubMed:20499158). Acts as an adapter for the dephosphorylation and inactivation of AKT1 by promoting recruitment of PP2A phosphatase to AKT1 (By similarity). {ECO:0000250\|UniProtKB:P68040, ECO:0000269\|PubMed:11884618, ECO:0000269\|PubMed:12589061, ECO:0000269\|PubMed:12958311, ECO:0000269\|PubMed:17108144, ECO:0000269\|PubMed:17244529, ECO:0000269\|PubMed:17956333, ECO:0000269\|PubMed:18088317, ECO:0000269\|PubMed:18258429, ECO:0000269\|PubMed:18621736, ECO:0000269\|PubMed:19423701, ECO:0000269\|PubMed:19674157, ECO:0000269\|PubMed:19785988, ECO:0000269\|PubMed:20499158, ECO:0000269\|PubMed:20541605, ECO:0000269\|PubMed:20573744, ECO:0000269\|PubMed:20976005, ECO:0000269\|PubMed:21212275, ECO:0000269\|PubMed:21347310, ECO:0000269\|PubMed:23636399, ECO:0000269\|PubMed:28132843, ECO:0000269\|PubMed:9584165};FUNCTION: (Microbial infection) Binds to Y.pseudotuberculosis yopK which leads to inhibition of phagocytosis and survival of bacteria following infection of host cells. {ECO:0000269\|PubMed:21347310};FUNCTION: (Microbial infection) Enhances phosphorylation of HIV-1 Nef by PKCs. {ECO:0000269\|PubMed:11312657};FUNCTION: (Microbial infection) In case of poxvirus infection, remodels the ribosomes so that they become optimal for the viral mRNAs (containing poly-A leaders) translation but not for host mRNAs. {ECO:0000269\|PubMed:28636603};FUNCTION: (Microbial infection) Contributes to the cap-independent internal ribosome entry site (IRES)-mediated translation by some RNA viruses. {ECO:0000269\|PubMed:25416947}. | activation of cysteine-type endopeptidase activity involved in apoptotic process [GO:0006919];cell cycle [GO:0007049];cellular response to glucose stimulus [GO:0071333];cellular response to growth factor stimulus [GO:0071363];cytoplasmic translation [GO:0002181];gastrulation [GO:0007369];negative regulation of cell growth [GO:0030308];negative regulation of endoplasmic reticulum unfolded protein response [GO:1900102];negative regulation of gene expression [GO:0010629];negative regulation of intrinsic apoptotic signaling pathway in response to hydrogen peroxide [GO:1903751];negative regulation of peptidyl-serine phosphorylation [GO:0033137];negative regulation of phagocytosis [GO:0050765];negative regulation of phosphatidylinositol 3-kinase signaling/protein kinase B signal transduction [GO:0051898];negative regulation of protein binding [GO:0032091];negative regulation of smoothened signaling pathway [GO:0045879];negative regulation of translation [GO:0017148];negative regulation of Wnt signaling pathway [GO:0030178];pigmentation [GO:0043473];positive regulation of apoptotic process [GO:0043065];positive regulation of cell migration [GO:0030335];positive regulation of cyclic-nucleotide phosphodiesterase activity [GO:0051343];positive regulation of gastrulation [GO:2000543];positive regulation of Golgi to plasma membrane protein transport [GO:0042998];positive regulation of GTPase activity [GO:0043547];positive regulation of intrinsic apoptotic signaling pathway [GO:2001244];positive regulation of mitochondrial depolarization [GO:0051901];positive regulation of proteasomal ubiquitin-dependent protein catabolic process [GO:0032436];positive regulation of protein phosphorylation [GO:0001934];positive regulation of protein-containing complex assembly [GO:0031334];protein ubiquitination [GO:0016567];regulation of cell cycle [GO:0051726];regulation of cell division [GO:0051302];regulation of establishment of cell polarity [GO:2000114];regulation of protein localization [GO:0032880];rescue of stalled ribosome [GO:0072344];rhythmic process [GO:0048511] | GO:0001891;GO:0001934;GO:0002181;GO:0003723;GO:0005080;GO:0005102;GO:0005634;GO:0005654;GO:0005737;GO:0005739;GO:0005829;GO:0006919;GO:0007049;GO:0007369;GO:0008200;GO:0008656;GO:0010629;GO:0015935;GO:0016567;GO:0017148;GO:0019899;GO:0019903;GO:0022627;GO:0030178;GO:0030292;GO:0030308;GO:0030332;GO:0030335;GO:0030425;GO:0030496;GO:0030971;GO:0031334;GO:0032091;GO:0032436;GO:0032880;GO:0033137;GO:0035591;GO:0042169;GO:0042803;GO:0042998;GO:0043022;GO:0043025;GO:0043065;GO:0043204;GO:0043473;GO:0043547;GO:0045182;GO:0045296;GO:0045879;GO:0048471;GO:0048511;GO:0050765;GO:0051302;GO:0051343;GO:0051434;GO:0051726;GO:0051898;GO:0051901;GO:0060090;GO:0070062;GO:0071333;GO:0071363;GO:0072344;GO:1900102;GO:1903751;GO:1990630;GO:2000114;GO:2000543;GO:2001244 |
| **ENSG00000205937** | **RNPS1** | **0.150559677** | **Q15287** | **RNPS1_HUMAN** | **RNA-binding protein with serine-rich domain 1 (SR-related protein LDC2)** | **RNPS1 LDC2** | **305** | **FUNCTION: Part of pre- and post-splicing multiprotein mRNP complexes. Auxiliary component of the splicing-dependent multiprotein exon junction complex (EJC) deposited at splice junction on mRNAs. The EJC is a dynamic structure consisting of core proteins and several peripheral nuclear and cytoplasmic associated factors that join the complex only transiently either during EJC assembly or during subsequent mRNA metabolism. Component of the ASAP and PSAP complexes which bind RNA in a sequence-independent manner and are proposed to be recruited to the EJC prior to or during the splicing process and to regulate specific excision of introns in specific transcription subsets. The ASAP complex can inhibit RNA processing during in vitro splicing reactions. The ASAP complex promotes apoptosis and is disassembled after induction of apoptosis. Enhances the formation of the ATP-dependent A complex of the spliceosome. Involved in both constitutive splicing and, in association with SRP54 and TRA2B/SFRS10, in distinctive modulation of alternative splicing in a substrate-dependent manner. Involved in the splicing modulation of BCL2L1/Bcl-X (and probably other apoptotic genes);specifically inhibits formation of proapoptotic isoforms such as Bcl-X(S);the activity is different from the established EJC assembly and function. Participates in mRNA 3'-end cleavage. Involved in UPF2-dependent nonsense-mediated decay (NMD) of mRNAs containing premature stop codons. Also mediates increase of mRNA abundance and translational efficiency. Binds spliced mRNA 20-25 nt upstream of exon-exon junctions. {ECO:0000269\|PubMed:10449421, ECO:0000269\|PubMed:11546874, ECO:0000269\|PubMed:12665594, ECO:0000269\|PubMed:12944400, ECO:0000269\|PubMed:14729963, ECO:0000269\|PubMed:14752011, ECO:0000269\|PubMed:15684395, ECO:0000269\|PubMed:16209946, ECO:0000269\|PubMed:17586820, ECO:0000269\|PubMed:22203037}.** | **DNA-templated transcription [GO:0006351];mRNA splicing, via spliceosome [GO:0000398];negative regulation of mRNA splicing, via spliceosome [GO:0048025];nuclear-transcribed mRNA catabolic process, nonsense-mediated decay [GO:0000184];positive regulation of apoptotic process [GO:0043065];regulation of alternative mRNA splicing, via spliceosome [GO:0000381];RNA splicing [GO:0008380]** | **GO:0000184;GO:0000381;GO:0000398;GO:0003723;GO:0003730;GO:0005634;GO:0005654;GO:0005737;GO:0005829;GO:0006351;GO:0008380;GO:0016607;GO:0043065;GO:0048025;GO:0061574** |
| ENSG00000213585 | VDAC1 | 0.150559677 | P21796 | VDAC1_HUMAN | Voltage-dependent anion-selective channel protein 1 (VDAC-1) (hVDAC1) (Outer mitochondrial membrane protein porin 1) (Plasmalemmal porin) (Porin 31HL) (Porin 31HM) | VDAC1 VDAC | 283 | FUNCTION: Forms a channel through the mitochondrial outer membrane and also the plasma membrane. The channel at the outer mitochondrial membrane allows diffusion of small hydrophilic molecules;in the plasma membrane it is involved in cell volume regulation and apoptosis. It adopts an open conformation at low or zero membrane potential and a closed conformation at potentials above 30-40 mV. The open state has a weak anion selectivity whereas the closed state is cation-selective (PubMed:11845315, PubMed:18755977, PubMed:20230784, PubMed:8420959). Binds various signaling molecules, including the sphingolipid ceramide, the phospholipid phosphatidylcholine, and the sterol cholesterol (PubMed:31015432). In depolarized mitochondria, acts downstream of PRKN and PINK1 to promote mitophagy or prevent apoptosis;polyubiquitination by PRKN promotes mitophagy, while monoubiquitination by PRKN decreases mitochondrial calcium influx which ultimately inhibits apoptosis (PubMed:32047033). May participate in the formation of the permeability transition pore complex (PTPC) responsible for the release of mitochondrial products that triggers apoptosis (PubMed:15033708, PubMed:25296756). May mediate ATP export from cells (PubMed:30061676). {ECO:0000269\|PubMed:11845315, ECO:0000269\|PubMed:15033708, ECO:0000269\|PubMed:18755977, ECO:0000269\|PubMed:20230784, ECO:0000269\|PubMed:25296756, ECO:0000269\|PubMed:30061676, ECO:0000269\|PubMed:31015432, ECO:0000269\|PubMed:32047033, ECO:0000269\|PubMed:8420959}. | apoptotic process [GO:0006915];behavioral fear response [GO:0001662];epithelial cell differentiation [GO:0030855];learning [GO:0007612];monoatomic anion transport [GO:0006820];negative regulation of apoptotic process [GO:0043066];negative regulation of calcium import into the mitochondrion [GO:0110099];negative regulation of reactive oxygen species metabolic process [GO:2000378];neuron-neuron synaptic transmission [GO:0007270];positive regulation of parkin-mediated stimulation of mitophagy in response to mitochondrial depolarization [GO:1905091];pyruvate metabolic process [GO:0006090];regulation of autophagy of mitochondrion [GO:1903146] | GO:0001662;GO:0005634;GO:0005739;GO:0005741;GO:0005757;GO:0005886;GO:0006090;GO:0006820;GO:0006915;GO:0007270;GO:0007612;GO:0008308;GO:0015288;GO:0015485;GO:0016020;GO:0019901;GO:0030855;GO:0031210;GO:0031966;GO:0042645;GO:0042802;GO:0043066;GO:0044325;GO:0045121;GO:0045202;GO:0046930;GO:0070062;GO:0097001;GO:0110099;GO:1903146;GO:1905091;GO:2000378 |
| ENSG00000215021 | PHB2 | 0.124328135 | Q99623 | PHB2_HUMAN | Prohibitin-2 (B-cell receptor-associated protein BAP37) (D-prohibitin) (Repressor of estrogen receptor activity) | PHB2 BAP REA | 299 | FUNCTION: Protein with pleiotropic attributes mediated in a cell-compartment- and tissue-specific manner, which include the plasma membrane-associated cell signaling functions, mitochondrial chaperone, and transcriptional co-regulator of transcription factors and sex steroid hormones in the nucleus. {ECO:0000269\|PubMed:10359819, ECO:0000269\|PubMed:11302691, ECO:0000269\|PubMed:20959514, ECO:0000269\|PubMed:24003225, ECO:0000269\|PubMed:28017329, ECO:0000269\|PubMed:31522117};FUNCTION: In the mitochondria, together with PHB, forms large ring complexes (prohibitin complexes) in the inner mitochondrial membrane (IMM) and functions as chaperone protein that stabilizes mitochondrial respiratory enzymes and maintains mitochondrial integrity in the IMM, which is required for mitochondrial morphogenesis, neuronal survival, and normal lifespan (Probable). The prohibitin complex, with DNAJC19, regulates cardiolipin remodeling and the protein turnover of OMA1 in a cardiolipin-binding manner (By similarity). Also regulates cytochrome-c oxidase assembly (COX) and mitochondrial respiration (PubMed:20959514, PubMed:11302691). Binding to sphingoid 1-phosphate (SPP) modulates its regulator activity (PubMed:20959514, PubMed:11302691). Has a key role of mitophagy receptor involved in targeting mitochondria for autophagic degradation (PubMed:28017329). Involved in mitochondrial-mediated antiviral innate immunity, activates RIG-I-mediated signal transduction and production of IFNB1 and pro-inflammatory cytokine IL6 (PubMed:31522117). {ECO:0000250\|UniProtKB:O35129, ECO:0000269\|PubMed:11302691, ECO:0000269\|PubMed:20959514, ECO:0000269\|PubMed:28017329, ECO:0000269\|PubMed:31522117, ECO:0000305\|PubMed:25904163};FUNCTION: In the nucleus, serves as transcriptional co-regulator (Probable). Acts as a mediator of transcriptional repression by nuclear hormone receptors via recruitment of histone deacetylases. Functions as an estrogen receptor (ER)-selective coregulator that potentiates the inhibitory activities of antiestrogens and represses the activity of estrogens. Competes with NCOA1 for modulation of ER transcriptional activity (By similarity). {ECO:0000250\|UniProtKB:O35129, ECO:0000305\|PubMed:25904163};FUNCTION: In the plasma membrane, is involved in IGFBP6-induced cell migration (PubMed:24003225). Cooperates with CD86 to mediate CD86-signaling in B lymphocytes that regulates the level of IgG1 produced through the activation of distal signaling intermediates. Upon CD40 engagement, required to activate NF-kappa-B signaling pathway via phospholipase C and protein kinase C activation (By similarity). {ECO:0000250\|UniProtKB:O35129, ECO:0000269\|PubMed:24003225};FUNCTION: (Microbial infection) Involved in human enterovirus 71/EV-71 infection by enhancing the autophagy mechanism during the infection. {ECO:0000269\|PubMed:32276428}. | activation of phospholipase C activity [GO:0007202];activation of protein kinase C activity [GO:1990051];antiviral innate immune response [GO:0140374];B cell activation [GO:0042113];CD40 signaling pathway [GO:0023035];cell migration [GO:0016477];cellular response to hypoxia [GO:0071456];cellular response to retinoic acid [GO:0071300];intracellular estrogen receptor signaling pathway [GO:0030520];mammary gland alveolus development [GO:0060749];mammary gland branching involved in thelarche [GO:0060744];mammary gland epithelial cell proliferation [GO:0033598];mitochondrion organization [GO:0007005];mitophagy [GO:0000423];negative regulation of apoptotic process [GO:0043066];negative regulation of DNA-binding transcription factor activity [GO:0043433];negative regulation of DNA-templated transcription [GO:0045892];negative regulation of intracellular estrogen receptor signaling pathway [GO:0033147];negative regulation of mammary gland epithelial cell proliferation [GO:0033600];positive regulation of cell cycle G1/S phase transition [GO:1902808];positive regulation of DNA-binding transcription factor activity [GO:0051091];positive regulation of ERK1 and ERK2 cascade [GO:0070374];positive regulation of exit from mitosis [GO:0031536];positive regulation of immunoglobulin production [GO:0002639];positive regulation of non-canonical NF-kappaB signal transduction [GO:1901224];protein import into nucleus [GO:0006606];protein stabilization [GO:0050821];regulation of branching involved in mammary gland duct morphogenesis [GO:0060762];regulation of cardiolipin metabolic process [GO:1900208];regulation of cytochrome-c oxidase activity [GO:1904959];RIG-I signaling pathway [GO:0039529];sister chromatid cohesion [GO:0007062] | GO:0000423;GO:0002639;GO:0005634;GO:0005737;GO:0005739;GO:0005741;GO:0005743;GO:0005886;GO:0006606;GO:0007005;GO:0007062;GO:0007202;GO:0009986;GO:0014069;GO:0016363;GO:0016477;GO:0023035;GO:0030331;GO:0030424;GO:0030520;GO:0031536;GO:0032991;GO:0033147;GO:0033218;GO:0033598;GO:0033600;GO:0035632;GO:0039529;GO:0042113;GO:0042802;GO:0042803;GO:0043066;GO:0043433;GO:0045892;GO:0046625;GO:0046982;GO:0048786;GO:0050821;GO:0051091;GO:0060744;GO:0060749;GO:0060762;GO:0070374;GO:0071300;GO:0071456;GO:0071944;GO:0098978;GO:0098982;GO:0140374;GO:1900208;GO:1901224;GO:1902808;GO:1904959;GO:1990051 |

**Uncropped western blots**
